# Base-Induced Sulfoxide-Sulfenate Rearrangement of 2-Sulfinyl Dienes for the Regio- and Stereoselective Synthesis of Enantioenriched Dienyl Diols

**DOI:** 10.1021/acs.joc.2c02931

**Published:** 2023-03-03

**Authors:** Marina Velado, Manuel Martinović, Inés Alonso, Mariola Tortosa, Roberto Fernández de la Pradilla, Alma Viso

**Affiliations:** † Instituto de Química Orgánica General (IQOG), CSIC, Juan de la Cierva 3, 28006 Madrid, Spain; ‡ Organic Chemistry Department and Center for Innovation in Advanced Chemistry (ORFEO-CINQA) Universidad Autónoma de Madrid (UAM), 28049 Madrid, Spain; § Institute for Advanced Research in Chemical Sciences (IAdChem), Universidad Autónoma de Madrid (UAM), 28049 Madrid, Spain

## Abstract

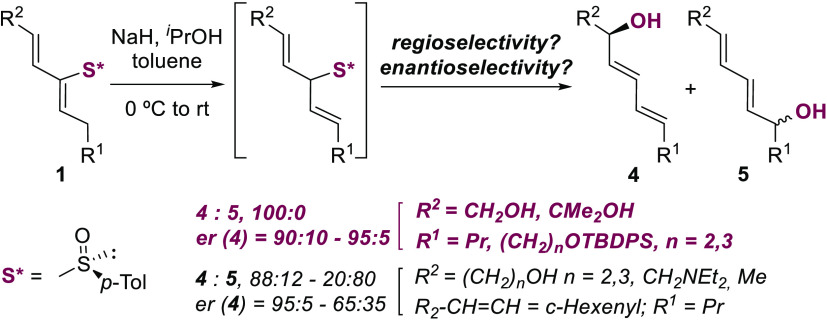

The base-induced [2,3]-sigmatropic rearrangement of a series of enantiopure 2-sulfinyl dienes has been examined and optimized using a combination of NaH and ^*i*^PrOH. The reaction takes place by allylic deprotonation of the 2-sulfinyl diene to give a bis-allylic sulfoxide anion intermediate that after protonation undergoes sulfoxide-sulfenate rearrangement. Different substitution at the starting 2-sulfinyl dienes has allowed us to study the rearrangement finding that a terminal allylic alcohol is determinant to achieve complete regioselectivity and high enantioselectivities (90:10–95:5) with the sulfoxide as the only element of stereocontrol. Density functional theory (DFT) calculations provide an interpretation of these results.

## Introduction

**Scheme 1:**
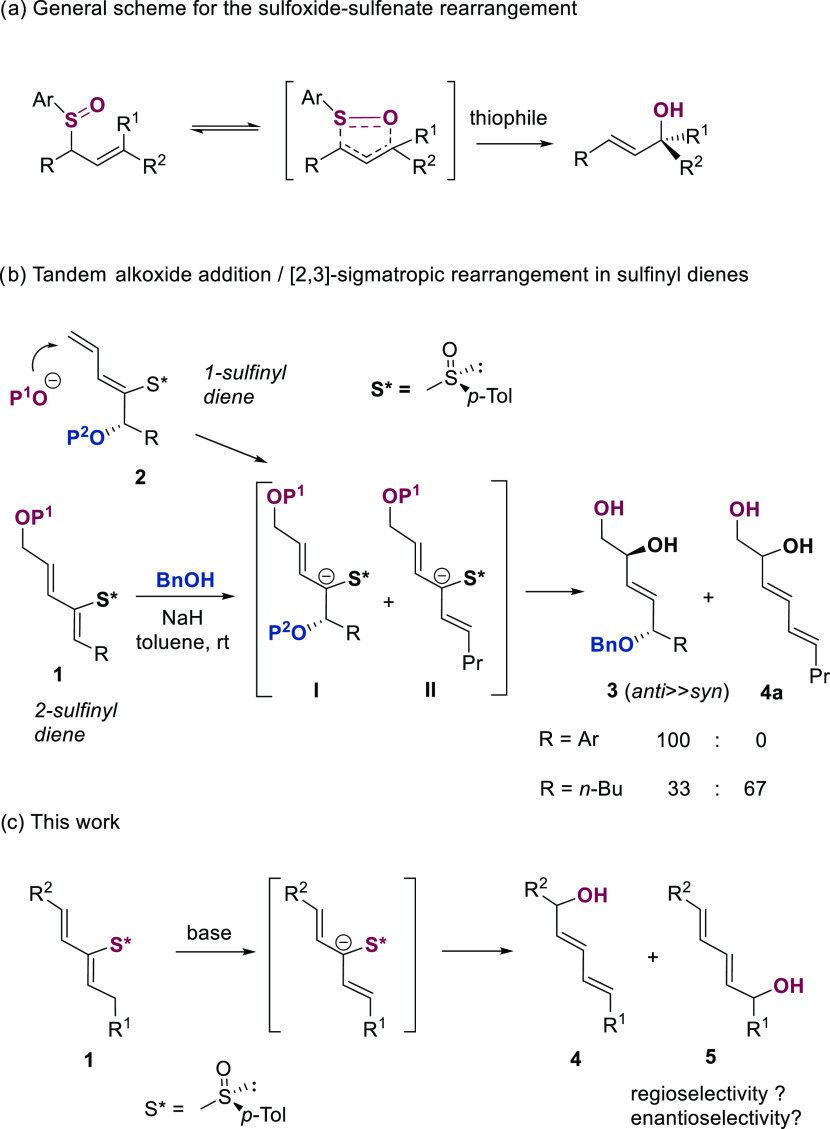
Sulfoxide-Sulfenate Rearrangement and Our Work

The rich chemistry of allylic sulfoxides along with their presence in natural products places them among the more interesting organosulfur motifs.^1^ One of their more versatile reactions is the [2,3]-sigmatropic rearrangement that allows for the transformation of allylic sulfoxides into allylic sulfenates and subsequently into allylic alcohols, in the presence of a suitable thiophile to cleave the sulfenate S–O bond. In this process, the stereochemistry is transferred from the C–S bond of the allylic sulfoxide to the new C–O bond in the final product (Scheme 1a).^2^ This reaction commonly referred to as sulfoxide-sulfenate or Mislow–Evans rearrangement has attracted the attention of chemists from a mechanistic^3^ and a synthetic standpoint.^4^ In particular, the [2,3]-sigmatropic rearrangement of allylic sulfoxides is a versatile tool in the synthesis of numerous target compounds because it can easily be coupled to other reactions such as 1,2-elimination, other sigmatropic rearrangements, Knoevenagel condensation (SPAC) and [4+2] cycloadditions.^2a^

In this context, during the last years, our group has developed the diastereoselective Michael-type addition to 1- and 2-sulfinyl dienes (**1** and **2**) to generate transient allylic sulfoxides **I** that evolved through [2,3]-sigmatropic rearrangement (Scheme 1b). We have applied this tandem process intramolecularly for the stereoselective synthesis of enantiopure functionalized dihydropyrans^5^ and tetrahydropyridinols^6^ as well as intermolecularly for the synthesis of acyclic 2-ene-1,4-diols,^7^ 1,4-aminoalcohols,^8^ and 1,4-hydroxysulfides^9^ with a high degree of stereocontrol.

Within this study and upon examination of the addition of alkoxides (NaOBn) to 2-sulfinyl dienes having alkyl substituents (**1a**, R = *n*-Bu), we identified the expected product **3** along with dienyl diol **4**, presumably formed by allylic deprotonation of the 2-sulfinyl diene to give a bis-allylic sulfoxide anion intermediate (**II**) that after protonation underwent a completely regioselective sulfoxide-sulfenate rearrangement toward the allylic alcohol double bond. Interestingly, **4** was isolated as a single product by treatment with base in the absence of alkoxide and lowering the reaction temperature (−40 °C to rt) with moderate yield (57%) and 92:8 enantiomeric ratio,^7^ remarkably high for the sulfoxide as the only element of stereocontrol. Encouraged by our previous result, we envisioned that treating with base a group of 2-sulfinyl-dienes **1**, selected by addressing the nature of R^1^ and R^2^, the stereochemistry of the double bonds, and the degree of substitution (Table 1, R^3^, R^4^), would give us the opportunity to study the regioselectivity of the [2,3]-sigmatropic rearrangement of bis-allylic sulfoxide intermediates and to explore the synthesis of dienyl diols **4**, valuable compounds in natural products and as synthetic intermediates^10^ (Scheme 1c). In spite of the widespread successful use of the sulfoxide-sulfenate rearrangement in synthesis, we could not find studies where both the regio- and enantioselectivities of the process were examined. Herein, we disclose a full account of our findings.

**Table 1:**
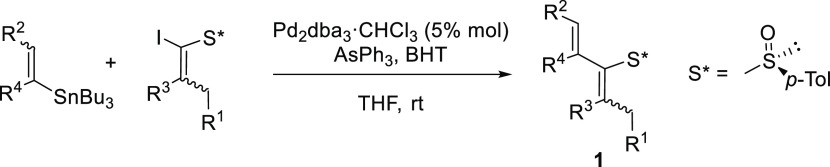

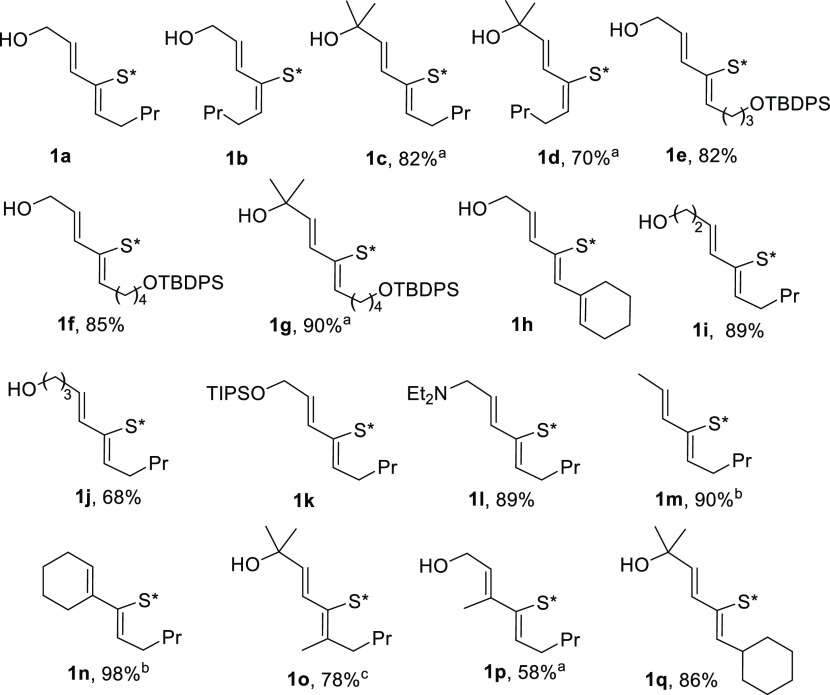
Preparation of Starting 2-Sulfinyl dienes

a Pd(CH_3_CN)_2_Cl_2_ (10% mol), *N*,*N*-dimethylformamide (DMF), rt.

b Pd(PPh_3_)_4_ (10% mol), CsF (4 equiv), vinyl boronic acid (2 equiv).

c 60 °C instead of rt.

## Results and Discussion

### Synthetic Studies

Selected 2-sulfinyl dienes were synthesized by the general and efficient Stille coupling of vinyl stannanes or boronates with (*Z*)- or (*E*)-iodo vinyl sulfoxides previously obtained from (−)-menthyl *p*-toluenesulfinate in three steps (Table 1).^11^ We prepared 2-sulfinyl dienes having a hydroxyl group at R^2^ (**1a–1j**, and **1o**-**q**) with different degree of substitution (**1c**, **1d**, **1g**, **1o**-**q**) and distance from sulfoxide to the OH group (**1i**, **1j**). Substrates with a protected hydroxyl group (**1k**), a tertiary amine (**1l**), or lacking the OH (**1m** and **1n**) in R^2^ were also chosen to examine regio- and enantioselectivities of the rearrangement. Finally, we also considered variations at R^1^ by introducing an additional double bond (**1h**), functionalized alkyl chains (**1e**–**1g**), and also by preparing (*E,E*)- and (*E,Z*)-dienes (**1a** and **1c***vs***1b** and **1d**) to examine the effect of the stereochemistry of the sulfinyl diene on the outcome of the process.

To optimize the conditions of the base-promoted [2,3]-rearrangement of 2-sulfinyl diene **1a**, we examined its reactivity with an excess of NaH in toluene (Table 2, entries 1–3) finding that **4a** was formed with complete regioselectivity and that both isolated yield and reactivity decreased by lowering the reaction temperature while the enantiomeric ratio increased from 86:14 to 92:8. Er dropped to 70:30 using tetrahydrofuran (THF) as solvent (Table 2, entry 4). At this point, we examined the influence of external thiophiles such as Et_2_NH using different bases (Table 1, entries 5–8), but we did not observe any improvement in yield or er. In our experience,^7^ alkoxides are efficient thiophiles in related sulfoxide-sulfenate rearrangements; therefore, we added 2 equiv of ^*i*^PrOH to generate hindered NaO^*i*^Pr that would not undergo Michael addition to **1a** but could improve the yield of the reaction. After some experimentation with different bases (Table 2, entries 9–11), we found that the combination NaH/^*i*^PrOH produced a notable increase in reactivity with a higher yield (62%) and 93:7 er at rt.

**Table 2:**
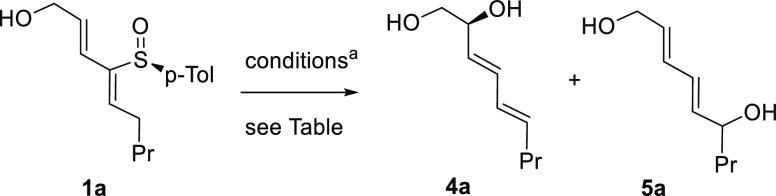
Optimization of the Base-Promoted Sulfoxide-Sulfenate Rearrangement

entry	base	*T*/*t*	thiophile	**4a**:**5a**	yield (**4a**)^b^	er^c^
1	NaH^d^	rt/1 h		100:0	61%	86:14
2	NaH^d^	0 °C to rt 3 h		100:0	50%	91:9
3	NaH^d^	–40 °C to rt, 21 h		100:0	57%	92:8
4	NaH^d^^,^^e^	–40 °C to rt, 21 h		100:0	36%	70:30
5	NaH^d^	0 °C to rt, 20 h	Et_2_NH^f^	100:0	47%	88:12
6	KH^d^	0 °C to rt 4 h	Et_2_NH^f^	g	29%	ND
7	K^*t*^BuO^d^	0 °C to rt 4 h	Et_2_NH^f^	g	20%	ND
8	KHMDS^d^	0 °C to rt 4 h	Et_2_NH^f^	100:0	50%	88:12
9	P_2_^*t*^Bu^d^	0 °C to rt 6 h	*i*PrOH^i^	80:20	25%	60:40
10	DBU^d^	rt to 85 °C, 16 h	^*i*^PrOH^i^	100:0	40%	64:36
**11**	**NaH**^h^	**0 °C to rt, 2 h**	^***i***^**PrOH**^i^	**100:0**	**62%**	**93:7**

a Toluene was used as solvent.

b Isolated yield of pure **4a**.

c Er and absolute configuration of **4a** were determined by ^1^H NMR as (*S*)-MPA esters **6a** and **7a**.

d 4 equiv of base.

e THF was used as solvent.

f 8 equiv of thiophile.

g Complex mixture.

h 6 equiv of base.

i 2 equiv of thiophile.

Subsequently, we applied the optimized conditions to the set of 2-sulfinyl dienes previously synthesized (Table 1) and the results are gathered in Table 3. In general, (*E,Z*)-2-sulfinyl dienes with a hydroxymethyl in R^2^ and a simple alkyl group in R^1^ (**1a**, **1e**, **1f**) reacted in 2 h with complete regioselectivity toward the allylic alcohol fragment affording **4a**, **4c**, and **4d** with good yields and enantiomeric ratios ranging from 93:7 to 90:10. Interestingly, increasing the steric hindrance at R^2^ by installing a tertiary carbinol (**1c**, **1g**) preserves the good results (**4b**, **4e**). A parallel behavior was observed when (*E,E*)-2-sulfinyl dienes were submitted to the reaction conditions, leading to the same dienyl diols with almost identical yield and enantiomeric ratio (**4a** from (*E,E*)-**1b** and **4b** from (*E,E*)-**1d**). We also observed complete regioselectivity when an additional conjugated double bond is present in the starting material (**1h**); however, a decay in yield and enantiomeric ratio was observed (**4f**). Increasing the distance between sulfoxide and OH (**1i**) resulted in a slower reaction (21 h) and a decrease in regioselectivity (**4g**:**5g**, 88:12) maintaining a good er only for **4g** (95:5). A similar trend was found for **1j** with a sluggish reaction and a higher decay in yield and regioselectivity affording a complex mixture that decomposed under purification giving only a small amount of **4h**. Installation of a TIPS protecting group (**1k**) led to a mixture of products where small amounts of isomeric sulfinyl trienes, resulting from the elimination of the silyloxy moiety, were tentatively identified.

**Table 3:**
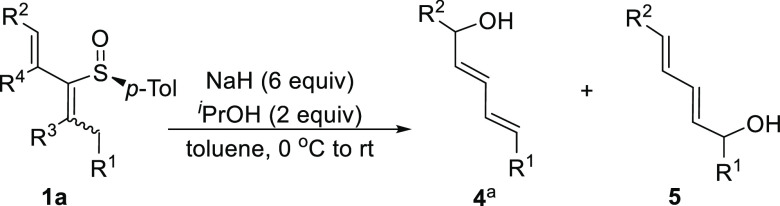

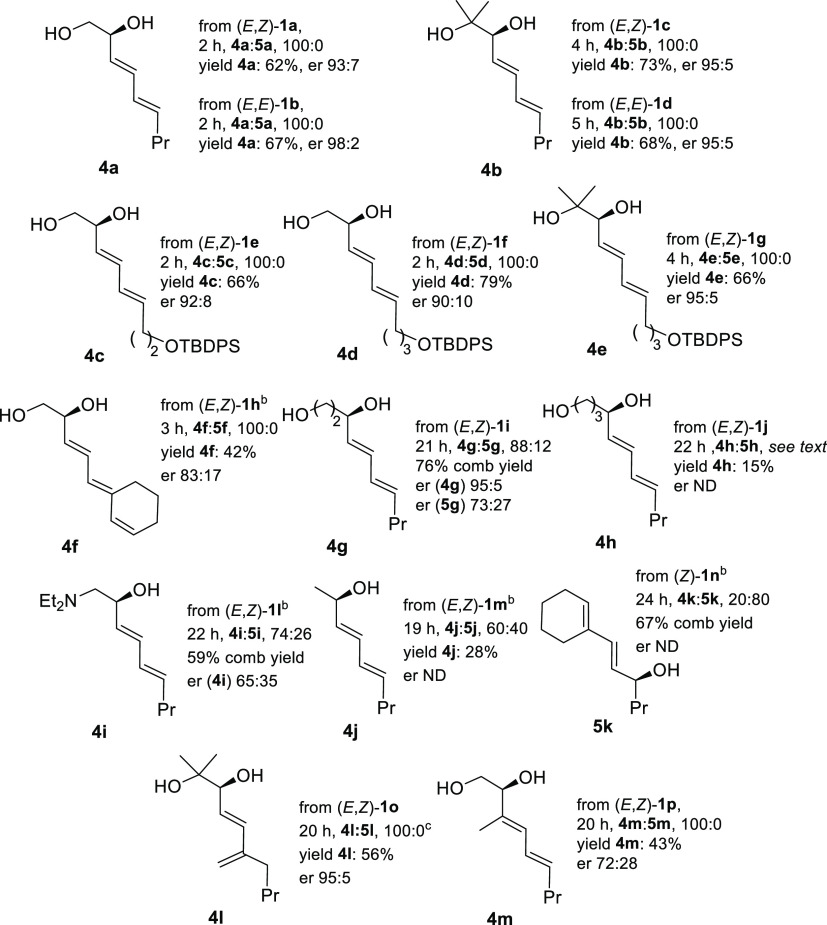
Scope of the Base-Promoted Sulfoxide-Sulfenate Rearrangement

a Er and absolute configuration of **4** were determined by ^1^H NMR as (*S*)-MPA esters **6** and **7**.

b 6 equiv of KH instead of NaH, which failed in promoting the reaction.

c A 16% of sulfinyl triene **4la** (not shown) was also isolated.

Additionally, we examined the effect of replacing the hydroxymethyl group (in R^2^) by a diethylaminomethyl and a methyl group, detecting a decay in regioselectivity, enantioselectivity, and reactivity (22 h for **1l** and 19 h for **1m**, using KH). Furthermore, **1n**, with a *c*-hexenyl fragment embedded in the diene structure underwent an inversion in regioselectivity (**4k**:**5k**, 20:80) upon treatment with KH. Also, 2-sulfinyl dienes with additional substitution **1o** (R^3^ = Me) and **1p** (R^4^ = Me) were submitted to the base-promoted sulfoxide-sulfenate rearrangement. Lower reactivity (20 h) but complete regioselectivity was found for both substrates [**4l** (er 95:5) and **4m** (er 72:28)] with the remarkable formal chemoselective deprotonation at the methyl group (R^3^) for **1o**. Finally, we explored an increase of substitution at the allylic position by attaching a *c*-hexyl group directly to the sulfinyl diene (**1q**) and we observed a decay in reactivity, namely, complete recovery of starting material for NaH and partial recovery (50%) along with a complex mixture of unidentified minor compounds for KH after 24 h at room temperature.

**Figure 1:**
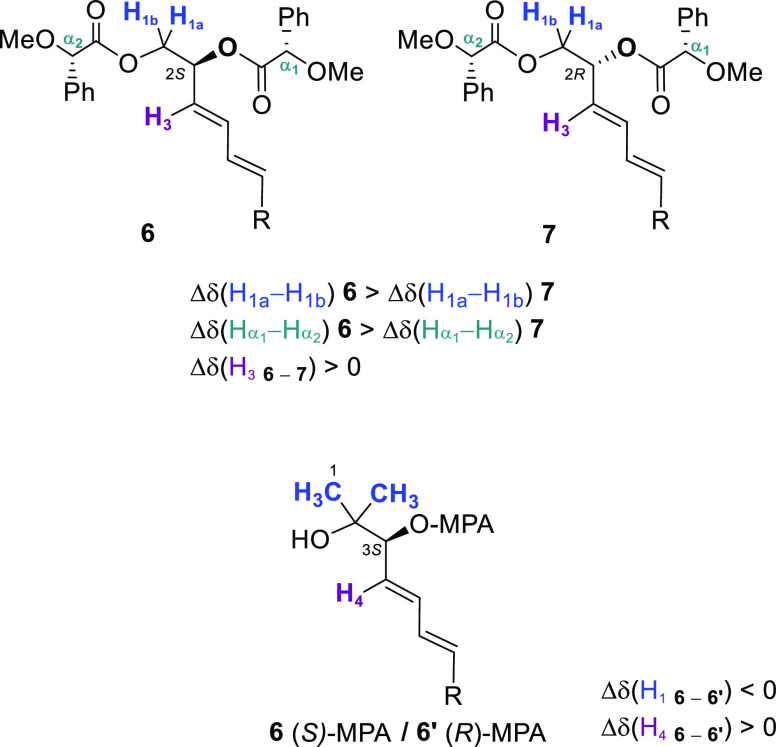
Stereochemical assignment of MPA esters.

Diols **4** were transformed into methoxyphenyl acetates (MPA) for full stereochemical assignment of pure or enriched samples (Figure 1).^12^ As previously documented,^7,9^ diesters of 1,2-diols [(*S*)-MPA] displayed larger differences in H_1_ chemical shifts for **6** (C_2_ (*S*), Δ*H*_1a–1b_ = 0.34–0.35 ppm) than for **7** (C_2_ (*R*), Δ*H*_1a–1b_ = 0.07–0.10 ppm), in H_α_ [Δ*H*_α1−α2_ (**6**) > Δ*H*_α1−α2_ (**7**)] and also H_3_ in **6** appears downfield relative to **7**. Similarly, differences in chemical shifts for H_1_ and H_4_ in monoesters of **4b**, **4e**, **4i**, and **4l** [(*S*)-MPA, **6** and [(*R*)-MPA, **6′**] were consistent with the stereochemistry proposed.

**Scheme 2:**
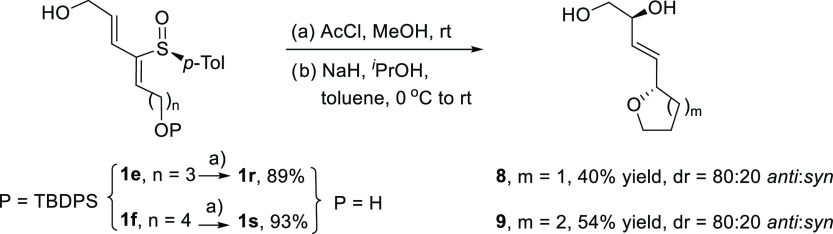
Intramolecular Conjugate Addition of Distal Alkoxides

Next, we examined the base-induced sulfoxide-sulfenate rearrangement on 2-sulfinyl dienes **1r** and **1s**, with an additional hydroxyl group in R^1^, which can compete by intramolecular Michael addition, smoothly prepared by acidic desilylation of **1e** and **1f** (Scheme 2). Upon treatment with NaH/^*i*^PrOH in toluene, both substrates underwent intramolecular conjugate addition of the distal alkoxide onto the dienyl sulfoxide followed by [2,3]-sigmatropic rearrangement to render tetrahydrofuran **8** and tetrahydropyran **9** with moderate yields and an 80:20 *anti*:*syn* diastereostereoselectivity (measured by integration of the ^1^H NMR as (*S*)-MPA esters **10** and **11**, not shown). The enantiomeric ratios found (80:20 for the major *anti* diastereomer), lower than in previously studied intermolecular reactions, could be tentatively attributed to partial racemization of the sulfinyl group upon deprotection under acidic conditions with AcCl in MeOH.

**Scheme 3:**
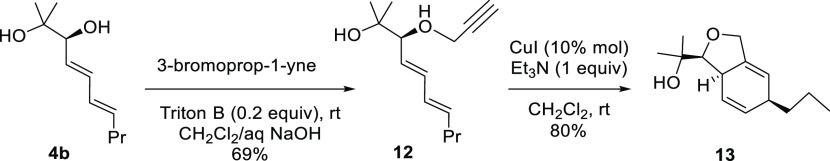
Intramolecular [4+2] Cycloaddition from Diene Diol **4b**

Finally, to illustrate the synthetic versatility of dienyl diols **4** accessible by our sequence, we addressed the chemoselective propargylation of the secondary hydroxyl group in **4b** to afford **12**, which underwent an intramolecular [4+2] cycloaddition upon treatment with a catalytic amount of CuI and Et_3_N,^13^ affording tetrahydroisobenzofuran **13** with complete diastereoselectivity and in 80% yield (Scheme 3).

### Theoretical Calculation at the Density Functional Theory (DFT) Level

**Figure 2:**
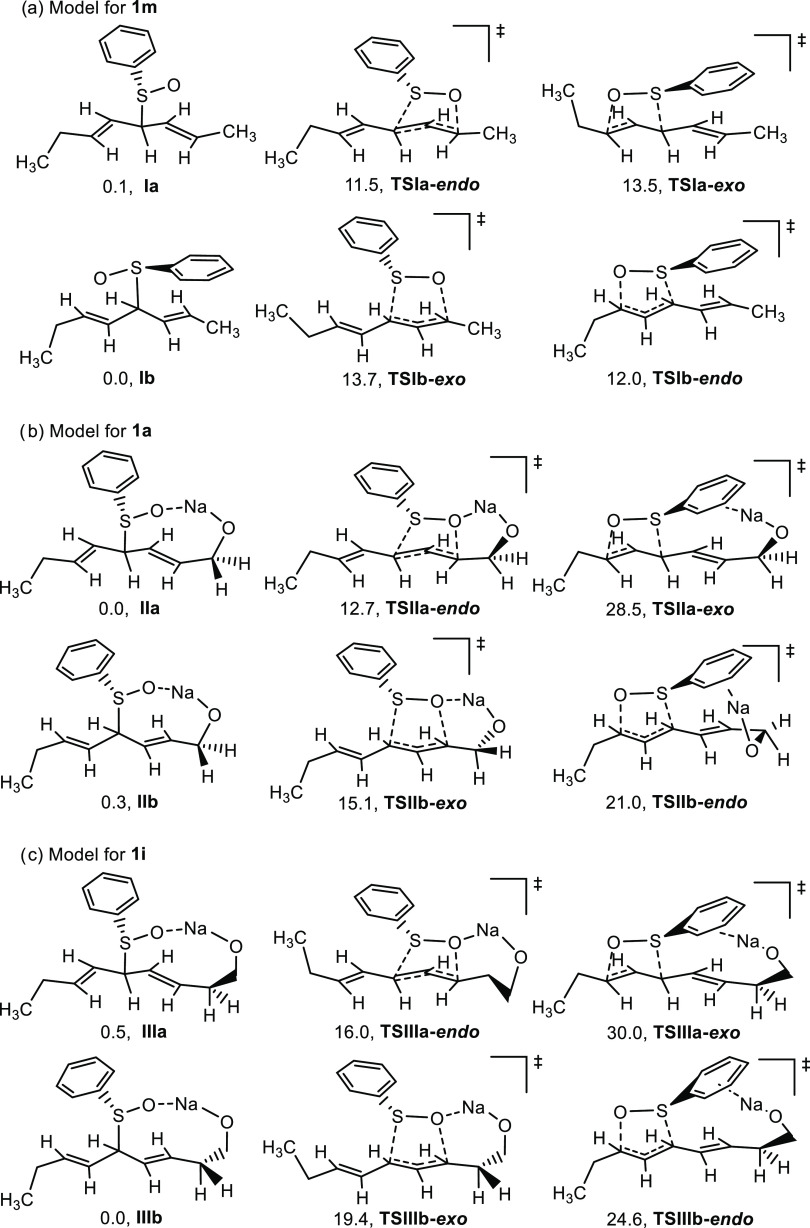
Spatial representation of the most stable conformation and transition states derived from (a) model **I**, (b) model **II**, and (c) model **III**, (M062X_SMD(toluene)_/6-311++G(d,p)//M062X_SMD(toluene)_/6-31G (d)). Relative *G* values at 298 K (kcal·mol^–1^).

To gain further understanding of the origin and influence of substituents on the regio- and stereoselectivities observed, the possible intermediates and transition states derived from selected model structures were studied by DFT calculations.^14^Figure 2a shows the structure of model **I**, a bis-allyl sulfoxide derived from the deprotonation/protonation of a linear alkyl substituted diene that could be used as a model for **1m**. Considering the excess of base required for the reaction and the high conformational freedom expected for the intermediate anions,^15^ the protonation and subsequent transition states for the sigmatropic rearrangement were analyzed to occur through both faces of the diene leading to equally stable bis-allyl sulfoxides (structures a and b). Under the reaction conditions, the source of protons is uncertain. Quenching the reaction with MeOH-*d*_4_ or D_2_O does not lead to any deuteration in the final compounds; therefore, we hypothesized that the sigmatropic rearrangement takes place on allylic sulfoxides rather than on the anionic species and protonation could derive from other molecules of diene since no deuteration of **4a** was observed when the reaction of **1a** was performed in toluene-*d*_8_ either in the presence or absence of ^*i*^PrOH (Table 2, entries 2 and 11).

Thus, the regioselectivity observed seems to be determined by the relative energy between **TSIa-*****endo*** and **TSIb-*****end*****o**(3a) that predicts a ratio of 70:30 in relatively good agreement with experimental result. The higher stability of **TSIa-*****endo*** against **TSIb-*****endo*** could be related to the low steric hindrance exerted by the methyl group close to the bond being formed. This trend could also explain the reverse selectivity observed in the case of diene **1n** (**4k**:**5k**, 20:80).

Model **II**, with a hydroxyl group, could be used as a model for **1a** (Figure 2b). Under the reaction conditions, the most important intramolecular stabilizing interactions are the coordination of the sodium alkoxide to the sulfoxide oxygen or to the aromatic ring depending on the conformation around the C–S bond. The difference in energy between **TSIIa-*****endo*** and **TSIIb-*****exo*** (2.4 kcal·mol^–1^) predicts a ratio of 98:2 in good agreement with experimental stereoselectivity observed. On the other hand, the relative energy between **TSIIa-*****endo*** and **TSIIb-*****endo*** would explain the complete regioselectivity observed in the reaction of **1a**.

The introduction of an additional methylene group between the olefinic carbon and the hydroxyl group yields **III** (model for **1i**, Figure 2c). Although the most important intramolecular stabilizing interactions are the same as in model **II**, the increase in cycle size results in an increase in possible conformations, of which the most stable for each approach are shown in the figure. According to the data, although the stereoselectivity of the main compound could be justified as in the previous model, the high instability of **TSIIIb-*****endo*** does not account for the formation of the minor regioisomer experimentally observed, suggesting a more complex model could be involved in this case. Additionally, according to these calculations, the presence of a hydroxyl group in the molecule does not translate into a decrease of the activation barrier for the sigmatropic rearrangement (compare relative energies for the most stable *endo* TSs in Figure 2a–c). Therefore, the experimental observation of an increase in the reaction speed when a hydroxyl group is present could be related to the formation of the corresponding alkoxide that could work as a directing group favoring the approach of the base to form the carbanion.

In summary, we have designed a series of 2-sulfinyl dienes having different substitutions for the study of the base-induced [2,3]-sigmatropic rearrangement. After allylic deprotonation/protonation, the bis-allylic sulfoxide intermediate undergoes sulfoxide-sulfenate rearrangement to afford dienyl diols **4**. The presence of an allylic alcohol is determinant for the good reactivity of 2-sulfinyl dienes and to achieve complete regioselectivity and high enantioselectivity (90:10–95:5) with the sulfoxide group as the only element of stereocontrol.

## Experimental Section

### General Section

Reagents and solvents were handled using standard syringe techniques. All reactions were carried out under an argon atmosphere. Anhydrous solvents (toluene, CH_3_CN, CH_2_Cl_2_, Et_2_O, THF, and DMF) were purified by filtration on a solvent purification system. So-collected toluene was stored over CaH_2_. Oil-free NaH and KH were obtained from the NaH or KH/mineral oil mixture that was carefully washed with hexane and dried prior to use. Crude products were purified by flash chromatography on 230–400 mesh silica gel with distilled solvents. Analytical thin-layer chromatography (TLC) was carried out on silica gel plates with detection by UV light, iodine, ninhydrin solution in ethanol, and 10% phosphomolybdic acid solution in ethanol. All reagents were commercial products. Through this section, the volume of solvents is reported in mL/mmol of starting material. ^1^H and ^13^C NMR spectra were recorded at 300, 400, or 500 MHz (^1^H) using CDCl_3_ as solvent unless otherwise indicated and with the residual solvent signal as internal reference unless otherwise noted. The following abbreviations are used to describe peak patterns when appropriate: s (singlet), d (doublet), t (triplet), q (quartet), m (multiplet), br (broad), ap (apparent). Optical rotations were measured at 20 °C using a sodium lamp and in CHCl_3_ solution unless otherwise stated. High-resolution mass spectra (HRMS) were recorded using Accurate Mass quadrupole time-of-flight (Q-TOF), liquid chromatography/mass spectrometer (LC/MS), Agilent Technologies 6520 spectrometer.

### Synthesis of Starting Materials

#### Synthesis of (*E*)-Vinyl Stannanes

Unless otherwise noted, vinyl stannanes were prepared following this modified procedure: A dry two-neck round-bottom flask fitted with a reflux condenser was charged with commercially available hydroxy alkyne (1 equiv) and AIBN (0.01 equiv) under argon and anhydrous toluene was added (1 mL × mmol). The mixture was heated at 115 °C using an oil bath, and then a solution of Bu_3_SnH (1.5 equiv) and AIBN (0.04 equiv) in toluene (1 mL × mmol) was added dropwise, maintaining a moderate reflux until disappearance of starting material (TLC, 4 h). The solvent was removed, and the crude product was purified by column chromatography on silica gel 5–20% (Et_2_O-hexane) to afford (*E*)-vinyl stannanes as colorless oils (15–20% of regio- and stereoisomers were identified in the crude mixtures).

##### (*E*)-2-Methyl-4-(tributylstannyl)but-3-en-2-ol

2.95 g, 7.86 mmol, yield: 66%. Spectroscopic data are in agreement with those previously reported.^16^

##### (*E*)-4-(Tributylstannyl)but-3-en-1-ol

4.07 g, 11.27 mmol, yield: 80%. Spectroscopic data are in agreement with those previously reported.^17^

##### (*E*)-5-(Tributylstannyl)pent-4-en-1-ol

3.26 g, 8.68 mmol, yield: 73%. Spectroscopic data are in agreement with those previously reported.^17^

#### Synthesis of Iodo Vinyl Sulfoxides

Iodo vinyl sulfoxides were originally prepared from (1*R*,2*S*,5*R*)-(−)-menthyl (*S*)-*p*-toluenesulfinate, checking the optical rotation for each batch prior to use.^11,18^

##### General Procedure for the Synthesis of Sulfinyl Vinyl Stannanes

To a solution of alkynyl sulfoxide (1 equiv) in anhydrous toluene (6 mL/mmol sulfoxide) at rt under argon, Pd(Ph_3_P)_4_ (0.02 equiv) was added. The mixture was cooled to −78 °C, and a solution of Bu_3_SnH (1.1 equiv) in toluene (1 mL/mmol sulfoxide) was added. The mixture was stirred and warmed up to room temperature until disappearance of starting material (TLC, 16 h). The solvent was removed, and the crude product was purified by column chromatography.

##### General Procedure for the Synthesis of Iodo Vinyl Sulfoxides

To a solution of I_2_ (1.2 equiv) in CH_2_Cl_2_ (6 mL/mmol sulfoxide) at rt under argon was added a solution of sulfinyl vinyl stannanes (1.0 equiv) in CH_2_Cl_2_ (5 mL/mmol sulfoxide). The mixture was stirred at rt until disappearance of starting material (TLC, 40 min), and then it was quenched with a Na_2_S_2_O_4_ solution (2 mL/mmol, 1 M) and diluted with EtOAc. The layers were separated, and the aqueous layer was extracted with EtOAc (2 × 4 mL/mmol sulfoxide). The combined organic layers were washed with brine, dried over MgSO_4_, filtered, and concentrated under reduced pressure, and the crude was purified by column chromatography on silica gel.

#### Synthesis of (*S*,*E*)-*tert*-Butyl((6-iodo-6-(*p*-tolylsulfinyl)hex-5-en-1-yl)oxy)diphenylsilane

##### (*S*)-*tert*-Butyldiphenyl((6-(*p*-tolylsulfinyl)hex-5-yn-1-yl)oxy)silane

A dry two-neck round-bottom flask fitted with a reflux condenser was charged with dry Mg turnings (454 mg, 18.66 mmol, 1.5 equiv) under argon and anhydrous Et_2_O was added (0.1 mL/mmol Mg) and 5–10 drops of EtBr. The mixture was heated slowly using a heat gun until the formation of the Grignard reagent was initiated. Then, a solution of EtBr (1.49 mL, 19.91 mmol, 1.6 equiv) in Et_2_O (1.0 mL/mmol Mg) was added dropwise, maintaining a moderate reflux using a heat gun if it is necessary, and after the addition was complete, the mixture was heated to reflux until complete disappearance of Mg was observed. To the solution of EtMgBr at rt, *tert*-butyl(hex-5-yn-1-yloxy)diphenylsilane^19^ (6.7 g, 19.91 mmol, 1.6 equiv) was added, and the mixture was heated to reflux using an oil bath for 1.5 h. The alkynyl Grignard was added (via syringe) to a cold solution (−40 °C) of (−)-menthyl (*S*)-*p*-toluenesulfinate (3.67 g, 12.44 mmol, 1 equiv) in anhydrous toluene (6 mL/mmol sulfoxide), and the reaction was stirred at low temperature until disappearance of starting material was observed (TLC, 3 h). The mixture was quenched by the addition of a saturated NH_4_Cl solution (4 mL/mmol sulfoxide) and H_2_O (4 mL/mmol sulfoxide) and diluted with EtOAc. The layers were separated, the aqueous layer was extracted with EtOAc (2 × 4 mL/mmol), and the combined organic layers were washed with brine, dried over MgSO_4_, filtered, and concentrated under reduced pressure. The crude product was purified by column chromatography on silica gel (5–30% EtOAc-hexane) to afford the title compound as a colorless oil (4.48 g, 9.44 mmol, yield: 76%). Data: *R*_*f*_ 0.31 (20% EtOAc-hexane). [α]_D_^20^ +41.8 (*c* = 0.63). ^1^H NMR (400 MHz) δ 7.70–7.62 (m, 6H), 7.45–7.30 (m, 8H), 3.65 (t, 2H, *J* = 5.8 Hz), 2.44–2.39 (m, 5H), 1.72–1.59 (m, 4H), 1.04 (s, 9H). ^13^C{^1^H} NMR (100 MHz) δ 142.4, 141.4, 135.7 (4C), 133.9 (2C), 130.3 (2C), 129.8 (2C), 127.8 (4C), 125.3 (2C), 105.8, 78.6, 63.2, 31.6, 27.0 (3C), 24.3, 21.6, 19.7, 19.3. HRMS (ESI) *m*/*z* calcd for C_29_H_34_NaO_2_SSi [M + Na]^+^ 497.1941; found 497.1941.

##### (*S*,*E*)-*tert*-Butyldiphenyl((6-(*p*-tolylsulfinyl)-6-(tributylstannyl)hex-5-en-1-yl)oxy)silane

From (*S*)-*tert*-butyldiphenyl((6-(*p*-tolylsulfinyl)hex-5-yn-1-yl)oxy)silane (3.48 g, 7.33 mmol), Pd(Ph_3_P)_4_ (169 mg, 0.147 mmol), and Bu_3_SnH (2.17 mL, 8.06 mmol) in toluene following the general procedure was obtained the title compound as a colorless oil (3.85 g, 5.03 mmol, yield: 69%) after column chromatography on silica gel (2–10% EtOAc-hexane). Data: *R*_*f*_ 0.52 (20% EtOAc-hexane). [α]_D_^20^ −32.0 (*c* = 0.60). ^1^H NMR (400 MHz) δ 7.69–7.64 (m, 4H), 7.45–7.34 (m, 8H), 7.26–7.21 (m, 2H), 6.16 (dd, 1H, *J* = 8.6, 5.7 Hz), 3.69 (t, 2H, *J* = 5.7 Hz), 2.75–2.61 (m, 1H), 2.44–2.31 (m, 4H), 1.68–1.54 (m, 4H), 1.46–1.15 (m, 12H), 1.06 (s, 9H), 0.83 (m, 15H). ^13^C{^1^H} NMR (100 MHz) δ 156.5, 148.9, 142.7, 140.1, 135.7 (4C), 134.04, 134.02, 129.7 (2C), 129.6 (2C), 127.7 (4C), 124.5 (2C), 63.6, 32.8, 32.1, 28.9 (3C), 27.3 (3C), 27.0 (3C), 25.8, 21.4, 19.3, 13.8 (3C), 11.5 (3C). HRMS (ESI) *m*/*z* calcd for C_41_H_63_O_2_SSiSn [M + H]^+^ 767.3336; found 767.3305.

##### (*S*,*E*)-*tert*-Butyl((6-iodo-6-(*p*-tolylsulfinyl)hex-5-en-1-yl)oxy)diphenylsilane

From (*S,E*)-*tert*-butyldiphenyl((6-(*p*-tolylsulfinyl)-6-(tributylstannyl)hex-5-en-1-yl)oxy)silane (3.85 g, 5.02 mmol) and I_2_ (1.53 g, 6.03 mmol) in CH_2_Cl_2_ following the general procedure was obtained after column chromatography on silica gel (5–20% EtOAc-hexane) the title compound as a colorless oil (2.83 g, 4.70 mmol, yield: 94%). Data: *R*_*f*_ 0.36 (20% EtOAc-hexane). [α]_D_^20^ −49.8 (*c* = 0.60). ^1^H NMR (400 MHz) δ 7.68–7.65 (m, 4H), 7.46–7.37 (m, 8H), 7.29 (d, 2H, *J* = 8.2 Hz), 6.83 (dd, 1H, *J* = 8.6, 7.2 Hz), 3.71 (m, 2H), 2.83–2.72 (m, 1H), 2.63–2.54 (m, 1H), 2.40 (s, 3H), 1.65 (m, 4H), 1.07 (s, 9H). ^13^C{^1^H} NMR (100 MHz) δ 151.9, 141.8, 140.1, 135.7 (4C), 133.9 (2C), 130.0 (2C), 129.8 (2C), 127.8 (4C), 124.5 (2C), 114.7, 63.3, 33.5, 32.0, 27.0 (3C), 25.5, 21.7, 19.4. HRMS (ESI) *m*/*z* calcd for C_29_H_35_INaO_2_SSi [M + Na]^+^ 625.1064; found 625.1064.

#### Synthesis of (*S*,*E*)-1-((2-Cyclohexyl-1-iodovinyl)sulfinyl)-4-methylbenzene

##### (*S*,*E*)-Tributyl(2-cyclohexyl-1-(*p*-tolylsulfinyl)vinyl)stannane

From (*S*)-1-((cyclohexylethynyl)sulfinyl)-4-methylbenzene^20^ (645 mg, 2.62 mmol), Pd(Ph_3_P)_4_ (60 mg, 0.052 mmol), and Bu_3_SnH (0.77 mL, 2.88 mmol) in toluene following the general procedure was obtained the title compound as a colorless oil (994 mg, 1.85 mmol, yield: 70%) after column chromatography on silica gel (5–40% Et_2_O-hexane). Data: *R*_*f*_ 0.32 (20% EtOAc-hexane). [α]_D_^20^ −66.5 (*c* = 1.55). ^1^H NMR (400 MHz) δ 7.38 (d, 2H, *J* = 7.6 Hz), 7.29 (d, 2H, *J* = 7.6 Hz), 6.00 (d, 2H, *J* = 10.2 Hz), 2.91–2.82 (m, 1H), 2.38 (s, 3H), 1.87–1.62 (m, 6H), 1.43–1.13 (m, 22H), 0.89–0.74 (m, 9H). ^13^C{^1^H} NMR (100 MHz) δ 155.0, 152.8, 142.7, 140.1, 129.7 (2C), 124.7 (2C), 41.6, 33.0, 32.7, 28.9 (3C), 27.3 (3C), 25.9, 25.5, 25.4, 21.4, 13.8 (3C), 11.5 (3C). HRMS (ESI) *m*/*z* calcd for C_27_H_46_OSSn [M + H]^+^ 539.2368; found 539.2366.

##### (*S*,*E*)-1-((2-Cyclohexyl-1-iodovinyl)sulfinyl)-4-methylbenzene

From I_2_ (545 mg, 2.15 mmol) and (*S*,*E*)-tributyl(2-cyclohexyl-1-(*p*-tolylsulfinyl)vinyl)stannane (962 mg, 1.79 mmol) in CH_2_Cl_2_ following the general procedure was obtained the title compound after chromatography on silica gel (5–20% EtOAc-hexane) as a white solid (549 mg, 1.37 mmol, yield: 77%). Data: *R*_*f*_ 0.30 (10% EtOAc-hexane). [α]_D_^20^ −116.7 (*c* = 1.17). Mp: 84–86 °C. ^1^H NMR (400 MHz, acetone-*d*_6_) δ 7.48 (d, 2H, *J* = 8.5 Hz), 7.39 (d, 2H, *J* = 8.4 Hz), 6.83 (d, 1H, *J* = 10.2 Hz), 3.29–3.17 (m, 1H), 2.39 (s, 3H), 1.88–1.67 (m, 6H), 1.51–1.17 (m, 4H). ^13^C{^1^H} NMR (100 MHz, acetone-*d*_6_) δ 157.6, 142.4, 142.0, 130.6 (2C), 125.4 (2C), 114.6, 43.5, 33.1, 32.8, 26.2, 25.91, 25.87, 21.4. HRMS (ESI) *m*/*z* calcd for C_15_H_19_IOS [M + H]^+^ 375.0274; found 375.0258.

#### General Procedure for the Synthesis of Sulfinyl Dienes, **1**

Dienes (*E*,*Z*)-**1a**,^18^ (*E*,*E*)-**1b**,^21^ (*E*,*Z*)-**1h**,^18^ and (*E*,*Z*)-**1k**^9^ were already reported.

##### Method A

A degassed solution of iodo vinyl sulfoxide (1.0 equiv), vinylstannane (1.3 equiv), BHT (1.0 equiv), Ph_3_As (0.1 equiv), and Pd_2_(dba)_3_·CHCl_3_ (0.05 equiv) in THF 10 mL/mmol of iodo vinyl sulfoxide) was stirred under Ar and at rt until disappearance of the starting material (TLC). The solvent was removed under vacuum, and the crude residue was purified by chromatography on silica gel.

##### Method B

A degassed solution of iodo vinyl sulfoxide (1.0 equiv), vinylstannane (1.3 equiv), and Pd(CH_3_CN)_2_Cl_2_ (0.1 equiv) in DMF (2.5 mL/mmol of iodo vinyl sulfoxide) was stirred under Ar and at rt until disappearance of the starting material (TLC). Then, it was filtered through celite and the solution was concentrated in vacuo. The crude material was purified by column chromatography on silica gel.

##### Method C

A solution of iodo vinyl sulfoxide (1.0 equiv), (*E*)-prop-1-en-1-ylboronic acid or 1-cyclohexen-1-yl-boronic acid (2.0 equiv), CsF (4.0 equiv), and Pd(PPh_3_)_4_ (0.1 equiv) in THF (15 mL/mmol of iodo vinyl sulfoxide) was stirred under Ar and at rt until disappearance of the starting material (TLC). Then, it was filtered through celite and the solution was concentrated in vacuo. The crude material was purified by column chromatography on silica gel.

##### Method D

To a 0 °C solution of 1.0 equiv of 2-sulfinyl diene **1e** or **1f** in MeOH (6 mL/mmol), acetyl chloride (0.3 equiv) was added. The mixture was stirred under Ar and warmed up to room temperature until disappearance of the starting material (TLC). CHCl_3_ and a saturated solution of NaHCO_3_ (10 mL/mmol sulfinyl diene) were added to the reaction mixture, and then it was stirred for 5 min. The aqueous layer was extracted with CHCl_3_ (4 × 5 mL), and the combined organic layers were washed with saturated NaCl solution, dried over MgSO_4_, filtered, and concentrated under vacuum to obtain a crude product that was purified by column chromatography on silica gel using EtOAc as eluent.

##### (3*E*,5*Z*)-2-Methyl-5-((*R*)-*p*-tolylsulfinyl)deca-3,5-dien-2-ol, (*E*,*Z*)-**1c**

From (*S*,*E*)-1-((1-iodohex-1-en-1-yl)sulfinyl)-4-methylbenzene^18^ (630 mg, 1.810 mmol), (*E*)-2-methyl-4-(tributylstannyl)but-3-en-2-ol (883 mg, 2.350 mmol), and Pd(CH_3_CN)_2_Cl_2_ (47 mg, 0.181 mmol) in DMF (4.5 mL) according to method B (20 h), (*E,Z*)-**1c** was obtained. Purification by column chromatography (10–40% EtOAc-hexane) afforded (*E,Z*)-**1c** (536 mg, 1.485 mmol, yield: 82%) as a brown oil. Data for (*E*,*Z*)-**1c**: *R*_*f*_ 0.37 (10% EtOAc-hexane). [α]_D_^20^ +72.3 (*c* = 1.0). ^1^H NMR (400 MHz), COSY δ 7.41 (dm, 2H, *J* = 8.3 Hz, ArH), 7.27 (dm, 2H, *J* = 8.2 Hz, ArH), 6.19 (t, 1H, *J* = 7.8 Hz, H-6), 6.13–6.03 (m, 2H, H-3, H-4), 2.71 (m, 1H, H-7), 2.52 (m, 1H, H-7), 2.39 (s, 3H, CH_3_*p*-Tol), 1.71 (brs, 1H, OH), 1.54–1.39 (m, 4H), 1.21 (s, 3H, CH_3_), 1.19 (s, 3H, CH_3_), 0.95 (t, 3H, *J* = 7.2 Hz, CH_3_). ^13^C{^1^H} NMR (100 MHz), HSQC, HMBC δ 142.6, 142.4, 140.7, 140.0, 138.4, 129.8 (2C), 124.4 (2C), 118.0, 71.0, 31.7, 29.7, 29.5, 28.7, 22.5, 21.5, 14.0. IR (film): 3401, 2962, 2928, 2872, 1631, 1597, 1492, 1464, 1376, 1232, 1157, 1082, 1038, 966, 808, 623, 573, 503 cm^–1^. HRMS (ESI) *m*/*z* calcd for C_18_H_26_NaO_2_S [M + Na]^+^ 329.1546; found 329.1543.

##### (3*E*,5*E*)-2-Methyl-5-((*R*)-*p*-tolylsulfinyl)deca-3,5-dien-2-ol, (*E*,*E*)-**1d**

From (*S*,*Z*)-1-((1-iodohex-1-en-1-yl)sulfinyl)-4-methylbenzene^11^ (623 mg, 1.789 mmol), (*E*)-2-methyl-4-(tributylstannyl)but-3-en-2-ol (872 mg, 2.326 mmol), and Pd(CH_3_CN)_2_Cl_2_ (46 mg, 0.179 mmol) in DMF (4.5 mL) according to method B (24 h), (*E,E*)-**1d** was obtained. Purification by column chromatography (20–60% EtOAc-hexane) afforded (*E,E*)-**1d** (384 mg, 1.252 mmol, yield: 70%) as a brown oil. Data for (*E*,*E*)-**1d**: *R*_*f*_ 0.40 (20% EtOAc-hexane). [α]_D_^20^ +81.1 (*c* = 0.84). ^1^H NMR (400 MHz) δ 7.45 (d, 2H, *J* = 8.0 Hz), 7.21 (d, 2H, *J* = 8.0 Hz), 6.46 (t, 1H, *J* = 7.6 Hz), 6.12 (d, 1H, *J* = 16.2 Hz), 6.00 (d, 1H, *J* = 16.2 Hz), 2.35 (s, 3H), 2.33–2.26 (m, 2H), 1.92 (brs, 1H), 1.51–1.42 (m, 2H), 1.39–1.30 (m, 2H), 1.20 (s, 3H), 1.19 (s, 3H), 0.90 (t, 3H, *J* = 7.3 Hz). ^13^C{^1^H} NMR (100 MHz) δ 143.7, 141.4, 140.8, 140.7, 135.0, 129.7 (2C), 125.6 (2C), 115.9, 71.0, 31.2, 29.8, 29.7, 28.1, 22.4, 21.5, 14.0. HRMS (ESI) *m*/*z* calcd for C_18_H_26_NaO_2_S [M + Na]^+^ 329.1546; found 329.1557.

##### (2*E*,4*Z*)-8-((*tert*-Butyldiphenylsilyl)oxy)-4-((*R*)-*p*-tolylsulfinyl)octa-2,4-dien-1-ol, (*E*,*Z*)-**1e**

From (*S*,*E*)-*tert*-butyl((5-iodo-5-(*p*-tolylsulfinyl)pent-4-en-1-yl)oxy)diphenylsilane^18^ (182 mg, 0.309 mmol), (*E*)-3-(tributylstannyl)prop-2-en-1-ol (140 mg, 0.402 mmol),^22^ BHT (68 mg, 0.309 mmol), Ph_3_As (10 mg, 0.031 mmol), and Pd_2_(dba)_3_·CHCl_3_ (16 mg, 0.015 mmol) in THF (10 mL) according to method A (20 h), (*E,Z*)-**1e** was obtained. Purification by column chromatography (40–50% Et_2_O-CH_2_Cl_2_) afforded (*E,Z*)-**1e** (132 mg, 0.254 mmol, yield: 82%) as a brown oil. Data for (*E,Z*)-**1e**: *R*_*f*_ 0.38 (80% Et_2_O-CH_2_Cl_2_). [α]_D_^20^ −74.2 (*c* = 1.02). ^1^H NMR (400 MHz) δ 7.67 (dm, 4H, *J* = 7.8 Hz), 7.46–7.35 (m, 8H), 7.25 (dm, 2H, *J* = 7.5 Hz), 6.23 (t, 1H, *J* = 7.9 Hz), 6.17–6.04 (m, 2H), 4.09 (m, 2H), 3.75 (m, 2H), 2.84–2.63 (m, 2H), 2.39 (s, 3H), 1.79–1.70 (m, 3H), 1.06 (s, 9H). ^13^C{^1^H} NMR (100 MHz) δ 142.7, 140.8, 139.8, 138.0, 135.70 (2C), 135.68 (2C), 133.9, 133.8 (2C), 130.0 (2C), 129.83, 129.82, 127.8 (4C), 124.4 (2C), 121.7, 63.3, 63.2, 32.4, 27.0 (3C), 25.8, 21.5, 19.4. HRMS (ESI) *m*/*z* calcd for C_31_H_39_O_3_SSi [M + H]^+^ 519.2384; found 519.2375.

##### (2*E*,4*Z*)-9-((*tert*-Butyldiphenylsilyl)oxy)-4-((*R*)-*p*-tolylsulfinyl)nona-2,4-dien-1-ol, (*E*,*Z*)-**1f**

From (*S*,*E*)-*tert*-butyl((6-iodo-6-(*p*-tolylsulfinyl)hex-5-en-1-yl)oxy)diphenylsilane (378 mg, 0.629 mmol), (*E*)-3-(tributylstannyl)prop-2-en-1-ol^21^ (290 mg, 0.834 mmol), BHT (142 mg, 0.642 mmol), Ph_3_As (20 mg, 0.064 mmol), and Pd_2_(dba)_3_·CHCl_3_ (17 mg, 0.016 mmol) in THF (7 mL) according to method A (20 h), (*E,Z*)-**1f** was obtained. Purification by column chromatography (40–50% EtOAc-hexane) afforded (*E,Z*)-**1f** (285 mg, 0.530 mmol, yield: 85%) as a brown oil. Data for (*E,Z*)-**1f**: *R*_*f*_ 0.30 (40% EtOAc-hexane). [α]_D_^20^ −75.1 (*c* = 0.76). ^1^H NMR (400 MHz) δ 7.67 (dm, 4H, *J* = 7.7 Hz), 7.45–7.35 (m, 8H), 7.26 (d, 2H, *J* = 8.2 Hz), 6.23 (t, 1H, *J* = 7.8 Hz), 6.19–6.05 (m, 2H), 4.09 (brs, 2H), 3.71 (m, 2H), 2.74–2.64 (m, 1H), 2.58–2.49 (m, 1H), 2.38 (s, 3H), 2.00 (brs, 1H), 1.64 (m, 4H), 1.06 (s, 9H). ^13^C{^1^H} NMR (100 MHz) δ 142.5, 140.8, 139.7, 138.4, 135.7 (4C), 134.0, 133.9 (2C), 130.0 (2C), 129.7 (2C), 127.8 (4C), 124.3 (2C), 121.6, 63.5, 63.2, 32.2, 28.8, 27.0 (3C), 26.0, 21.5, 19.3. HRMS (ESI) *m*/*z* calcd for C_32_H_41_O_3_SSi [M + H]^+^ 533.2540; found 533.2528.

##### (3*E*,5*Z*)-10-((*tert*-Butyldiphenylsilyl)oxy)-2-methyl-5-((*R*)-*p*-tolylsulfinyl)deca-3,5-dien-2-ol, (*E*,*Z*)-**1g**

From (*S*,*E*)-*tert*-butyl((6-iodo-6-(*p*-tolylsulfinyl)hex-5-en-1-yl)oxy)diphenylsilane (360 mg, 0.597 mmol), (*E*)-2-methyl-4-(tributylstannyl)but-3-en-2-ol (291 mg, 0.776 mmol), and Pd(CH_3_CN)_2_Cl_2_ (16 mg, 0.060 mmol) in DMF (1.8 mL) according to method B (18 h), (*E,Z*)-**1g** was obtained. Purification by column chromatography (20–40% EtOAc-hexane) afforded (*E,Z*)-**1g** (303 mg, 0.540 mmol, yield: 90%) as a brown oil. Data for (*E,Z*)-**1g**: *R*_*f*_ 0.47(60% EtOAc-hexane). [α]_D_^20^ +96.7 (*c* = 0.89). ^1^H NMR (400 MHz) δ 7.67 (dm, 4H, *J* = 7.5 Hz), 7.44–7.36 (m, 8H), 7.25 (d, 2H, *J* = 7.5 Hz), 6.17 (t, 1H, *J* = 7.7 Hz), 6.14–6.05 (m, 2H), 3.70 (m, 2H), 2.74–2.64 (m, 1H), 2.58–2.50 (m, 1H), 2.38 (s, 3H), 1.64 (m, 5H), 1.21 (s, 3H), 1.20 (s, 3H), 1.06 (s, 9H). ^13^C{^1^H} NMR (100 MHz) δ 142.8, 142.4, 140.7, 140.0, 138.1, 135.7 (4C), 134.0 (2C), 129.81 (2C), 129.75 (2C), 127.8 (4C), 124.4 (2C), 118.0, 71.1, 63.5, 32.2, 29.7, 29.6, 28.8, 27.0 (3C), 26.0, 21.5, 19.4. HRMS (ESI) *m*/*z* calcd for C_34_H_45_O_3_SSi [M + H]^+^ 561.2853; found 561.2858.

##### (3*E*,5*Z*)-5-((*R*)-*p*-Tolylsulfinyl)deca-3,5-dien-1-ol, (*E*,*Z*)-**1i**

From (*S*,*E*)-1-((1-iodohex-1-en-1-yl)sulfinyl)-4-methylbenzene^18^ (3.01 g, 8.66 mmol), (*E*)-4-(tributylstannyl)but-3-en-1-ol (3.90 g, 10.39 mmol), BHT (1.91 g, 8.66 mmol), Ph_3_As (520 mg, 1.73 mmol), and Pd_2_(dba)_3_·CHCl_3_ (448 mg, 0.43 mmol) in THF (87 mL) according to method A (18 h), (*E,Z*)-**1i** was obtained. Purification by column chromatography (30–80% EtOAc-hexane) afforded (*E,Z*)-**1i** (2.26 g, 7.73 mmol, yield: 89%) as a brown oil. Data for (*E,Z*)-**1i**: *R*_*f*_ 0.27 (80% EtOAc-hexane). [α]_D_^20^ −155.0 (*c* = 0.66). ^1^H NMR (300 MHz) δ 7.42 (d, 2H, *J* = 8.3 Hz), 7.28 (d, 2H, *J* = 8.3 Hz), 6.19 (t, 1H, *J* = 7.9 Hz), 5.95–5.89 (m, 2H), 3.58–3.46 (m, 2H), 2.80–2.64 (m, 1H), 2.58–2.45 (m, 1H), 2.39 (s, 3H), 2.29–2.21 (m, 2H), 1.59–1.36 (m, 5H), 0.95 (t, 3H, *J* = 7.1 Hz). ^13^C{^1^H} NMR (75 MHz) δ 142.5, 140.6, 139.5, 137.9, 132.5, 129.7 (2C), 124.1 (2C), 123.0, 61.2, 36.4, 31.5, 28.5, 22.3, 21.2, 13.8. HRMS (ESI) *m*/*z* calcd for C_17_H_24_NaO_2_S [M + Na]^+^ 315.1395; found 315.1389.

##### (4*E*,6*Z*)-6-((*R*)-*p*-Tolylsulfinyl)undeca-4,6-dien-1-ol, (*E*,*Z*)-**1j**

From (*S*,*Z*)-1-((1-iodohex-1-en-1-yl)sulfinyl)-4-methylbenzene^18^ (350 mg, 1.01 mmol), (*E*)-5-(tributylstannyl)pent-4-en-1-ol (490 mg, 1.31 mmol), BHT (222 mg, 1.01 mmol), Ph_3_As (31 mg, 0.101 mmol), and Pd_2_(dba)_3_·CHCl_3_ (52 mg, 0.05 mmol) in THF (15 mL) according to method A (24 h), (*E,Z*)-**1j** was obtained. Purification by column chromatography (20–80% EtOAc-hexane) afforded (*E,Z*)-**1j** (210 mg, 0.685 mmol, yield: 68%) as a brown oil. Data for (*E,Z*)-**1j**: *R*_*f*_ 0.36 (80% EtOAc-hexane). [α]_D_^20^ −159.3 (*c* = 1.00). ^1^H NMR (400 MHz) δ 7.40 (dm, 2H, *J* = 8.3 Hz), 7.27 (d, 2H, *J* = 8.2 Hz), 6.15 (t, 1H, *J* = 7.8 Hz), 5.99–5.82 (m, 2H), 3.47 (t, 2H, *J* = 6.4 Hz), 2.74–2.64 (m, 1H), 2.54–2.44 (m, 1H), 2.38 (s, 3H), 2.07 (ap q, 2H, *J* = 7.1 Hz), 1.85 (brs, 1H), 1.57–1.38 (m, 6H), 0.94 (t, 3H, *J* = 7.2 Hz). ^13^C{^1^H} NMR (100 MHz) δ 143.0, 140.6, 140.0, 137.4, 135.4, 129.8 (2C), 124.4 (2C), 121.4, 62.0, 31.82, 31.76, 29.5, 28.6, 22.5, 21.4, 14.0. HRMS (ESI) *m*/*z* calcd for C_18_H_27_O_2_S [M + H]^+^ 307.1726, found 307.1717.

##### (2*E*,4*Z*)-*N*,*N*-Diethyl-4-((*R*)-*p*-tolylsulfinyl)nona-2,4-dien-1-amine, (*E*,*Z*)-**1l**

From (*S*,*Z*)-1-((1-iodohex-1-en-1-yl)sulfinyl)-4-methylbenzene^18^ (300 mg, 0.861 mmol), (*E*)-*N*,*N*-diethyl-3-(tributylstannyl)prop-2-en-1-amine^23^ (450 mg, 1.12 mmol), BHT (190 mg, 0.861 mmol), Ph_3_As (26 mg, 0.086 mmol), and Pd_2_(dba)_3_·CHCl_3_ (45 mg, 0.043 mmol) in THF (15 mL) according to method A (20 h), (*E,Z*)-**1l** was obtained. Purification by column chromatography (0.5–4% MeOH-CHCl_3_) afforded (*E,Z*)-**1l** (257 mg, 0.771 mmol, yield: 89%) as a brown oil. Data for (*E,Z*)-**1l**: *R*_*f*_ 0.33 (10% MeOH-CHCl_3_). [α]_D_^20^ +141.7 (*c* = 1.00). ^1^H NMR (400 MHz) δ 7.40 (dm, 2H, *J* = 8.2 Hz), 7.26 (d, 2H, *J* = 8.2 Hz), 6.22 (t, 1H, *J* = 7.8 Hz), 6.07–5.89 (m, 2H), 3.05 (dd, 1H, *J* = 14.2, 6.1 Hz), 2.96 (dd, 1H, *J* = 14.2, 7.2 Hz), 2.76–2.67 (m, 1H), 2.56–2.47 (m, 1H), 2.37 (s, 3H), 2.35–2.23 (m, 4H), 1.55–1.39 (m, 4H), 0.95 (t, 3H, *J* = 7.2 Hz), 0.89 (t, 6H, *J* = 7.2 Hz). ^13^C{^1^H} NMR (100 MHz) δ 142.8, 140.5, 140.2, 138.0, 132.4, 129.8 (2C), 124.4 (2C), 123.4, 55.3, 46.6 (2C), 31.8, 28.7, 22.5, 21.4, 14.0, 11.8 (2C). HRMS (ESI) *m*/*z* calcd for C_20_H_32_NOS [M + H]^+^ 334.2199; found 334.2204.

##### 1-Methyl-4-((*R*)-((2*E*,4*Z*)-nona-2,4-dien-4-yl)sulfinyl)benzene, (*E*,*Z*)-**1m**

From (*S*,*Z*)-1-((1-iodohex-1-en-1-yl)sulfinyl)-4-methylbenzene^18^ (65 mg, 0.186 mmol), (*E*)-prop-1-en-1-ylboronic acid (32 mg, 0.373 mmol) [previously obtained by treatment of *trans*-1-propenylboronic acid MIDA ester with an aqueous solution of NaOH],^24^ CsF (113 mg, 0.744 mmol), and Pd(PPh_3_)_4_ (22 mg, 0.019 mmol) in THF (3 mL) according to method C (17 h), (*E,Z*)-**1m** was obtained. Purification by column chromatography (5–20% EtOAc-hexane) afforded (*E,Z*)-**1m** (44 mg, 0.168 mmol, yield: 90%) as an orange oil. Data for (*E,Z*)-**1m**: *R*_*f*_ 0.37 (20% EtOAc-hexane). [α]_D_^20^ −159.6 (*c* = 1.10). ^1^H NMR (400 MHz), COSY δ 7.42 (d, 2H, *J* = 8.3 Hz), 7.27 (d, 2H, *J* = 8.3 Hz), 6.14 (t, 1H, *J* = 7.9 Hz), 6.03–5.95 (m, 1H), 5.84 (brd, 1H, *J* = 15.6 Hz), 2.74–2.65 (m, 1H), 2.54–2.44 (m, 1H), 2.39 (s, 3H), 1.66 (dd, 3H, *J* = 6.6, 1.4 Hz), 1.54–1.36 (m, 4H), 0.94 (t, 3H, *J* = 7.2 Hz). ^13^C{^1^H} NMR (100 MHz), HSQC, HMBC δ 143.2, 140.5, 140.2, 136.9, 130.8, 129.8 (2C), 124.4 (2C), 122.0, 31.8, 28.6, 22.5, 21.5, 18.7, 14.0. IR (film): 2962, 2923, 2868, 1643, 1490, 1449, 1081, 1048, 960, 807, 752, 623, 554, 499 cm^–1^. HRMS (ESI) *m*/*z* calcd for C_16_H_23_OS [M + H]^+^ 263.1464; found 263.1461.

##### (*S*,*Z*)-1-((1-(Cyclohex-1-en-1-yl)hex-1-en-1-yl)sulfinyl)-4-methylbenzene, (*Z*)-**1n**

From (*S*,*Z*)-1-((1-iodohex-1-en-1-yl)sulfinyl)-4-methylbenzene^18^ (250 mg, 0.718 mmol), commercially available cyclohex-1-en-1-ylboronic acid (181 mg, 1.436 mmol), CsF (436 mg, 2.872 mmol), and Pd(PPh_3_)_4_ (166 mg, 0.144 mmol) in THF (10 mL) according to method C (18 h), (*Z*)-**1n** was obtained. Purification by column chromatography (10–40% EtOAc-hexane) afforded (*Z*)-**1n** (212 mg, 0.701 mmol, yield: 98%) as a brown oil. Data for (*Z*)-**1n**: *R*_*f*_ 0.22 (2% EtOAc-CH_2_Cl_2_). [α]_D_^20^ −44.9 (*c* = 1.00). ^1^H NMR (400 MHz), COSY δ 7.39 (d, 2H, *J* = 8.4 Hz), 7.24 (d, 2H, *J* = 8.2 Hz), 6.03 (dd, 1H, *J* = 8.4, 7.1 Hz), 5.76–5.73 (m, 1H), 2.74–2.64 (m, 1H), 2.58–2.49 (m, 1H), 2.38 (s, 3H), 1.98–1.88 (m, 3H), 1.67–1.57 (m, 1H), 1.52–1.36 (m, 8H), 0.93 (t, 3H, *J* = 7.4 Hz). ^13^C{^1^H} NMR (100 MHz), HSQC δ 146.8, 140.5, 140.2, 138.9, 131.6, 129.8, 129.5 (2C), 124.5 (2C), 31.9, 29.4, 28.3, 25.6, 22.7, 22.4, 21.7, 21.5, 14.0. IR (film): 2929, 2852, 1624, 1594, 1492, 1446, 1084, 1045, 919, 840, 807, 705, 623, 535, 502 cm^–1^. HRMS (ESI) *m*/*z* calcd for C_19_H_27_OS [M + H]^+^ 303.1777; found 303.1778.

##### (3*E*,5*Z*)-2,6-Dimethyl-5-((*S*)-*p*-tolylsulfinyl)deca-3,5-dien-2-ol, (*E*,*Z*)-**1o**

From (*S*,*E*)-((1-iodo-2-methylhex-1-en-1-yl)sulfinyl)benzene^11^ (103 mg, 0.284 mmol), (*E*)-2-methyl-4-(tributylstannyl)but-3-en-2-ol (139 mg, 0.370 mmol), BHT (63 mg, 0.284 mmol), Ph_3_As (9 mg, 0.028 mmol), and Pd_2_(dba)_3_·CHCl_3_ (15 mg, 0.014 mmol) in THF (7 mL) according to method A (60 °C, 20 h), (*E*,*Z*)-**1o** was obtained. Purification by column chromatography (5–40% EtOAc-hexane) afforded (*E*,*Z*)-**1o** (71 mg, 0.222 mmol, yield: 78%) as a brown oil. Data for (*E*,*Z*)-**1o**: *R*_*f*_ 0.25 (60% EtOAc-hexane). [α]_D_^20^ −66.9 (*c* = 0.25). ^1^H NMR (500 MHz) δ 7.36 (d, 2H, *J* = 7.9 Hz), 7.24 (d, 2H, *J* = 7.8 Hz), 5.89 (s, 2H), 2.79–2.62 (m, 2H), 2.38 (s, 3H), 1.92 (s, 3H), 1.68–1.33 (m, 5H), 1.18 (s, 3H), 1.14 (s, 3H), 0.97 (t, 3H, *J* = 7.2 Hz). ^13^C{^1^H} NMR (125 MHz) δ 149.1, 145.7, 140.4, 140.3, 137.1, 129.5 (2C), 124.7 (2C), 116.2, 71.1, 34.9, 31.3, 29.7, 29.6, 22.9, 21.4, 20.9, 14.1. HRMS (ESI) *m*/*z* calcd for C_19_H_28_NaO_2_S [M + Na]^+^ 343.1702, found 343.1704.

##### (2*E*,4*Z*)-3-Methyl-4-((*S*)-*p*-tolylsulfinyl)nona-2,4-dien-1-ol, (*E*,*Z*)-**1p**

From (*S*,*Z*)-1-((1-iodohex-1-en-1-yl)sulfinyl)-4-methylbenzene^18^ (163 mg, 0.50 mmol), (*E*)-3-(tributylstannyl)but-2-en-1-ol^25^ (235 mg, 0.65 mmol), and Pd(CH_3_CN)_2_Cl_2_ (26 mg, 0.10 mmol) in DMF (1.3 ml) according to method B (48 h), (*E*,*Z*)-**1p** was obtained. Purification by column chromatography (5–60% EtOAc-hexane) afforded (*E*,*Z*)-**1p** (84 mg, 0.287 mmol, yield: 58%) as a brown oil. Data for (*E*,*Z*)-**1p**: *R*_*f*_ 0.30 (60% EtOAc-hexane). [α]_D_^20^ −134.2 (*c* = 0.83). ^1^H NMR (400 MHz) δ 7.39 (d, 2H, *J* = 8.7 Hz), 7.26 (d, 2H, *J* = 8.6 Hz), 6.11 (dd, 1H, *J* = 8.5, 7.1 Hz), 5.55 (td, 1H, *J* = 6.7, 1.3 Hz), 4.06 (m, 2H), 2.76–2.67 (m, 1H), 2.58–2.49 (m, 1H), 2.38 (s, 3H), 1.88 (brs, 1H), 1.55 (s, 3H), 1.53–1.38 (m, 4H), 0.95 (t, 3H, *J* = 7.2 Hz). ^13^C{^1^H} NMR (100 MHz) δ 147.4, 140.6, 140.3, 140.0, 132.6, 131.7, 129.7 (2C), 124.4 (2C), 59.2, 31.7, 28.5, 22.5, 21.5, 17.8, 14.0. HRMS (ESI) *m*/*z* calcd for C_17_H_25_O_2_S [M + H]^+^ 293.1570; found 293.1555.

##### (3*E*,5*Z*)-6-Cyclohexyl-2-methyl-5-((*R*)-*p*-tolylsulfinyl)hexa-3,5-dien-2-ol, (*E*,*Z*)-**1q**

From (*S*,*E*)-1-((2-cyclohexyl-1-iodovinyl)sulfinyl)-4-methylbenzene (240 mg, 0.600 mmol), (*E*)-2-methyl-4-(tributylstannyl)but-3-en-2-ol (313 mg, 0.833 mmol), BHT (141 mg, 0.641 mmol), Ph_3_As (20 mg, 0.064 mmol), and Pd_2_(dba)_3_·CHCl_3_ (33 mg, 0.032 mmol) in THF (20 mL) according to method A (20 h), (*E,Z*)-**1q** was obtained. Purification by column chromatography (40–80% Et_2_O-hexane) afforded (*E,Z*)-**1q** (171 mg, 0.510 mmol, yield: 86%) as a white solid. Data for (*E,Z*)-**1q**: *R*_*f*_ 0.30 (40% EtOAc-hexane). [α]_D_^20^ −71.6 (*c* = 0.76). Mp: 97–97 °C. ^1^H NMR (400 MHz) δ 7.40 (d, 2H, *J* = 8.2 Hz), 7.26 (d, 2H, *J* = 8.1 Hz), 6.11–5.99 (m, 3H), 3.08–2.98 (m, 1H), 2.38 (s, 3H), 1.88–1.66 (m, 6H), 1.42–1.30 (m, 2H), 1.27–1.20 (m, 2H), 1.19 (s, 3H), 1.17 (s, 3H). ^13^C{^1^H} NMR (100 MHz) δ 143.8, 142.4, 140.7, 140.6, 139.9, 129.8 (2C), 124.6 (2C), 118.0, 71.0, 37.8, 33.12, 33.05, 29.6, 29.5, 25.8, 25.6, 25.4, 21.4. HRMS (ESI) *m*/*z* calcd for C_20_H_28_O_2_S [M + H]^+^ 333.1883; found 333.1878.

##### (2*E*,4*Z*)-4-((*R*)-*p*-Tolylsulfinyl)octa-2,4-diene-1,8-diol, (*E*,*Z*)-**1r**

From (*E,Z*)-**1e** (208 mg, 0.4 mmol) in MeOH (2 mL) and acetyl chloride (9 μL, 0.120 mmol) according to method D (20 h), (*E,Z*)-**1r** was obtained. Purification by column chromatography (100% EtOAc) afforded (*E,Z*)-**1r** (100 mg, 0.357 mmol, yield: 89%) as a colorless oil. Data for **1r**: *R*_*f*_ 0.12 (100% Et_2_OAc). [α]_D_^20^ −92.3 (*c* = 1.17). ^1^H NMR (400 MHz) δ 7.43 (brd, 2H, *J* = 7.3 Hz), 7.27 (brd, 2H, *J* = 7.3 Hz), 6.30–6.02 (m, 3H), 4.08 (brd, 2H, *J* = 4.3 Hz), 3.70 (brt, 2H, *J* = 5.8 Hz), 2.87–2.74 (m, 1H), 2.71–2.56 (m, 2H), 2.38 (s, 3H), 1.83–1.71 (m, 2H). ^13^C{^1^H} NMR (100 MHz) δ 142.8, 141.0, 139.2, 138.3, 134.2, 130.0 (2C), 124.4 (2C), 121.9, 63.0, 61.4, 32.1, 25.4, 21.5. HRMS (ESI) *m*/*z* calcd for C_15_H_21_O_3_S [M + H]^+^ 281.1206; found 281.1222.

##### (2*E*,4*Z*)-4-((*R*)-*p*-Tolylsulfinyl)nona-2,4-diene-1,9-diol, (*E*,*Z*)-**1s**

From (*E,Z*)-**1f** (207 mg, 0.388 mmol) in MeOH (2.4 mL) and acetyl chloride (8 μL, 0.117 mmol) according to method D (20 h), (*E,Z*)-**1s** was obtained. Purification by column chromatography (100% EtOAc) afforded (*E,Z*)-**1s** (106 mg, 0.360 mmol, yield: 93%) as a colorless oil. Data for (*E,Z*)-**1s**: *R*_*f*_ 0.10 (100% Et_2_OAc). [α]_D_^20^ −84.0 (*c* = 0.83). ^1^H NMR (400 MHz) δ 7.42 (brd, 2H, *J* = 8.2 Hz), 7.28 (brd, 2H, *J* = 8.1 Hz), 6.27–6.03 (m, 3H), 4.08 (brd, 2H, *J* = 5.1 Hz), 3.69 (m, 2H), 2.82–2.71 (m, 1H), 2.60–2.50 (m, 1H), 2.39 (s, 3H), 1.84 (brs, 2H), 1.72–1.57 (m, 4H). ^13^C{^1^H} NMR (100 MHz) δ 142.7, 140.9, 139.6, 138.3, 134.1, 130.2 (2C), 124.4 (2C), 121.8, 63.3, 62.5, 32.2, 28.7, 25.8, 21.5. HRMS (ESI) *m*/*z* calcd for C_16_H_23_O_3_S [M + H]^+^ 295.1362; found 295.1364.

#### General Procedures for the Base-Promoted Sulfoxide-Sulfenate Rearrangement and for the Synthesis of 2-Methoxy-2-phenylacetates

##### Method E

To a suspension of 6 equiv of NaH or KH (washed with hexanes) in toluene (10 mL/mmol of diene) under Ar and at rt was added ^*i*^PrOH (2 equiv). After 10 min, the mixture was cooled to 0 °C and then a solution of the starting sulfinyl diene in toluene (15 mL/mmol) was added dropwise and the mixture was stirred to reach rt. When the starting material disappears (TLC), a saturated solution of NH_4_Cl was added (2 mL/mmol) and the aqueous layer was extracted with CH_2_Cl_2_, dried with Na_2_SO_4_, and evaporated under vacuum. The crude mixture was purified by column chromatography on silica gel using as eluent the mixtures of solvents indicated.

##### Method F

To a solution of 1.0 equiv of alcohol in CH_2_Cl_2_ (20 mL/mmol of alcohol) were added (*R*)- or (*S*)-2-methoxy-2-phenylacetic acid (1.5–4.0 equiv), EDC**·**HCl [1-ethyl-3-(3-dimethyl aminopropyl)carbodiimide hydrochloride, 1.5–4.0 equiv], and 0.1–0.2 equiv of dimethylaminopyridine (DMAP). The reaction was stirred at room temperature until disappearance of starting material (TLC) adding increasing amounts of reagents (up to 4.0 equiv) when necessary to reach completion. The mixture was then washed with H_2_O (50 mL/mmol of alcohol), saturated NaHCO_3_ solution (50 mL/mmol of alcohol), and H_2_O (50 mL/mmol of alcohol). The aqueous layers were extracted with CH_2_Cl_2_ (1 × 4 mL), and the combined organic layers were dried over Na_2_SO_4_, filtered, and concentrated under vacuum to obtain a crude product that was purified by column chromatography on silica gel. Integration of ^1^H NMR of the crude mixtures indicated the diastereomeric and enantiomeric ratios.

##### (2*S*,3*E*,5*E*)-Nona-3,5-diene-1,2-diol, **4a**

From (*E*,*Z*)-**1a** (100 mg, 0.359 mmol) in toluene (3.6 mL), NaH (52 mg, 2.154 mmol), and ^*i*^PrOH (55 μL, 0.718 mmol) according to method E (2 h), **4a** was obtained. Purification by column chromatography (20–80% Et_2_O-CH_2_Cl_2_) afforded **4a** (35 mg, 0.224 mmol, yield: 62%) as a colorless oil. Alternatively from (*E*,*E*)-**1b** (29 mg, 0.104 mmol), following the same procedure (2 h), **4a** was obtained (11 mg, 0.070 mmol, yield: 67%). Data for **4a**: *R*_*f*_ 0.21 (50% EtOAc-hexane). [α]_D_^20^ +9.5 (*c* = 0.51). ^1^H NMR (500 MHz) COSY δ 6.29 (dd, 1H, *J* = 15.4, 10.5 Hz, H-4), 6.02 (dd, 1H, *J* = 15.3, 10.5 Hz, H-5), 5.73 (apdt, 1H, *J* = 14.2, 7.1 Hz, H-6), 5.54 (dd, 1H, *J* = 15.3, 6.5 Hz, H-3), 4.28–4.25 (m, 1H, H-2), 3.65 (dd, 1H, *J* = 11.2, 3.6 Hz, H-1), 3.51 (dd, 1H, *J* = 11.7, 7.4 Hz, H-1), 2.15 (brs, 1H, OH), 2.07–2.04 (m, 2H, H-7), 1.63 (brs, 1H, OH), 1.43–1.39 (m, 2H, H-8), 0.90 (t, 3H, *J* = 7.4 Hz, H-9). ^13^C{^1^H} NMR (125 MHz) δ 136.5, 133.1, 129.5, 128.7, 73.1, 66.6, 34.8, 22.5, 13.8. IR (film): 3367, 2925, 1660, 1450, 1379, 1338, 1263, 1075, 988, 872, 809, 741, 586 cm^–1^. HRMS (ESI) *m*/*z* calcd for C_9_H_16_NaO_2_ [M + Na]^+^ 179.1043; found 179.1041.

##### (2*S*,3*E*,5*E*)-Nona-3,5-diene-1,2-diyl (2*S*,2′*S*)-bis(2-Methoxy-2-phenylacetate), **6a** and (2*R*,3*E*,5*E*)-Nona-3,5-diene-1,2-diyl (2*S*,2′*S*)-bis(2-Methoxy-2-phenylacetate), **7a**

From **4a** (4 mg, 0.026 mmol), generated from (*E*,*Z*)-**1a**, in CH_2_Cl_2_ (1.0 mL), (*S*)-(+)-2-methoxy-2-phenylacetic acid (18 mg, 0.104 mmol), EDC**·**HCl (20 mg, 0.104 mmol), and DMAP (0.4 mg, 0.0026 mmol) according to method F (20 h), a 93:7 mixture **6a**:**7a** was obtained. Purification by column chromatography (5–10% EtOAc-hexane) afforded **6a** (6 mg, 0.013 mmol, 51%), a mixture **6a**:**7a** (1 mg, 0.002 mmol, 8%), and **7a** (1 mg, 0.002 mmol, 8%) as colorless oils (67% combined yield). From **4a** (10 mg, 0.058 mmol), generated from (*E*,*E*)-**1b**, in CH_2_Cl_2_ (3 mL), (*S*)-(+)-2-methoxy-2-phenylacetic acid (29 mg, 0.173 mmol), EDC**·**HCl (33 mg, 0.173 mmol), and DMAP (0.7 mg, 0.006 mmol) according to method F (20 h), a 98:2 mixture **6a**:**7a** was obtained (22 mg, 0.049 mmol, yield: 85%). Data for major **6a**: *R*_*f*_ 0.27 (20% EtOAc-hexane). [α]_D_^20^ +66.5 (*c* = 0.71). ^1^H NMR (400 MHz), COSY δ 7.48–7.42 (m, 2H, ArH), 7.41–7.35 (m, 2H, ArH), 7.34–7.27 (m, 6H, ArH), 6.19 (dd, 1H, *J* = 15.3, 10.4 Hz, H-4), 5.90 (dd, 1H, *J* = 15.3, 10.4 Hz, H-5), 5.69 (dt, 1H, *J* = 14.0, 6.8 Hz, H-6), 5.59–5.52 (m, 1H, H-2), 5.32 (dd, 1H, *J* = 15.3, 7.3 Hz, H-3), 4.74 (s, 1H, H-α), 4.49 (s, 1H, H-α), 4.28 (dd, 1H, *J* = 11.8, 3.8 Hz, H-1), 3.93 (dd, 1H, *J* = 11.8, 7.5 Hz, H-1), 3.36 (s, 3H, OMe), 3.33 (s, 3H, OMe), 2.05 (m, 2H, H-7), 1.40 (m, 2H, H-8), 0.90 (t, 3H, *J* = 7.3 Hz, H-9). ^13^C{^1^H} NMR (100 MHz), HSQC δ 170.3, 170.0, 138.0 (C-6), 136.4 (C-4), 136.2, 135.8, 129.0, 128.9, 128.84, 128.77 (2C), 128.7 (2C), 127.4 (2C), 127.3 (2C), 122.7 (C-3), 82.7, 82.2, 72.8 (C-2), 65.2 (C-1), 57.6, 57.5, 34.8, 22.3, 13.8. IR (film): 3064, 2959, 2931, 1753, 1659, 1495, 1455, 1352, 1266, 1199, 1174, 1115, 993, 852, 736, 698, 606 cm^–1^. HRMS (ESI) *m*/*z* calcd for C_27_H_36_NO_6_ [M + NH_4_]^+^ 470.2537; found 470.2537. Δ*H*_1_ = 4.28 – 3.93 = +0.35 ppm; Δ*H*_α_ = 4.74 – 4.49 = +0.25 ppm. Data for minor **7a**: *R*_*f*_ 0.19 (20% EtOAc-hexane). [α]_D_^20^ +39.0 (*c* = 0.10). ^1^H NMR (500 MHz) δ 7.38–7.34 (m, 10 H, ArH), 5.83–5.74 (m, 2H, H-4, H-5), 5.50–5.44 (m, 2H, H-2, H-6), 5.21 (dd, 1H, *J* = 14.7, 6.4 Hz, H-3), 4.69 (s, 1H, H-α), 4.65 (s, 1H, H-α), 4.24 (dd, 1H, *J* = 11.9, 3.7 Hz, H-1), 4.16 (dd, 1H, *J* = 11.9, 7.2 Hz, H-1), 3.38 (s, 6H, OMe), 2.01–1.97 (m, 2H, H-7), 1.39–1.35 (m, 2H, H-8), 0.88 (t, 3H, *J* = 7.1 Hz, H-9). ^13^C{^1^H} NMR (125 MHz) δ 170.4, 169.7, 137.6, 136.3, 136.1, 134.8, 129.0 (2C), 128.9, 128.84 (2C), 128.82 (2C), 127.6 (2C), 127.4 (2C), 122.5, 82.6, 82.5, 72.4, 65.3, 57.6, 57.5, 34.8, 22.4, 13.9. IR (film): 3032, 2958, 2930, 1754, 1659, 1495, 1455, 1258, 1198, 1173, 1116, 993, 851, 735, 698, 603 cm^–1^. HRMS (ESI) *m*/*z* calcd for C_27_H_36_NO_6_ [M + NH_4_]^+^ 470.2537, found 470.2544. Δ*H*_1_ = 4.24 – 4.16 = +0.08 ppm; Δ*H*_α_ = 4.69 – 4.65 = +0.04 ppm; Δ*H*_3_(**6***SS*-**7***RS*) = 5.32 – 5.21 = +0.11 ppm.

##### (3*S*,4*E*,6*E*)-2-Methyldeca-4,6-diene-2,3-diol, **4b**

From (*E,Z*)-**1c** (500 mg, 1.63 mmol) in toluene (24 mL), NaH (235 mg, 9.80 mmol), and ^*i*^PrOH (0.249 mL, 3.26 mmol) according to method E (4 h), **4b** was obtained. Purification by column chromatography (20–70% EtOAc-hexane) afforded **4b** (218 mg, 1.18 mmol, yield: 73%). Alternatively from (*E,E*)-**1d** (100 mg, 0.46 mmol) following the same procedure **4b** (5 h) was obtained (57.6 mg, 0.32 mmol, yield: 68%). Data for **4b**: *R*_*f*_ 0.38 (40% EtOAc-hexane). [α]_D_^20^ −27.2 (*c* = 1.08). ^1^H NMR (500 MHz), COSY δ 6.25 (dd, 1H, *J* = 15.3, 10.4 Hz), 6.03 (dd, 1H, *J* = 15.2, 10.4 Hz), 5.72 (m, 1H), 5.58 (dd, 1H, *J* = 15.4, 7.4 Hz), 3.91 (d, 1H, *J* = 7.4 Hz), 2.18 (brs, 1H), 2.09–2.04 (m, 2H), 1.45–1.38 (m, 2H), 1.21 (s, 3H), 1.15 (s, 3H), 0.90 (t, 3H, *J* = 7.4 Hz). ^13^C{^1^H} NMR (125 MHz), HSQC δ 136.1, 133.9, 129.6, 128.8, 79.7, 73.1, 34.8, 26.5, 24.0, 22.5, 13.9. IR (film): 3401, 2963, 2873, 1659, 1464, 1379, 1166, 1093, 1028, 989, 896, 854, 809, 739, 606, 572 cm^–1^. HRMS (ESI) *m*/*z* calcd for C_11_H_20_NaO_2_ [M + Na]^+^ 207.1355; found 207.1363.

##### (3*S*,4*E*,6*E*)-2-Hydroxy-2-methyldeca-4,6-dien-3-yl (*S*)-2-Methoxy-2-phenylacetate, **6b** and (3*S*,4*E*,6*E*)-2-Hydroxy-2-methyldeca-4,6-dien-3-yl (*R*)-2-Methoxy-2-phenylacetate, **6b′**

From **4b** (5 mg, 0.027 mmol), generated from (*E*,*Z*)-**1c**, in CH_2_Cl_2_ (1.0 mL), (*S*)-(−)-2-methoxy-2-phenylacetic acid (9 mg, 0.054 mmol), EDC**·**HCl (10 mg, 0.054 mmol), and DMAP (0.7 mg, 0.0054 mmol) according to method F (24 h), a 95:5 mixture **6b**:**7b** was obtained. Purification by column chromatography (20–40% EtOAc-hexane) afforded **6b** (4.5 mg, 0.014 mmol, 52%) and mixture **6b**:**7b** (1 mg, 0.002 mmol, 11%) as colorless oils (63% combined yield). Also from **4b** (7 mg, 0.038 mmol), generated from (*E*,*Z*)-**1c**, in CH_2_Cl_2_ (1.5 mL), (*R*)-(+)-2-methoxy-2-phenylacetic acid (13 mg, 0.076 mmol), EDC**·**HCl (15 mg, 0.076 mmol), and DMAP (0.9 mg, 0.0076 mmol) according to method F (7 h), a 95:5 mixture **6b′**:**7b′** was obtained. Purification by column chromatography (20–40% EtOAc-hexane) afforded **6b′** (6 mg, 0.018 mmol, 48%) and mixture **6b′**:**7b′** (2 mg, 0.006 mmol, 16%) as colorless oils (64% combined yield). Alternatively from **4b**, generated from (*E*,*E*)-**1d**, (*S*)-(+)-2-methoxy-2-phenylacetic acid a 95:5 mixture of the diastereomeric esters **6b**:**7b** was obtained as colorless oils. Data for major **6b**: *R*_*f*_ 0.52 (40% EtOAc-hexane). [α]_D_^20^ +38.3 (*c* = 0.38). ^1^H NMR (400 MHz), COSY δ 7.47–7.43 (m, 2H, ArH), 7.40–7.31 (m, 3H, ArH), 6.26 (dd, 1H, *J* = 15.5, 10.5 Hz, H-4), 5.99 (dd, 1H, *J* = 14.9, 10.5 Hz, H-5), 5.73 (dt, 1H, *J* = 15.3, 6.4 Hz, H-6), 5.50 (dd, 1H, *J* = 15.3, 8.2 Hz, H-4), 5.11 (d, 1H, *J* = 8.3 Hz), 4.78 (s, 1H, H-α), 3.42 (s, 3H, OMe), 2.08–2.03 (m, 2H), 1.57 (br s, 1H), 1.46–1.40 (m, 2H), 0.98 (s, 3H), 0.92 (s, 3H), 0.90 (t, 3H, *J* = 7.3 Hz). ^13^C{^1^H} NMR (100 MHz), HSQC δ 169.8, 137.4, 136.7, 136.4, 129.3, 129.1, 129.0 (2C), 127.4 (2C), 124.0, 82.7, 81.7, 72.3, 57.5, 34.9, 25.8, 24.8, 22.4, 13.9. IR (film): 3474, 2962, 2932, 1746, 1658, 1495, 1456, 1377, 1260, 1177, 1116, 992, 735, 699 cm^–1^. HRMS (ESI) *m*/*z* calcd for C_20_H_28_NaO_4_ [M + Na]^+^ 355.1880; found 355.1877. Data for major **6b′**: *R*_*f*_ 0.50 (40% EtOAc-hexane). [α]_D_^20^ −62.5 (*c* = 0.42). ^1^H NMR (400 MHz), COSY δ 7.47–7.43 (dm, 2H, *J* = 7.4 Hz), 7.39–7.33 (m, 3H), 5.93–5.84 (m, 2H), 5.53 (m, 1H), 5.39 (m, 1H, H-4), 5.15 (d, 1H, *J* = 7.1 Hz), 4.82 (s, 1H, H-α), 3.43 (s, 3H, OMe), 2.03–1.98 (m, 2H), 1.62 (br s, 1H), 1.42–1.33 (m, 2H), 1.15 (s, 3H), 1.14 (s, 3H), 0.89 (t, 3H, *J* = 7.1 Hz). ^13^C{^1^H} NMR (100 MHz), HSQC δ 169.9, 137.0, 136.4, 135.5, 129.2, 128.9, 128.8 (2C), 127.3 (2C), 123.6 (C-4), 82.9, 81.3, 72.3, 57.6, 34.8, 26.2, 25.1, 22.4, 13.8. IR (film): 3474, 2962, 2932, 1746, 1658, 1495, 1456, 1377, 1260, 1177, 1116, 992, 735, 699 cm^–1^. HRMS (ESI) *m*/*z* calcd for C_20_H_28_NaO_4_ [M + Na]^+^ 355.1880; found 355.1884. Δ*H*_1_(**6***SS*-**6′***SR*) = 0.92 – 1.14 = −0.22 ppm; Δ*H*_1_(**6***SS*-**6′***SR*) = 0.98 – 1.15 = −0.17 ppm; Δ*H*_4_(**6***SS*-**6′***SR*) = 5.50 – 5.39 = +0.11 ppm.

##### (2*S*,3*E*,5*E*)-8-((*tert*-Butyldiphenylsilyl)oxy)octa-3,5-diene-1,2-diol, **4c**

From (*E,Z*)-**1e** (100 mg, 0.193 mmol) in toluene (2.9 mL), NaH (28 mg, 1.157 mmol), and ^*i*^PrOH (30 μL, 0.386 mmol) according to method E (3 h), **4c** was obtained. Purification by column chromatography (20–60% Et_2_O-CH_2_Cl_2_) afforded **4c** (50 mg, 0.126 mmol, yield: 66%) as a colorless oil. Data for **4c**: *R*_*f*_ 0.40 (20% EtOAc-hexane). [α]_D_^20^ +9.9 (*c* = 0.92). ^1^H NMR (400 MHz) δ 7.67–7.65 (m, 4H), 7.44–7.35 (m, 6H), 6.28 (dd, 1H, *J* = 15.3, 10.3 Hz), 6.06 (dd, 1H, *J* = 15.1, 10.3 Hz), 5.73 (dd, 1H, *J* = 15.3, 6.7 Hz), 5.55 (dd, 1H, *J* = 15.1, 6.5 Hz), 4.27 (m, 1H), 3.70 (t, 2H, *J* = 6.7 Hz), 3.66 (dd, 1H, *J* = 11.2, 3.5 Hz), 3.50 (dd, 1H, *J* = 11.2, 7.3 Hz), 2.37–2.31 (m, 2H), 2.09 (brs, 1H), 1.96 (brs, 1H), 1.05 (s, 9H). ^13^C{^1^H} NMR (100 MHz) δ 135.7 (4C), 134.0, 132.8, 132.6, 131.2, 129.7 (2C), 129.3, 127.8 (4C), 73.1, 66.6, 63.6, 36.1, 27.0 (3C), 19.4. HRMS (ESI) *m*/*z* calcd for C_24_H_32_NaO_3_Si [M + Na]^+^ 419.2013; found 419.2012.

##### (2*S*,3*E*,5*E*)-8-((*tert*-Butyldiphenylsilyl)oxy)octa-3,5-diene-1,2-diyl (2*S*,2′*S*)-bis(2-Methoxy-2-phenylacetate), **6c** and (2*R*,3*E*,5*E*)-8-((*tert*-Butyldiphenylsilyl)oxy)octa-3,5-diene-1,2-diyl (2*S*,2′*S*)-bis(2-Methoxy-2-phenylacetate), **7c**

From **4c** (6.4 mg, 0.016 mmol), generated from (*E*,*Z*)-**1e**, in CH_2_Cl_2_ (1.7 mL), (*S*)-(+)-2-methoxy-2-phenylacetic acid (8 mg, 0.048 mmol), EDC**·**HCl (9 mg, 0.048 mmol) and DMAP (1 mg, 0.009 mmol) according to method F (23 h), a 92:8 mixture **6c**:**7c** was obtained. Purification by column chromatography (10–40% Et_2_O-hexane) afforded **6c** (6 mg, 0.009 mmol, 55%), mixture **6c**:**7c** (1 mg, 0.001 mmol, 9%), and **7c** (1 mg, 0.001 mmol, 9%) as colorless oils (73% combined yield). Data for major **6c**: *R*_*f*_ 0.34 (40% Et_2_O-hexane). [α]_D_^20^ +59.2 (*c* = 0.84). ^1^H NMR (400 MHz) δ 7.62–7.60 (m, 6H), 7.39–7.22 (m, 14H), 6.13 (dd, 1H, *J* = 15.3, 10.3 Hz), 5.88 (dd, 1H, *J* = 15.3, 10.8 Hz), 5.64 (dt, 1H, *J* = 15.3, 7.0 Hz), 5.51 (m, 1H), 5.31 (dd, 1H, *J* = 15.3, 7.2 Hz), 4.70 (s, 1H, H-α), 4.45 (s, 1H, H-α), 4.23 (dd, 1H, *J* = 11.6, 3.9 Hz, H-1), 3.89 (dd, 1H, *J* = 11.6, 7.7 Hz, H-1), 3.65 (t, 2H, *J* = 6.6 Hz), 3.32 (s, 3H), 3.29 (s, 3H), 2.27 (m, 2H), 1.01 (s, 9H). ^13^C{^1^H} NMR (100 MHz) δ 170.3, 170.0, 136.3, 136.1, 135.7 (4C), 135.6, 134.2, 134.0, 130.7, 129.8 (2C), 128.89, 128.85, 128.78 (2C), 128.72 (2C), 127.8 (4C), 127.34 (2C), 127.30 (2C), 123.3, 82.7, 82.2, 72.7, 65.2, 63.4, 57.59, 57.55, 36.1, 27.0 (3C), 19.3. HRMS (ESI) *m*/*z* calcd for C_42_H_52_NO_7_Si [M + NH_4_]^+^ 710.3508; found 710.3526. Δ*H*_1_ = 4.23 – 3.89 = +0.34 ppm; Δ*H*_α_ = 4.70 – 4.45 = +0.25 ppm. Partial data for minor **7c**: *R_f_* 0.22 (40% Et_2_O-hexane). ^1^H NMR (400 MHz) δ 7.66–7.63 (m, 4H), 7.40–7.30 (m, 16H), 5.79 (m, 2H), 5.48 (m, 2H), 5.20 (dd, 1H, *J* = 14.5, 6.4 Hz), 4.68 (s, 1H, H-α), 4.64 (s, 1H, H-α), 4.24 (dd, 1H, *J* = 12.6, 3.6 Hz, H-1), 4.14 (dd, 1H, *J* = 12.0, 7.1 Hz, H-1), 3.67–3.61 (m, 2H), 3.37 (s, 6H, OMe), 2.36–2.17 (m, 2H), 1.04 (s, 9H). HRMS (ESI) *m*/*z* calcd for C_42_H_52_NO_7_Si [M + NH_4_]^+^ 710.3508; found 710.3476. Δ*H*_1_ = 4.24 – 4.14 = +0.1 ppm; Δ*H*_α_ = 4.68 – 4.64 = +0.04 ppm; Δ*H*_3_(**6***SS*-**7***RS*) = 5.28 – 5.20 = +0.08 ppm.

##### (2*S*,3*E*,5*E*)-9-((*tert*-Butyldiphenylsilyl)oxy)nona-3,5-diene-1,2-diol, **4d**

From (*E,Z*)-**1f** (500 mg, 0.938 mmol) in toluene (14 mL), NaH (135 mg, 5.63 mmol), and ^*i*^PrOH (0.143 mL, 1.876 mmol) according to method E (2 h), **4d** was obtained. Purification by column chromatography (10–50% Et_2_O-CH_2_Cl_2_) afforded **4d** (304 mg, 0.740 mmol, yield: 79%) as a colorless oil. Data for **4d**: *R*_*f*_ = 0.29 (40% Et_2_O-CH_2_Cl_2_). [α]_D_^20^ +25.5 (*c* = 0.33). ^1^H NMR (400 MHz) δ 7.66 (dm, 4H, *J* = 7.9 Hz), 7.44–7.36 (m, 6H), 6.27 (dd, 1H, *J* = 15.6, 10.3 Hz), 6.02 (dd, 1H, *J* = 14.6, 10.1 Hz), 5.71 (dt, 1H, *J* = 15.3, 7.0 Hz), 5.54 (dd, 1H, *J* = 15.6, 7.0 Hz), 4.28 (m, 1H), 3.67 (m, 3H), 3.51 (dd, 1H, *J* = 11.1, 7.0 Hz), 2.19 (m, 2H), 2.06 (brs, 1H), 1.92 (brs, 1H), 1.65 (m, 2H), 1.05 (s, 9H). ^13^C{^1^H} NMR (100 MHz) δ 136.0, 135.7 (4C), 134.1, 133.0, 129.7 (2C), 129.6, 128.9, 127.8 (4C), 73.1, 66.6, 63.3, 32.1, 29.1, 27.0 (3C), 19.4. HRMS (ESI) *m*/*z* calcd for C_25_H_34_NaO_3_Si [M + Na]^+^ 433.2169; found 433.2156.

##### (2*S*,3*E*,5*E*)-9-((*tert*-Butyldiphenylsilyl)oxy)nona-3,5-diene-1,2-diyl (2*S*,2′*S*)-bis(2-Methoxy-2-phenylacetate), **6d** and (2*R*,3*E*,5*E*)-9-((*tert*-Butyldiphenylsilyl)oxy)nona-3,5-diene-1,2-diyl (2*S*,2′*S*)-bis(2-Methoxy-2-phenylacetate), **7d**

From **4d** (4.0 mg, 0.01 mmol), generated from (*E*,*Z*)-**1f**, in CH_2_Cl_2_ (1.7 mL), (*S*)-(+)-2-methoxy-2-phenylacetic acid (5 mg, 0.03 mmol), EDC**·**HCl (5.6 mg, 0.03 mmol), and DMAP (0.25 mg, 0.002 mmol) according to method F (23 h), a 90:10 mixture **6d**:**7d** was obtained. Purification by column chromatography (20–50% Et_2_O-hexane) afforded **6d** (5 mg, 0.007 mmol, 71%), mixture **6d**:**7d** (0.5 mg), and **7d** (0.5 mg) as colorless oils (85% combined yield). Data for major **6d**: *R*_*f*_ 0.30 (40% Et_2_O-hexane). [α]_D_^20^ +42.5 (*c* = 0.42). ^1^H NMR (500 MHz) δ 7.65 (m, 4H), 7.46–7.30 (m, 16H), 6.16 (dd, 1H, *J* = 15.3, 10.2 Hz), 5.90 (dd, 1H, *J* = 14.9, 10.4 Hz), 5.67 (dt, 1H, *J* = 14.2, 6.2 Hz), 5.55 (m, 1H), 5.31 (dd, 1H, *J* = 15.7, 7.8 Hz), 4.74 (s, 1H, H-α), 4.49 (s, 1H, H-α), 4.27 (dd, 1H, *J* = 12.7, 3.5 Hz, H-1), 3.93 (dd, 1H, *J* = 12.7, 8.1 Hz, H-1), 3.66 (t, 2H, *J* = 8.1 Hz), 3.36 (s, 3H), 3.33 (s, 3H), 2.18 (m, 2H), 1.64 (m, 2H), 1.05 (s, 9H). ^13^C{^1^H} NMR (125 MHz) δ 170.3, 170.0, 137.3, 136.3, 136.2, 135.73, 135.71 (4C), 134.1, 129.7 (2C), 129.1, 128.89, 128.85, 128.79 (2C), 128.7 (2C), 127.8 (4C), 127.4 (2C), 127.3 (2C), 122.8, 82.7, 82.2, 72.8, 65.2, 63.2, 57.60, 57.55, 32.0, 29.0, 27.0 (3C), 19.4. HRMS (ESI) *m*/*z* calcd for C_43_H_54_NO_7_Si [M + NH_4_]^+^ 724.3664; found 724.3647. Δ*H*_1_ = 4.27 – 3.93 = +0.34 ppm; Δ*H*_α_ = 4.74 – 4.49 = +0.25 ppm. Partial data for minor **7d**: *R*_*f*_ 0.27 (20% Et_2_O-hexane). ^1^H NMR (400 MHz) δ 7.66–7.63 (m, 4H), 7.40–7.30 (m, 16H), 5.80–5.74 (m, 2H), 5.49–5.44 (m, 2H), 5.19 (dd, 1H, *J* = 14.9, 6.3 Hz), 4.69 (s, 1H, H-α), 4.65 (s, 1H, H-α), 4.24 (dd, 1H, *J* = 11.7, 3.5 Hz, H-1), 4.17 (dd, 1H, *J* = 11.7, 7.1 Hz, H-1), 3.63 (t, 2H, *J* = 6.6 Hz), 3.38 (s, 6H, OMe), 2.17–2.08 (m, 2H), 1.62–1.58 (m, 2H), 1.04 (s, 9H). Δ*H*_1_ = 4.24 – 4.17 = +0.07 ppm; Δ*H*_α_ = 4.69 – 4.65 = +0.04 ppm; Δ*H*_3_(**6***SS*-**7***RS*) = 5.31 – 5.19 = +0.12 ppm.

##### (3*S*,4*E*,6*E*)-10-((*tert*-Butyldiphenylsilyl)oxy)-2-methyldeca-4,6-diene-2,3-diol, **4e**

From (*E,Z*)-**1g** (110 mg, 0.196 mmol) in toluene (2.9 mL), NaH (28 mg, 1.177 mmol), and ^*i*^PrOH (30 μL, 0.392 mmol) according to method E (4 h), **4e** was obtained. Purification by column chromatography (10–30% Et_2_O-CH_2_Cl_2_) afforded **4e** (57 mg, 0.130 mmol, yield: 66%) as a colorless oil. Data for **4e**: *R*_*f*_ 0.36 (40% Et_2_O-CH_2_Cl_2_). [α]_D_^20^ +22.7 (*c* = 0.42). ^1^H NMR (500 MHz) δ 7.70–7.61 (m, 4H), 7.46–7.34 (m, 6H), 6.31–6.18 (m, 1H), 6.10–5.97 (m, 1H), 5.70 (dt, 1H, *J* = 14.6, 6.9 Hz), 5.58 (dd, 1H, *J* = 15.3, 7.4 Hz), 3.92 (d, 1H, *J* = 7.4 Hz), 3.67 (t, 2H, *J* = 6.3 Hz), 2.23–2.15 (m, 2H), 2.06 (s, 1H), 1.71–1.56 (m, 3H), 1.22 (s, 3H), 1.16 (s, 3H), 1.05 (s, 9H). ^13^C{^1^H} NMR (125 MHz) δ 135.71 (4C), 135.69, 134.1, 133.78, 129.77, 129.7 (2C), 128.9, 127.8 (4C), 79.7, 73.1, 63.3, 32.2, 29.1, 27.0 (3C), 26.5, 24.0, 19.4. HRMS (ESI) *m*/*z* calcd for C_27_H_38_NaO_3_Si [M + Na]^+^ 461.2482, found 461.2480.

##### (3*S*,4*E*,6*E*)-10-((*tert*-Butyldiphenylsilyl)oxy)-2-hydroxy-2-methyldeca-4,6-dien-3-yl (*S*)-2-methoxy-2-phenylacetate, **6e** and (3*S*,4*E*,6*E*)-10-((*tert*-Butyldiphenylsilyl)oxy)-2-hydroxy-2-methyldeca-4,6-dien-3-yl (*R*)-2-Methoxy-2-phenylacetate, **6e′**

From **4e** (5.0 mg, 0.011 mmol), generated from (*E*,*Z*)-**1g**, in CH_2_Cl_2_ (1.5 mL), (*S*)-(+)-2-methoxy-2-phenylacetic acid (3.8 mg, 0.023 mmol), EDC**·**HCl (4.4 mg, 0.023 mmol), and DMAP (1 mg, 0.002 mmol) according to method F (23 h), a 95:5 mixture **6e**:**7e** was obtained. Purification by column chromatography (20–40% Et_2_O-hexane) afforded **6e** (5.5 mg, 0.009 mmol, 82%) and **7e** (0.5 mg) as colorless oils (89% combined yield). Similarly, from **4e** (5.0 mg, 0.011 mmol) and (*R*)-(−)-2-methoxy-2-phenylacetic acid (3.8 mg, 0.023 mmol), a 95:5 mixture **6e′**:**7e′** was obtained. Purification by column chromatography (10–60% Et_2_O-hexane) afforded **6e′** (4.7 mg, 0.008 mmol, 73%) and **7e′** (0.5 mg) as colorless oils (78% combined yield). Data for major **6e**: *R*_*f*_ 0.40 (70% Et_2_O-hexane). [α]_D_^20^ +33.4 (*c* = 0.42). ^1^H NMR (500 MHz) δ 7.66 (dd, 4H, *J* = 8.1, 1.5 Hz), 7.49–7.31 (m, 11H), 6.23 (dd, 1H, *J* = 15.3, 10.4 Hz), 5.99 (dd, 1H, *J* = 15.2, 10.4 Hz), 5.70 (dt, 1H, *J* = 14.6, 6.9 Hz), 5.50 (dd, 1H, *J* = 15.3, 8.0 Hz), 5.11 (d, 1H, *J* = 8.0 Hz), 4.78 (s, 1H), 3.66 (t, 2H, *J* = 6.2 Hz), 3.42 (s, 3H), 2.24–2.13 (m, 2H), 1.65 (dq, 2H, *J* = 7.7, 6.3 Hz), 1.31 (s, 1H), 1.05 (s, 9H), 0.98 (s, 3H), 0.93 (s, 3H). ^13^C{^1^H} NMR (125 MHz) δ 169.8, 136.9, 136.7, 136.3, 135.7 (4C), 134.1, 129.7 (2C), 129.4, 129.1, 128.9 (2C), 127.8 (4C), 127.4 (2C), 124.1, 82.7, 81.6, 72.3, 63.3, 57.5, 32.1, 29.1, 27.0 (3C), 25.8, 24.8, 19.4. HRMS (ESI) *m*/*z* calcd for C_36_H_46_NaO_5_Si [M + Na]^+^ 609.3007, found 609.2983. Data for major **6e′**: *R*_*f*_ 0.39 (80% Et_2_O-hexane). [α]_D_^20^ −13.3 (*c* = 0.33). ^1^H NMR (300 MHz) δ 7.66 (dm, 4H, *J* = 7.7 Hz), 7.45–7.30 (m, 11H), 5.93–5.83 (m, 2H), 5.49 (m, 1H), 5.38 (m, 1H), 5.15 (d, 1H, *J* = 7.1 Hz), 4.82 (s, 1H), 3.64 (t, 2H, *J* = 6.3 Hz), 3.42 (s, 3H), 2.14 (q, 2H, *J* = 7.2 Hz), 1.66–1.57 (m, 3H), 1.14 (s, 6H), 1.06 (s, 9H). ^13^C{^1^H} NMR (125 MHz) δ 169.9, 136.5, 136.4, 135.71 (4C), 135.69, 135.4, 134.1, 129.7 (2C), 129.4, 128.9, 128.8 (2C), 127.7 (4C), 127.3 (2C), 123.8, 82.9, 81.3, 72.3, 63.3, 57.6, 32.1, 29.0, 27.0 (3C), 26.2, 25.1, 19.4. HRMS (ESI) *m*/*z* calcd for C_36_H_46_NaO_5_Si [M + Na]^+^ 609.3007, found 609.2979. Δ*H*_1_(**6***SS*-**6′***SR*) = 0.93 – 1.14 = −0.21 ppm; Δ*H*_1_(**6***SS*-**6′***SR*) = 0.98–1.14 = −0.16 ppm; Δ*H*_4_(**6***SS*-**6′**) = 5.50 – 5.38 = +0.12 ppm.

##### (2*S*,3*E*,5*E*)-5-(Cyclohex-2-en-1-ylidene)pent-3-ene-1,2-diol, **4f**

From (*E,Z*)-**1h** (40 mg, 0.132 mmol) in toluene (2.4 mL), KH (32 mg, 0.794 mmol), and ^*i*^PrOH (20 μL, 0.264 mmol) at 0 °C, according to method E (3 h), **4f** was obtained. Purification by column chromatography (40–100% Et_2_O-CH_2_Cl_2_) afforded **4f** (10 mg, 0.055 mmol, yield: 42%). Data for **4f**: *R*_*f*_ 0.34 (80% Et_2_O-CH_2_Cl_2_). ^1^H NMR (400 MHz), COSY δ 6.64 (dd, 1H, *J* = 15.3, 11.2 Hz, H-4), 6.08 (dt, 1H, *J* = 9.8, 1.7 Hz, H-2′), 5.90–5.82 (m, 2H, H-5, H-3′), 5.63 (dd, 1H, *J* = 15.3, 6.6 Hz, H-3), 4.33 (m, 1H, H-2), 3.68 (dd, 1H, *J* = 11.2, 3.3 Hz, H-1), 3.53 (dd, 1H, *J* = 11.2, 7.3 Hz, H-1), 2.47 (m, 2H, H-6′), 2.19–2.12 (m, 3H, OH, 2 H-4′), 1.96 (brs, 1H, OH), 1.72 (m, 2H). NOESY-2D: H-2/H-4, H-2/H-3, H-4/H-3, H-4/H-6′. ^13^C{^1^H} NMR (100 MHz), HSQC δ 138.2, 131.00, 130.98, 130.3, 128.6, 124.7, 73.4, 66.7, 25.9, 25.6, 22.4. HRMS (ESI) *m*/*z* calcd for C_11_H_16_NaO_2_ [M + Na]^+^ 203.1043; found 203.1045.

##### (2*S*,3*E*,5*E*)-5-(Cyclohex-2-en-1-ylidene)pent-3-ene-1,2-diyl (2*S*,2′*S*)-bis(2-Methoxy-2-phenylacetate), **6f** and (2*R*,3*E*,5*E*)-5-(Cyclohex-2-en-1-ylidene)pent-3-ene-1,2-diyl (2*S*,2′*S*)-bis(2-Methoxy-2-phenylacetate), **7f**

From **4f** (5 mg, 0.028 mmol), generated from (*E*,*Z*)-**1h**, in CH_2_Cl_2_ (1.2 mL), (*S*)-(+)-2-methoxy-2-phenylacetic acid (14 mg, 0.083 mmol), EDC**·**HCl (16 mg, 0.083 mmol), and DMAP (0.6 mg, 0.0056 mmol) according to method F (4 h), an 83:17 mixture **6f**:**7f** was obtained. Purification by column chromatography (10–40% Et_2_O-hexane) afforded **6f** (3 mg, 0.006 mmol, 23%), mixture **6f**:**7f** (1 mg, 0.002 mmol, 8%), and **7f** (1.5 mg, 0.003 mmol, 11%) as colorless oils (42% combined yield). Data for major **6f**: *R*_*f*_ 0.34 (40% Et_2_O-CH_2_Cl_2_). ^1^H NMR (400 MHz), COSY δ 7.47–7.28 (m, 10H), 6.54 (ddd, 1H, *J* = 15.1, 11.4, 0.9 Hz), 6.05 (dd, 1H, *J* = 9.9, 1.7 Hz), 5.89 (m, 1H), 5.71 (dd, 1H, *J* = 11.4, 10.8 Hz), 5.61 (m, 1H), 5.39 (dd, 1H, *J* = 15.1, 7.5 Hz), 4.75 (s, 1H, H-α), 4.50 (s, 1H, H-α), 4.30 (dd, 1H, *J* = 11.8, 3.7 Hz, H-1), 3.96 (dd, 1H, *J* = 11.8, 7.5 Hz, H-1), 3.36 (s, 3H), 3.33 (s, 3H), 2.40–2.33 (m, 2H), 2.19–2.12 (m, 2H), 1.70 (m, 2H). ^13^C{^1^H} NMR (100 MHz), HSQC δ 170.4, 170.0, 139.5, 136.4, 136.2, 131.8, 131.4, 130.8, 128.87, 128.86, 128.79 (2C), 128.72 (2C), 127.34 (2C), 127.30 (2C), 124.2, 124.0, 82.7, 82.3, 73.1, 65.3, 57.60, 57.56, 25.9, 25.6, 22.4. HRMS (ESI) *m*/*z* calcd for C_29_H_32_NaO_6_[M + Na]^+^ 499.2091; found 499.2081. Δ*H*_1_ = 4.30 – 3.96 = +0.34 ppm; Δ*H*_α_ = 4.75 – 4.50 = +0.25 ppm. Data for minor **7f**: *R*_*f*_ 0.24 (40% Et_2_O-CH_2_Cl_2_). ^1^H NMR (400 MHz), COSY δ 7.40–7.34 (m, 10H), 6.22 (dd, 1H, *J* = 15.2, 11.4 Hz), 6.00 (d, 1H, *J* = 9.8 Hz), 5.86 (m, 1H), 5.63 (d, 1H, *J* = 11.5 Hz), 5.54 (m, 1H), 5.30 (dd, 1H, *J* = 15.2, 6.6 Hz), 4.70 (s, 1H, H-α), 4.65 (s, 1H, H-α), 4.28 (dd, 1H, *J* = 11.8, 3.6 Hz, H-1), 4.18 (dd, 1H, *J* = 11.8, 7.1 Hz, H-1), 3.38 (s, 6H), 2.19–2.11 (m, 4H), 1.65 (m, 2H). ^13^C NMR{^1^H} (100 MHz), HSQC δ 170.4, 169.7, 139.0, 136.2, 136.1, 131.5, 130.8, 130.2, 128.93, 128.87, 128.79 (4C), 127.5 (2C), 127.4 (2C), 124.3, 124.0, 82.5, 82.4, 72.6, 65.3, 57.51, 57.48, 25.8, 25.5, 22.3. HRMS (ESI) *m*/*z* calcd for C_29_H_32_NaO_6_[M + Na]^+^ 499.2091; found 499.2085. Δ*H*_1_ = 4.28 – 4.18 = +0.1 ppm; Δ*H*_α_ = 4.70 – 4.65 = +0.05 ppm; Δ*H*_3_(**6***SS*-**7***RS*) = 5.39 – 5.30 = +0.09 ppm.

##### (3*R*,4*E*,6*E*)-Deca-4,6-diene-1,3-diol, **4g** and (7*S*,3*E*,5*E*)-Deca-3,5-diene-1,7-diol, **5g**

From (*E,Z*)-**1i** (25 mg, 0.085 mmol) in toluene (1.5 mL), NaH (12 mg, 0.510 mmol), and ^*i*^PrOH (13μL, 0.170 mmol), according to method E (21 h), an 88:12 mixture of **4g** and **5g** was obtained. Purification by column chromatography (20–60% EtOAc-hexane) afforded **4g** (9.5 mg, 0.056 mmol, 66%) and **5g** (1.5 mg, 0.009 mmol, 10%) as colorless oils (76% combined yield). Data for **4g**: *R*_*f*_ 0.31 (20% EtOAc-hexane). [α]_D_^20^ −18.6 (*c* = 0.58). ^1^H NMR (400 MHz), COSY δ 6.22 (dd, 1H, *J* = 15.5, 10.3 Hz), 6.03 (dd, 1H, *J* = 14.9, 10.3 Hz), 5.72 (dt, 1H, *J* = 14.9, 6.6 Hz), 5.62 (dd, 1H, *J* = 15.8, 6.9 Hz), 4.41 (m, 1H), 3.90–3.79 (m, 2H), 2.25 (br s, 2H, OH), 2.06 (m, 2H), 1.80 (m, 2H), 1.41 (m, 2H), 0.90 (t, 3H, *J* = 7.6 Hz). ^13^C{^1^H} NMR (100 MHz), HSQC δ 136.0, 132.8, 131.1, 129.5, 72.7, 61.4, 38.7, 34.9, 22.5, 13.8. IR (film): 3352, 2958, 1660, 1435, 1379, 1338, 1053, 988, 666 cm^–1^. HRMS (ESI) *m*/*z* calcd for C_10_H_19_O_2_ [M + H]^+^ 171.1380; found 171.1373. Data for **5g**: *R*_*f*_ 0.42 (20% EtOAc-hexane). ^1^H NMR (400 MHz), COSY δ 6.23–6.10 (m, 2H), 5.71–5.61 (m, 2H), 4.14 (m, 1H, H-7), 3.69 (t, 2 H *J* = 6.3 Hz, H-1), 2.36 (m, 2H), 1.58–1.30 (m, 6H), 0.93 (t, 3H, *J* = 7.5 Hz). ^13^C{^1^H} NMR (125 MHz), HSQC δ 135.2, 132.6, 130.5, 130.3, 72.6, 62.1, 39.6, 36.2, 18.8, 14.1. HRMS (ESI) *m*/*z* calcd for C_10_H_22_NO_2_ [M + NH_4_]^+^ 188.1645; found 188.1652.

##### (3*R*,4*E*,6*E*)-Deca-4,6-diene-1,3-diyl (2*S*,2′*S*)-bis(2-Methoxy-2-phenylacetate), **6g** and (3*R*,4*E*,6*E*)-Deca-4,6-diene-1,3-diyl (2*R*,2′*R*)-bis(2-Methoxy-2-phenylacetate), **6g**′

From **4g** (3 mg, 0.018 mmol), generated from (*E*,*Z*)-**1i**, in CH_2_Cl_2_ (1 mL), (*S*)-(+)-2-methoxy-2-phenylacetic acid (9 mg, 0.054 mmol), EDC**·**HCl (10 mg, 0.054 mmol), and DMAP (0.2 mg, 0.0018 mmol) according to method F (3 h), a 95:5 mixture **6g**:**7g** was obtained. Purification by column chromatography (10–20% EtOAc-hexane) afforded mixture **6g**:**7g** (5 mg, 0.011 mmol, 63% combined yield) as a colorless oil. Also from **4g** (3 mg, 0.018 mmol), generated from (*E*,*Z*)-**1i**, in CH_2_Cl_2_ (1.2 mL), (*R*)-(−)-2-methoxy-2-phenylacetic (9 mg, 0.054 mmol), EDC**·**HCl (10 mg, 0.054 mmol), and DMAP (0.2 mg, 0.0018 mmol) according to method F (3 h), a 95:5 mixture **6g′**:**7g′** was obtained. Purification by column chromatography (5–20% EtOAc-hexane) afforded **6g′** (4 mg, 0.009 mmol, 48%), mixture **6g′**:**7g′** (2 mg, 0.004 mmol, 23%), and **7g′** (1 mg, 0.002 mmol, 12%) as colorless oils (83% combined yield). Data for major **6g**: *R*_*f*_ 0.27 (20% EtOAc-hexane). ^1^H NMR (400 MHz), COSY δ 7.44–7.28 (m, 10H), 6.12 (dd, 1H, *J* = 14.7, 10.6 Hz), 5.93 (dd, 1H, *J* = 15.4, 10.6 Hz), 5.69 (dt, 1H, *J* = 14.7, 5.0 Hz), 5.40 (dd, 1H, *J* = 15.4, 7.6 Hz, H-4), 5.28 (m, 1H), 4.70 (s, 1H), 4.69 (s, 1H), 3.98–3.92 (m, 1H), 3.70–3.64 (m, 1H), 3.38 (s, 3H), 3.37 (s, 3H), 2.05 (m, 2H), 1.81 (m, 2H, H-2), 1.36–1.45 (m, 2H), 0.90 (t, 3H, *J* = 7.6 Hz). Partial data of ^13^C{^1^H} NMR (100 MHz) from HSQC δ 137.3, 134.4, 128.9, 126.5, 82.5 (2C), 72.1, 60.7, 57.1 (2C), 34.6, 32.9, 22.1, 13.6. HRMS (ESI) *m*/*z* calcd for C_28_H_34_NaO_6_[M + Na]^+^ 489.2248; found 489.2240. Data for major **6g′**: *R*_*f*_ 0.23 (20% EtOAc-hexane). [α]_D_^20^ +57.8 (*c* = 0.21). ^1^H NMR (400 MHz), COSY δ 7.45–7.31 (m, 10H), 5.80 (dd, 1H, *J* = 15.1, 10.4 Hz), 5.67 (dd, 1H, *J* = 15.1, 10.4 Hz), 5.46 (dt, 1H, *J* = 15.1, 6.9 Hz), 5.26 (m, 1H), 5.19 (dd, 1H, *J* = 15.1, 7.0 Hz, H-4), 4.74 (s, 2H, H-α), 4.13–4.04 (m, 2H), 3.40 (s, 6H), 1.98 (m, 2H), 1.95–1.88 (m, 1H, H-2), 1.86–1.79 (m, 1H, H-2), 1.40–1.33 (m, 2H), 0.87 (t, 3H, *J* = 7.2 Hz). ^13^C{^1^H} NMR (100 MHz), HSQC δ 170.6, 169.9, 137.0, 136.33, 136.30, 133.7, 129.0, 128.93, 128.87 (2C), 128.78 (2C), 127.43 (2C), 127.36 (2C), 126.4, 82.8, 82.5, 72.0, 61.2, 57.48, 55.51, 34.8, 33.4, 22.4, 13.8. HRMS (ESI) *m*/*z* calcd for C_28_H_38_NO_6_[M + NH_4_]^+^ 484.2694; found 484.2706. Δ*H*_2_(**6***RS*-**6′***RR*) = 1.81–1.87 = −0.06 ppm; Δ*H*_4_(**6***RS*-**6′***RR*) = 5.40 – 5.19 = +0.21 ppm.

##### (7*RS*,3*E*,5*E*)-Deca-3,5-diene-1,7-diyl (2*R*,2′*R*)-bis(2-Methoxy-2-phenylacetate), **5g**′ and **5g″**

From **5g** (1.5 mg, 0.009 mmol), generated from (*E,Z*)-**1i**, and (*R*)-(−)-2-methoxy-2-phenylacetic acid (4.5 mg, 0.027 mmol), EDC**·**HCl (5.2 mg, 0.027 mmol) and DMAP (0.11 mg, 0.0009 mmol) according to method F (4 h), a 73:27 mixture of the diastereomeric esters **5g′**:**5g″** was obtained as colorless oils (2.39 mg, 0.005 mmol, 57%). A parallel result was obtained using (*S*)-(+)-2-methoxy-2-phenylacetic acid. Partial data for **5g′**and **5g″** from the mixture: *R*_*f*_ 0.28 (20% EtOAc-hexane). ^1^H NMR (500 MHz) δ 7.44–7.31 (m, 10H), 6.08 (dd, 1H, *J* = 15.2, 10.1 Hz, minor), 5.91 (dd, 1H, *J* = 15.2, 11.0 Hz, minor), 5.83–5.73 (m, 2H), 5.52–5.44 (2H, major), 4.75 (s, 1H, H-α, minor), 4.74 (s, 1H, H-α, major), 4.16 (t, 2H, *J* = 6.9 Hz, H-1 minor), 4.13 (t, 2H, *J* = 6.9 Hz, H-1 major), 2.39–2.28 (m, 2H).

##### (*R*,5*E*,7*E*)-Undeca-5,7-diene-1,4-diol, **4h** and (*S*,4*E*,6*E*)-Undeca-4,6-diene-1,8-diol, **5h**

From (*E,Z*)-**1j** (30 mg, 0.098 mmol) in toluene (1.8 mL), NaH (14 mg, 0.587 mmol), and ^*i*^PrOH (15 μL, 0.196 mmol), according to method E (22 h), a complex mixture (75:25) of regioisomers was obtained that partially decomposed under manipulation. Purification by column chromatography (5–20% EtOAc-hexane) afforded slightly impure **4h** (2.7 mg, 0.015 mmol, 15%). Data for **4h**: *R*_*f*_ 0.27 (20% EtOAc-Et_2_O). ^1^H NMR (300 MHz) δ 6.19 (dd, 1H, *J* = 15.3, 10.6 Hz), 6.02 (dd, 1H, *J* = 14.8, 10.5 Hz), 5.70 (dt, 1H, *J* = 14.5, 7.0 Hz), 5.59 (dd, 1H, *J* = 15.1, 6.9 Hz), 4.18 (m, 1H), 3.68 (m, 2H), 2.10–1.89 (m, 4H), 1.67 (m, 4H), 1.41 (m, 2H), 0.91 (t, 3H, *J* = 7.2 Hz). HRMS (ESI) *m*/*z* calcd for C_11_H_20_NaO_2_[M + Na]^+^ 207.1356, found 207.1347.

##### (2*S*,3*E*,5*E*)-1-(Diethylamino)nona-3,5-dien-2-ol, **4i** and (4*S*,5*E*,7*E*)-9-(Diethylamino)nona-5,7-dien-4-ol, **5i**

From (*E,Z*)-**1l** (25 mg, 0.075 mmol) in toluene (1.6 mL), KH (18 mg, 0.450 mmol), and ^*i*^PrOH (11 μL, 0.150 mmol), according to method E (22 h), a 74:26 mixture of **4i** and **5i** was obtained. Purification by column chromatography using silica gel deactivated by washing with a 5% solution of NaHCO_3_ in MeOH (1–6% MeOH-CHCl_3_) afforded **4i** (5.5 mg, 0.026 mmol, 34%) and **5i** (4 mg, 0.019 mmol, 25%) as colorless oils (combined yield: 59%). Data for **4i**: *R*_*f*_ 0.44 (3% MeOH-CHCl_3_). ^1^H NMR (400 MHz), COSY δ 6.26 (dd, 1H, *J* = 15.2, 10.4 Hz), 6.03 (dd, 1H, *J* = 15.2, 10.4 Hz), 5.68 (dt, 1H, *J* = 14.4, 7.0 Hz), 5.48 (dd, 1H, *J* = 15.2, 6.6 Hz), 4.07 (m, 1H), 2.70–2.60 (m, 2H), 2.56–2.42 (m, 3H), 2.36–2.26 (m, 2H), 2.05 (m, 2H), 1.40 (m, 2H), 1.02 (t, 6H, *J* = 7.1 Hz), 0.89 (t, 3H, *J* = 7.3 Hz). ^13^C{^1^H} NMR (100 MHz), HSQC δ 135.2, 132.7, 131.1, 129.9, 67.9, 59.5, 47.0 (2C), 34.8, 22.6, 13.8, 12.1 (2C). HRMS (ESI) *m*/*z* calcd for C_13_H_26_NO [M + H]^+^ 212.2009; found 212.2008. Data for **5i**: *R*_*f*_ 0.18 (3% MeOH-CHCl_3_). ^1^H NMR (400 MHz), COSY δ 6.25–6.11 (m, 2H), 5.74 (dt, 1H, *J* = 14.6, 6.2 Hz), 5.64 (dd, 1H, *J* = 14.5, 6.9 Hz), 4.14 (q, 1H, *J* = 6.5 Hz), 3.13 (d, 2H, *J* = 6.9 Hz), 2.52 (q, 4H, *J* = 7.2 Hz), 1.70–1.30 (m, 5H), 1.03 (t, 6H, *J* = 7.2 Hz), 0.93 (t, 3H, *J* = 7.2 Hz). ^13^C{^1^H} NMR (100 MHz), HSQC δ 135.4, 132.1, 131.7, 130.3, 72.6, 55.2, 46.8 (2C), 39.6, 18.8, 14.1, 11.8 (2C). HRMS (ESI) *m*/*z* calcd for C_13_H_26_NO [M + H]^+^ 212.2009; found 212.2008.

##### (2*S*,3*E*,5*E*)-1-(Diethylamino)nona-3,5-dien-2-yl (*S*)-2-methoxy-2-phenylacetate, **6i** and (2*R*,3*E*,5*E*)-1-(Diethylamino)nona-3,5-dien-2-yl (*S*)-2-methoxy-2-phenylacetate, **6i′**

From **4i** (2.5 mg, 0.012 mmol), generated from (*E,Z*)-**1l**, in CH_2_Cl_2_ (1.3 mL), (*S*)-(+)-2-methoxy-2-phenylacetic acid (3 mg, 0.018 mmol), EDC**·**HCl (3.5 mg, 0.018 mmol), and DMAP (0.2 mg, 0.0012 mmol) according to method F (7 h), a 65:35 mixture **6i**:**6i′** was obtained. Purification by column chromatography (0.5–1% MeOH-CH_2_Cl_2_) afforded **6i** (1 mg, 0.003 mmol, 25%) and **6i′** (1 mg, 0.003 mmol, 25%) as colorless oils (50% combined yield). Data for major **6i**: *R*_*f*_ 0.51 (5% MeOH-CH_2_Cl_2_). ^1^H NMR (500 MHz), COSY δ 7.46–7.44 (m, 2H), 7.36–7.31 (m, 3H), 6.18 (dd, 1H, *J* = 14.9, 10.4 Hz), 5.98 (dd, 1H, *J* = 15.4, 10.9 Hz), 5.68 (dt, 1H, *J* = 15.4, 6.8 Hz), 5.50 (dd, 1H, *J* = 14.7, 6.7 Hz, H-3), 5.45 (m 1H), 4.75 (s, 1H), 3.43 (s, 3H), 2.54 (dd, 1H, *J* = 13.3, 4.3 Hz, H-1), 2.46 (dd, 1H, *J* = 13.3, 4.3 Hz, H-1), 2.34 (m, 4H), 2.05 (m, 2H), 1.40 (m, 2H), 0.91 (t, 3H, *J* = 7.6 Hz), 0.84 (t, 6H, *J* = 6.9 Hz). HRMS (ESI) *m*/*z* calcd for C_22_H_34_NO_3_[M + H]^+^ 360.2533; found 360.2535. Partial data for minor **6i′**: *R*_*f*_ 0.73 (5% MeOH-CH_2_Cl_2_). ^1^H NMR (400 MHz), COSY δ 7.46–7.44 (m, 2H), 7.38–7.33 (m, 3H), 5.87 (ddm, 1H, *J* = 14.5, 10.3 Hz), 5.80 (dd, 1H, *J* = 14.5, 10.6 Hz), 5.49–5.44 (m, 2H), 5.39 (dd, 1H, *J* = 14.8, 6.3 Hz, H-3), 4.77 (s, 1H, H-α), 3.43 (s, 3H), 2.63 (m, 1H, H-1), 2.51 (m, 1H, H-1), 2.57–2.47 (m, 4H), 1.99 (m, 2H), 1.36 (m, 2H), 0.97 (t, 6H, *J* = 7.2 Hz), 0.88 (t, 3H, *J* = 7.6 Hz). HRMS (ESI) *m*/*z* calcd for C_22_H_34_NO_3_[M + H]^+^ 360.2533; found 360.2530. Δ*H*_1_(**6***SS*-**6′***RS*) = 2.50 – 2.57 = −0.07 ppm; Δ*H*_3_(**6***SS*-**6′***RS*) = 5.50 – 5.39 = +0.11 ppm.

##### (*R*,3*E*,5*E*)-Nona-3,5-dien-2-ol, **4j** and (*S*,5*E*,7*E*)-Nona-5,7-dien-4-ol, **5j**

From (*E,Z*)-**1m** (20 mg, 0.076 mmol) in toluene (1.8 mL), KH (18 mg, 0.456 mmol), and ^*i*^PrOH (12 μL, 0.152 mmol), according to method E (19 h), a 60:40 mixture of **4j** and **5j** was obtained. This mixture mostly decomposed under purification by column chromatography (5–10% EtOAc-hexane) affording a sample of slightly impure **4j** (3 mg, 0.021 mmol, 28%). Data for major **4j**: *R*_*f*_ 0.32 (40% Et_2_O-hexane). ^1^H NMR (500 MHz) COSY δ 6.18 (dd, 1H, *J* = 15.3, 10.3 Hz), 6.05–5.99 (m, 1H), 5.70 (dt, 1H, *J* = 15.1, 7.0 Hz), 5.62 (dd, 1H, *J* = 15.3, 6.6 Hz), 4.33 (m, 1H), 2.06 (m, 2H), 1.43–1.38 (m, 3H), 1.28 (d, 3H, *J* = 6.4 Hz), 0.90 (t, 3H, *J* = 7.2 Hz).^13^C{^1^H} NMR (125 MHz) δ 135.6, 134.7, 130.3, 129.7, 68.9, 34.9, 23.5, 22.6, 13.8. HRMS (ESI) *m*/*z* calcd for C_9_H_15_[M + H – H_2_O]^+^ 123.1168, found 123.1174.

##### (*S*,*E*)-1-(Cyclohex-1-en-1-yl)hex-1-en-3-ol, **5k** and (*R*,*E*)-2-((*E*)-Hex-2-en-1-ylidene)cyclohexan-1-ol, **4k**

From (*E,Z*)-**1n** (45 mg, 0.149 mmol) in toluene (1.5 mL), KH (36 mg, 0.894 mmol), and ^*i*^PrOH (23 μL, 0.298 mmol) according to method E (24 h), an inseparable 20:80 mixture of **4k** and **5k** was obtained. Purification by column chromatography (1–2% Et_2_O-CH_2_Cl_2_) afforded an inseparable 20:80 mixture **4k**:**5k** (18 mg, 0.1 mmol, combined yield: 67%) as a colorless oil. Data for **5k** from the mixture: *R*_*f*_ 0.27 (2% Et_2_O-CH_2_Cl_2_). ^1^H NMR (500 MHz), COSY, ROESY δ 6.19 (d, 1H, *J* = 15.6 Hz, H-1), 5.75 (m, 1H, H-2′), 5.53 (ddd, 1H, *J* = 15.7, 7.3, 0.6 Hz, H-2), 4.14 (m, 1H, H-3), 2.12 (m, 5H), 1.66–1.41 (m, 8H), 0.93 (t, 3H, *J* = 7.2 Hz). ROESY cross point between H-2′/H-3′ (2.12 ppm); H-3/H-1; H-3/H-2; H-3/H-4 (1.58). ^13^C{^1^H} NMR (125 MHz), HSQC, HMBC δ 135.2, 134.4, 130.1, 128.6, 73.3, 39.8, 26.0, 24.7, 22.64, 22.58, 18.9, 14.2. Partial data for **4k** from the mixture: *R*_*f*_ 0.27 (2% Et_2_O-CH_2_Cl_2_). ^1^H NMR (500 MHz), COSY, ROESY δ 6.29 (ddt, 1H, *J* = 14.8, 10.8, 1.0 Hz, H-2′), 6.01 (d, 1H, *J* = 10.9 Hz, H-1′) 5.70 (dd, 1H, *J* = 14.4, 7.0 Hz, H-3′), 4.14 (m, 1H, H-1), 2.58 (m, 1H), 1.84 (m, 2H), 0.91 (t, 3H, *J* = 7.2 Hz). ROESY cross point between H-1′/H–1. ^13^C{^1^H} NMR (125 MHz), HSQC, HMBC δ 134.9, 134.6, 125.6, 121.0, 73.7, 22.77, 22.54, 13.9. HRMS (ESI) *m*/*z* calcd for C_12_H_19_O[M – H]^−^ 179.1441; found 179.1436.

##### (3*S*,*E*)-2-Methyl-6-methylenedec-4-ene-2,3-diol, **4l** and (*S*,*Z*)-1-Methyl-4-((2-methyl-6-methylene deca-2,4-dien-5-yl)sulfinyl)benzene, **4la**

From (*E,Z*)-**1o** (52 mg, 0.162 mmol) in toluene (3.6 mL), NaH (23 mg, 0.972 mmol), and ^*i*^PrOH (25 μL, 0.324 mmol), according to method E (20 h), an 80:20 mixture of **4l** and **4la** was obtained. Purification by column chromatography (10–60% EtOAc-hexane) afforded **4l** (18 mg, 0.091 mmol, 56%) and **4la** (8 mg, 0.026 mmol, 16%) as colorless oils (combined yield: 72%). Data for **4l**: *R*_*f*_ 0.30 (40% EtOAc-hexane). [α]_D_^20^ −10.5 (*c* = 0.40). ^1^H NMR (400 MHz) δ 6.30 (dt, 1H, *J* = 15.9, 0.9 Hz), 5.71 (ddd, 1H, *J* = 15.9, 7.5, 0.6 Hz), 5.07–4.93 (m, 2H), 3.96 (d, 1H, *J* = 7.7 Hz), 2.32–2.07 (m, 4H), 1.51–1.40 (m, 2H), 1.40–1.31 (m, 2H), 1.23 (s, 3H), 1.16 (s, 3H), 0.92 (t, 3H, *J* = 7.2 Hz). ^13^C{^1^H} NMR (100 MHz), HSQC δ 145.7, 135.5, 127.1, 116.2, 80.0, 73.1, 31.9, 30.5, 26.6, 24.0, 22.7, 14.1. HRMS (ESI) *m*/*z* calcd for C_12_H_22_NaO_2_ [M + Na]^+^ 221.1512; found 221.1506. Data for **4la**: *R*_*f*_ 0.60 (40% EtOAc-hexane). ^1^H NMR (400 MHz) δ 7.46 (d, 2H, *J* = 8.5 Hz), 7.23 (d, 2H, *J* = 8.5 Hz), 7.14 (d, 1H, *J* = 11.2 Hz), 6.01 (dm, 1H, *J* = 11.5 Hz), 5.07 (s, 1H), 4.64 (s, 1H), 2.38 (s, 3H), 1.96 (m, 1H), 1.94 (s, 3H), 1.89–1.81 (m, 1H), 1.85 (s, 3H), 1.24–1.17 (m, 4H), 0.80 (d, 3H, *J* = 7.1 Hz). ^13^C{^1^H} NMR (100 MHz), HSQC δ 144.3, 143.1, 141.5, 141.4, 140.5, 129.6 (2C), 126.3, 125.9 (2C), 120.3, 118.4, 36.4, 29.5, 26.8, 22.4, 21.6, 19.1, 14.0. HRMS (ESI) *m*/*z* calcd for C_19_H_27_OS [M + H]^+^ 303.1777; found 303.1776.

##### (3*S*,*E*)-2-Hydroxy-2-methyl-6-methylenedec-4-en-3-yl (*S*)-2-Methoxy-2-phenylacetate, **6l** and (3*S*,*E*)-2-Hydroxy-2-methyl-6-methylenedec-4-en-3-yl (*R*)-2-Methoxy-2-phenylacetate, **6l′**

From **4l** (5.0 mg, 0.025 mmol), generated from (*E*,*Z*)-**1o**, in CH_2_Cl_2_ (1.5 mL), (*S*)-(+)-2-methoxy-2-phenylacetic acid (8.0 mg, 0.050 mmol), EDC**·**HCl (4.4 mg, 0.050 mmol), and DMAP (1 mg, 0.005 mmol) according to method F (20 h), a 95:5 mixture **6l**:**7l** was obtained. Purification by column chromatography (5–20% Et_2_O-hexane) afforded **6l** (5.4 mg, 0.016 mmol, 64%) and **7l** (0.5 mg) as colorless oils (68% combined yield). Similarly from **4l** (5.0 mg, 0.025 mmol) and (*R*)-(−)-2-methoxy-2-phenylacetic acid (8.0 mg, 0.050 mmol), a 95:5 mixture **6l′**:**7l′** was obtained. Purification by column chromatography (10–40% Et_2_O-hexane) afforded **6l′** (6.0 mg, 0.017 mmol, 68%) and **7l′** (0.5 mg) as colorless oils (75% combined yield). Data for major **6l**: *R*_*f*_ 0.30 (30% EtOAc-hexane). [α]_D_^20^ +60.5 (*c* = 0.42). ^1^H NMR (500 MHz) δ 7.49–7.43 (m, 2H), 7.42–7.31 (m, 3H), 6.29 (d, 1H, *J* = 15.9 Hz), 5.63 (dd, 1H, *J* = 15.9, 7.9 Hz), 5.15 (d, 1H, *J* = 7.5 Hz), 5.01 (s, 2H), 4.80 (s, 1H), 3.42 (s, 3H), 2.16 (t, 2H, *J* = 7.6 Hz), 1.48–1.28 (m, 5H), 1.00 (s, 3H), 0.94 (s, 3H), 0.91 (t, 3H, *J* = 7.2 Hz). ^13^C{^1^H} NMR (125 MHz) δ 169.8, 145.3, 137.9, 136.7, 129.1, 129.0 (2C), 127.4 (2C), 122.5, 117.2, 82.8, 81.8, 72.3, 57.5, 31.7, 30.4, 26.0, 25.0, 22.7, 14.1. HRMS (ESI) *m*/*z* calcd for C_21_H_30_NaO_4_ [M + Na]^+^ 369.2036; found 369.2042. Data for major **6l′**: *R*_*f*_ 0.30 (30% EtOAc-hexane). [α]_D_^20^ −31.2 (*c* = 0.58). ^1^H NMR (300 MHz) δ 7.46–7.32 (m, 5H), 5.97 (d, 1H, *J* = 16.0 Hz), 5.49 (dd, 1H, *J* = 16.0, 7.3 Hz), 5.19 (d, 1H, *J* = 7.1 Hz), 4.92 (s, 1H), 4.83 (s, 1H), 4.81 (s, 1H), 3.43 (s, 3H), 2.08–2.01 (m, 2H), 1.64 (s, 1H), 1.39–1.22 (m, 4H), 1.16 (s, 3H), 1.15 (s, 3H), 0.88 (t, 3H, *J* = 7.1 Hz). ^13^C{^1^H} NMR (125 MHz) δ 169.9, 145.2, 137.1, 136.3, 129.00, 128.8 (2C), 127.3 (2C), 122.1, 117.0, 82.9, 81.49, 72.3, 57.5, 31.6, 30.3, 26.3, 25.2, 22.7, 14.1. HRMS (ESI) *m*/*z* calcd for C_21_H_30_NaO_4_ [M + Na]^+^ 369.2036; found 369.2029. Δ*H*_1_(**6***SS*-**6′***SR*) = 0.94 – 1.15 = −0.21 ppm; Δ*H*_1_(**6***SS*-**6′***SR*) = 1.00 – 1.16 = −0.16 ppm; Δ*H*_4_(**6***SS*-**6′***SR*) = 5.63 – 5.49 = +0.14 ppm.

##### (*S*,3*E*,5*E*)-3-Methylnona-3,5-diene-1,2-diol, **4m**

From (*E,Z*)-**1p** (30 mg, 0.094 mmol) in toluene (2.1 mL), NaH (14 mg, 0.564 mmol), and ^*i*^PrOH (14 μL, 0.188 mmol), according to method E (20 h), **4m** was obtained. Purification by column chromatography (5–50% EtOAc-CH_2_Cl_2_) afforded **4m** (7.0 mg, 0.041 mmol, 43%) as a colorless oil. Data for **4m**: *R*_*f*_ 0.35 (20% EtOAc-CH_2_Cl_2_). ^1^H NMR (500 MHz) δ 6.29–6.20 (m, 1H), 6.11 (d, 1H, *J* = 10.6 Hz), 5.73 (dt, 1H, *J* = 14.6, 7.0 Hz), 4.18 (dd, 1H, *J* = 7.7, 3.7 Hz), 3.66 (dd, 1H, *J* = 11.1, 3.7 Hz), 3.56 (dd, 1H, *J* = 11.1, 7.6 Hz), 2.09 (q, 2H, *J* = 7.2 Hz), 1.75 (s, 3H), 1.42 (hex, 2H, *J* = 7.3 Hz), 0.91 (t, 3H, *J* = 7.3 Hz). ^13^C{^1^H} NMR (125 MHz) δ 136.0, 133.6, 126.6, 125.8, 77.2, 65.4, 35.2, 22.7, 13.9, 13.3. HRMS (ESI) *m*/*z* calcd for C_10_H_18_NaO_2_ [M + Na]^+^ 193.1199; found 193.1208.

##### (2*S*,3*E*,5*E*)-3-Methylnona-3,5-diene-1,2-diyl (2*S*,2′*S*)-bis(2-Methoxy-2-phenylacetate), **6m** and (2*R*,3*E*,5*E*)-3-Methylnona-3,5-diene-1,2-diyl (2*S*,2′*S*)-bis(2-Methoxy-2-phenylacetate), **7m**

From **4m** (6 mg, 0.035 mmol), generated from (*E*,*Z*)-**1p**, in CH_2_Cl_2_ (1.5 mL), (*S*)-(+)-2-methoxy-2-phenylacetic acid (18 mg, 0.105 mmol), EDC**·**HCl (20 mg, 0.105 mmol), and DMAP (0.9 mg, 0.007 mmol) according to method F (20 h), a 72:28 mixture **6m**:**7m** was obtained. Purification by column chromatography (5–50% EtOAc-hexane) afforded **6m** (3.3 mg, 0.007 mmol, 20%) and a mixture **6m**:**7m** (2.7 mg, 0.006 mmol, 17%) as colorless oils (37% combined yield). Data for major **6m**: *R*_*f*_ 0.30 (10% EtOAc-hexane). ^1^H NMR (300 MHz) δ 7.48–7.28 (m, 10H), 6.21–6.07 (m, 1H), 5.95 (d, 1H, *J* = 10.9 Hz), 5.65 (dt, 1H, *J* = 14.5, 7.0 Hz), 5.42 (dd, 1H, *J* = 8.0, 3.9 Hz), 4.76 (s, 1H), 4.49 (s, 1H), 4.32 (dd, 1H, *J* = 11.7, 4.0 Hz), 3.95 (dd, 1H, *J* = 11.7, 8.0 Hz), 3.36 (s, 3H), 3.32 (s, 3H), 2.12–2.01 (m, 2H), 1.66 (s, 3H), 1.48–1.38 (m, 2H), 0.91 (t, 3H, *J* = 7.4 Hz). ^13^C{^1^H} NMR (100 MHz) δ 170.4, 137.3, 131.8, 129.3, 128.80 (2C), 128.76 (2C), 127.3 (2C), 127.2 (2C), 125.4, 82.8, 82.3, 64.5, 57.7 (2C), 54.3, 35.2, 24.7, 22.6, 13.9. HRMS (ESI) *m*/*z* calcd for C_28_H_38_NO_6_[M + NH_4_]^+^ 484.2694; found 484.2684. Δ*H*_1_ = 4.32 – 3.95 = +0.37 ppm. Δ*H*_α_ = 4.76 – 4.49 = +0.27 ppm. Partial data for minor **7m**: *R*_*f*_ 0.28 (10% EtOAc-hexane). ^1^H NMR (300 MHz) δ 7.48–7.31 (m, 10H), 6.00 (dd, 1H, *J* = 15.0, 10.8 Hz), 5.57 (d, 1H, *J* = 11.0 Hz), 5.48–5.26 (m, 2H), 4.69 (s, 1H), 4.64 (s, 1H), 4.26–4.16 (m, 2H), 3.38 (s, 6H), 2.10–1.97 (m, 2H), 1.46 (s, 3H), 1.45–1.32 (m, 2H), 1.26 (s, 3H), 0.89 (t, 3H, *J* = 7.4 Hz). Δ*H*_1_ = 4.26 – 4.16 = +0.10 ppm; Δ*H*_α_ = 4.49 – 4.64 = +0.05 ppm.

##### (2*SR*,*E*)-4-((*S*)-Tetrahydrofuran-2-yl)but-3-ene-1,2-diol, **8**

From (*E,Z*)-**1r** (89 mg, 0.317 mmol, 1 equiv) in toluene (2.7 mL) and THF (0.25 mL), NaH (102 mg, 4.237 mmol, 12 equiv) and ^*i*^PrOH (162 μL, 2.118 mmol, 6 equiv) according to method E (2 h), *anti*-**8** and *syn*-**8** were obtained as an inseparable 80:20 mixture. Purification by column chromatography (1–6% MeOH-CH_2_Cl_2_) afforded the mixture of **8** (20 mg, 0.126 mmol, yield: 40%) as a colorless oil. Data for the mixture of **8**: *R*_*f*_ 0.33 (5% CH_3_OH-CH_2_Cl_2_). [α]_D_^20^ +2.8 (*c* = 0.71). ^1^H NMR (500 MHz), COSY δ 5.80 (ddd, 1H, *J* = 15.5, 6.5, 1.2 Hz, minor), 5.79 (ddd, 1H, *J* = 15.5, 6.5, 1.2 Hz, major), 5.69 (ddd, 1H, *J* = 15.5, 5.6, 1.0 Hz, major), 5.68 (ddd, 1H, *J* = 15.5, 5.6, 1.0 Hz, minor), 4.30 (apq, 1H), 4.25–4.20 (m, 1H), 3.89 (m, 1H), 3.77 (m, 1H), 3.64 (dd, 1H, *J* = 11.3, 3.5 Hz, minor), 3.63 (dd, 1H, *J* = 11.3, 3.5 Hz, major), 3.49 (dd, 1H, *J* = 11.3, 7.5 Hz, major), 3.48 (m, 1H, minor), 2.86 (brs, 1H), 2.72 (brs, 1H), 2.09–2.01 (m, 1H), 1.99–1.84 (m, 2H), 1.65–1.57 (m, 1H). ^13^C{^1^H} NMR (125 MHz), HSQC δ 133.4 (minor), 133.3 (major), 130.3 (major), 130.1 (minor), 79.21 (major), 79.15 (minor), 72.53 (minor), 72.46 (major), 68.2, 66.44 (minor), 66.37 (major), 32.2, 26.0 (major), 25.9 (minor). HRMS (ESI) *m*/*z* calcd for C_8_H_13_O_3_ [M – H]^−^ 157.0870; found 157.0880.

##### (*S*,*E*)-4-((*S*)-Tetrahydrofuran-2-yl)but-3-ene-1,2-diyl (2*S*,2′*S*)-bis(2-Methoxy-2-phenylacetate), **10a** and (*R*,*E*)-4-((*S*)-Tetrahydrofuran-2-yl)but-3-ene-1,2-diyl (2*S*,2′*S*)-bis(2-Methoxy-2-phenylacetate), **11a**

From an 80:20 mixture of *anti*-**8** and *syn*-**8** (5 mg, 0.032 mmol), generated from (*E*,*Z*)-**1r**, in CH_2_Cl_2_ (1.5 mL), (*S*)-(+)-2-methoxy-2-phenylacetic acid (16 mg), EDC**·**HCl (18 mg, 0.095 mmol), and DMAP (0.4 mg, 0.003 mmol) according to method F (4 h), an 80:20 *anti*:*syn* mixture of **10a**:**11a** was obtained. After purification by column chromatography (20–60% Et_2_O-hexane) **10a** was isolated along with a 20% of **10a′**, minor diastereomer from *anti ent*-**8** (3 mg, 0.007 mmol), and a mixture **10a**:**11a** (8 mg, 0.018 mmol) as colorless oils (76% combined yield).

Data for major **10a** and **10a′**: *R*_*f*_ 0.28 (80% Et_2_O-hexane). ^1^H NMR (500 MHz), COSY δ 7.46–7.42 (m, 2H), 7.40–7.27 (m, 8H), 5.76 (dd, 1H, *J* = 14.4, 5.6 Hz, minor), 5.72 (dd, 1H, *J* = 14.4, 5.7 Hz, major), 5.58–5.49 (m, 2H), 4.75 (s, 1H, H-α, major), 4.74 (s, 1H, H-α, minor), 4.47 (s, 1H, H-α), 4.30 (dd, 1H, *J* = 11.8, 3.3 Hz, H-1, minor), 4.28 (dd, 1H, *J* = 11.8, 3.4 Hz, H-1, major), 4.26–4.19 (m, 1H), 3.91 (dd, 1H, *J* = 11.9, 7.4 Hz, H-1, major), 3.90 (dd, 1H, *J* = 11.8, 7.4 Hz, H-1, minor), 3.86–3.81 (m, 1H), 3.78–3.73 (m, 1H), 3.36 (s, 3H), 3.33 (s, 3H, major), 3.32 (s, 3H, minor), 2.00–1.93 (m, 1H), 1.89–1.81 (m, 2H), 1.51–1.43 (m, 1H). ^13^C{^1^H} NMR (125 MHz), HSQC δ 170.3, 170.0, 137.1 (minor), 136.8 (major), 136.3, 136.1, 128.89 (2C, minor), 128.86 (2C, minor), 128.8 (2C, major), 128.7 (2C, major), 127.34 (2C), 127.29 (2C), 123.4 (major), 123.3 (minor), 82.7, 82.2, 78.34 (major), 78.28 (minor), 72.2 (minor), 72.1 (major), 68.3, 65.3, 57.6, 57.5, 32.0, 25.7. HRMS (ESI) *m*/*z* calcd for C_26_H_34_NO_7_ [M + NH_4_]^+^ 472.2330, found 472.2306. Δ*H*_1_ = 4.28–3.91 = +0.37 ppm (for major *anti*-**10a**); Δ*H*_α_ = 4.75–4.47 = +0.28 ppm (for major *anti*-**10a**). Partial data for minor *syn*-**11a** from the 80:20 mixture: 4.69 (s, 1H, H-α), 4.63 (s, 1H, H-α). Δ*H*_α_ = 4.69 – 4.63 = +0.06 ppm.

##### (2*RS*,*E*)-4-((*S*)-Tetrahydro-2*H*-pyran-2-yl)but-3-ene-1,2-diol, **9**

From (*E,Z*)-**1s** (105 mg, 0.357 mmol, 1 equiv) in toluene (2.9 mL) and THF (0.25 mL), NaH (108 mg, 4.484 mmol, 12 equiv) and ^*i*^PrOH (171 μL, 2.238 mmol, 6 equiv) according to method E (1 h), *anti*-**9** and *syn*-**9** were obtained as an inseparable 80:20 mixture. Purification by column chromatography (1–6% MeOH-CH_2_Cl_2_) afforded the mixture of **9** (33 mg, 0.192 mmol, yield: 54%) as a colorless oil. Data for the mixture **9**: *R*_*f*_ 0.30 (100% EtOAc). ^1^H NMR (500 MHz) δ 5.82 (dd, 1H, *J* = 15.8, 5.2 Hz), 5.70 (dd, 1H, *J* = 15.8, 5.9 Hz), 4.25 (brs, 1H), 4.04–3.99 (m, 1H), 3.84–3.79 (m, 1H), 3.69–3.63 (m, 1H), 3.54–3.44 (m, 2H), 2.09 (brs, 1H), 1.95 (brs, 1H), 1.89–1.82 (m, 1H), 1.70–1.63 (m, 1H), 1.60–1.48 (m, 3H), 1.40–1.32 (m, 1H). ^13^C{^1^H} NMR (125 MHz), HSQC δ 134.3, 128.8, 77.4, 72.8, 68.6, 66.5, 32.2, 25.9, 23.5. HRMS (ESI) *m*/*z* calcd for C_9_H_16_NaO_3_ [M + Na]^+^ 195.0992; found 195.0999.

##### (*S*,*E*)-4-((*S*)-Tetrahydro-2*H*-pyran-2-yl)but-3-ene-1,2-diyl (2*S*,2′*S*)-bis(2-Methoxy-2-phenylacetate)), **10b** and (*R*,*E*)-4-((*S*)-Tetrahydro-2*H*-pyran-2-yl)but-3-ene-1,2-diyl (2*S*,2′*S*)-bis(2-Methoxy-2-phenylacetate), **11b**

From an 80:20 mixture of *anti*-**9** and *syn*-**9** (6 mg, 0.035 mmol), generated from (*E*,*Z*)-**1s**, in CH_2_Cl_2_ (1.5 mL), (*S*)-(+)-2-methoxy-2-phenylacetic acid (17 mg, 0.105 mmol), EDC**·**HCl (20 mg, 0.105 mmol), and DMAP (0.5 mg, 0.004 mmol) according to method F (17 h), an 80:20 *anti*:*syn* mixture **10b**:**11b** was obtained. After purification by column chromatography (10–30% EtOAc-hexane), **10b** was isolated along with 20% of **10b′**, minor diastereomer from *anti ent*-**9** (3 mg, 0.006 mmol), and a mixture **10b**:**11b** (10 mg, 0.021 mmol) as colorless oils (79% combined yield). Data for major *anti*-**10b** and **10b′**: *R*_*f*_ 0.33 (60% Et_2_O-hexane). ^1^H NMR (500 MHz), COSY δ 7.46–7.43 (m, 2H), 7.40–7.34 (m, 3H), 7.33–7.30 (m, 3H), 7.29–7.27 (m, 2H), 5.76 (dd, 1H, *J* = 14.8, 4.9 Hz, minor), 5.73 (dd, 1H, *J* = 14.8, 4.9 Hz, major), 5.59–5.50 (m, 2H), 4.75 (s, 1H, H-α, major), 4.74 (s, 1H, H-α, minor), 4.45 (s, 1H, H-α), 4.29 (dd, 1H, *J* = 11.8, 7.5 Hz, H-1, minor), 4.27 (dd, 1H, *J* = 11.8, 7.5 Hz, H-1, major), 4.01–3.97 (m, 1H), 3.88 (dd, 1H, *J* = 11.8, 7.5 Hz, H-1, minor), 3.87 (dd, 1H, *J* = 11.8, 7.5 Hz, H-1, major), 3.75–3.67 (m, 1H), 3.47–3.41 (m, 1H), 3.36 (s, 3H), 3.33 (s, 3H, major), 3.32 (s, 3H, minor), 1.83 (m, 1H), 1.55–1.44 (m, 4H), 1.28–1.20 (m, 1H). ^13^C{^1^H} NMR (125 MHz) δ 170.3, 169.9, 137.2 (minor), 136.9 (major), 136.4, 136.2, 128.9 (2C, minor), 128.82 (2C, minor), 128.78 (2C, major), 128.7 (2C, major), 127.3 (4C), 122.84 (major), 122.76 (minor), 82.6, 82.16 (minor), 82.14 (major), 76.8 (major), 76.7 (minor), 72.3 (minor), 72.2 (major), 68.48 (major), 68.46 (minor), 65.28 (major), 65.25 (minor), 57.6, 57.5, 31.9, 25.9, 23.4. HRMS (ESI) *m*/*z* calcd for C_27_H_36_NO_7_ [M + NH_4_]^+^ 486.2486, found 486.2494. Δ*H*_1_ = 4.27 – 3.87 = +0.4 ppm (for major *anti*-**10b**); Δ*H*_α_ = 4.75 – 4.45 = +0.3 ppm (for major *anti*-**10b**). Data for minor *syn*-**11b** from the mixture 80:20 *anti*:*syn*: 4.69 (s, 1H, H-α), 4.63 (s, 1H, H-α); Δ*H*_α_ = 4.69 – 4.63 = +0.06 ppm.

##### (*S*,4*E*,6*E*)-2-Methyl-3-(prop-2-yn-1-yloxy)deca-4,6-dien-2-ol, **12** and 2-((1*S*,5*S*,7*aR*)-5-Propyl-1,3,5,7a-tetrahydroisobenzofuran-1-yl)propan-2-ol, **13**

To a solution of **4b** (45 mg, 0.244 mmol, 1 equiv) in CH_2_Cl_2_ (10 mL/mmol), propargyl bromide (105 μL, 0.977 mmol, 4.0 equiv), benzyltrimethylammonium hydroxide, 40% solution in methanol (22 μL, 0.049 mmol, 0.2 equiv), and 60% NaOH solution (10 mL/mmol) were added. The mixture was stirred at room temperature and under Ar until disappearance of the starting material (TLC, 24 h). Then, it was filtered through celite and a saturated NaCl solution was added. The aqueous layer was extracted with CH_2_Cl_2_ (3 × 5 mL), and the combined organic layers were dried over MgSO_4_, filtered, and concentrated under vacuum. The crude product was purified by column chromatography on silica gel (5–15% EtOAc-hexane) to afford **12** (37 mg, 0.166 mmol, yield: 69%) as a colorless oil. Data for **12**: *R*_*f*_ 0.32 (20% EtOAc-hexane). [α]_D_^20^ +125.1 (*c* = 0.71). ^1^H NMR (400 MHz) δ 6.23 (dd, 1H, *J* = 15.3, 10.4 Hz), 6.05 (dd, 1H, *J* = 15.3, 10.5 Hz), 5.79–5.72 (m, 1H), 5.38 (dd, 1H, *J* = 15.3, 8.9 Hz), 4.21 (dd, 1H, *J* = 15.7, 2.4 Hz), 4.01 (dd, 1H, *J* = 15.7, 2.4 Hz), 3.71 (d, 1H, *J* = 9.2 Hz), 2.46 (brs, 1H), 2.40 (m, 1H), 2.10–2.03 (m, 2H), 1.47–1.36 (m, 2H), 1.17 (s, 3H), 1.15 (s, 3H), 0.91 (t, 3H, *J* = 7.4 Hz). ^13^C{^1^H} NMR (100 MHz) δ 136.8, 136.6, 129.4, 125.9, 86.4, 80.1, 74.3, 72.3, 55.6, 34.8, 26.3, 24.6, 22.4, 13.9. HRMS (ESI) *m*/*z* calcd for C_14_H_22_NaO_2_ [M + Na]^+^ 245.1512; found 245.1509.

To a solution of **12** (30 mg, 0.135 mmol, 1 equiv) in CH_2_Cl_2_ (4 mL/mmol), Et_3_N (19 μL, 0.135 mmol, 1 equiv) and CuI (3 mg, 0.014 mmol, 0.1 equiv) were added. The mixture was stirred at room temperature and under Ar until disappearance of the starting material (TLC, 28 h). The solvent was removed under vacuum, and the crude residue was purified by chromatography on silica gel (1–4% Et_2_O-CH_2_Cl_2_) to afford **13** (24 mg, 0.108 mmol, yield: 80%) as a colorless oil. Data for **13**: *R*_*f*_ 0.30 (2% Et_2_O-CH_2_Cl_2_). [α]_D_^20^ −5.2 (*c* = 1.47). ^1^H NMR (400 MHz), COSY δ 5.83–5.77 (m, 1H), 5.74–5.69 (m, 1H), 5.47 (m, 1H), 4.44–4.38 (m, 1H), 4.33–4.27 (m, 1H), 3.37 (d, 1H, *J* = 10.2 Hz), 3.03–2.96 (m, 1H), 2.76–2.68 (m, 1H), 2.26 (brs, 1H), 1.49–1.33 (m, 4H), 1.29 (s, 3H), 1.26 (s, 3H), 0.92 (t, 3H, *J* = 7.1 Hz). ^13^C{^1^H} NMR (100 MHz), HSQC δ 138.8, 132.6, 124.6, 120.5, 88.9, 71.6, 69.8, 39.4, 37.9, 35.8, 27.7, 24.7, 19.8, 14.4. HRMS (ESI) *m*/*z* calcd for C_14_H_26_NO_2_ [M + NH_4_]^+^ 240.1958; found 240.1956.

## Data Availability

The data underlying this study are available in the published article and its Supporting Information.

## Supporting Information Available

^1^H and ^13^C NMR spectroscopic data, computational study details (PDF)
