# Supplementary material for: Base-Induced Sulfoxide-Sulfenate Rearrangement of 2-Sulfinyl Dienes for the Regio- and Stereoselective Synthesis of Enantioenriched Dienyl Diols
Source: J Org Chem. 2023 Mar 3;88(6):3697–713. doi: 10.1021/acs.joc.2c02931 (PMC10028699; doi:10.1021/acs.joc.2c02931)
Supplement: Supplementary file 1 — jo2c02931_si_001.pdf [file jo2c02931_si_001.pdf]

Supporting Information for

Base-Induced Sulfoxide-Sulfenate Rearrangement of 2-Sulfinyl Dienes for the Regio- and Stereoselective Synthesis of Enantioenriched Dienyl Diols

Marina Velado,[a] Manuel Martinović,[a] Inés Alonso,[b,c] Mariola Tortosa,[b,c] Roberto Fernández de la Pradilla,[a] Alma Viso.[a*]

[a]Instituto de Química Orgánica General (IQOG), CSIC, Juan de la Cierva 3, 28006 Madrid, Spain.

[b]Organic Chemistry Department and Center for Innovation in Advanced Chemistry (ORFEO-CINQA) Universidad Autónoma de Madrid (UAM), 28049 Madrid, Spain.

[c]Institute for Advanced Research in Chemical Sciences (IAdChem). Universidad Autónoma de Madrid (UAM), 28049 Madrid, Spain.

e-mail: almaviso@iqog.csic.es

Table of contents

1. NMR data……………………………………………………………………………………………………….S3

2. Theoretical caculations………………………….………………………………………………………S86

1. NMR data

^{1}H NMR ($CDCl_3$, 400 MHz)

(*S*)-*tert*-Butyldiphenyl((6-(*p*-tolylsulfinyl)hex-5-yn-1-yl)oxy)silane.

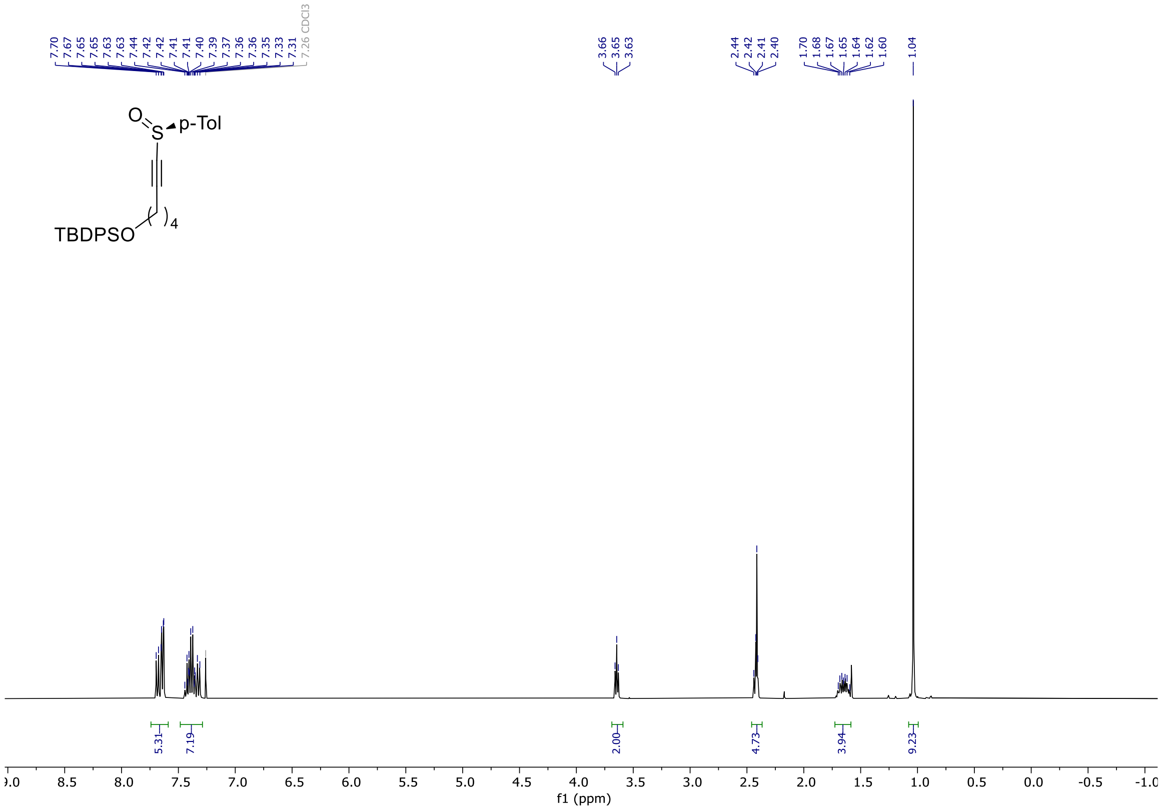

^{13}C NMR ($CDCl_3$, 100 MHz)

(*S*)-*tert*-Butyldiphenyl((6-(*p*-tolylsulfinyl)hex-5-yn-1-yl)oxy)silane.

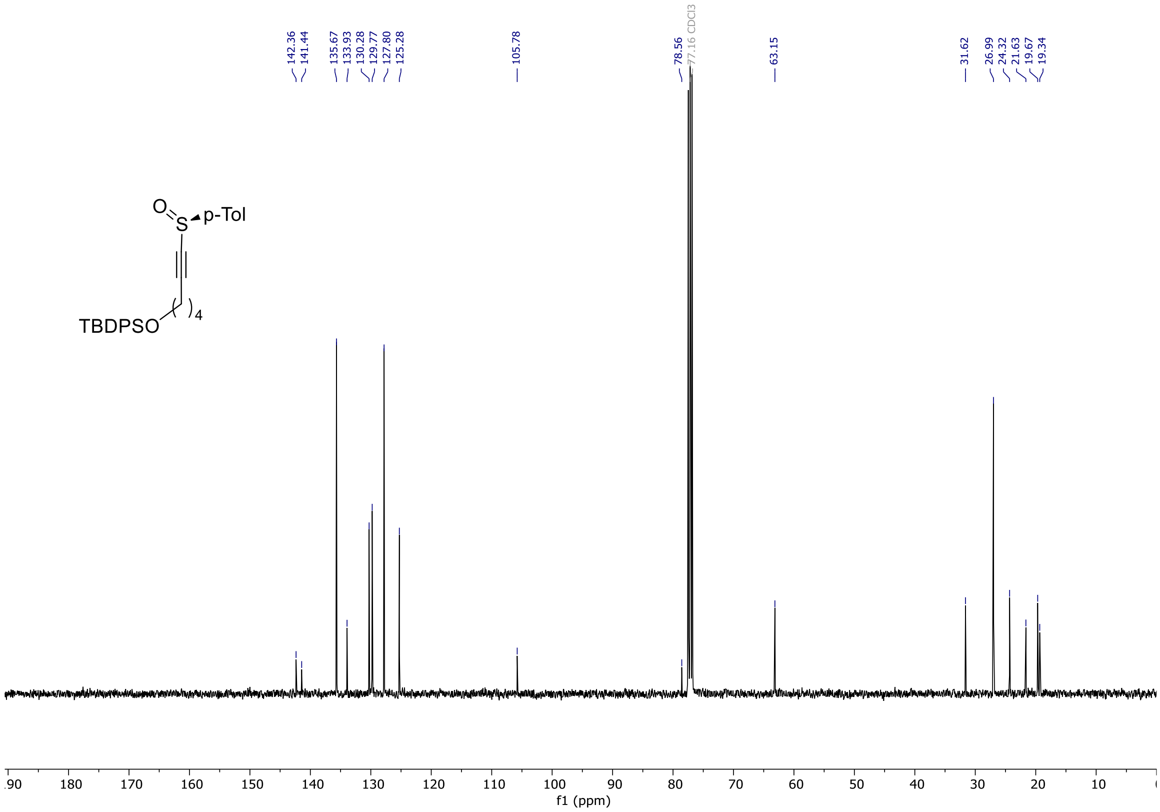

^{1}H NMR ($CDCl_3$, 400 MHz)

(*S*,*E*)-*tert*-Butyldiphenyl((6-(*p*-tolylsulfinyl)-6-(tributylstannyl)hex-5-en-1-yl)oxy)silane.

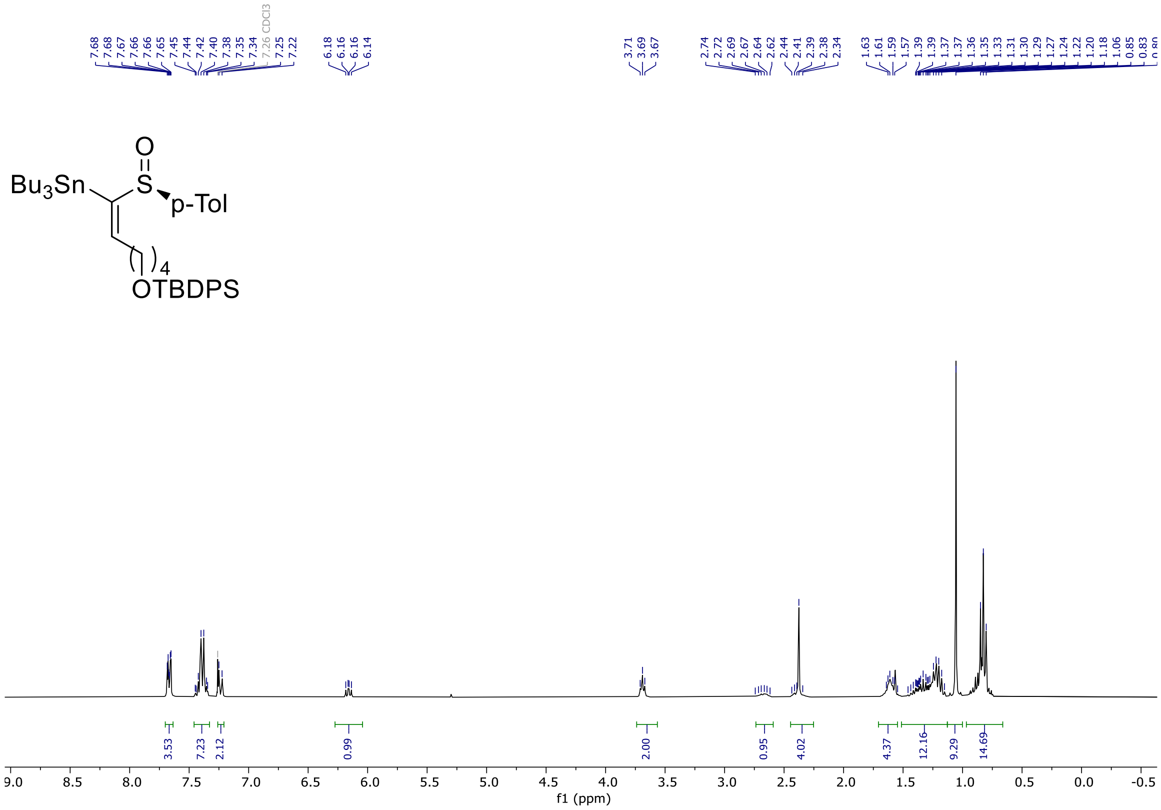

^{13}C NMR ($CDCl_3$, 100 MHz)

(*S*,*E*)-*tert*-Butyldiphenyl((6-(*p*-tolylsulfinyl)-6-(tributylstannyl)hex-5-en-1-yl)oxy)silane.

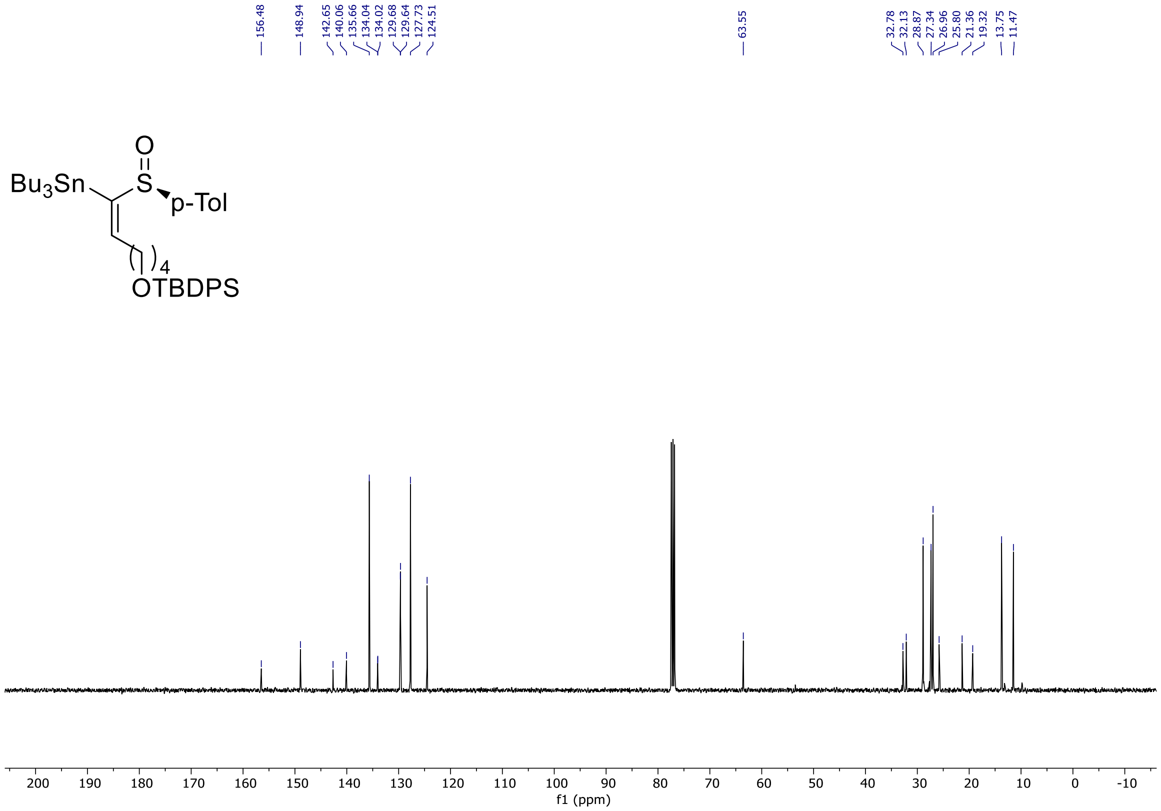

^{1}H NMR ($CDCl_3$, 400 MHz)

(*S*,*E*)-*tert*-Butyl((6-iodo-6-(*p*-tolylsulfinyl)hex-5-en-1-yl)oxy)diphenylsilane.

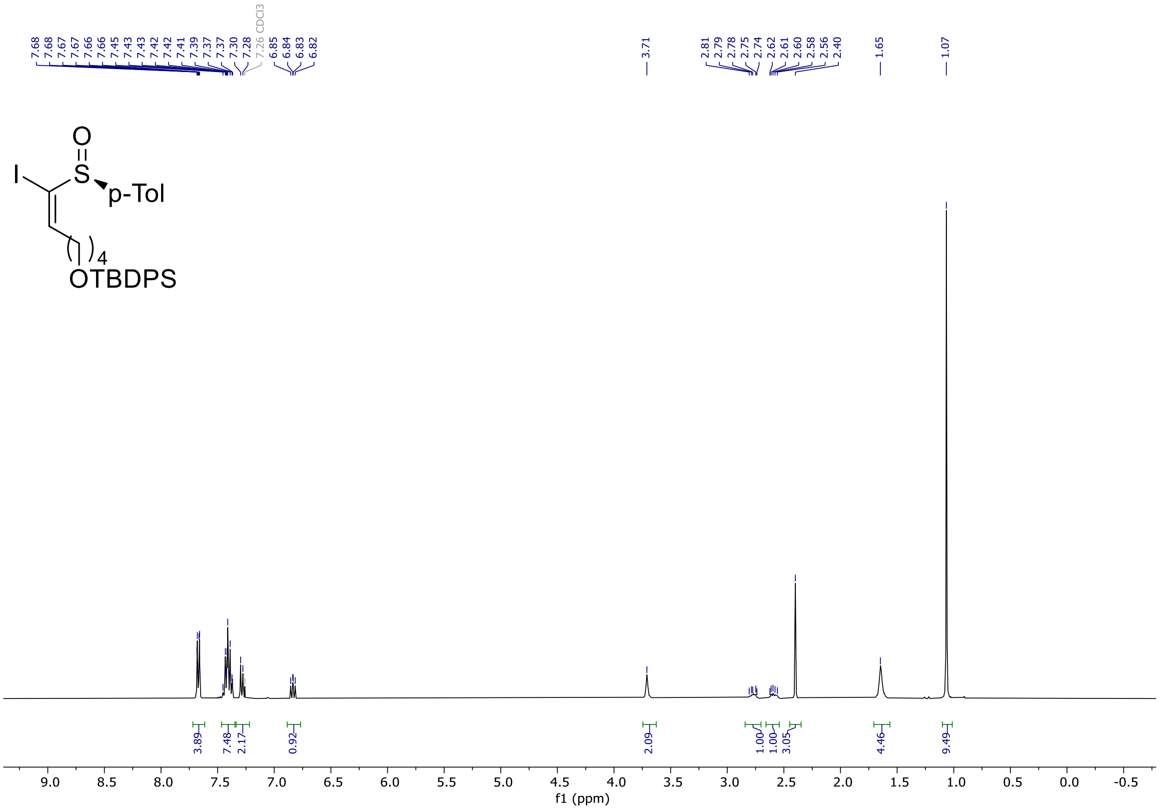

^{13}C NMR ($CDCl_3$, 100 MHz)

(*S*,*E*)-*tert*-Butyl((6-iodo-6-(*p*-tolylsulfinyl)hex-5-en-1-yl)oxy)diphenylsilane.

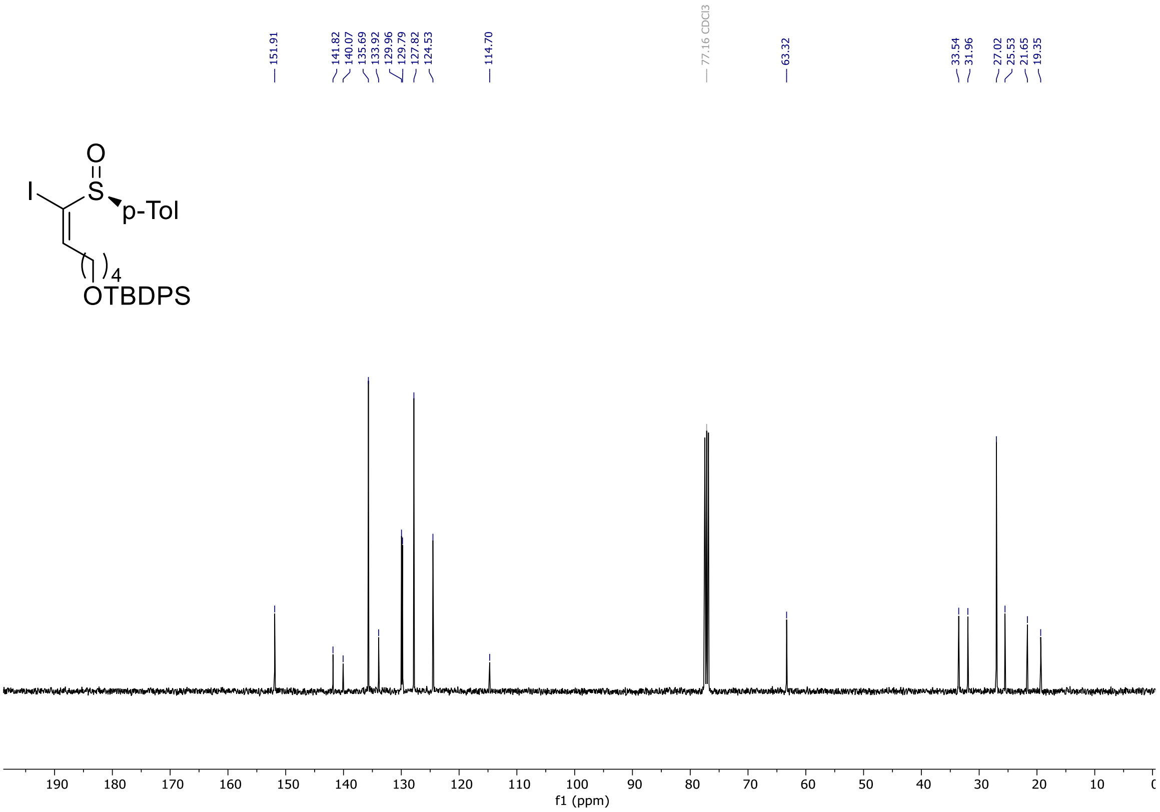

^{1}H NMR ($CDCl_3$, 400 MHz)

(*E*)-tributyl(2-cyclohexyl-1-(*p*-tolylsulfinyl)vinyl)stannane

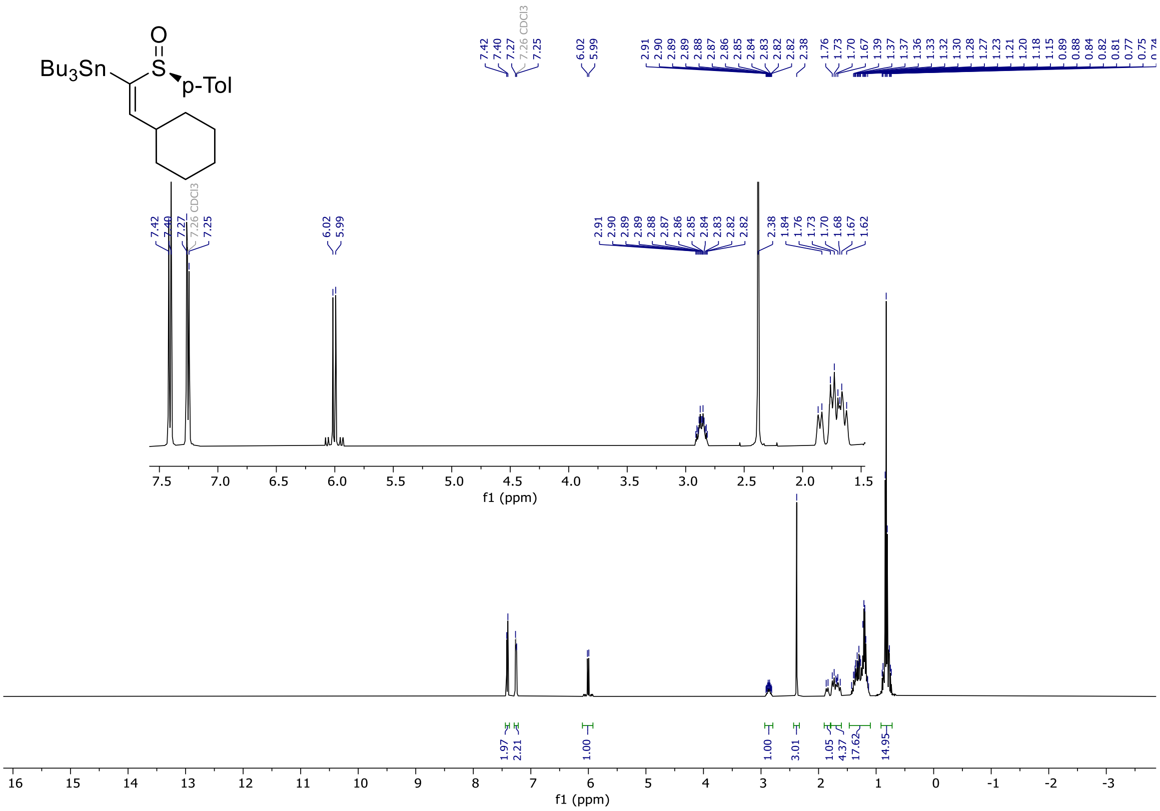

^{13}C NMR ($CDCl_3$, 100 MHz)

(*E*)-tributyl(2-cyclohexyl-1-(p-tolylsulfinyl)vinyl)stannane

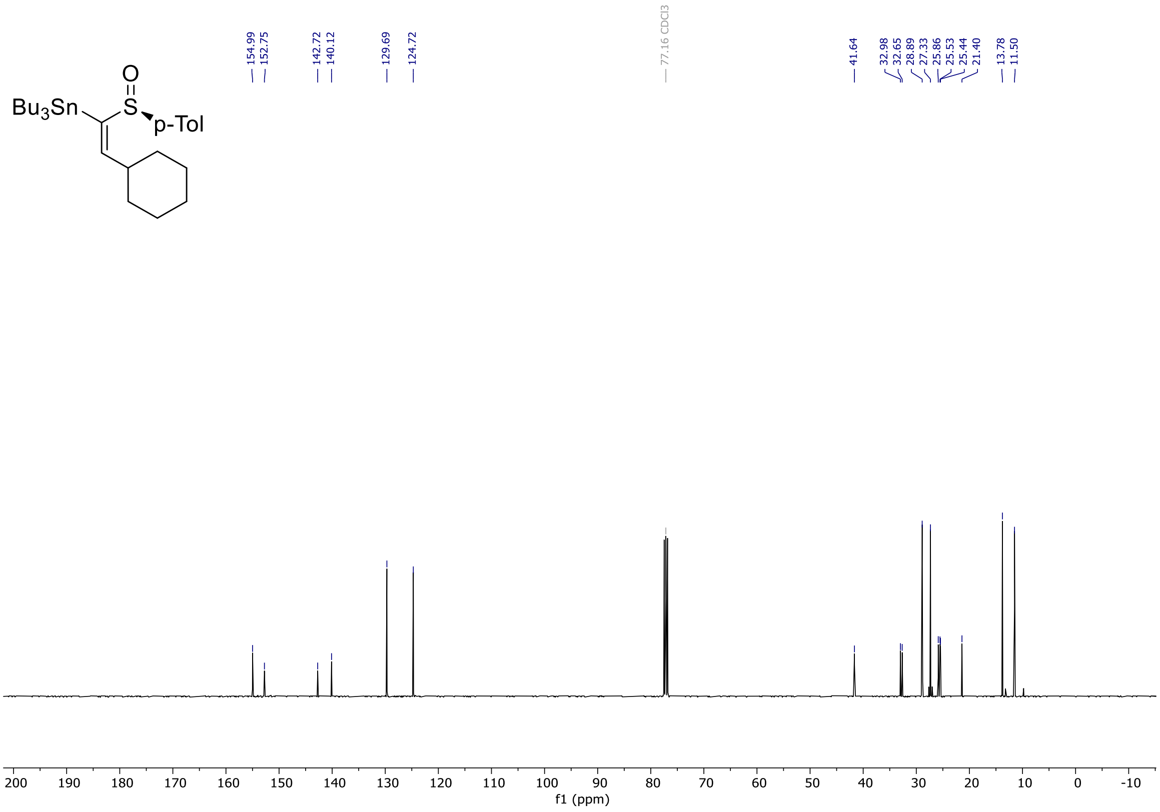

^{1}H NMR (acetone-d_6, 400 MHz)

1-(((1*Z*,3*E*)-1-cyclohexyl-4-iodobuta-1,3-dien-2-yl)sulfinyl)-4-methylbenzene

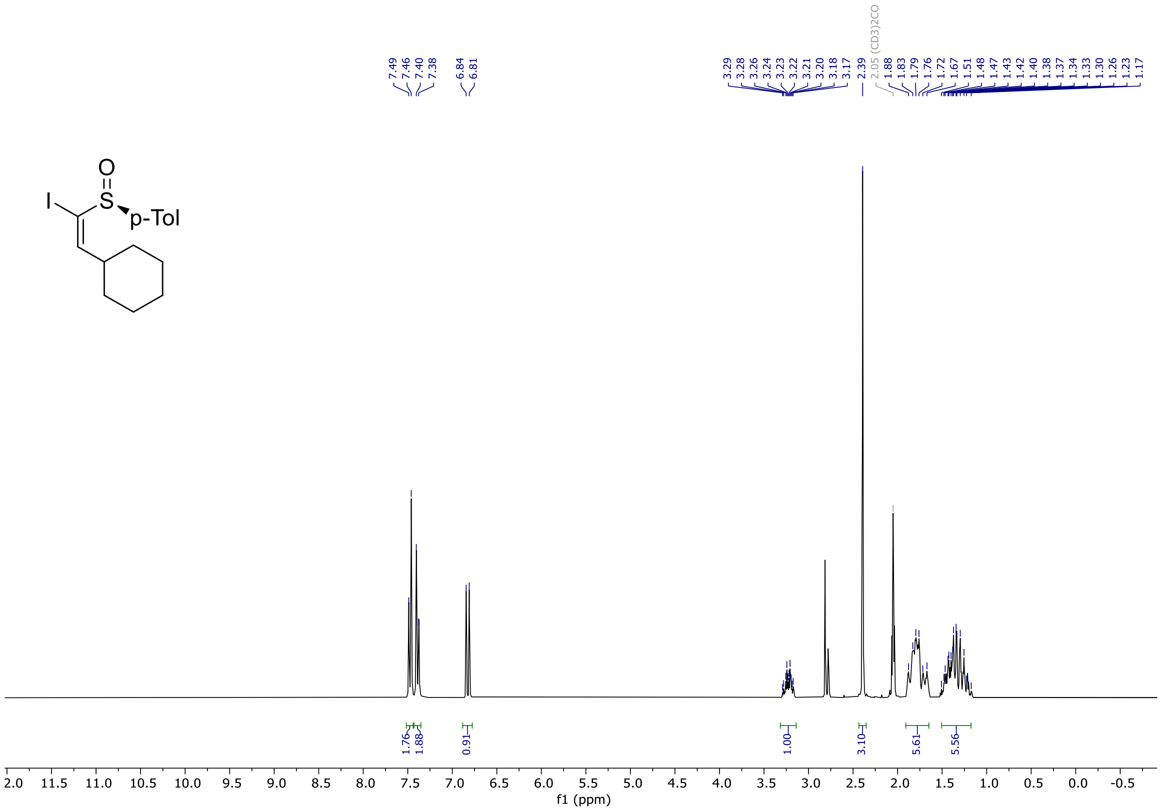

^{13}C NMR (acetone-d_6, 100 MHz)

1-(((1*Z*,3*E*)-1-cyclohexyl-4-iodobuta-1,3-dien-2-yl)sulfinyl)-4-methylbenzene

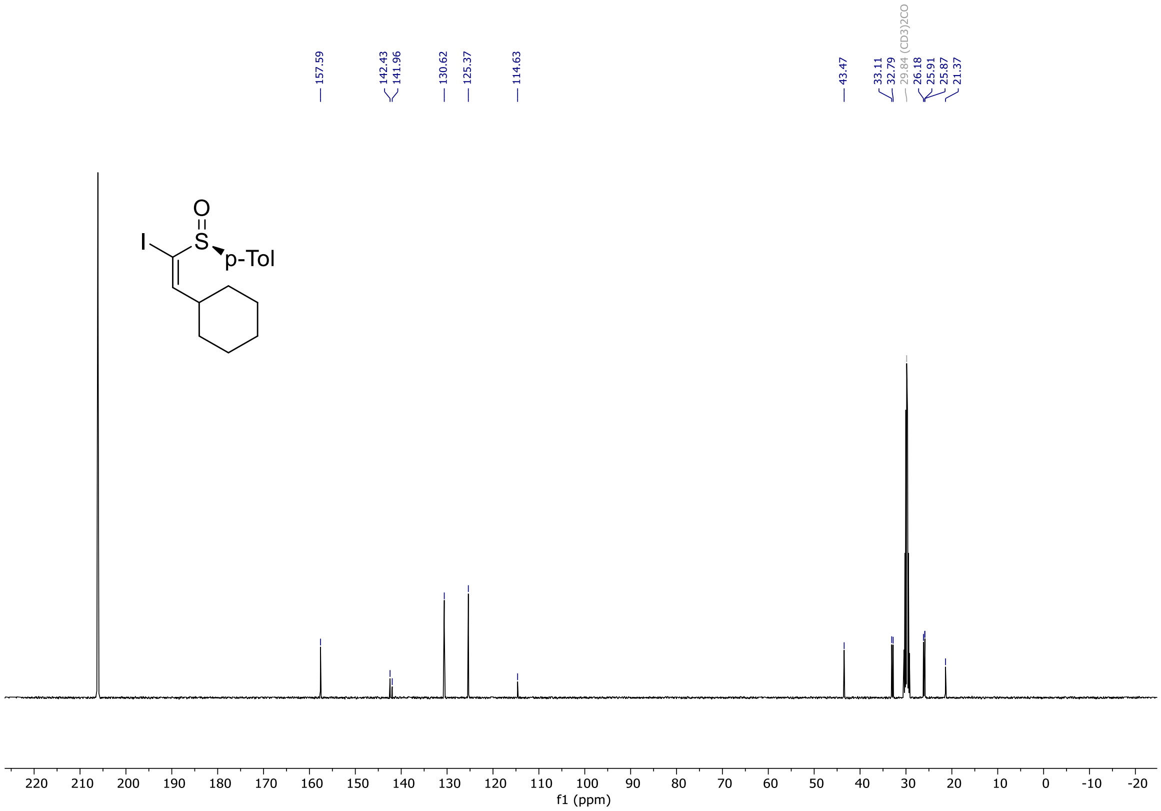

^{1}H NMR (CDCl$_3$, 400 MHz) (*E*,*Z*)-**1c**

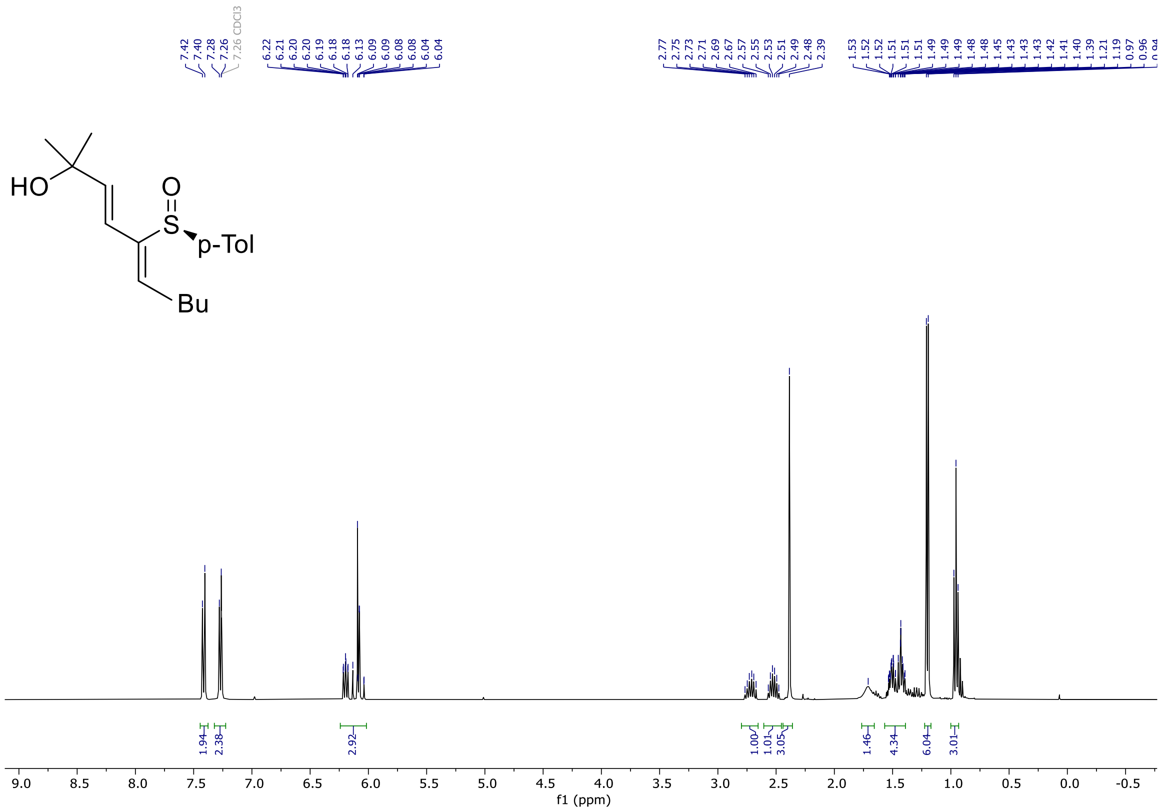

^{13}C NMR (CDCl$_3$, 100 MHz) (*E*,*Z*)-**1c**

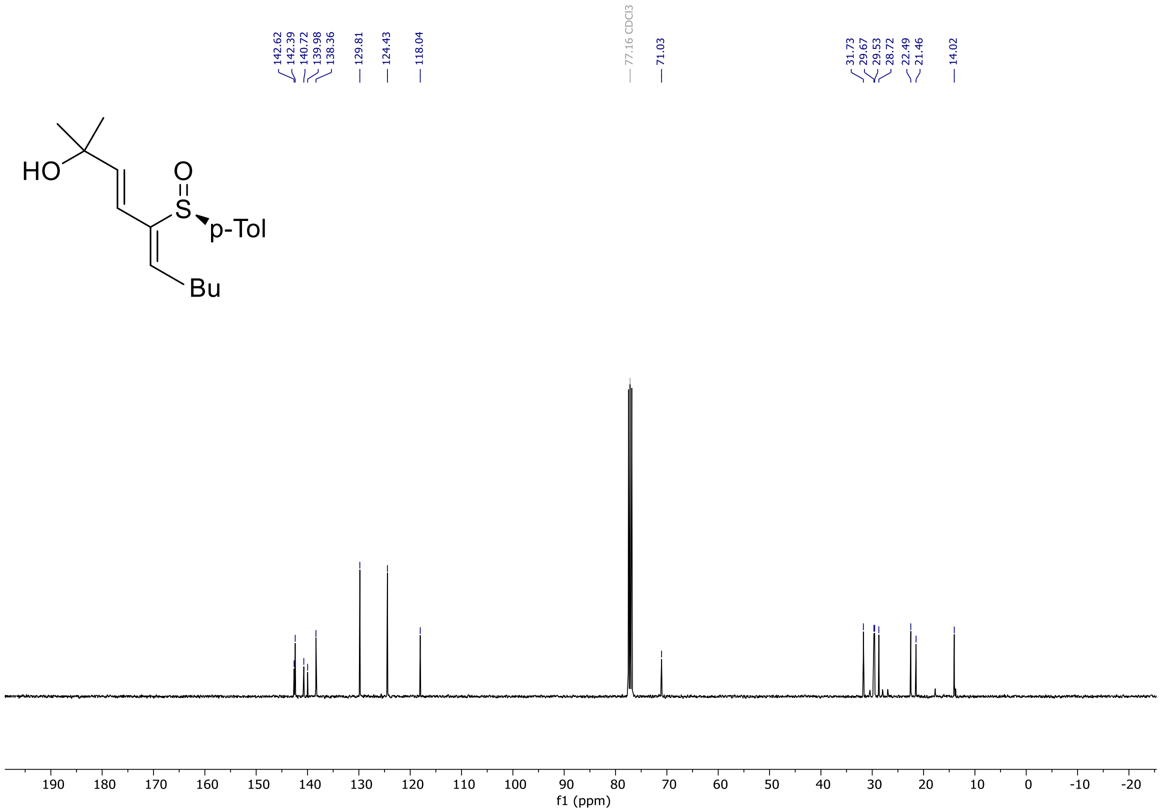

COSY (^{1}H, ^{1}H) (*E*,*Z*)-**1c**

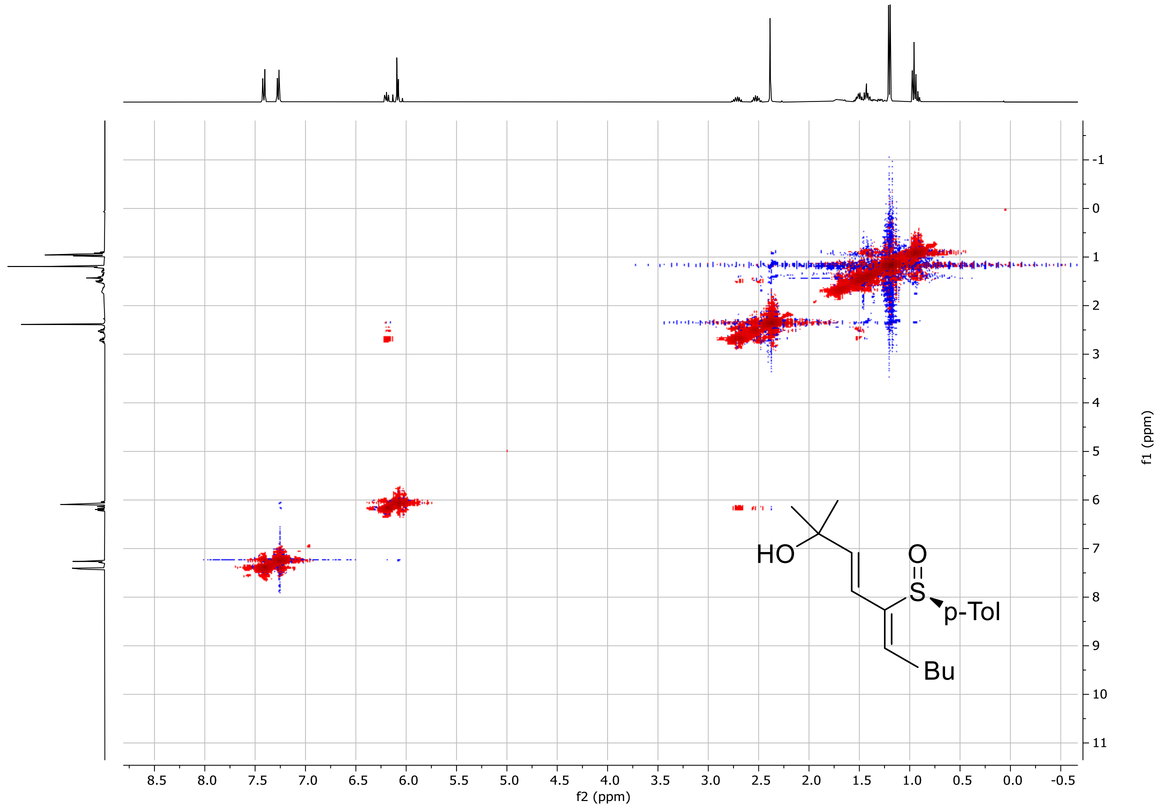

HSQC (^{1}H, ^{13}C) (*E*,*Z*)-**1c**

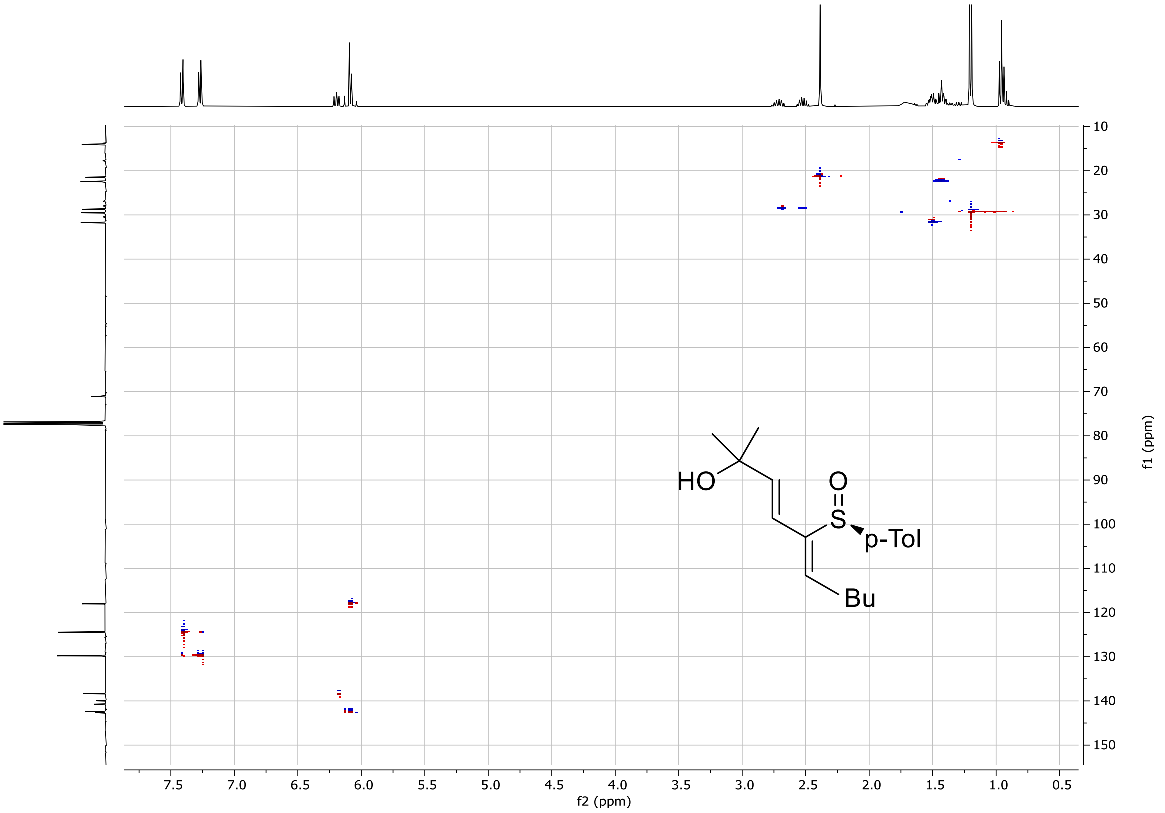

^{1}H NMR ($CDCl_3$, 400 MHz) (*E*,*E*)-**1d**

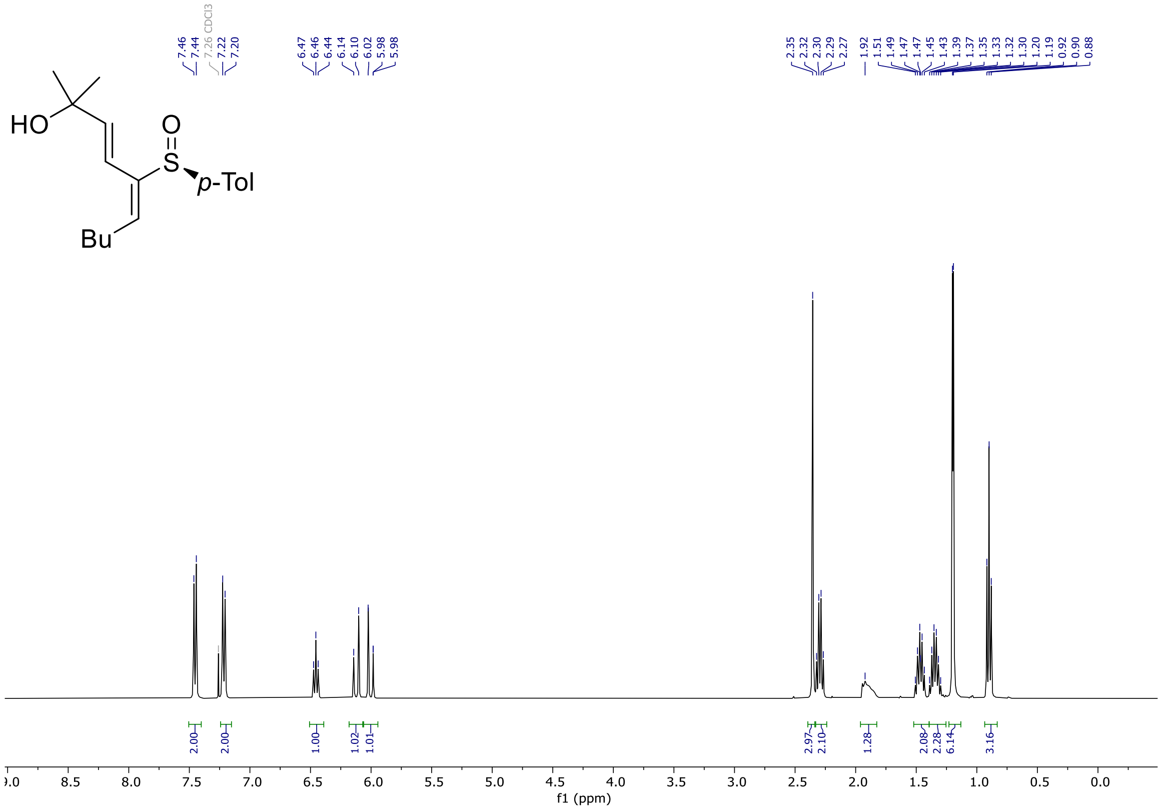

^{13}C NMR ($CDCl_3$, 100 MHz) (*E*,*E*)-**1d**

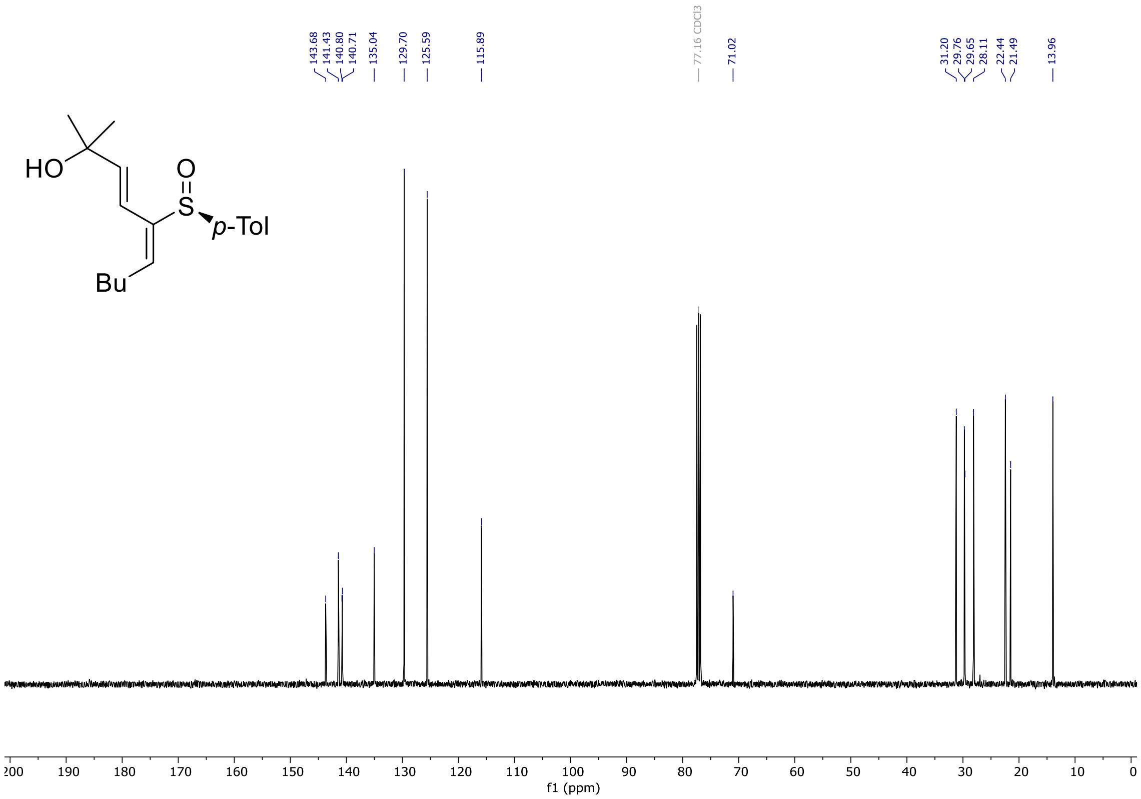

^{1}H NMR ($CDCl_3$, 400 MHz) (*E*,*Z*)-**1e**

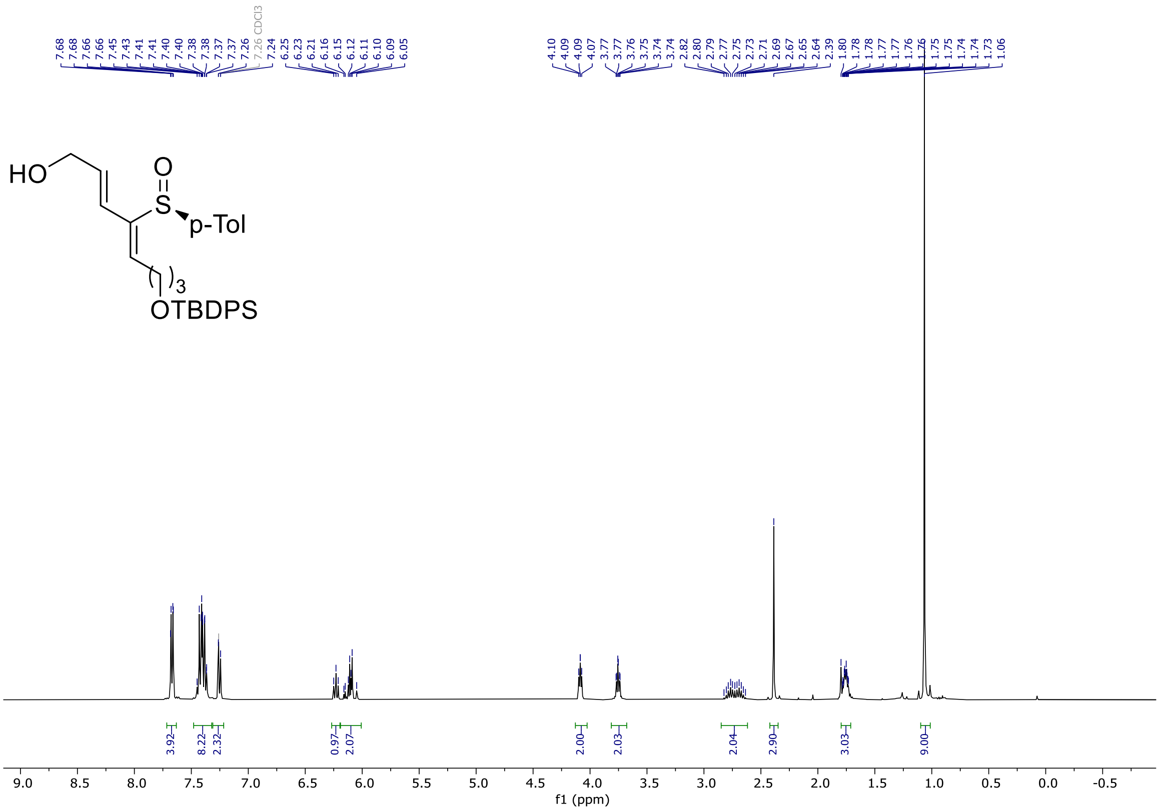

^{13}C NMR ($CDCl_3$, 100 MHz) (*E*,*Z*)-**1e**

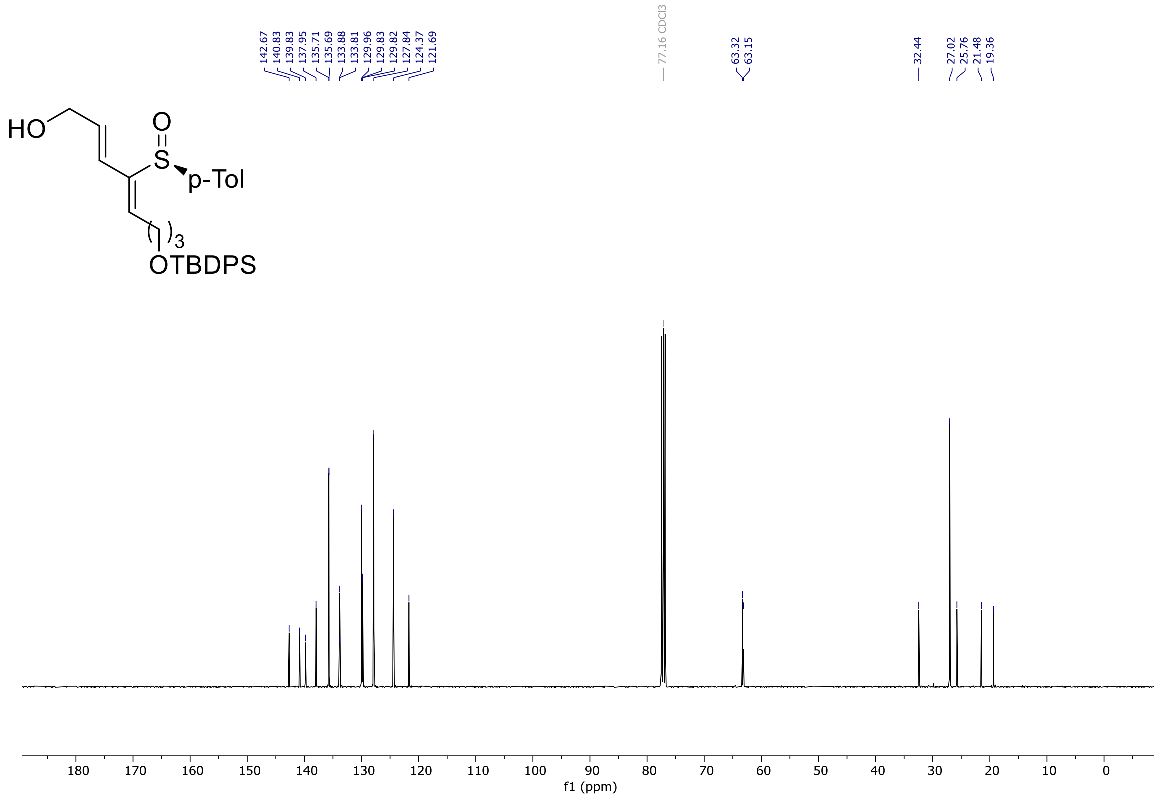

^{1}H NMR ($CDCl_3$, 400 MHz) (*E*,*Z*)-**1f**

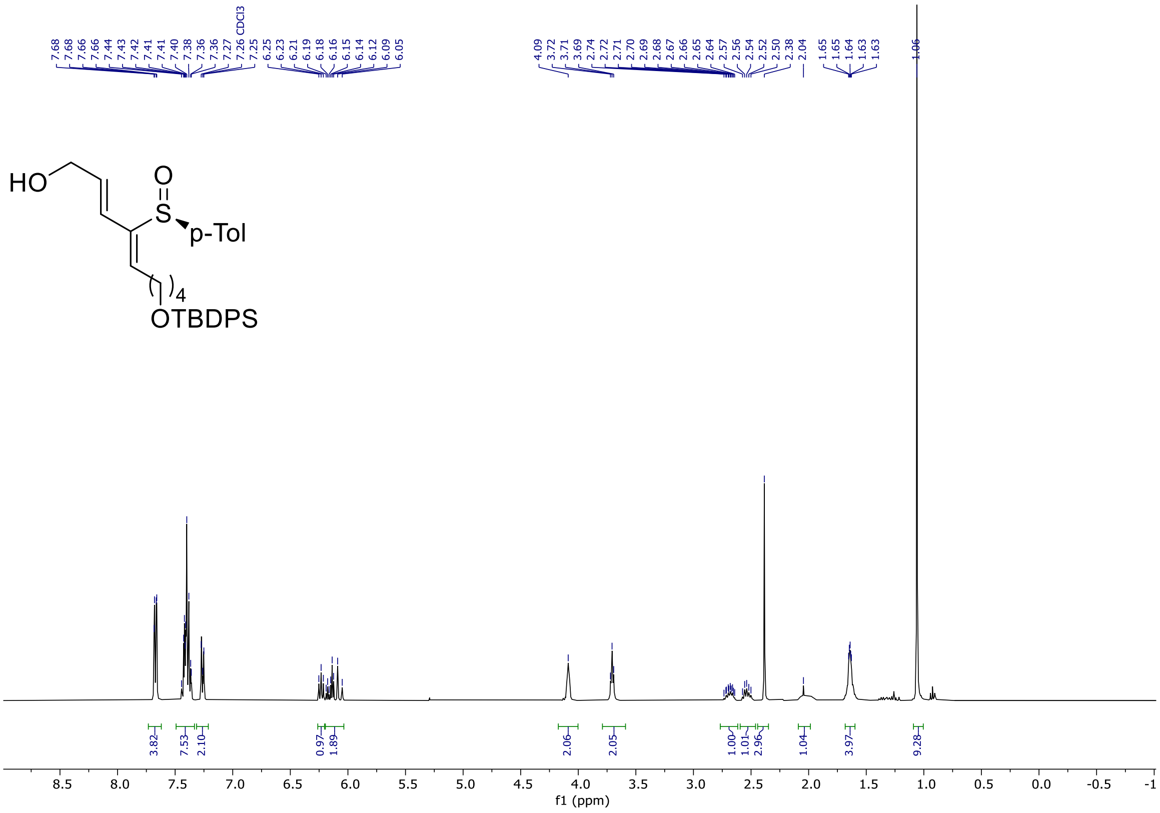

^{13}C NMR ($CDCl_3$, 100 MHz) (*E*,*Z*)-**1f**

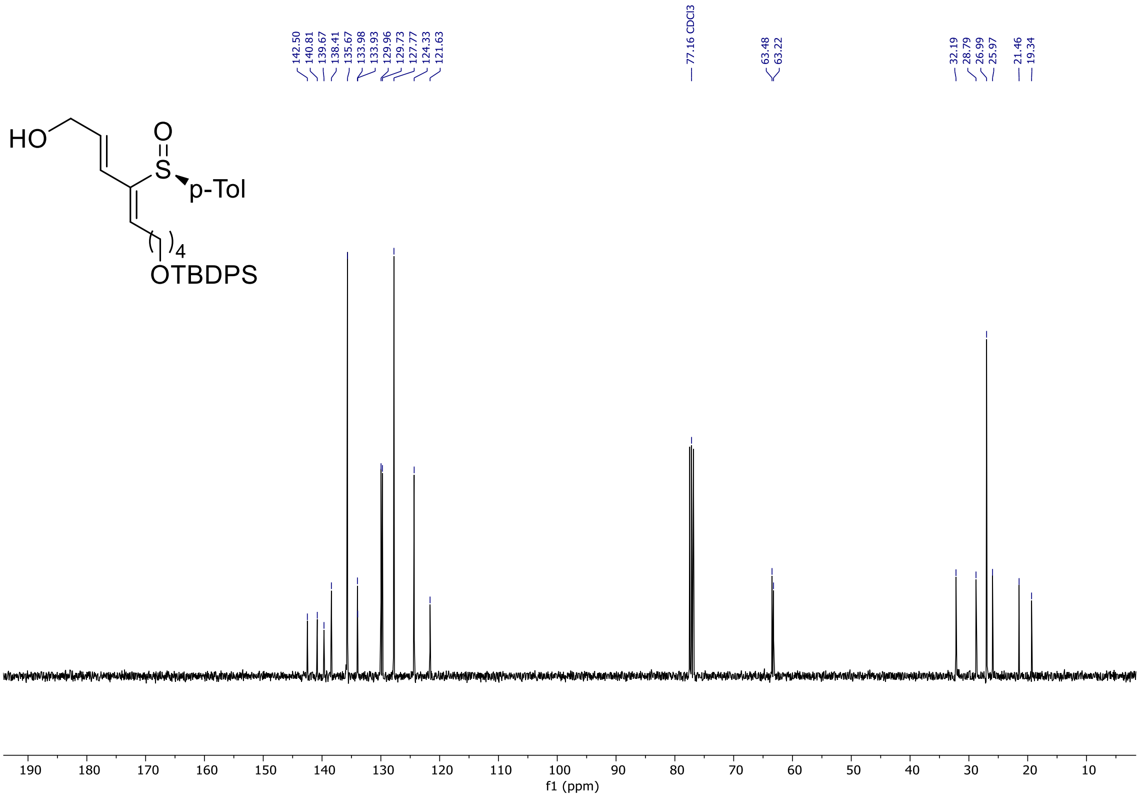

^{1}H NMR ($CDCl_3$, 400 MHz) (*E*,*Z*)-**1g**

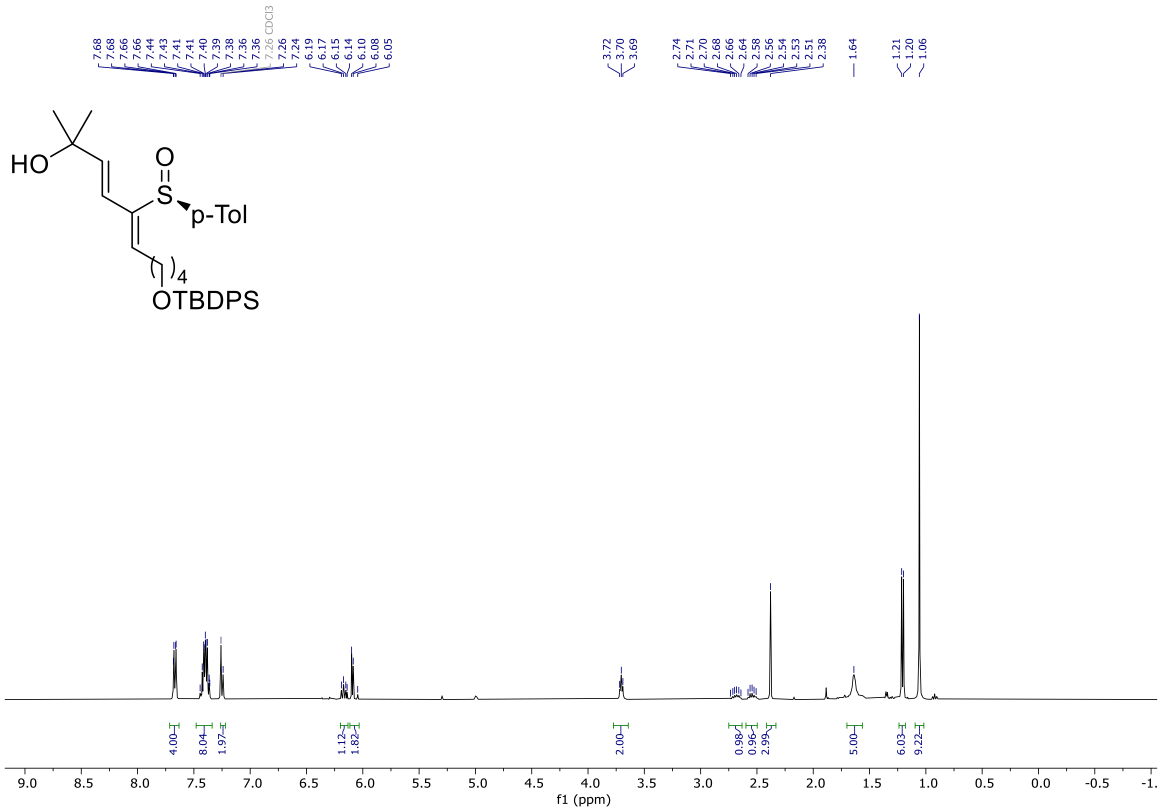

^{13}C NMR ($CDCl_3$, 100 MHz) (*E*,*Z*)-**1g**

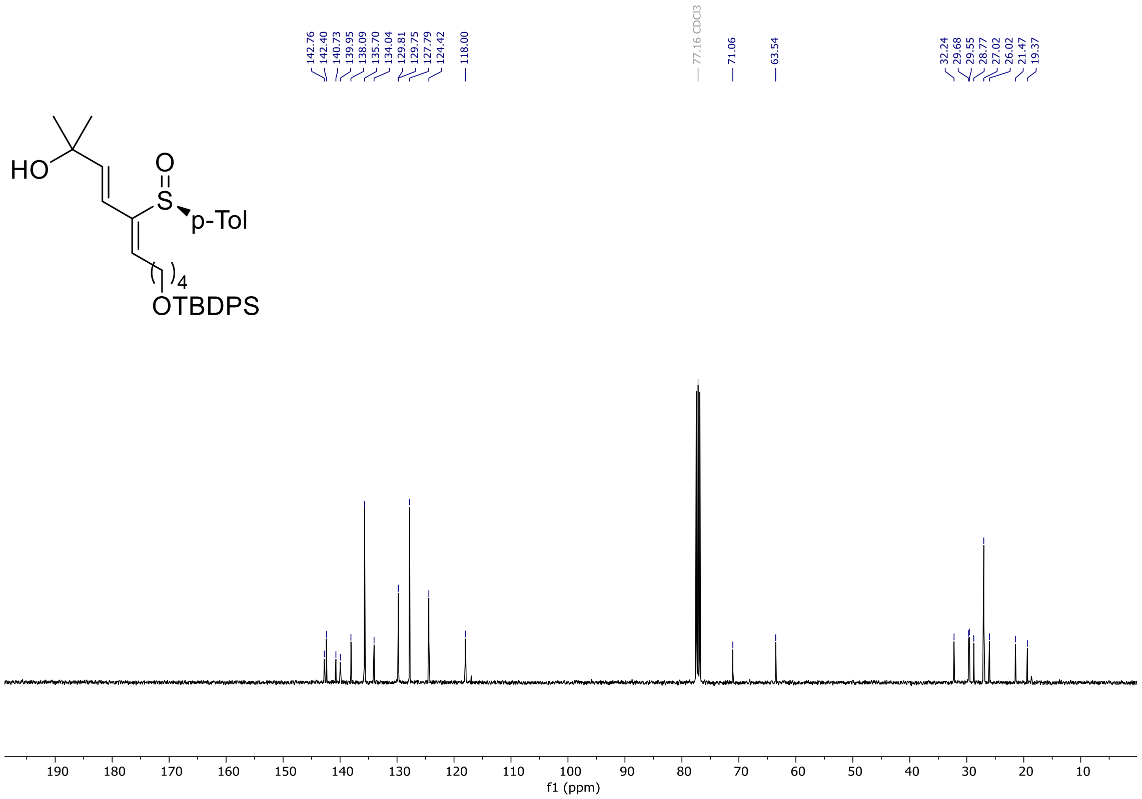

^{1}H NMR ($CDCl_3$, 300 MHz) (*E*,*Z*)-**1i**

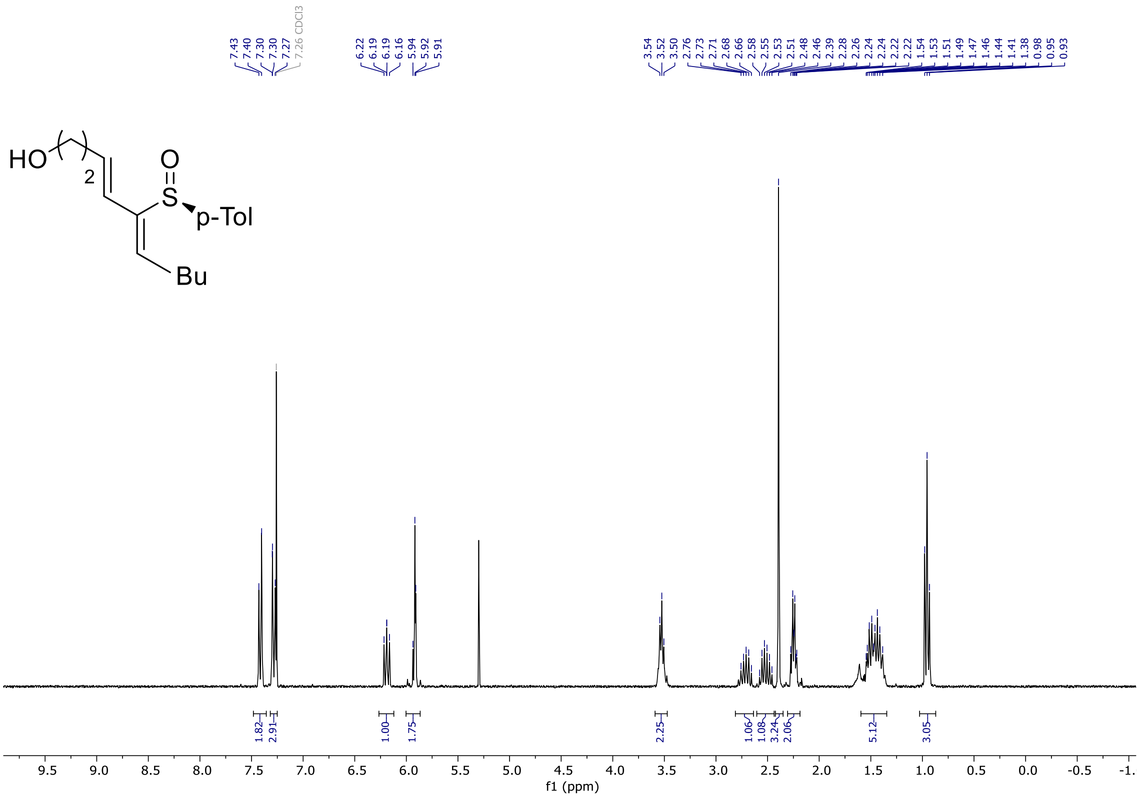

^{13}C NMR ($CDCl_3$, 75 MHz) (*E*,*Z*)-**1i**

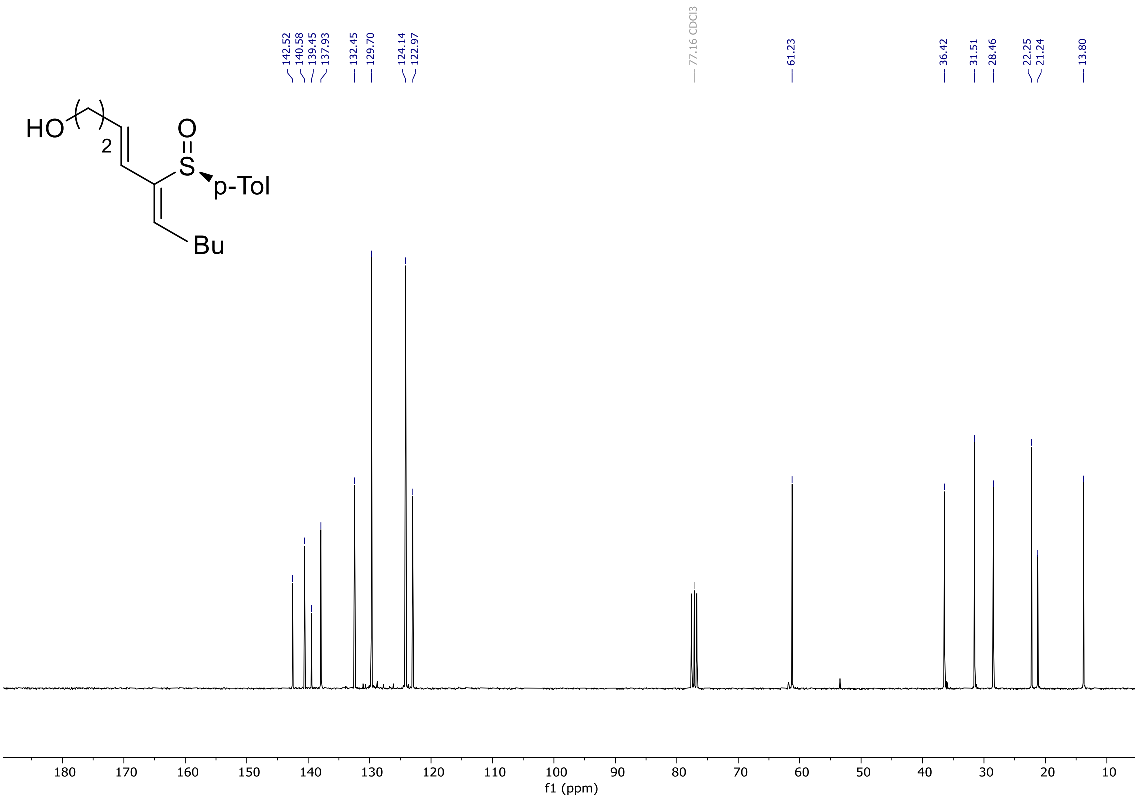

^{1}H NMR ($CDCl_3$, 400 MHz) (*E*,*Z*)-**1j**

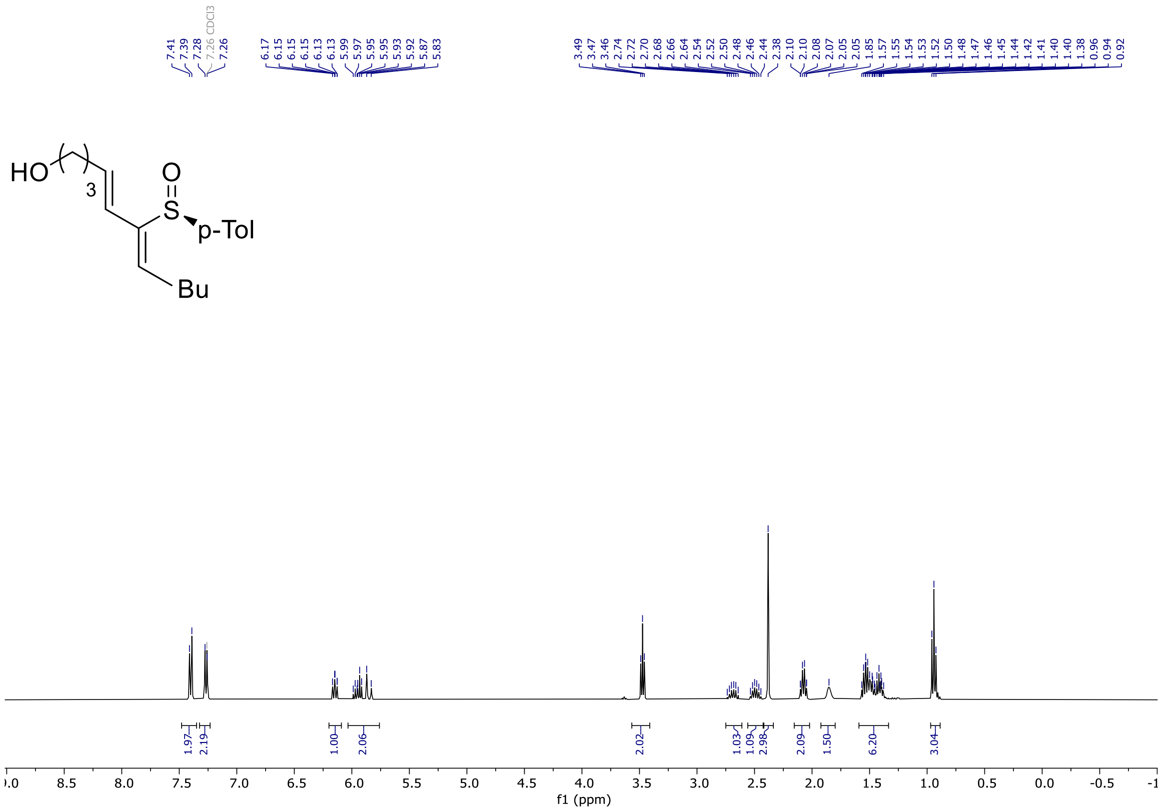

^{13}C NMR ($CDCl_3$, 100 MHz) (*E*,*Z*)-**1j**

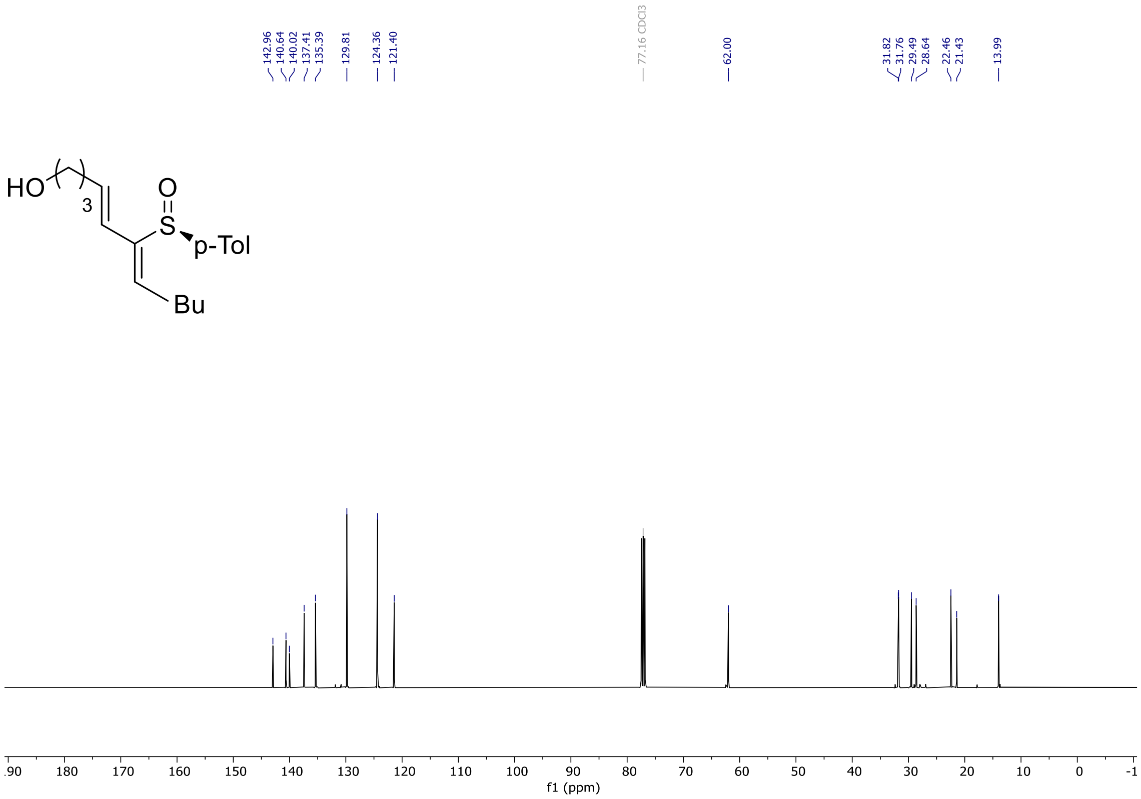

^{1}H NMR ($CDCl_3$, 400 MHz) (*E*,*Z*)-**1l**

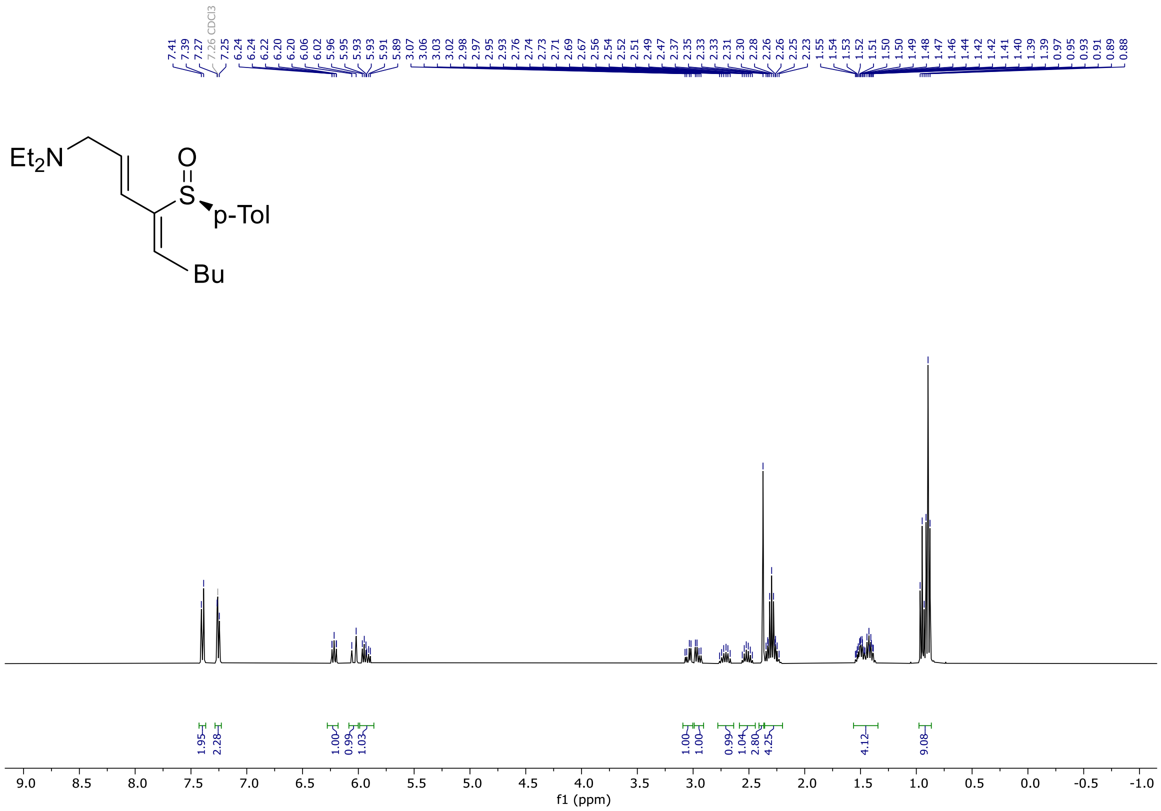

^{13}C NMR ($CDCl_3$, 100 MHz) (*E*,*Z*)-**1l**

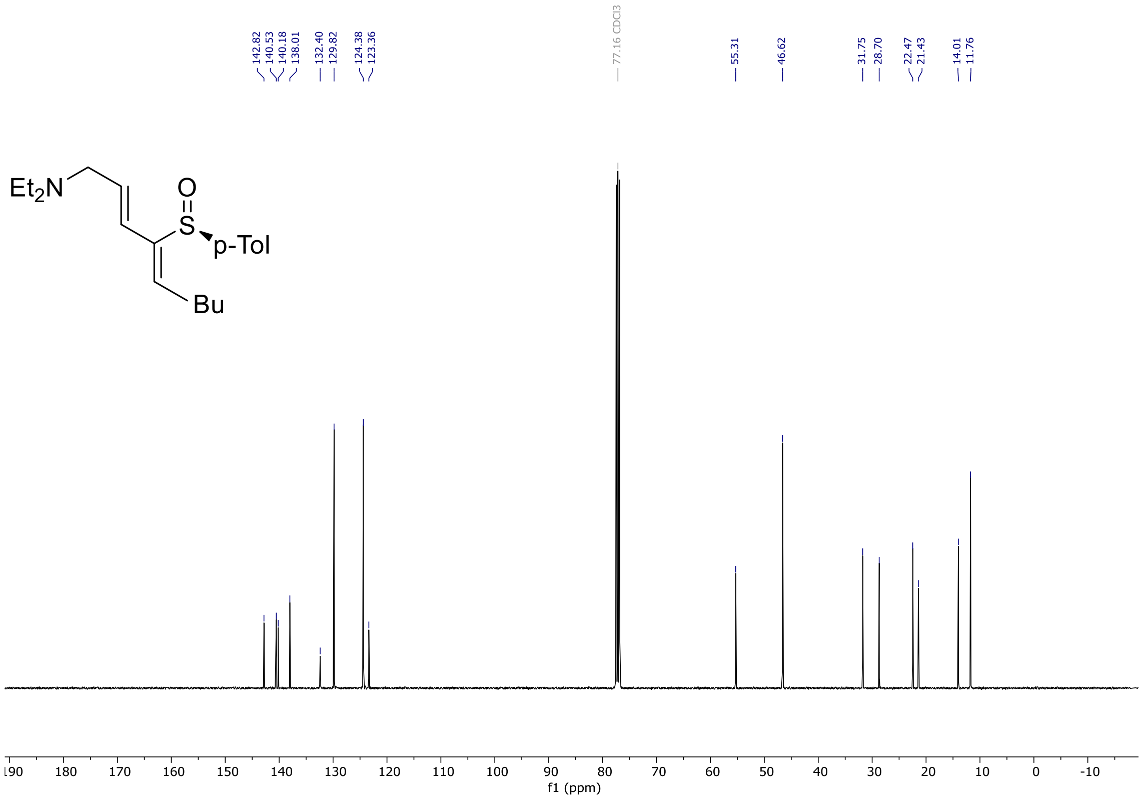

^{1}H NMR ($CDCl_3$, 400 MHz) (*E*,*Z*)-**1m**

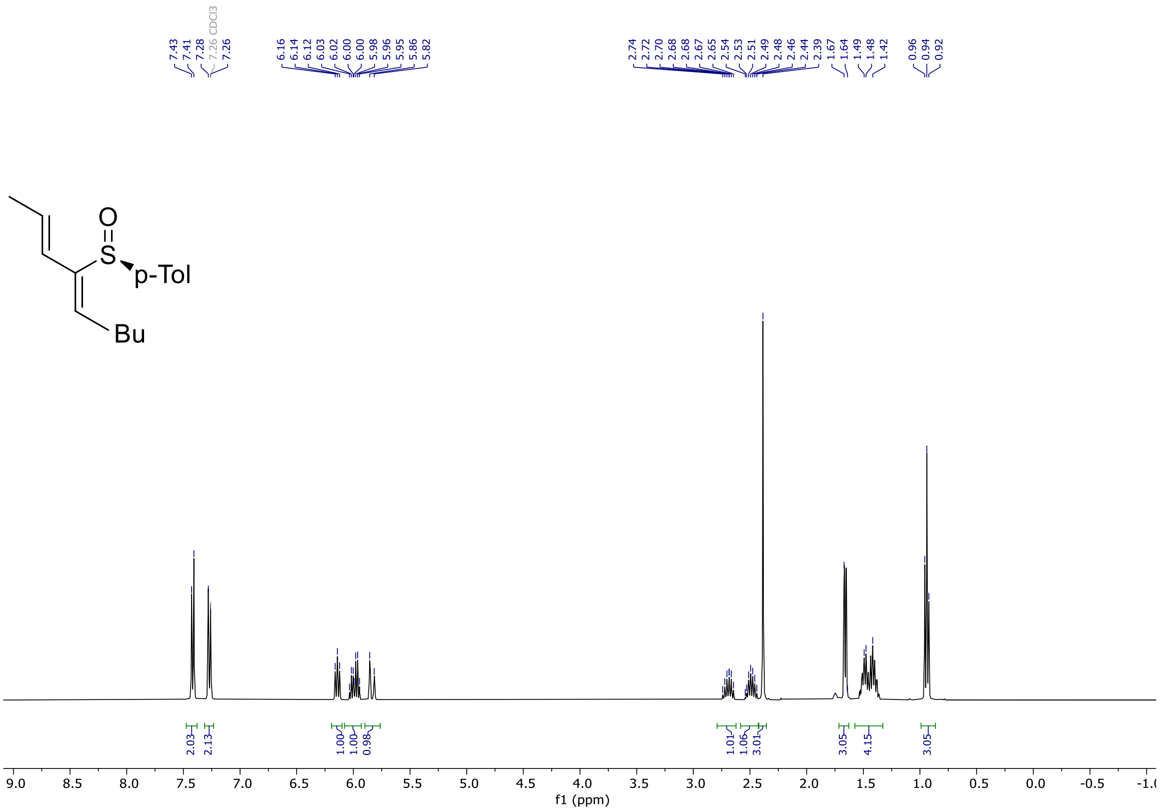

^{13}C NMR ($CDCl_3$, 100 MHz) (*E*,*Z*)-**1m**

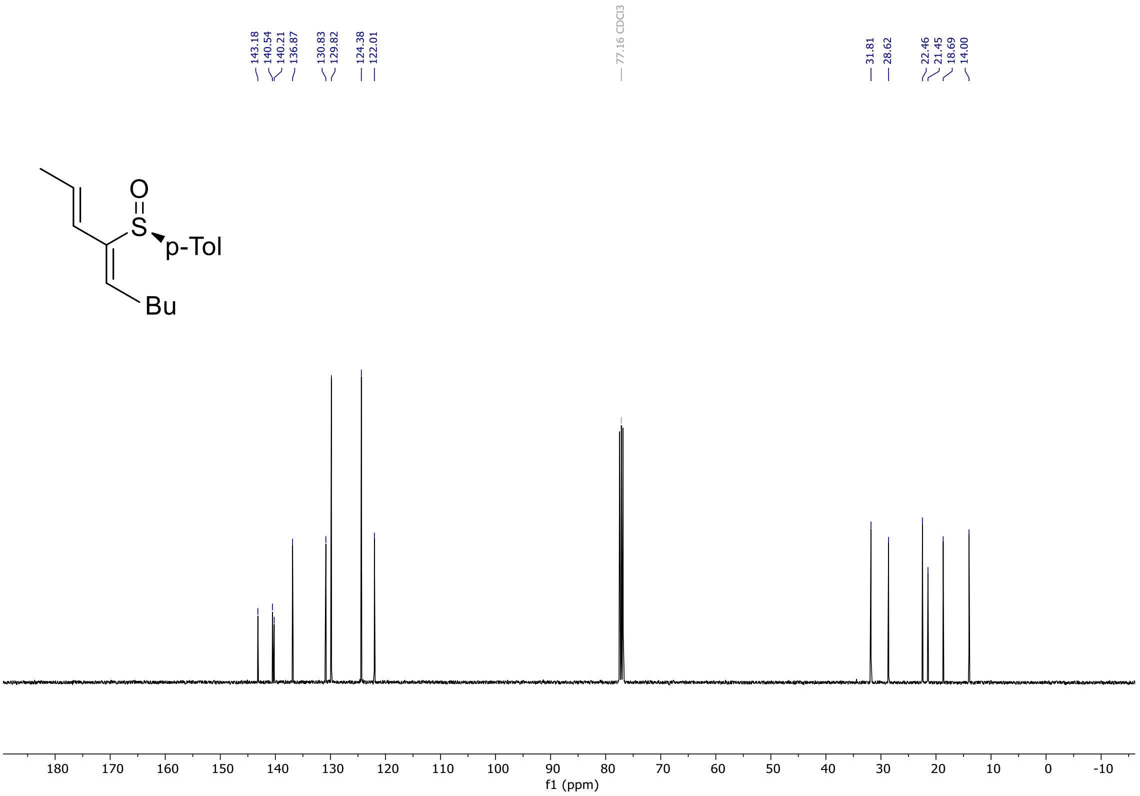

COSY (^{1}H, ^{1}H) (*E*,*Z*)-**1m**

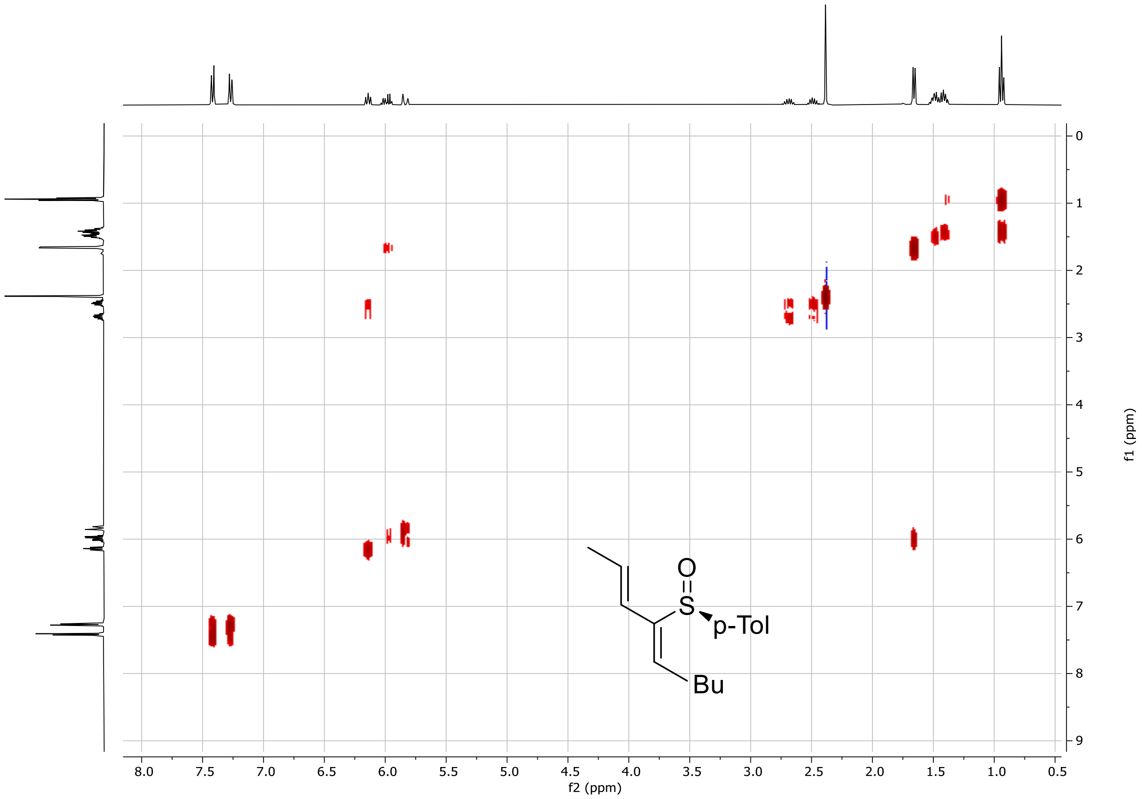

HSQC (^{1}H, ^{13}C) (*E*,*Z*)-**1m**

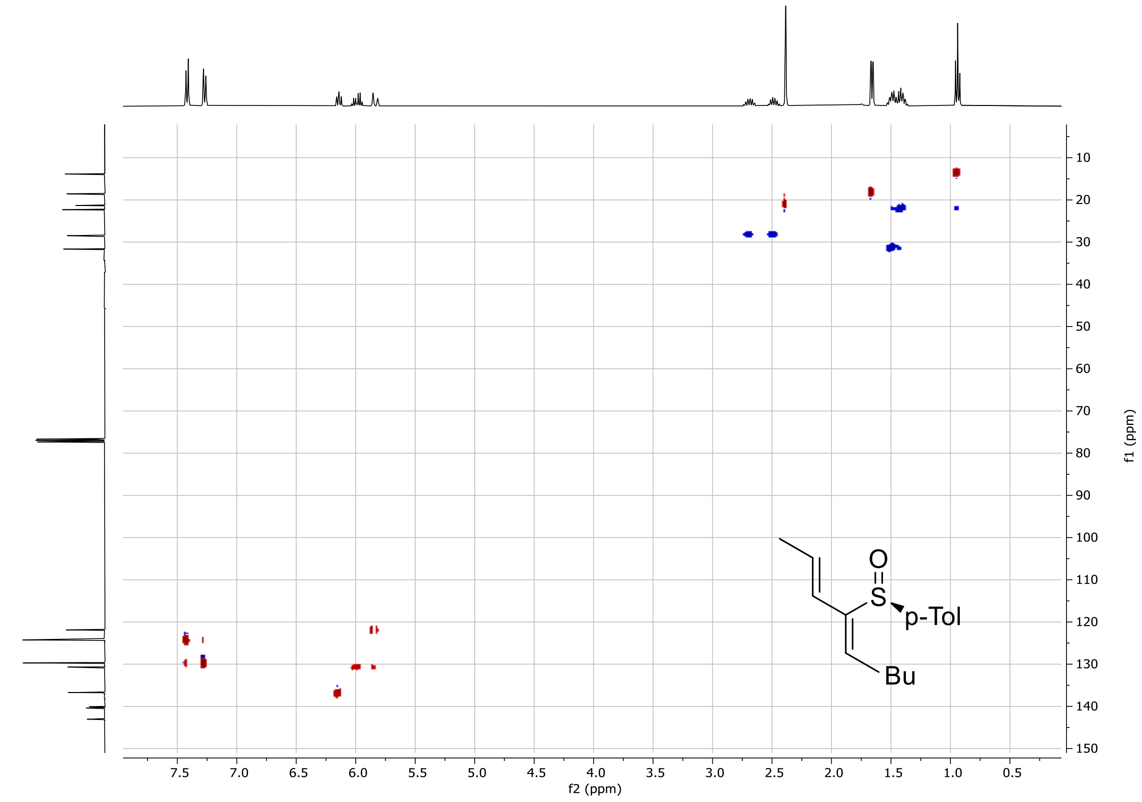

^{1}H NMR ($CDCl_3$, 400 MHz) (*Z*)-**1n**

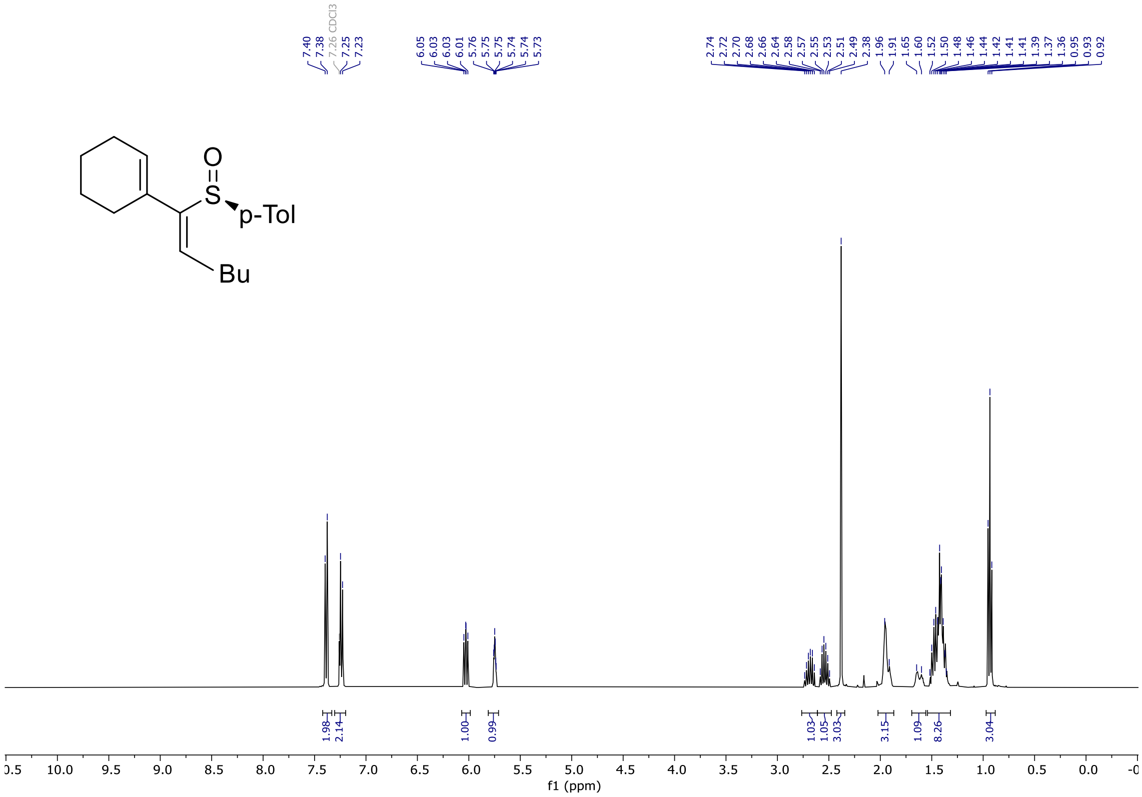

^{13}C NMR ($CDCl_3$, 100 MHz) (*Z*)-**1n**

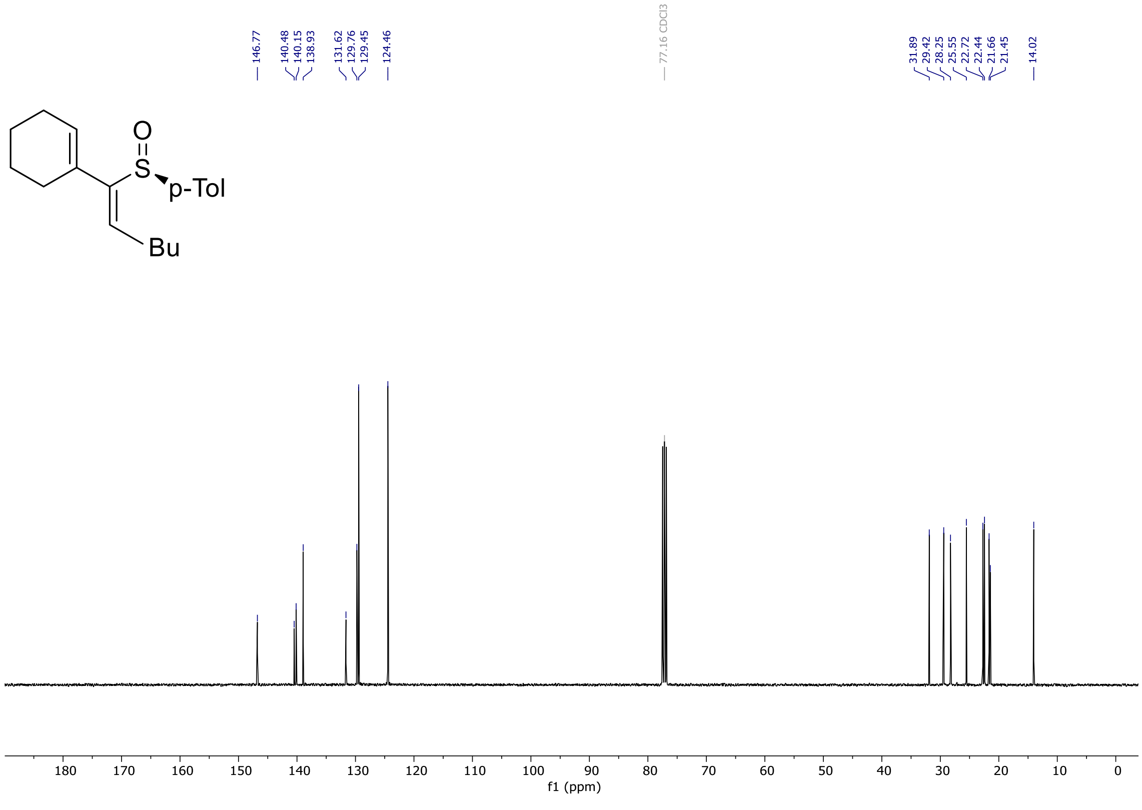

COSY (^{1}H, ^{1}H) (*E*,*Z*)-**1n**

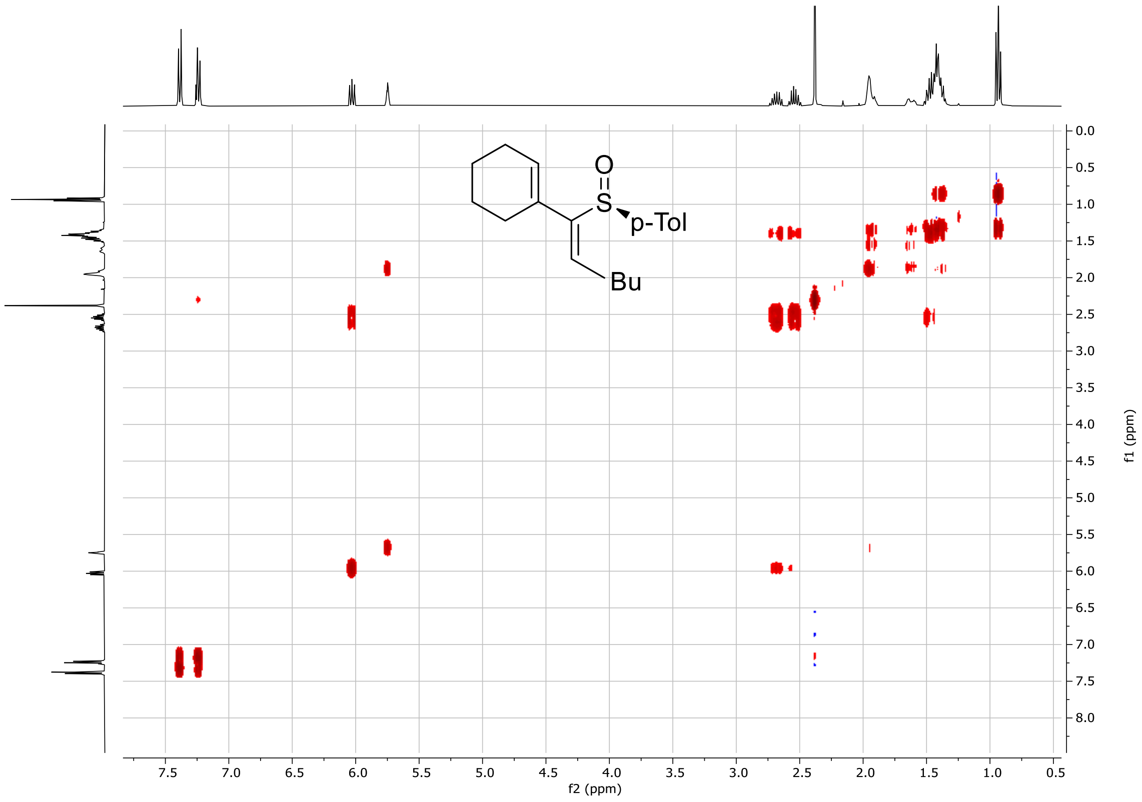

HSQC (^{1}H, ^{13}C) (*E*,*Z*)-**1n**

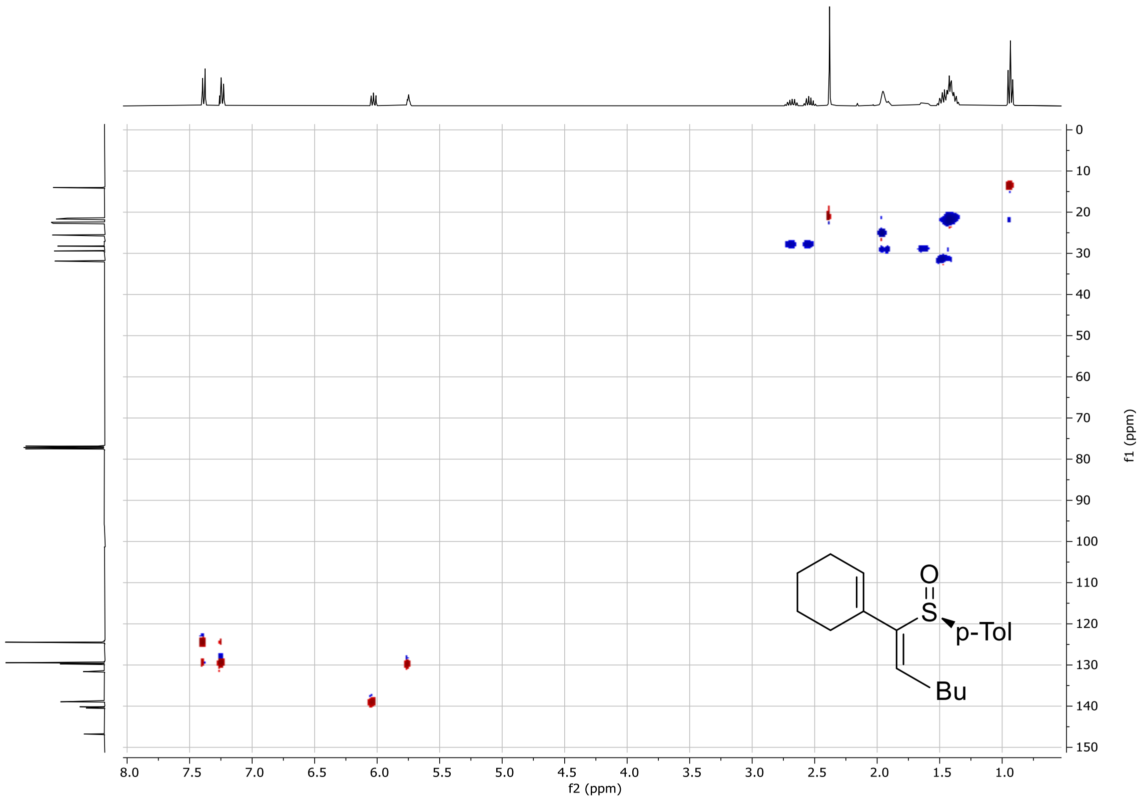

^{1}H NMR ($CDCl_3$, 500 MHz) (*E*,*Z*)-**1o**

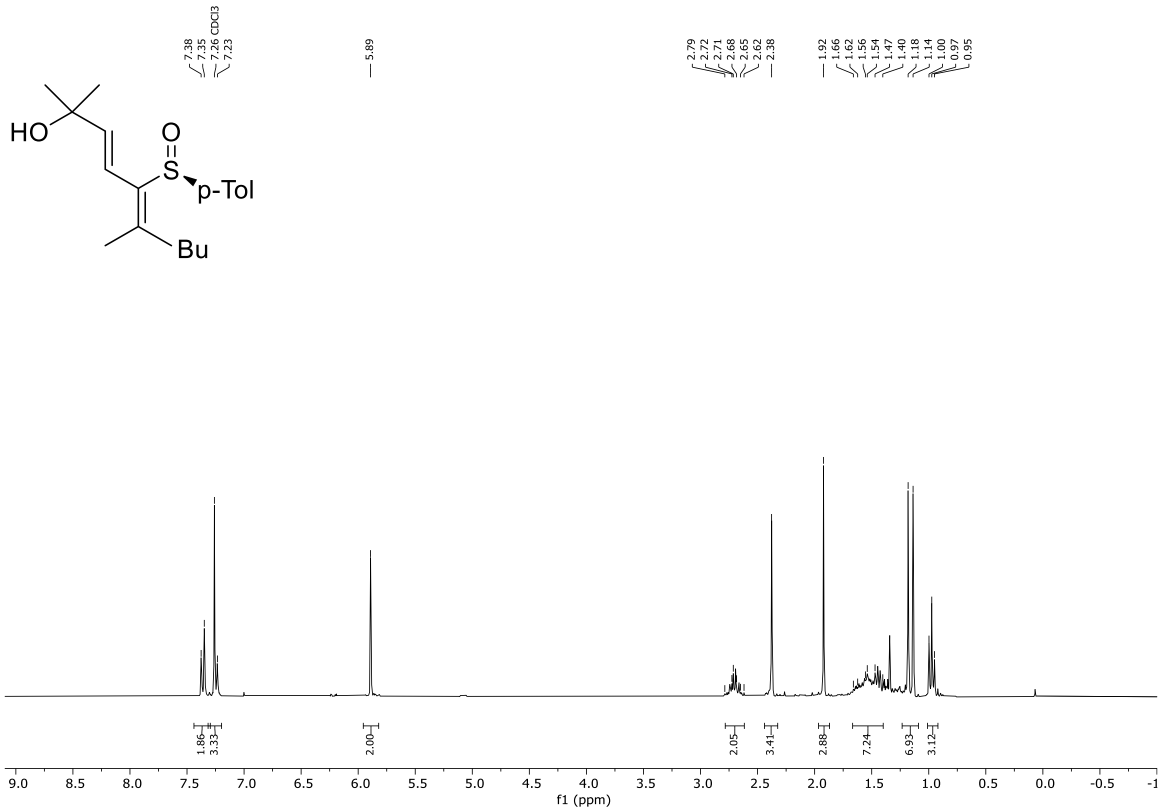

^{13}C NMR ($CDCl_3$, 125 MHz) (*E*,*Z*)-**1o**

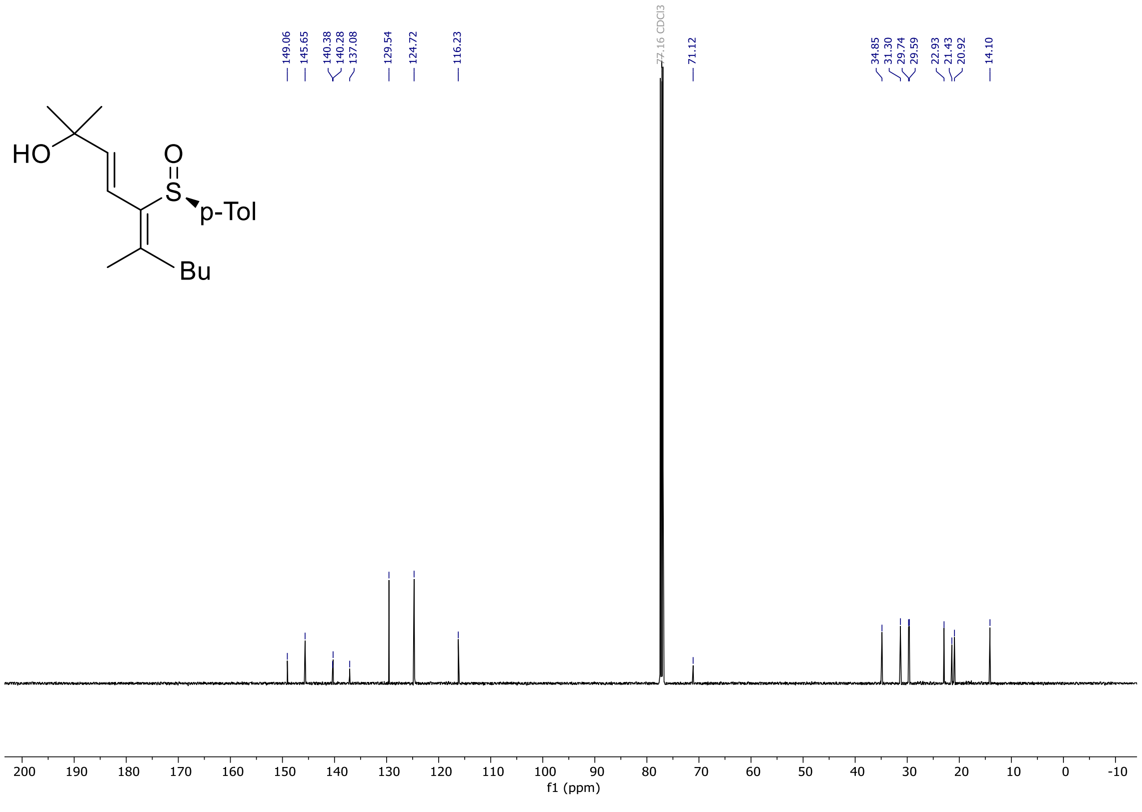

^{1}H NMR ($CDCl_3$, 400 MHz) (*E*,*Z*)-**1p**

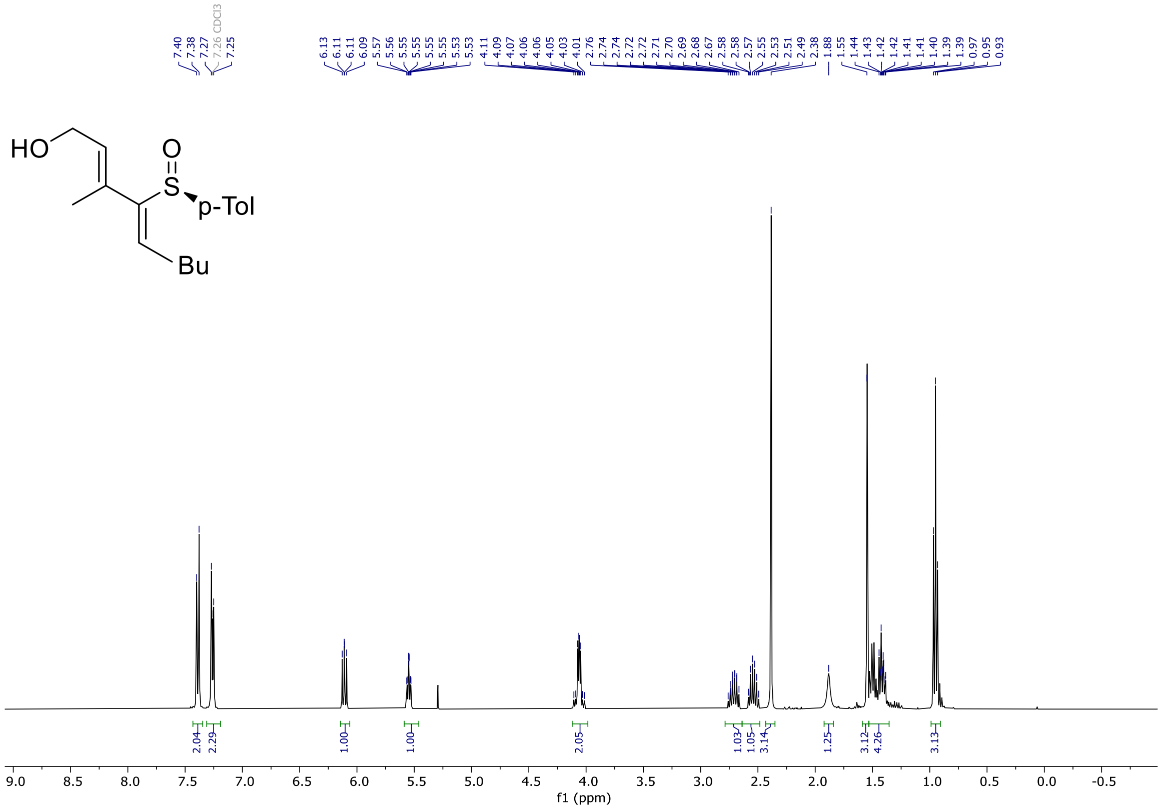

^{13}C NMR ($CDCl_3$, 100 MHz) (*E*,*Z*)-**1p**

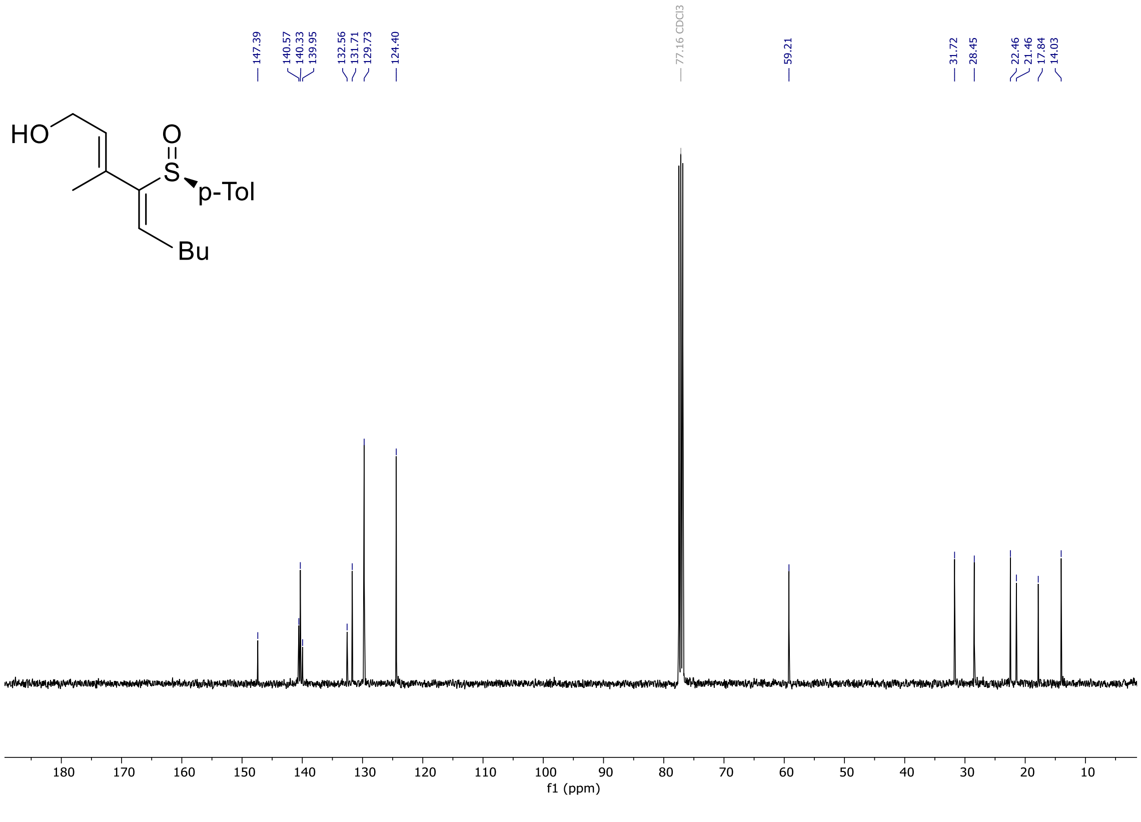

^{1}H NMR ($CDCl_3$, 400 MHz) (*E*,*Z*)-**1q**

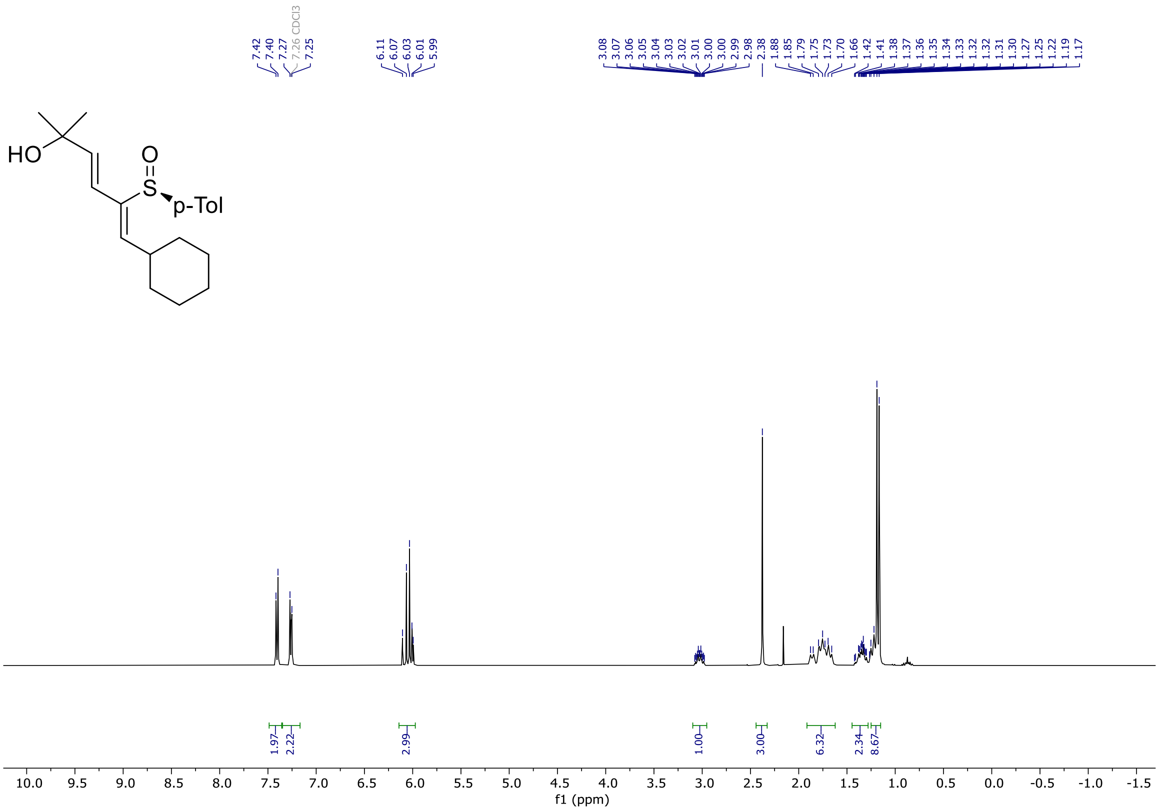

^{13}C NMR ($CDCl_3$, 100 MHz) (*E*,*Z*)-**1q**

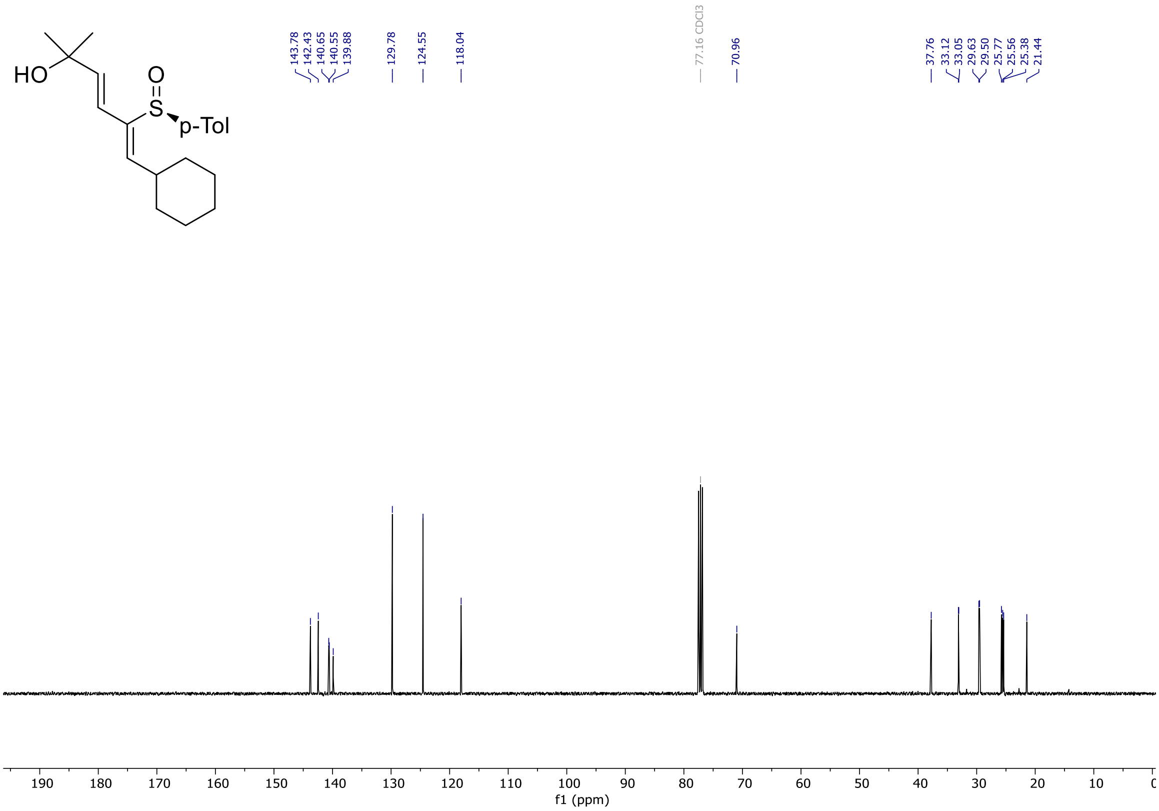

^{1}H NMR ($CDCl_3$, 400 MHz) (*E*,*Z*)-**1r**

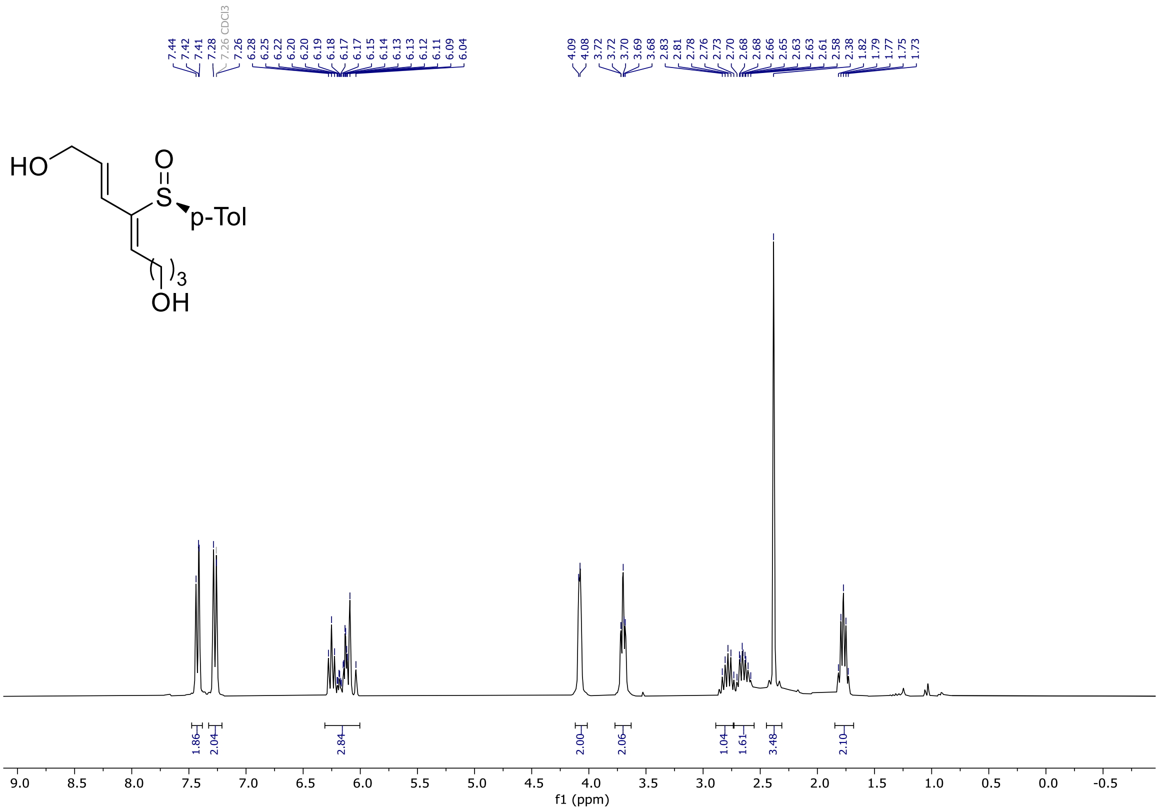

^{13}C NMR ($CDCl_3$, 100 MHz) (*E*,*Z*)-**1r**

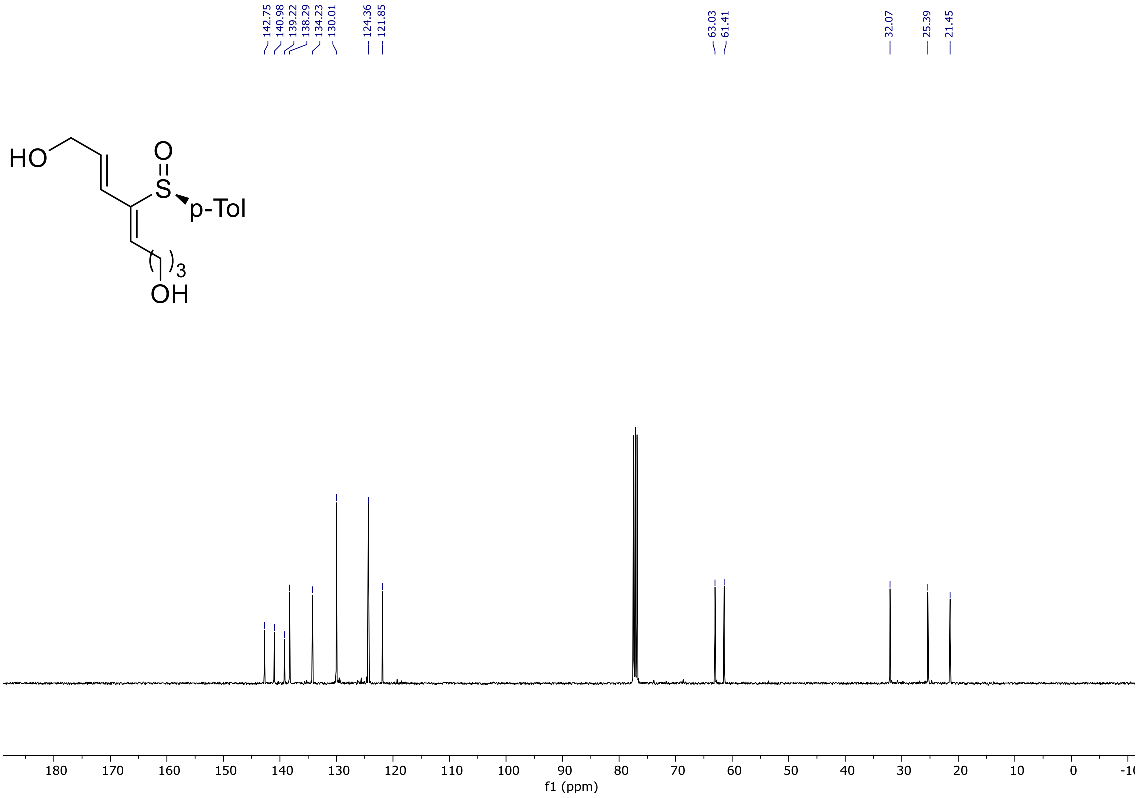

^{1}H NMR ($CDCl_3$, 400 MHz) (*E*,*Z*)-**1s**

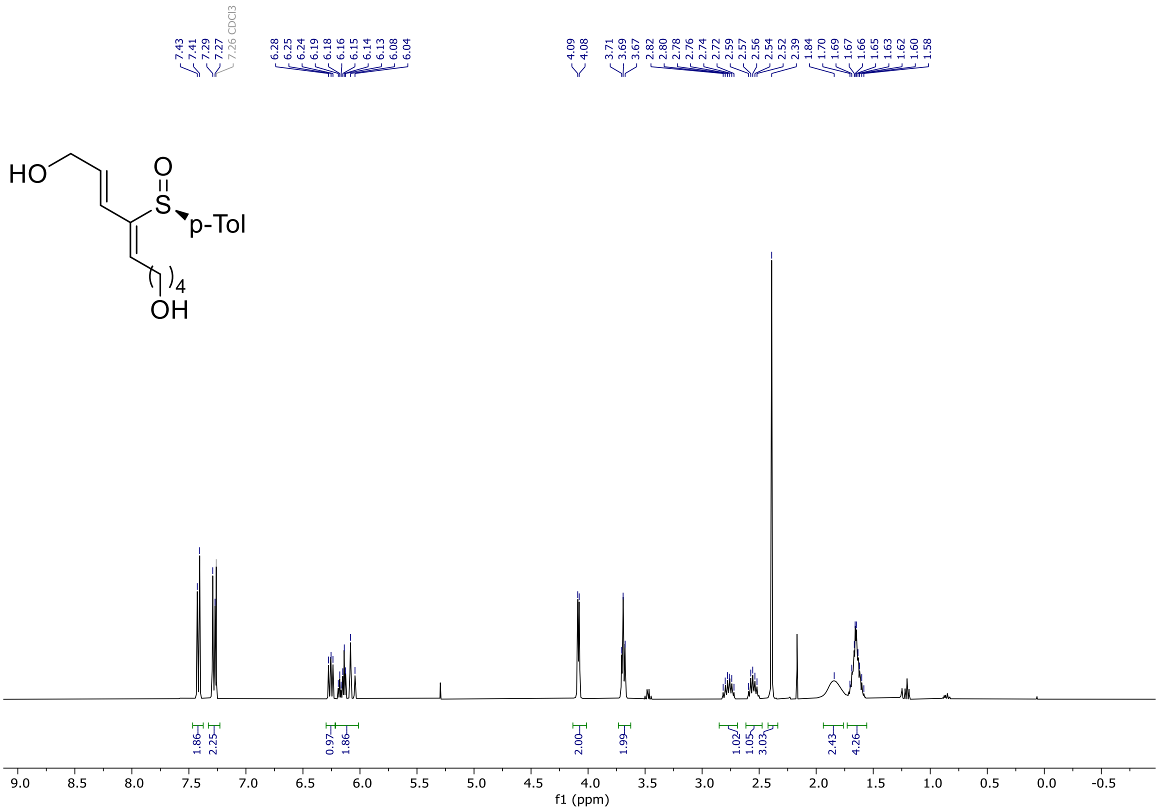

^{13}C NMR ($CDCl_3$, 100 MHz) (*E*,*Z*)-**1s**

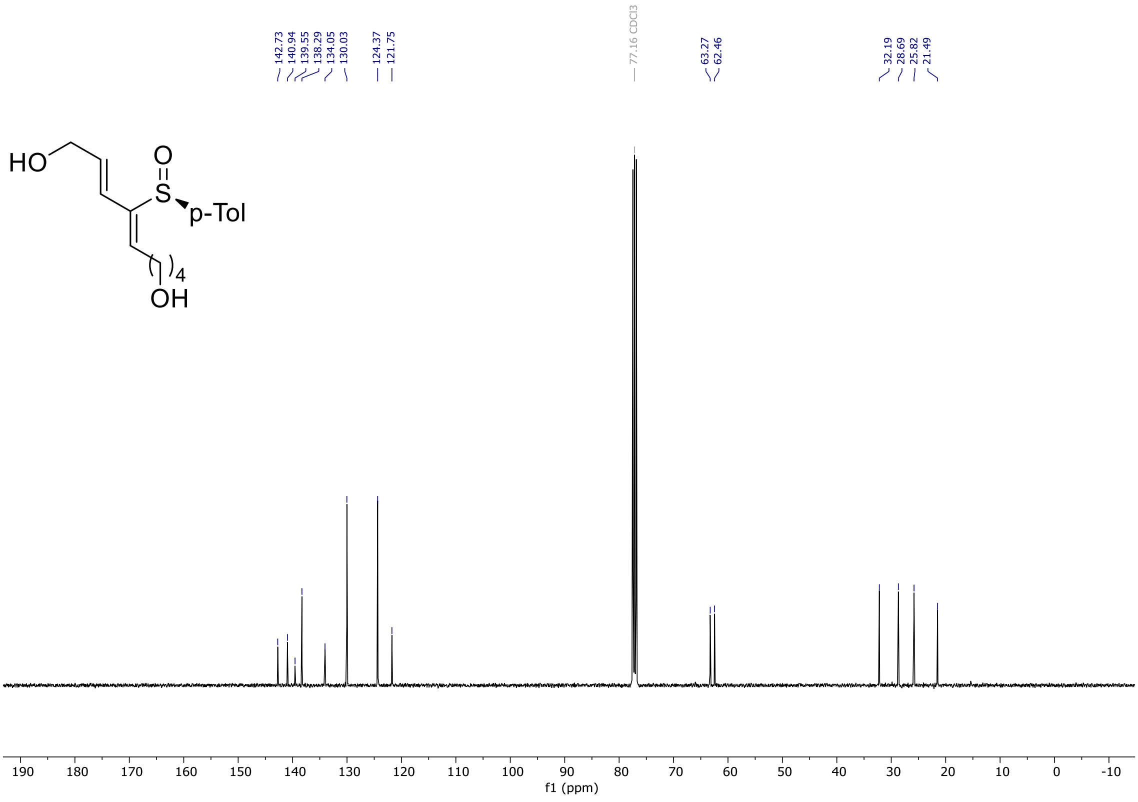

^{1}H NMR ($CDCl_3$, 500 MHz) **4a**

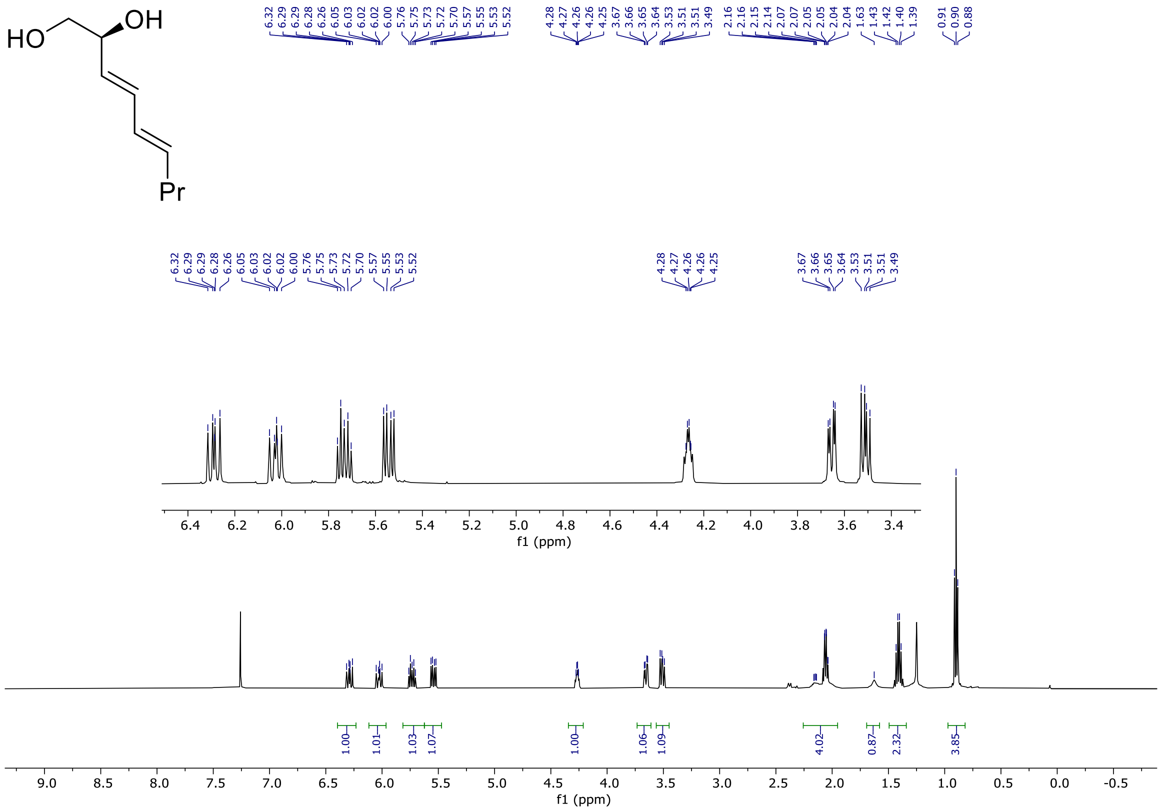

^{13}C NMR ($CDCl_3$, 125 MHz) **4a**

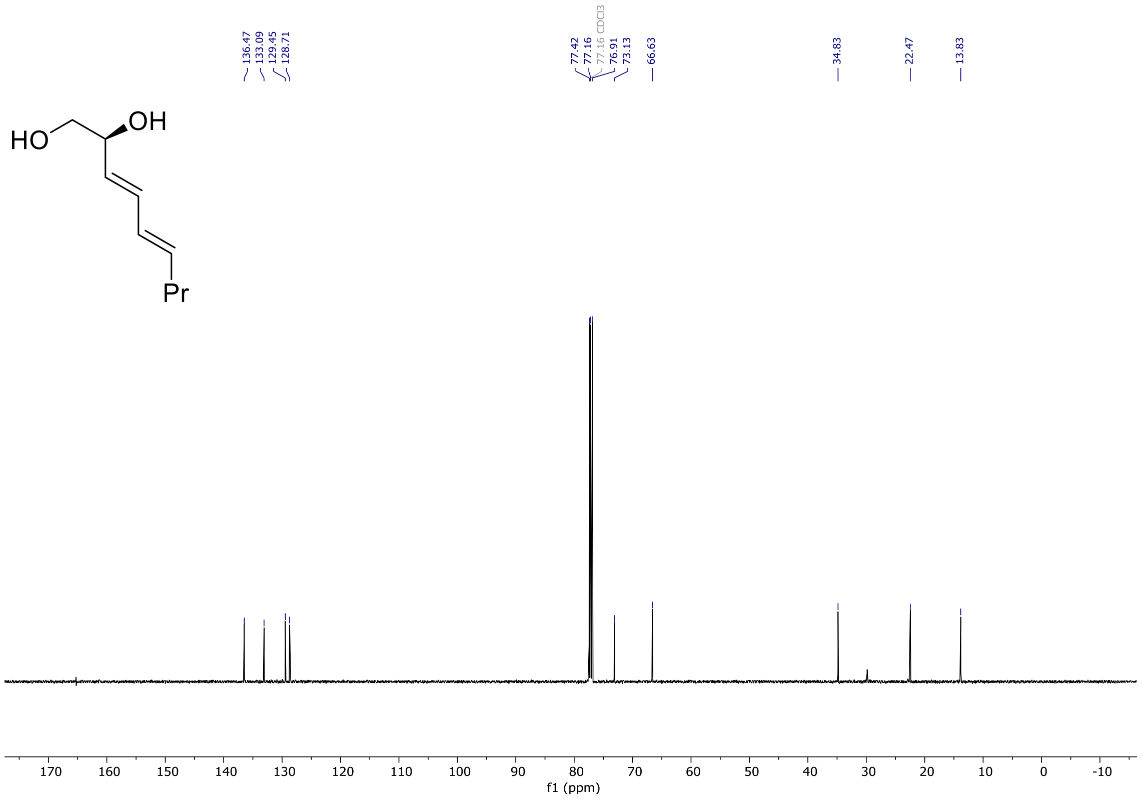

COSY (^{1}H, ^{1}H) **4a**

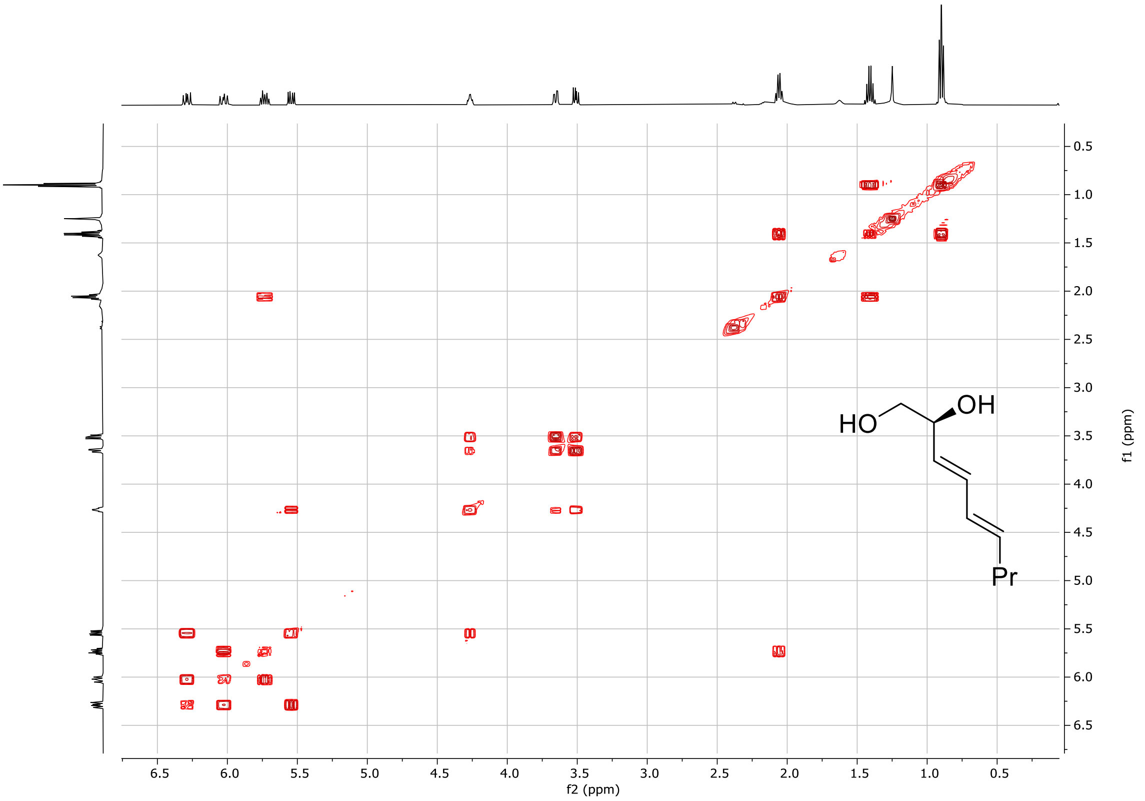

^{1}H NMR ($CDCl_3$, 400 MHz) **6a**

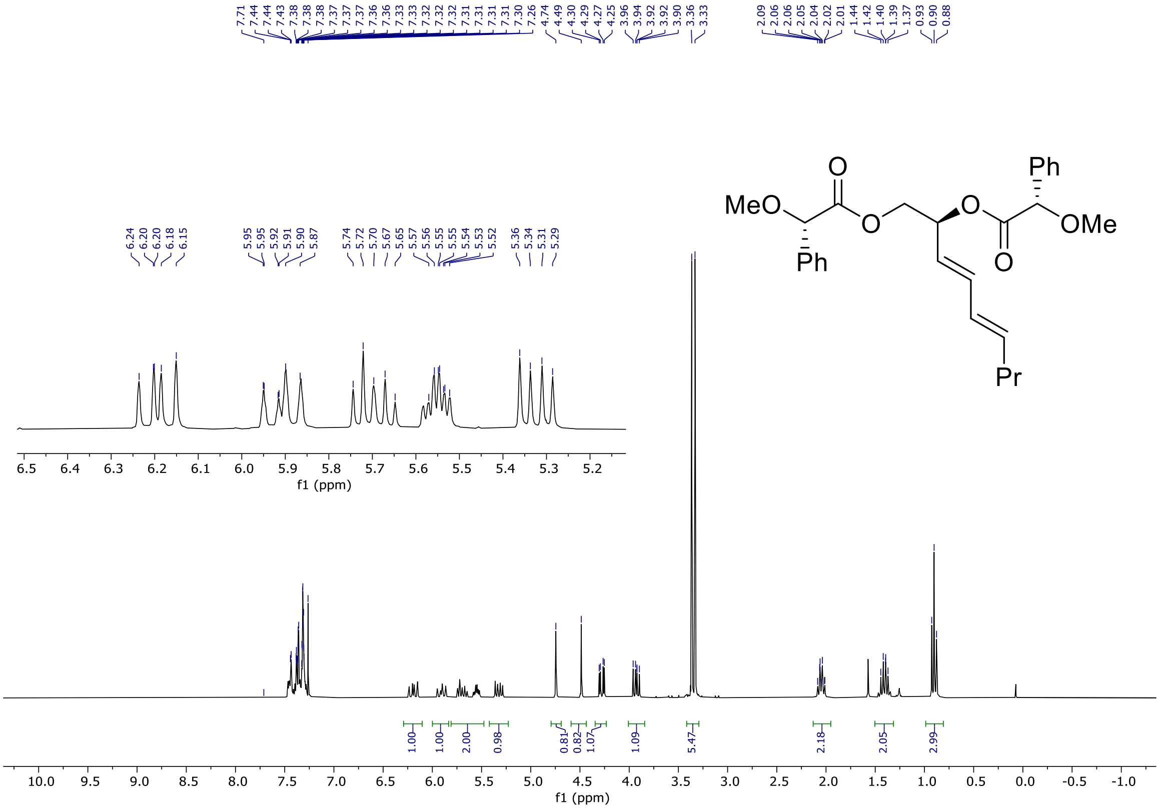

^{13}C NMR ($CDCl_3$, 100 MHz) **6a**

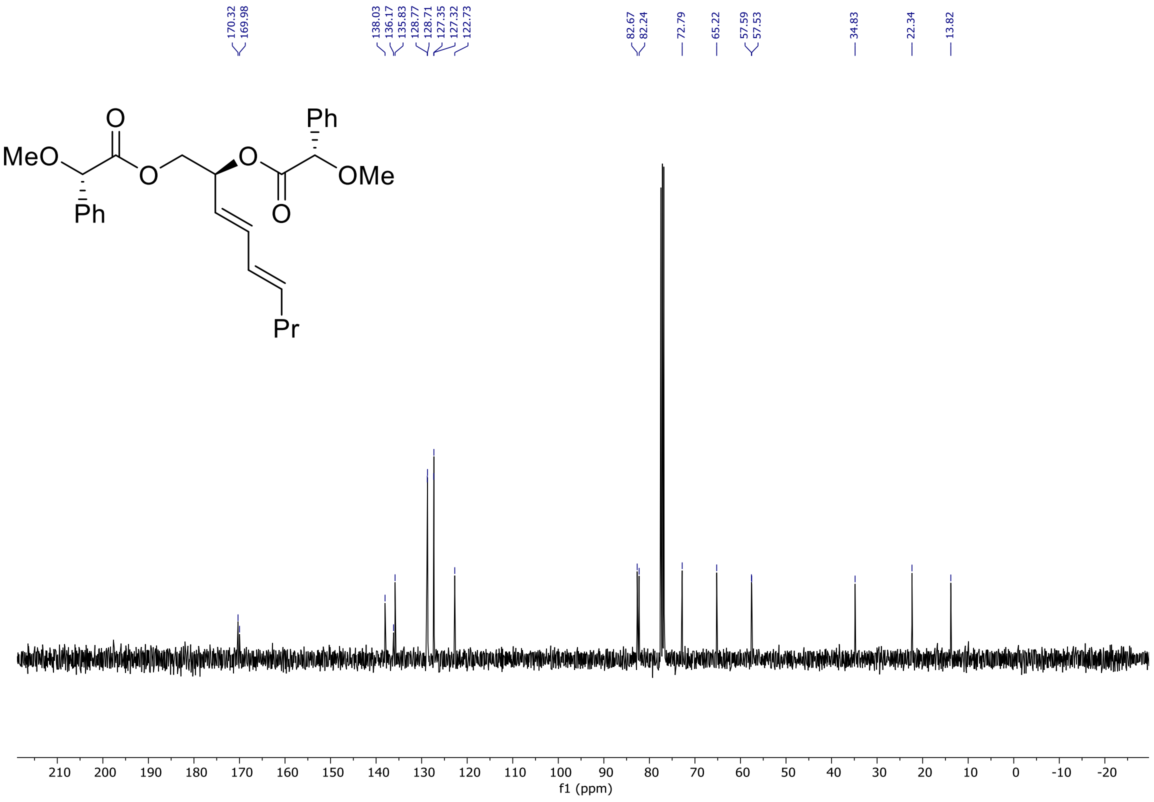

COSY (^1H, ^1H) **6a**

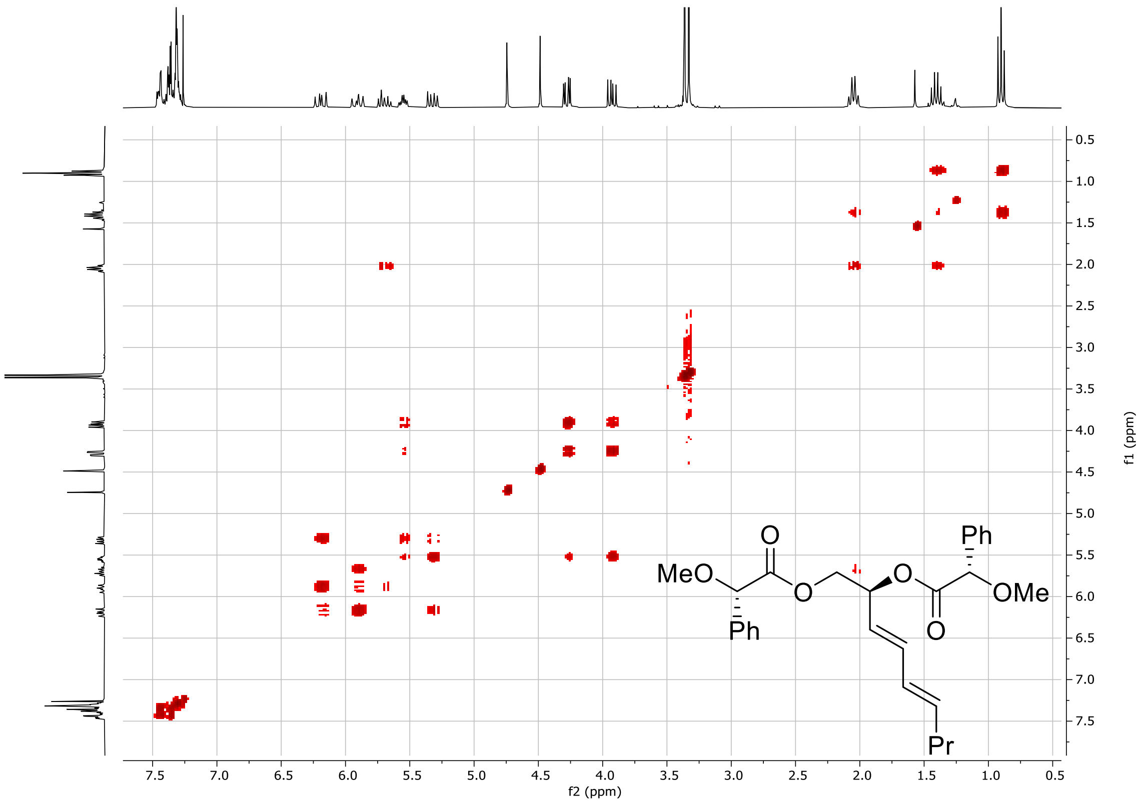

HSQC (^{1}H, ^{13}C) **6a**

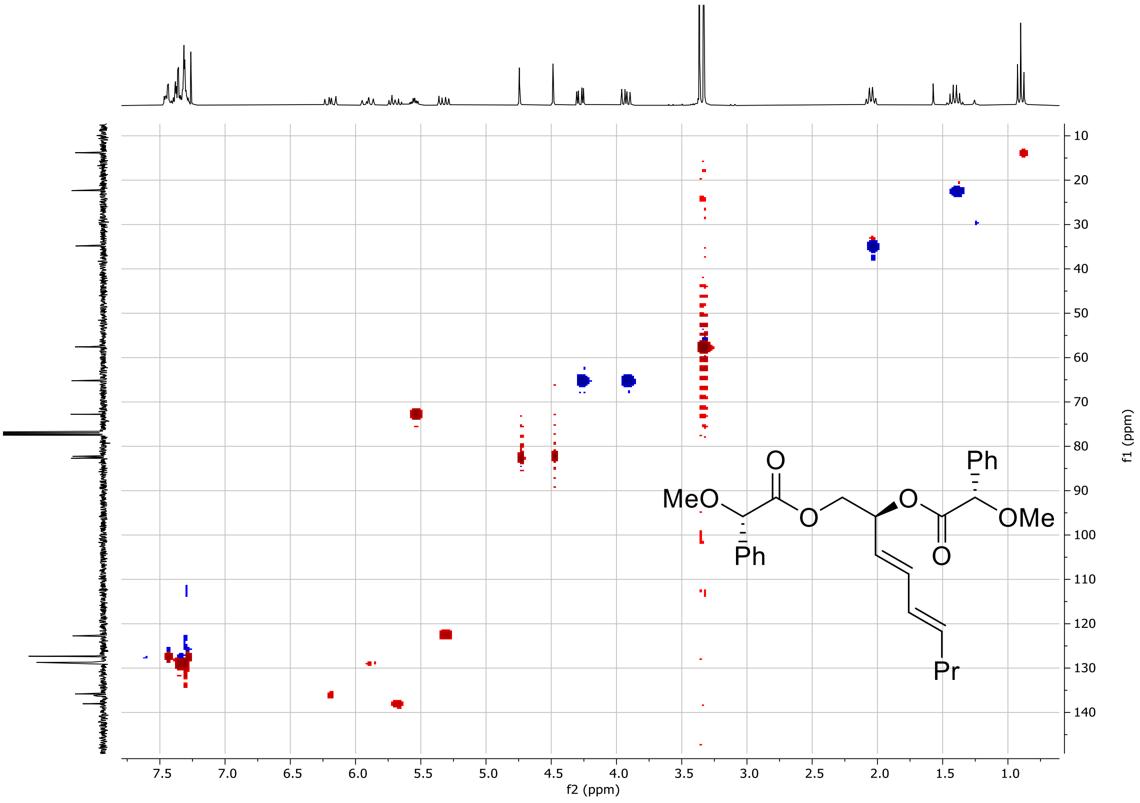

^{1}H NMR ($CDCl_3$, 500 MHz) **7a**

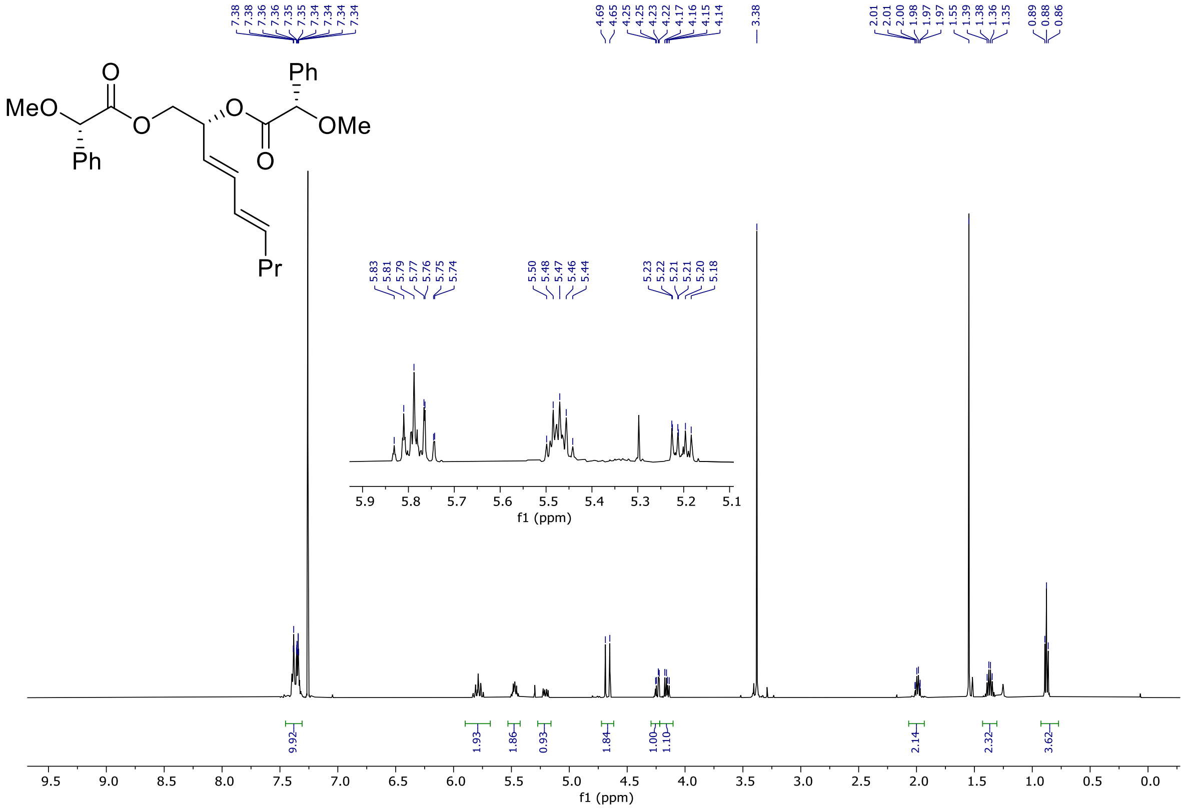

^{13}C NMR (CDCl$_3$, 125 MHz) **7a**

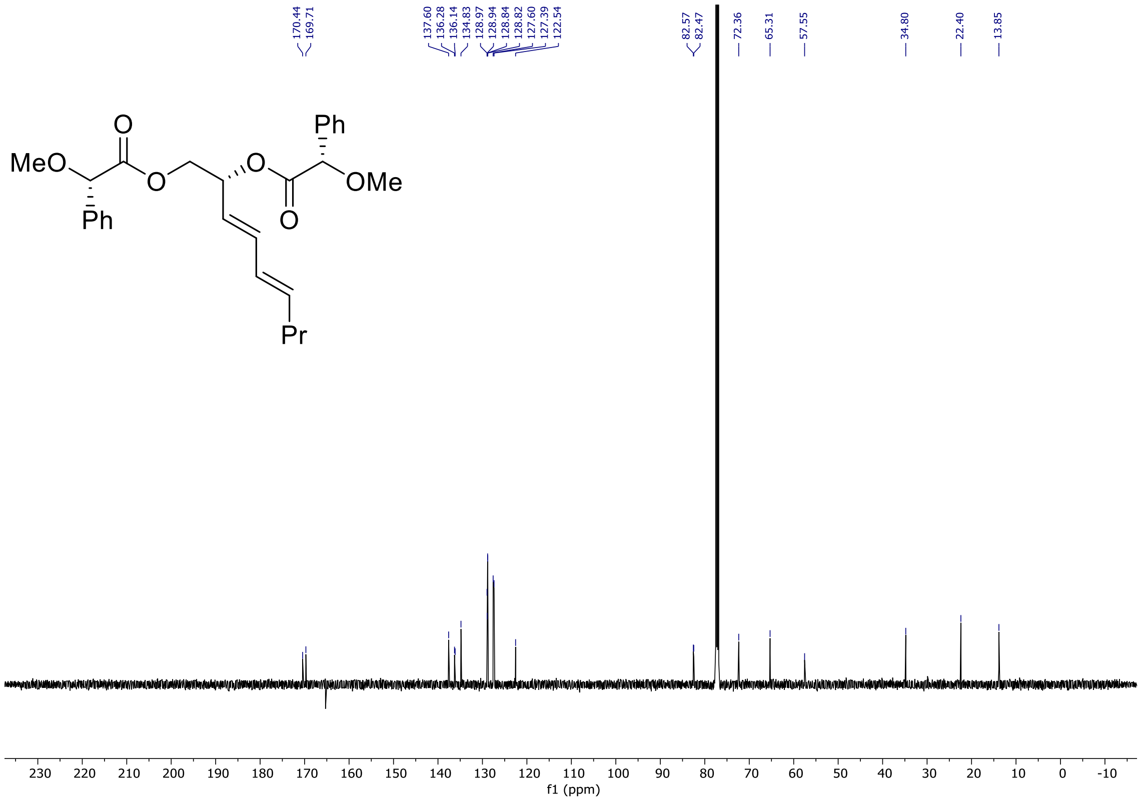

^{1}H NMR (CDCl$_3$, 500 MHz) **4b**

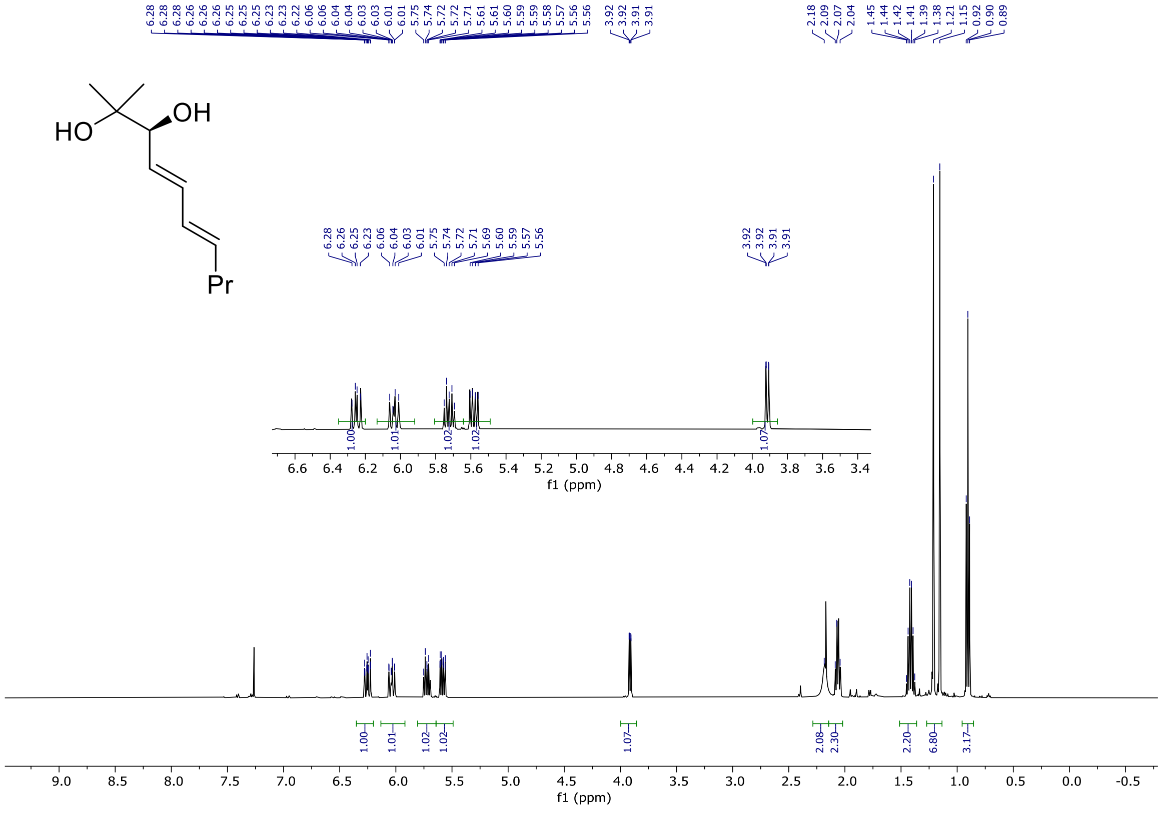

^{13}C NMR ($CDCl_3$, 125 MHz) **4b**

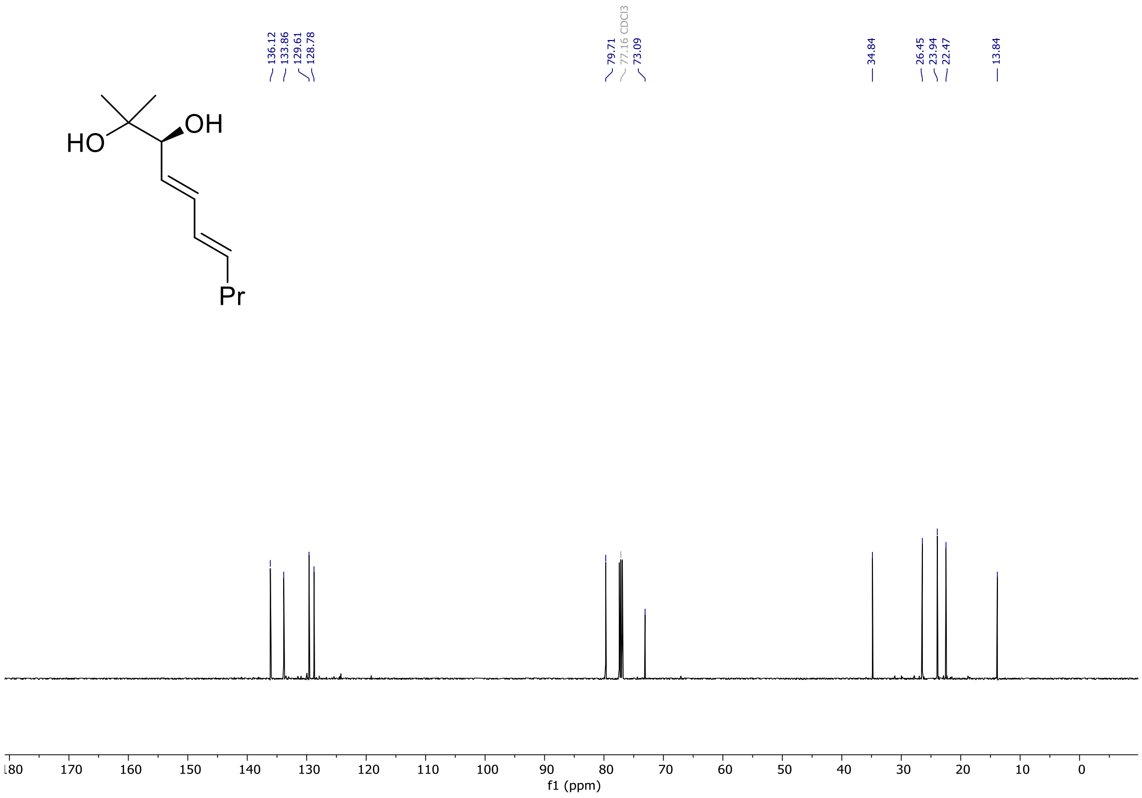

COSY (^1H, ^1H) **4b**

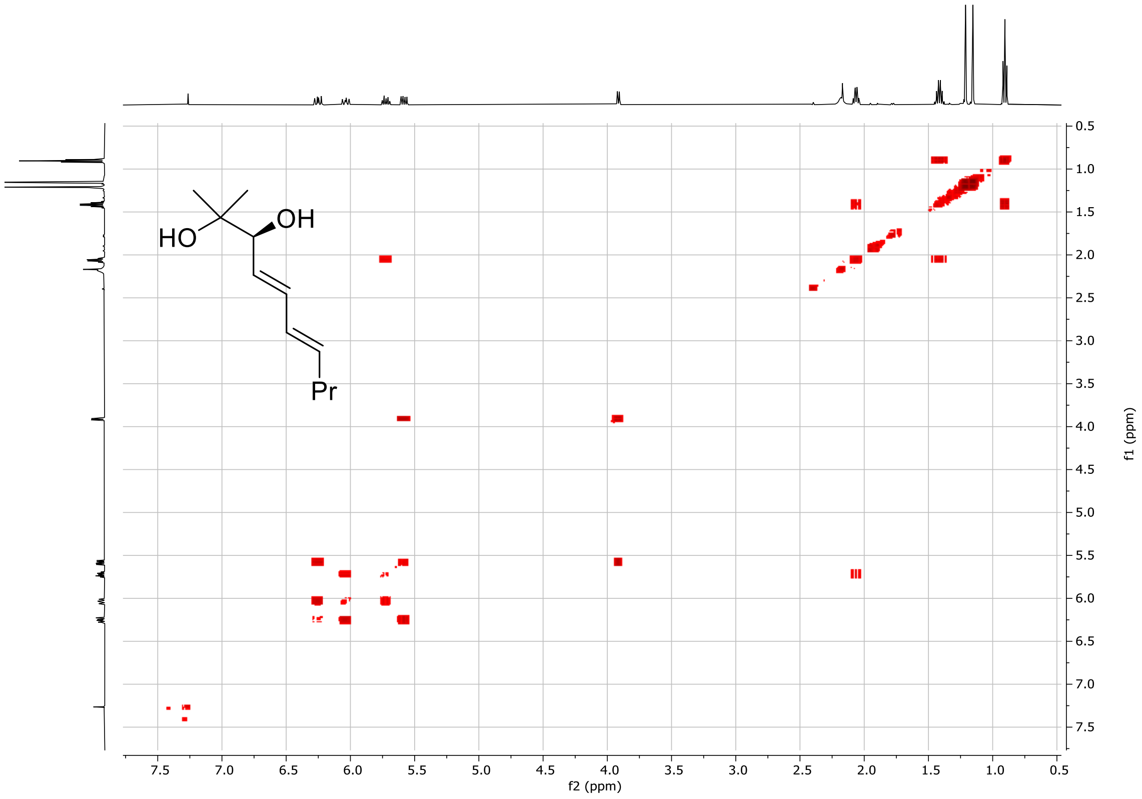

HSQC (^{1}H, ^{13}C) **4b**

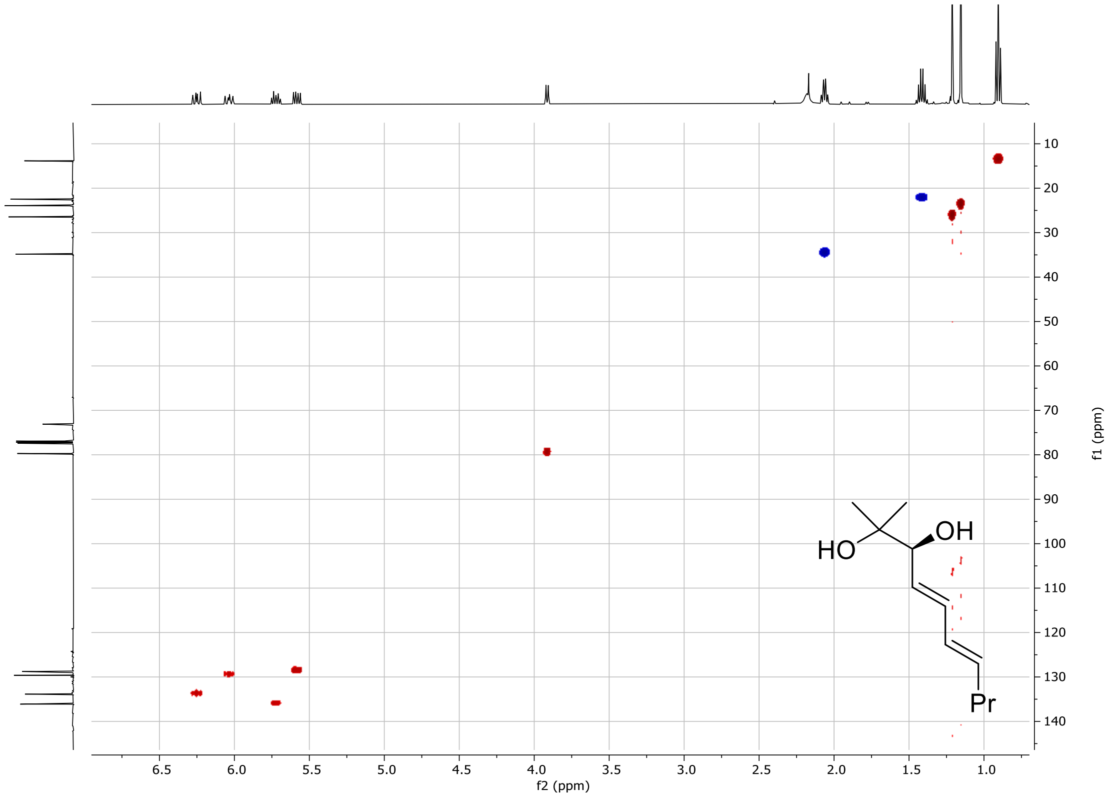

^{1}H NMR ($CDCl_3$, 400 MHz) **6b**

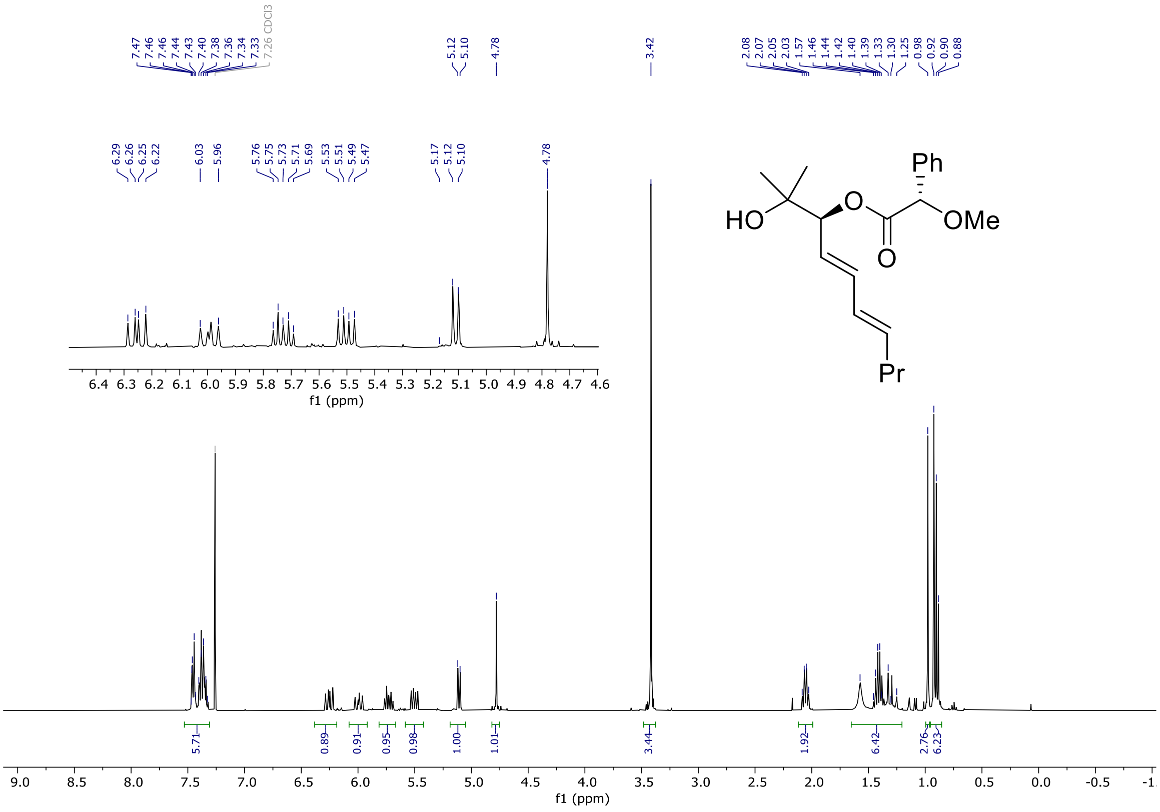

^{13}C NMR ($CDCl_3$, 100 MHz) **6b**

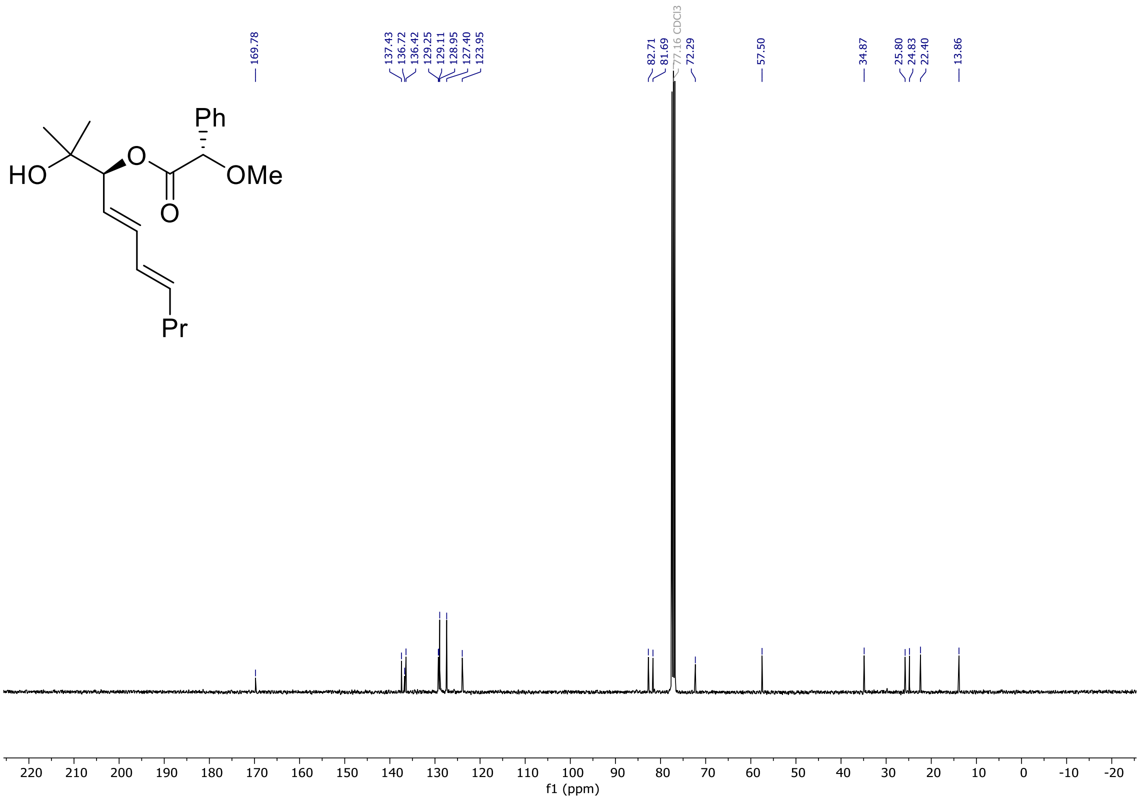

COSY (^1H, ^1H) **6b**

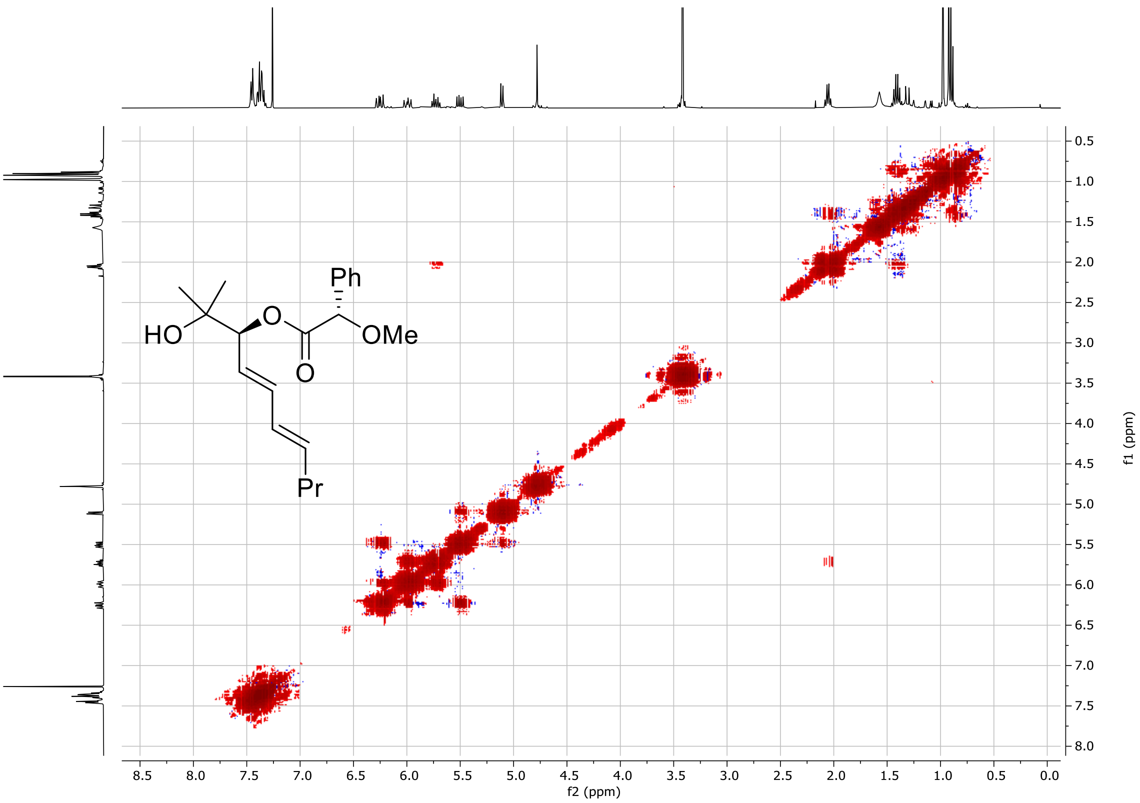

HSQC (^{1}H, ^{13}C) **6b**

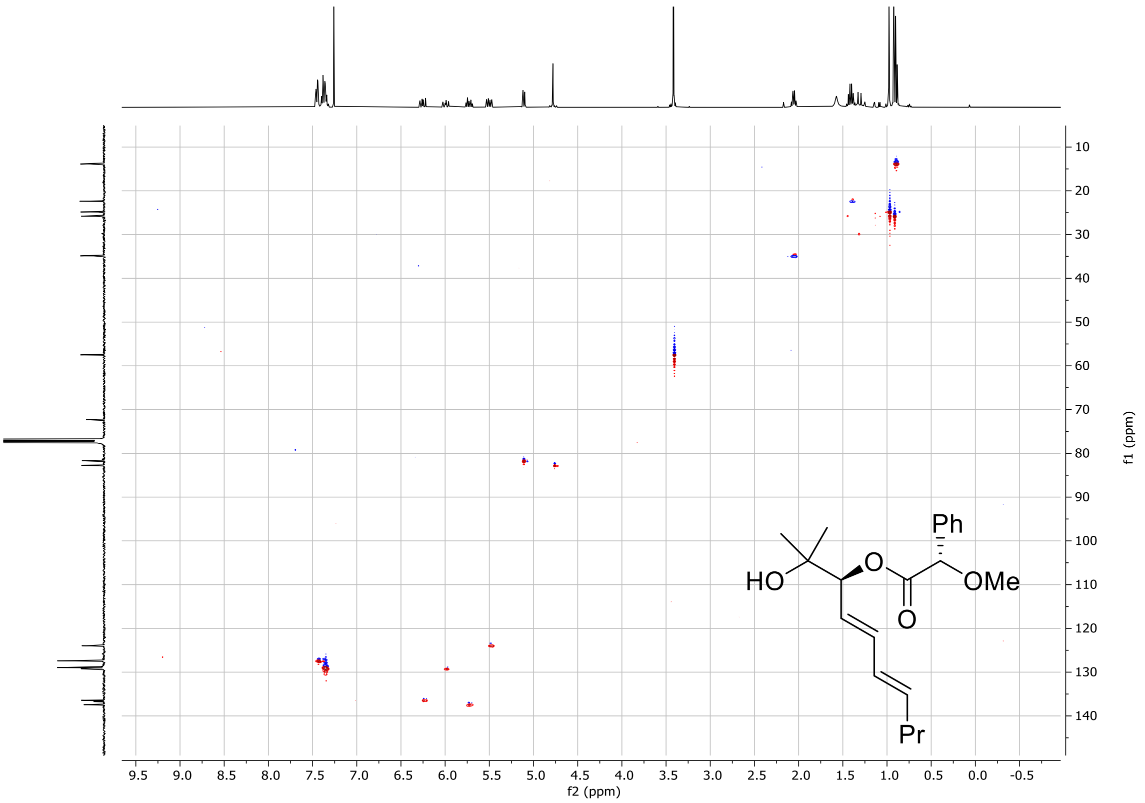

^{1}H NMR ($CDCl_3$, 400 MHz) **6b'**

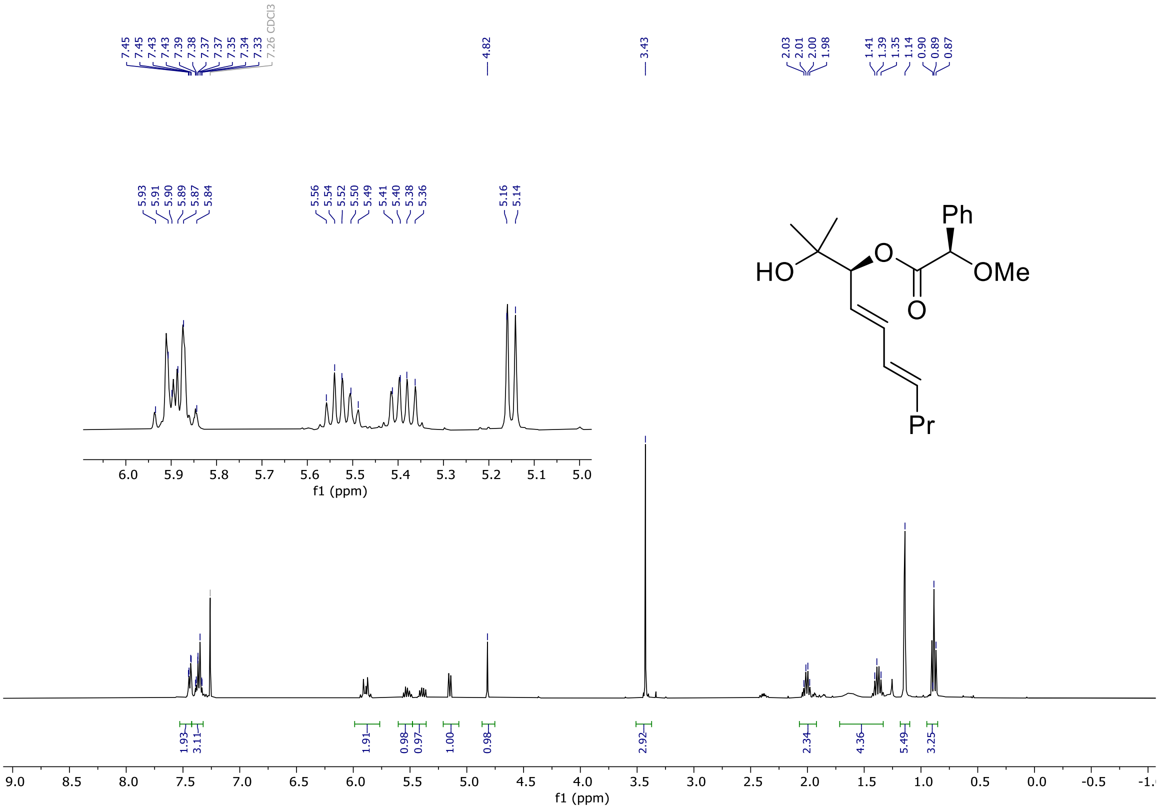

^{13}C NMR ($CDCl_3$, 100 MHz) **6b'**

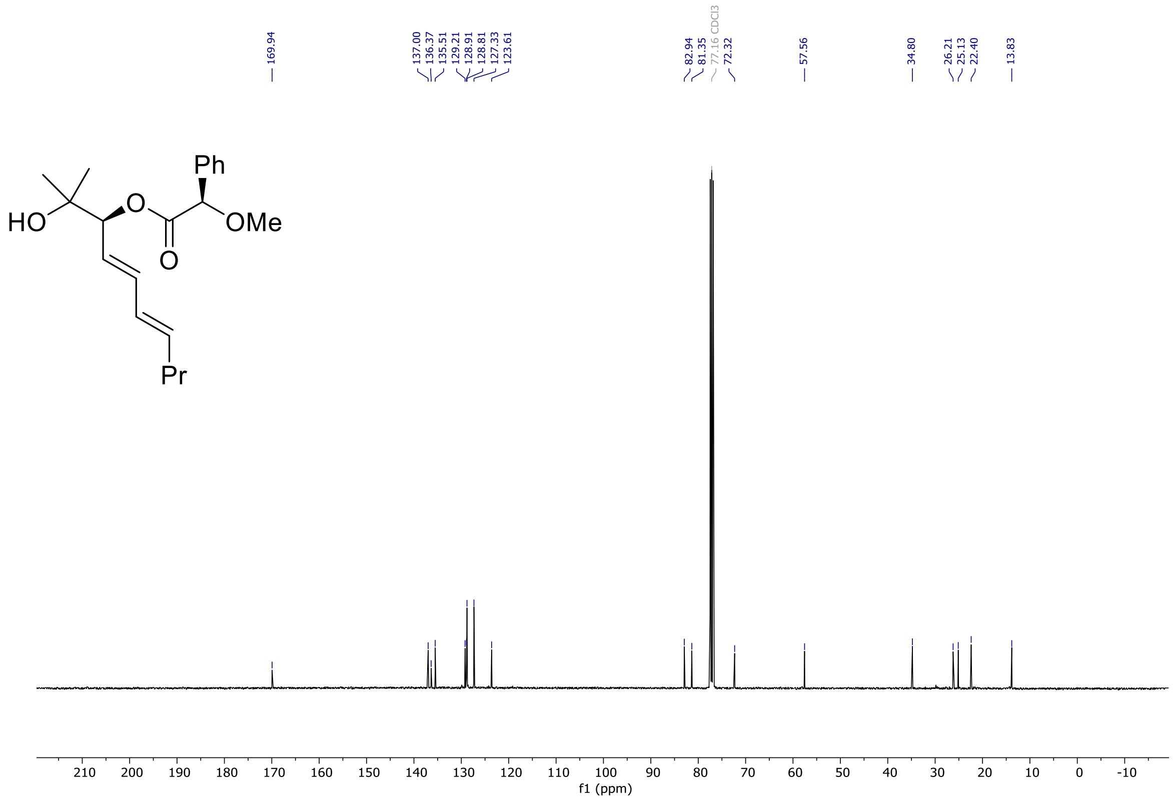

COSY (^1H, ^1H) **6b'**

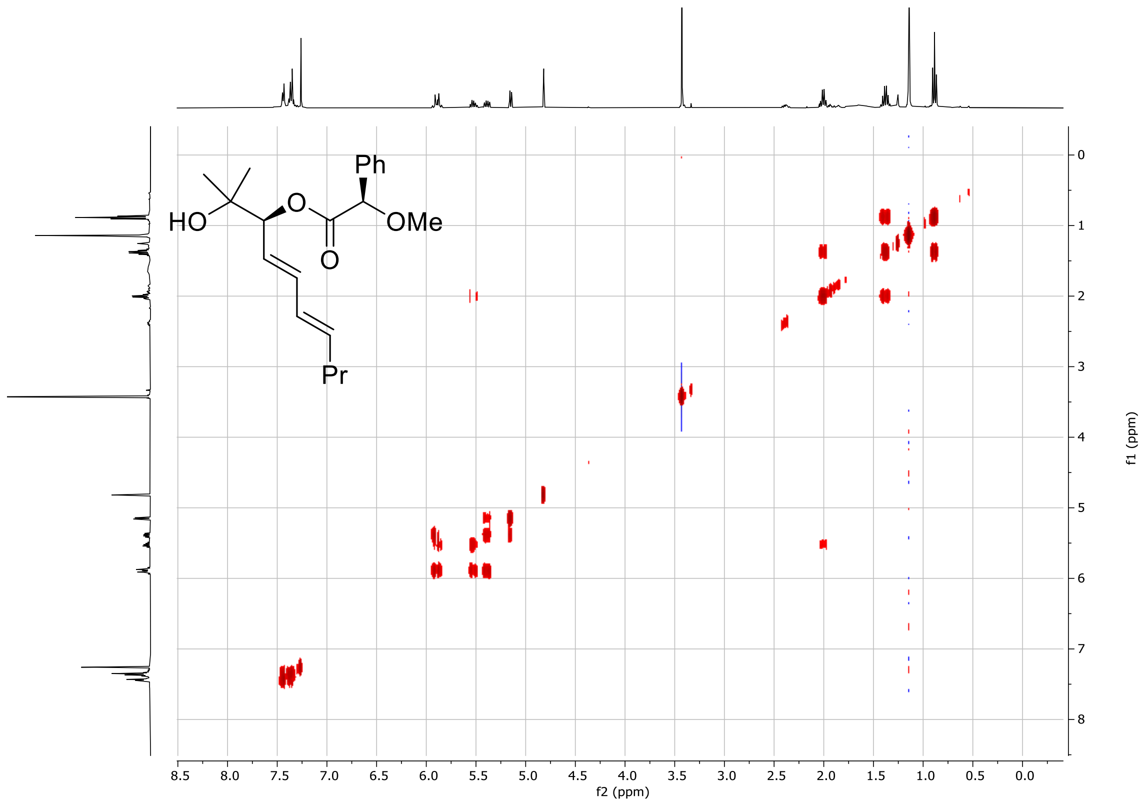

HSQC (^{1}H, ^{13}C) **6b’**

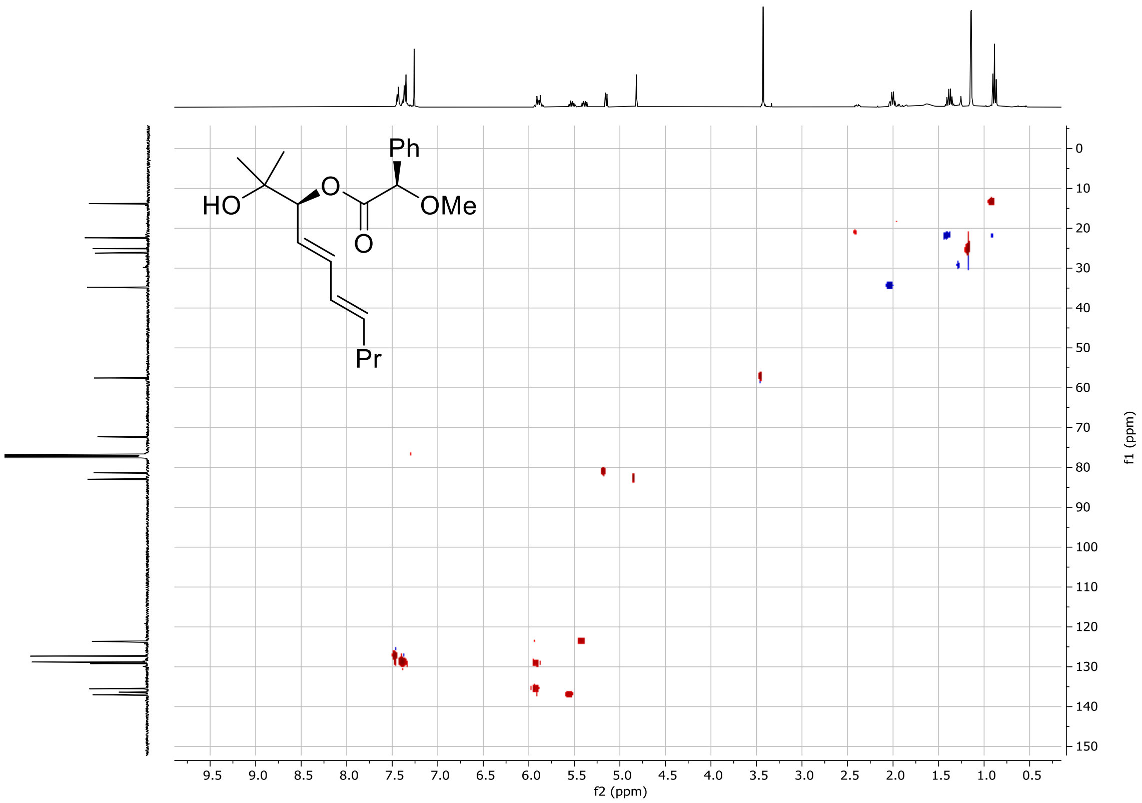

^{1}H NMR (CDCl$_3$, 400 MHz) **4c**

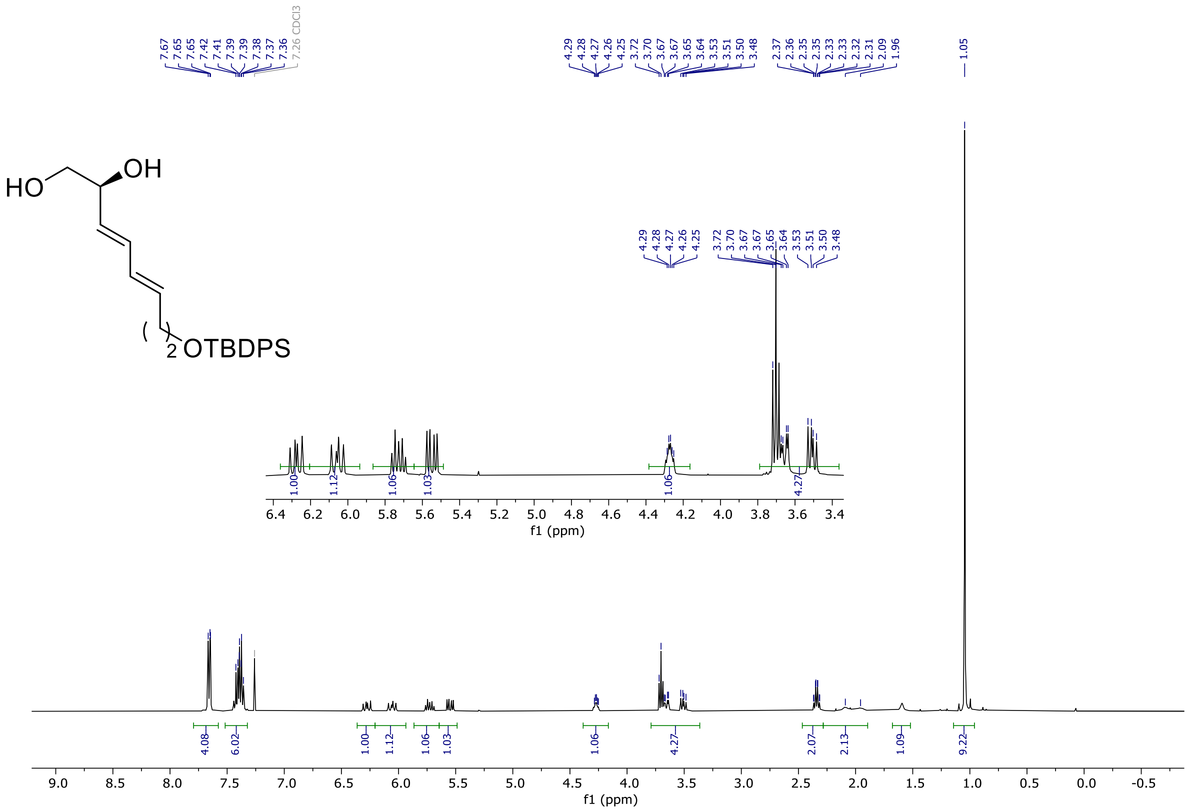

^{13}C NMR ($CDCl_3$, 100 MHz) **4c**

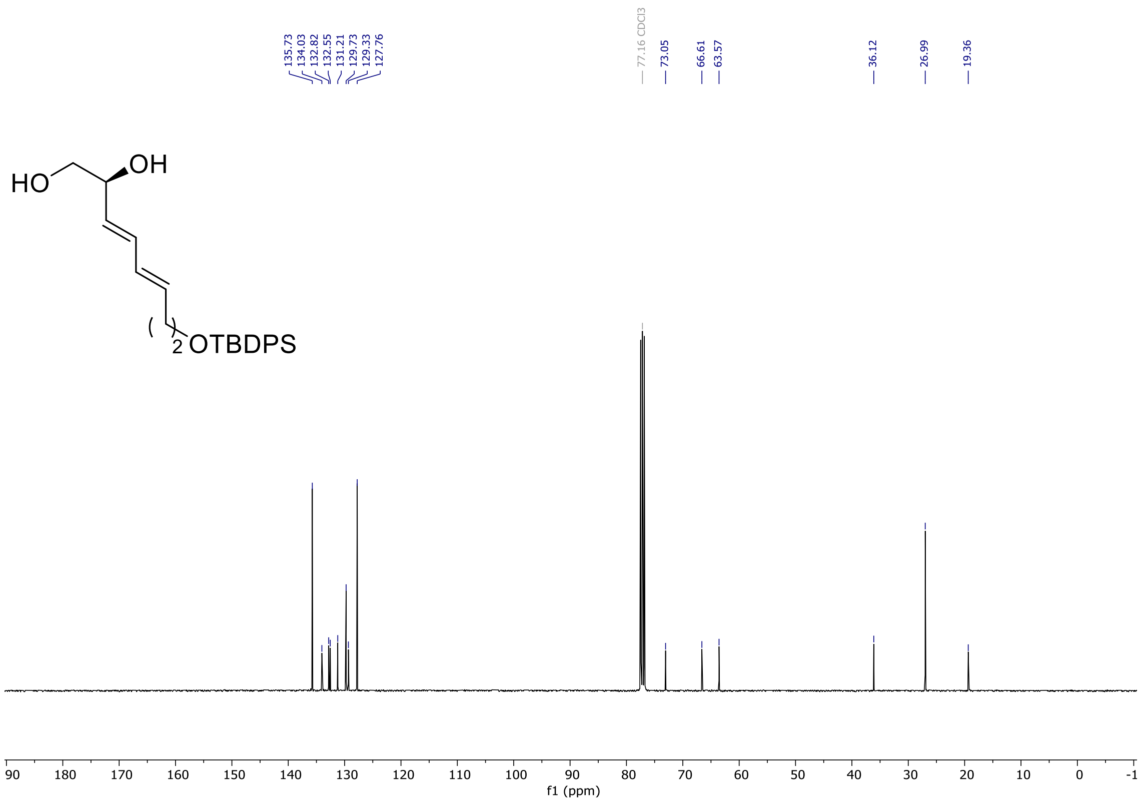

^{1}H NMR ($CDCl_3$, 400 MHz) **6c**

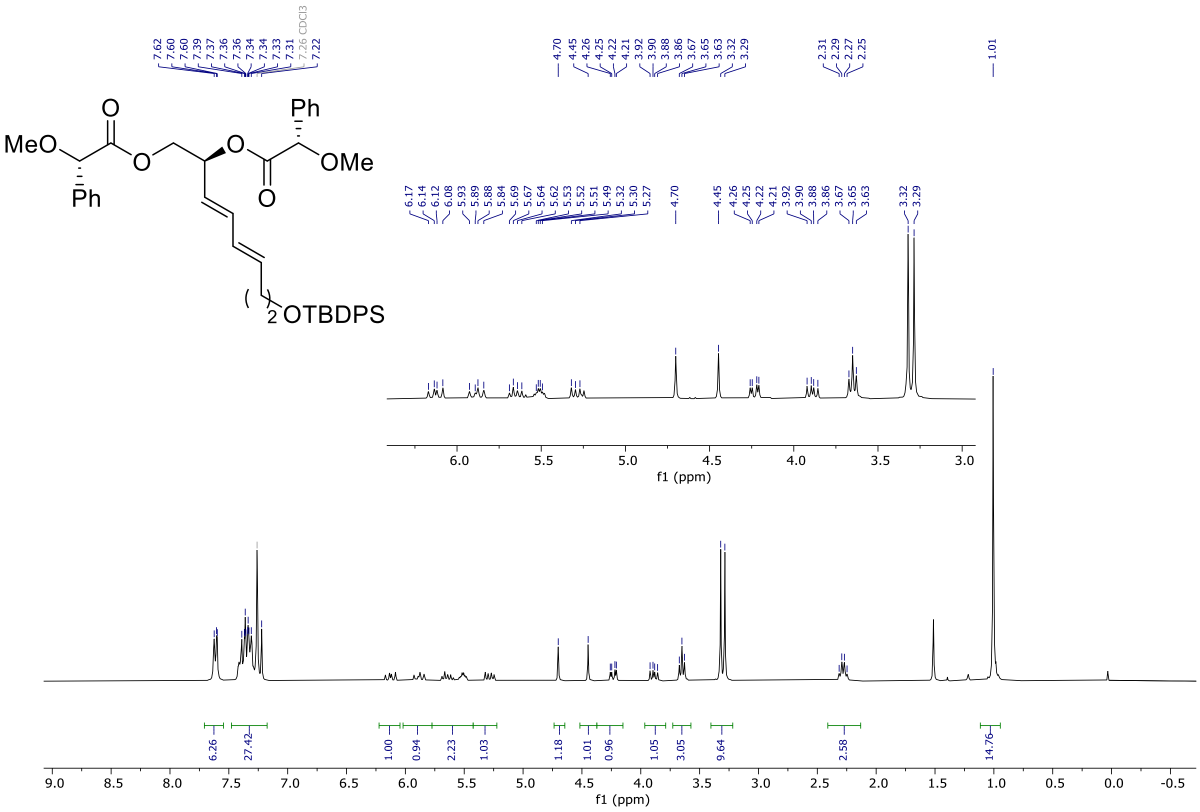

^{13}C NMR ($CDCl_3$, 100 MHz) **6c**

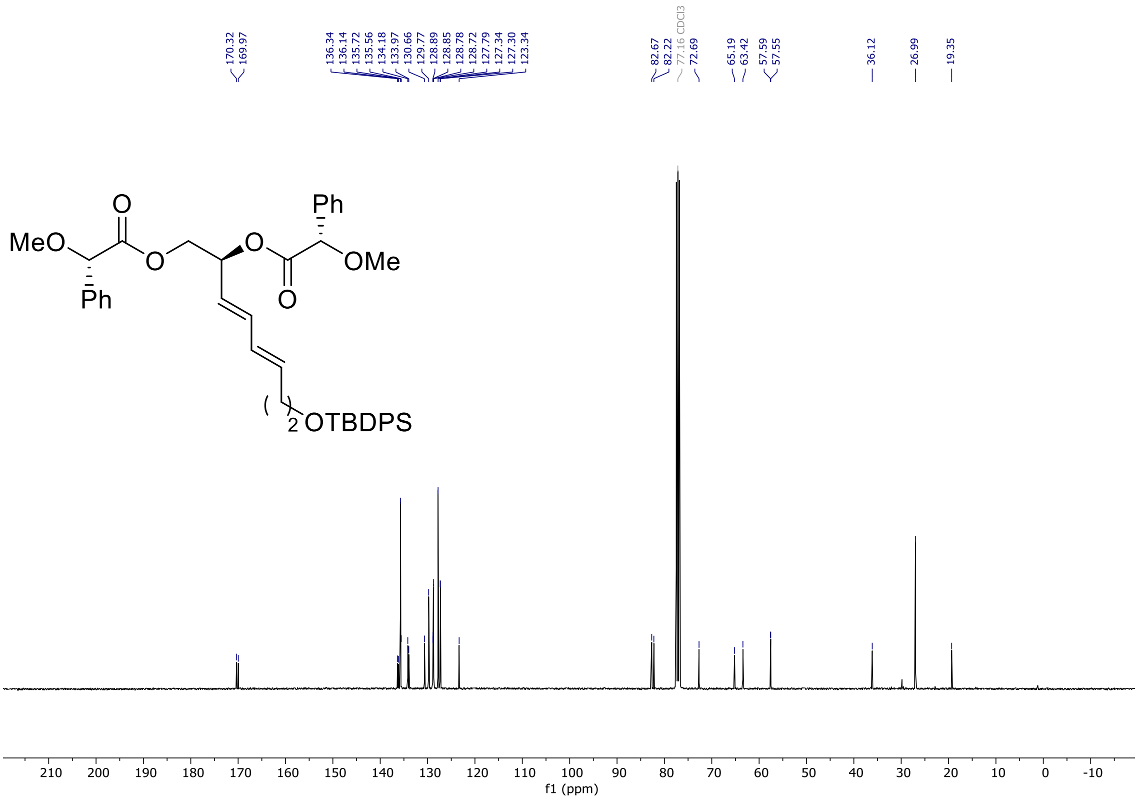

^{1}H NMR ($CDCl_3$, 400 MHz) **7c**

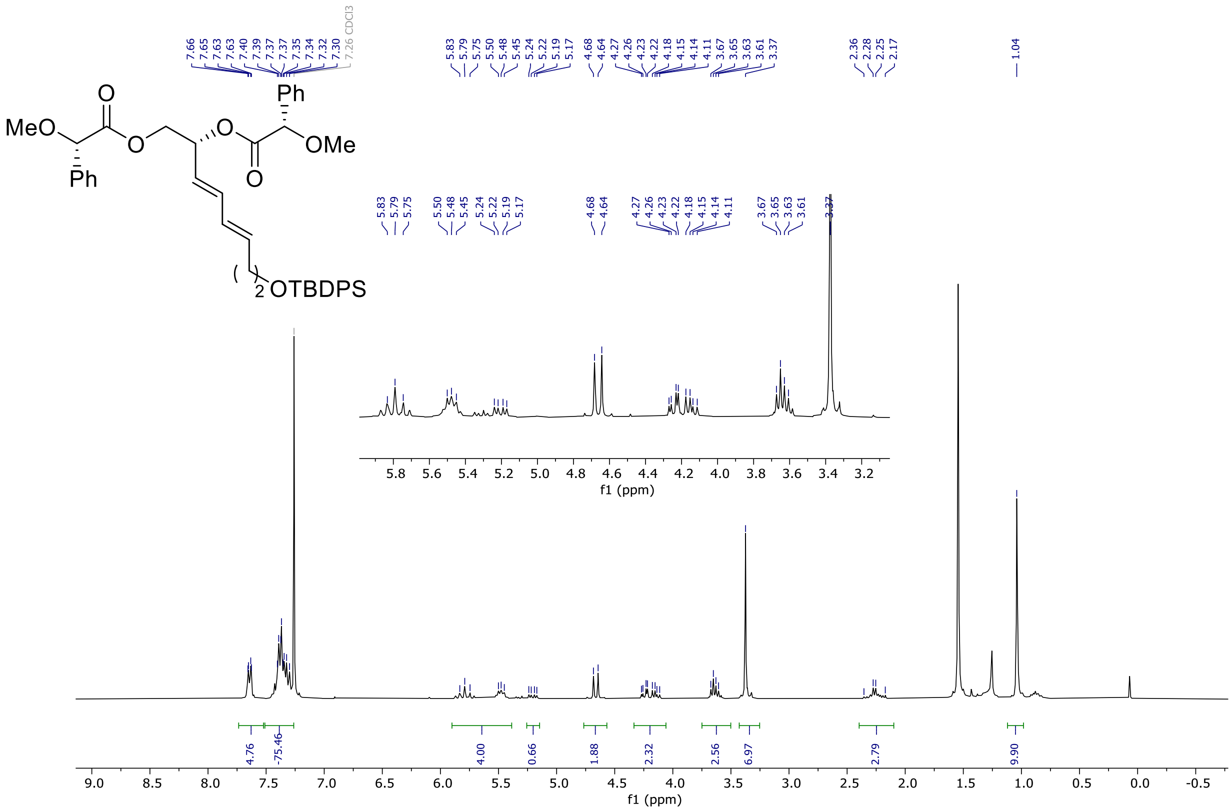

^{1}H NMR ($CDCl_3$, 400 MHz) **4d**

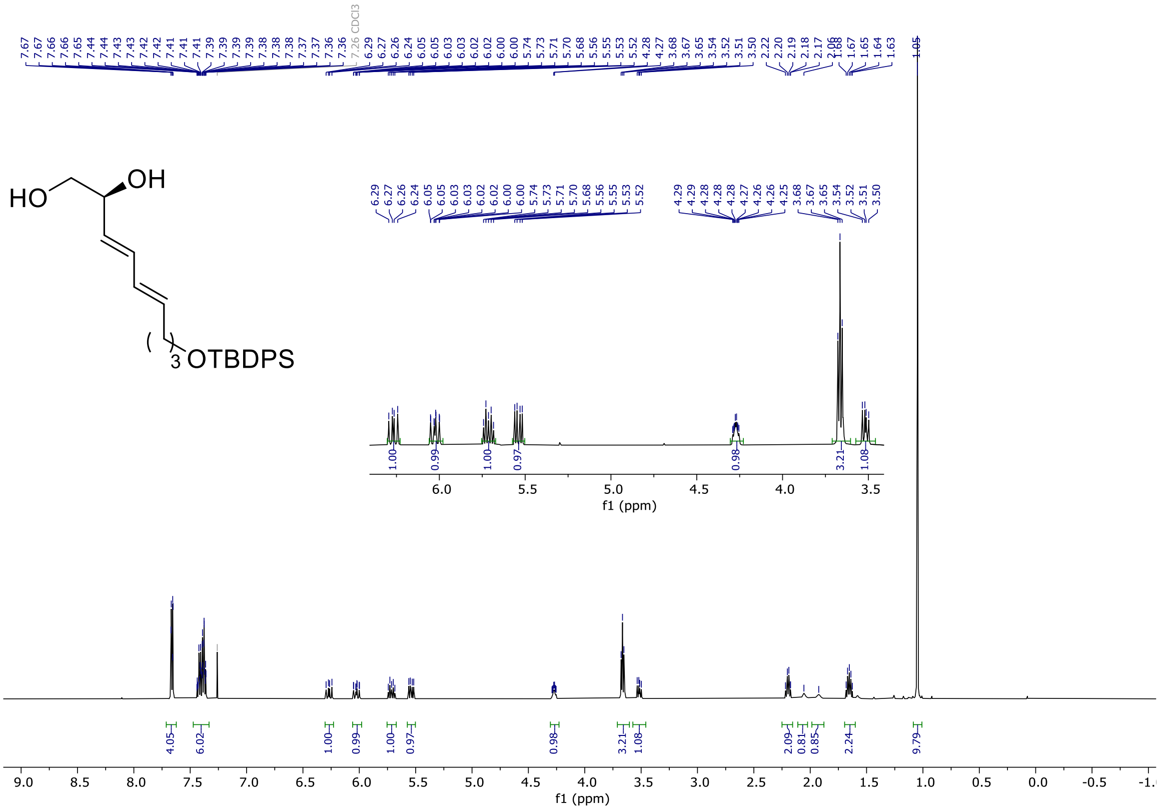

^{13}C NMR ($CDCl_3$, 100 MHz) **4d**

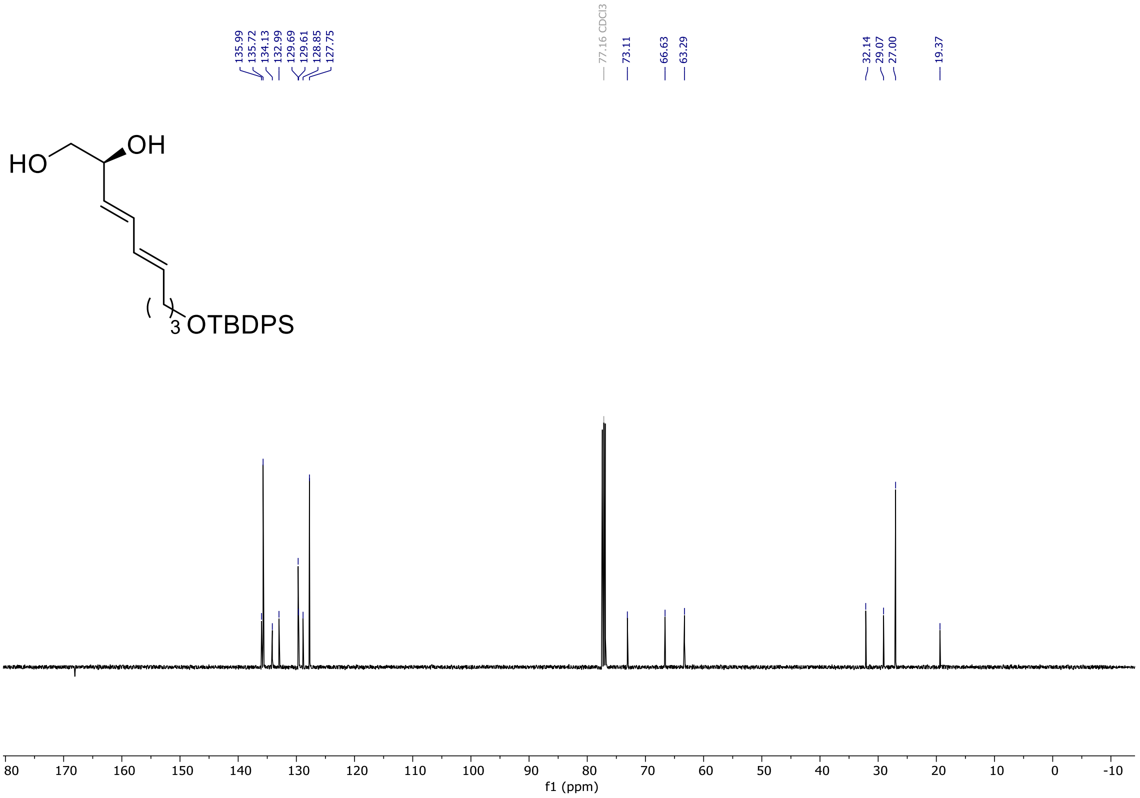

^{1}H NMR ($CDCl_3$, 500 MHz) **6d**

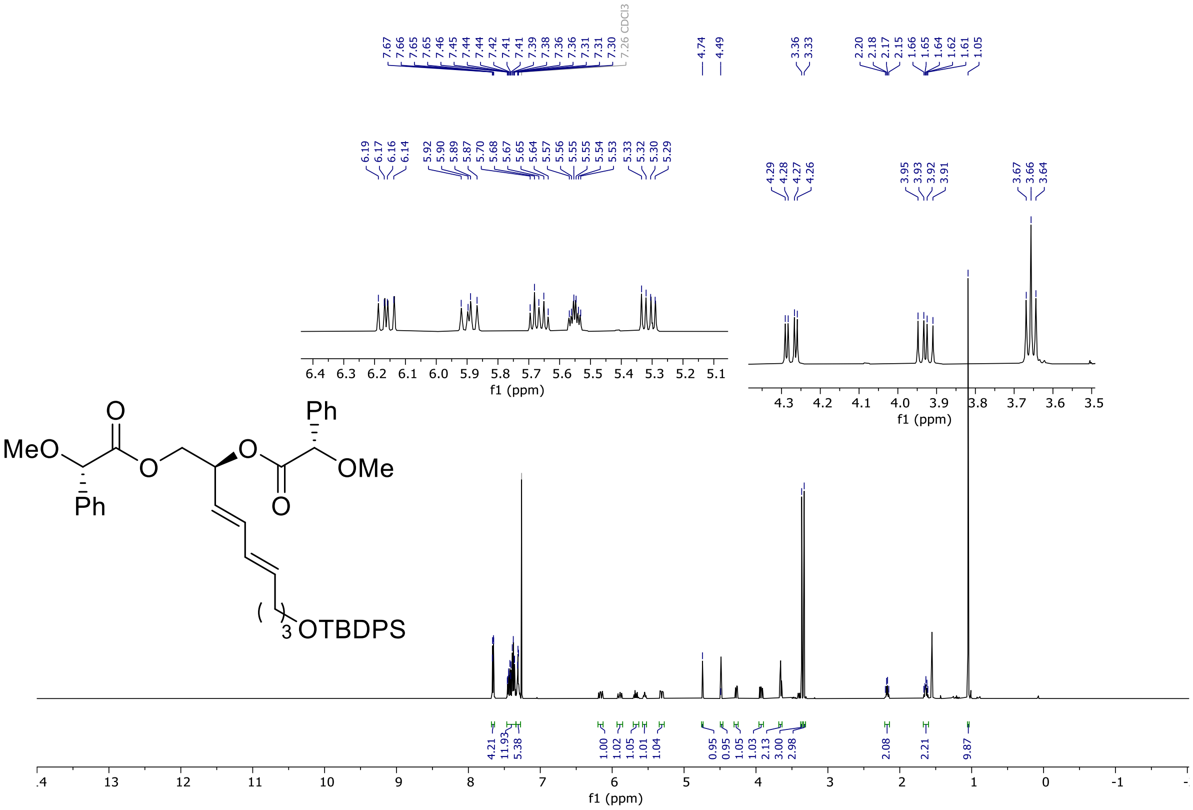

^{13}C NMR ($CDCl_3$, 125 MHz) **6d**

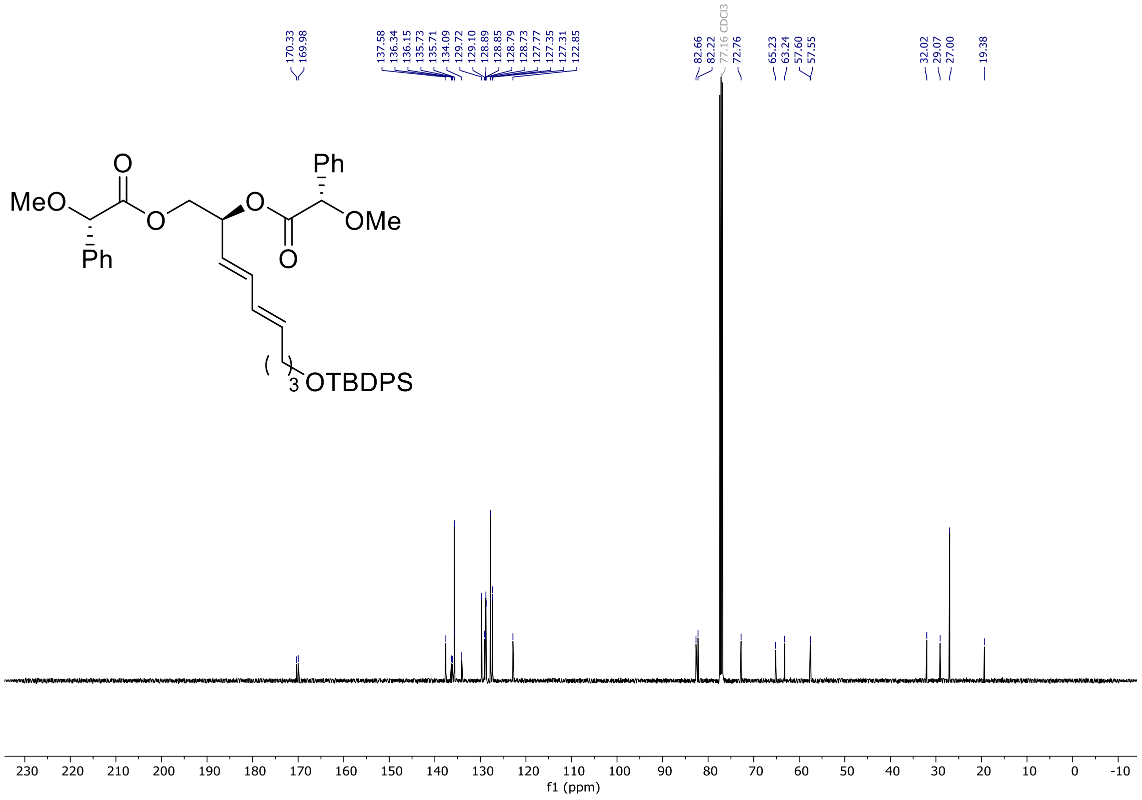

^{1}H NMR ($CDCl_3$, 400 MHz) **7d**

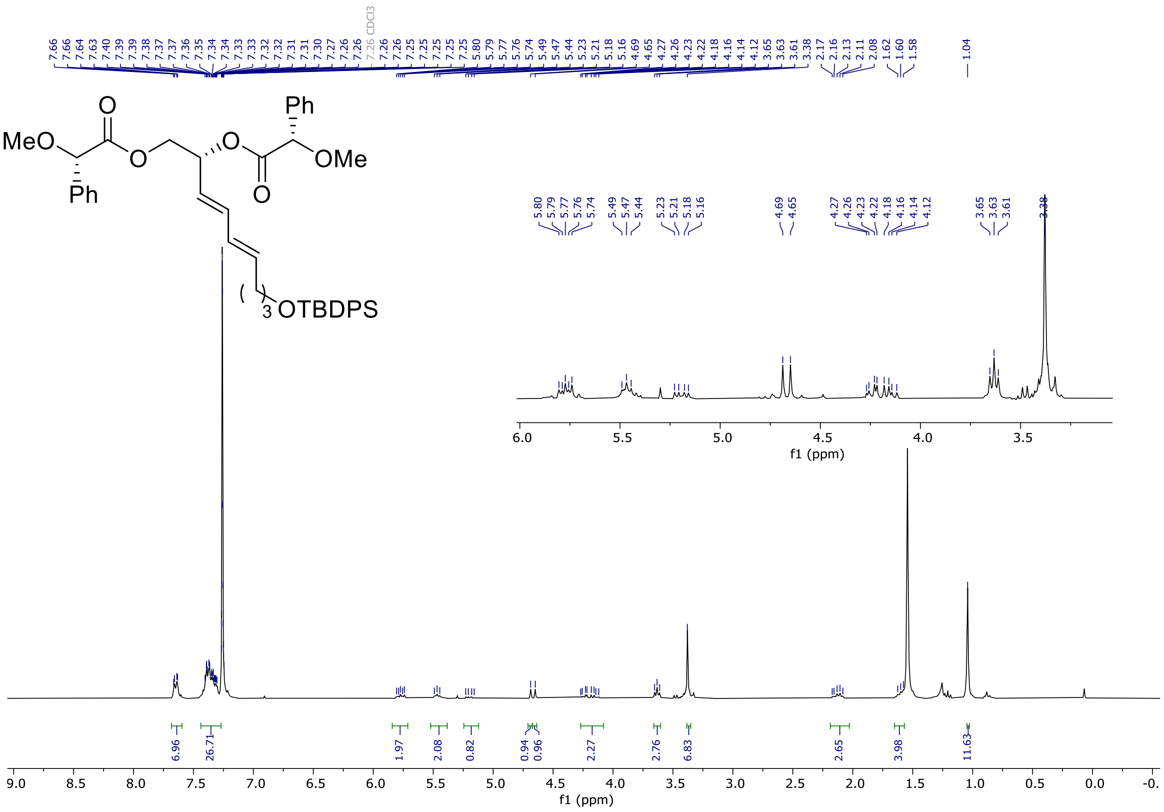

^{1}H NMR ($CDCl_3$, 500 MHz) **4e**

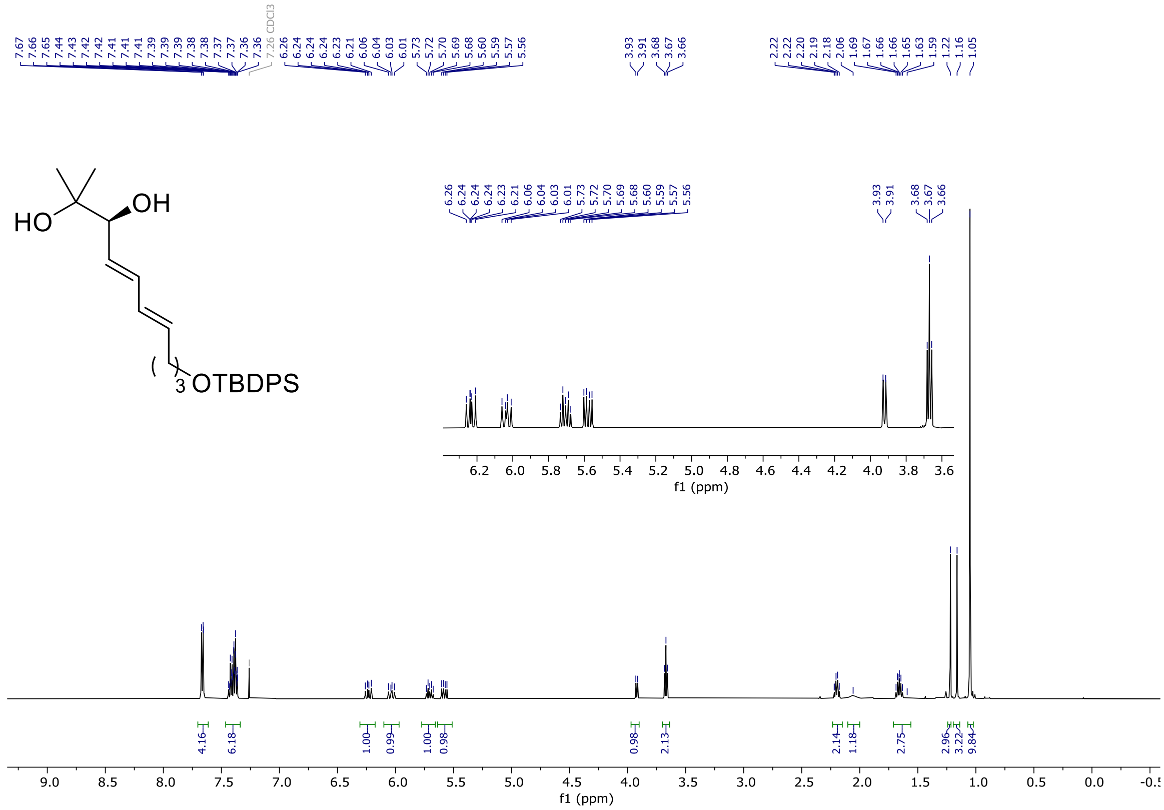

^{13}C NMR ($CDCl_3$, 125 MHz) **4e**

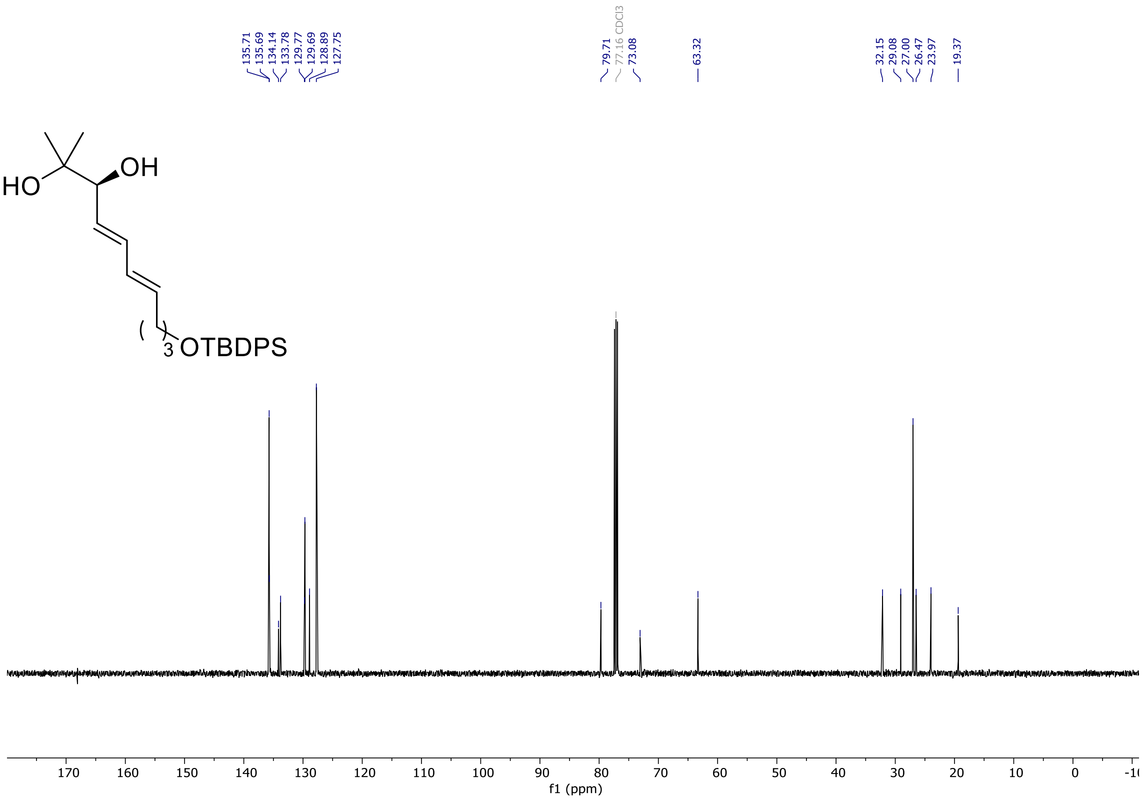

^{1}H NMR ($CDCl_3$, 500 MHz) **6e**

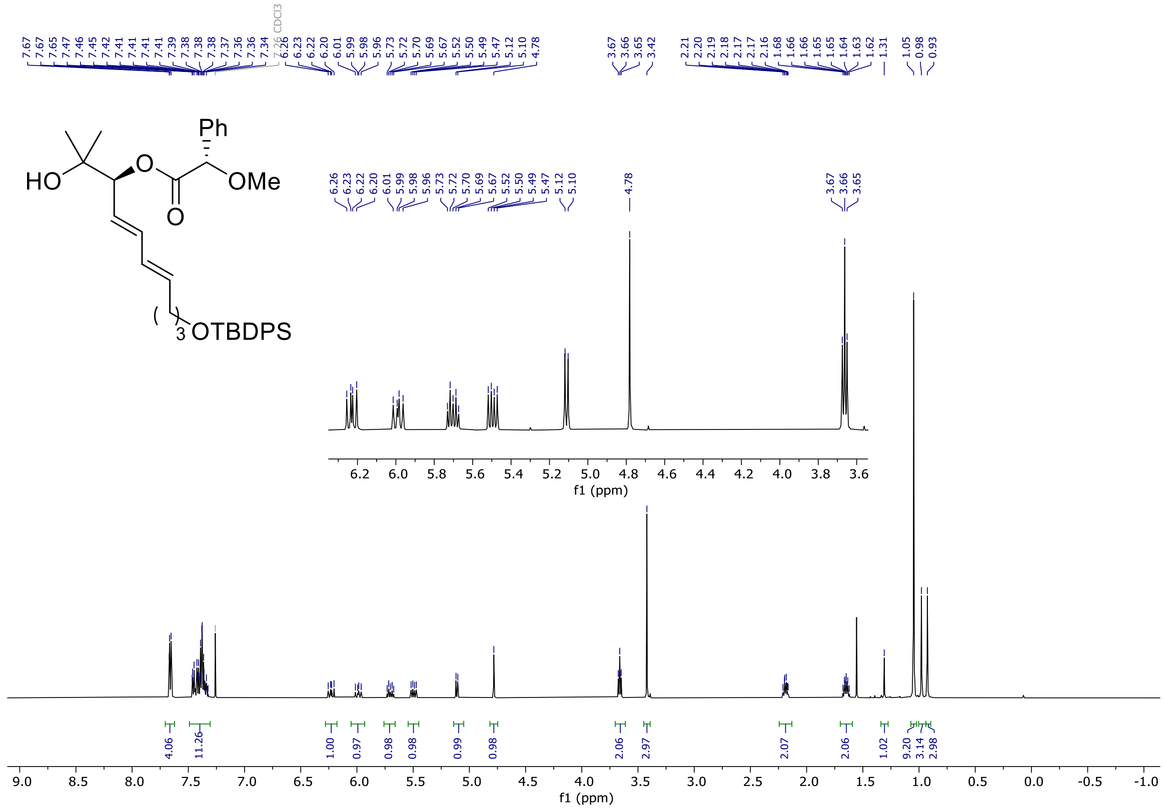

^{13}C NMR ($CDCl_3$, 125 MHz) **6e**

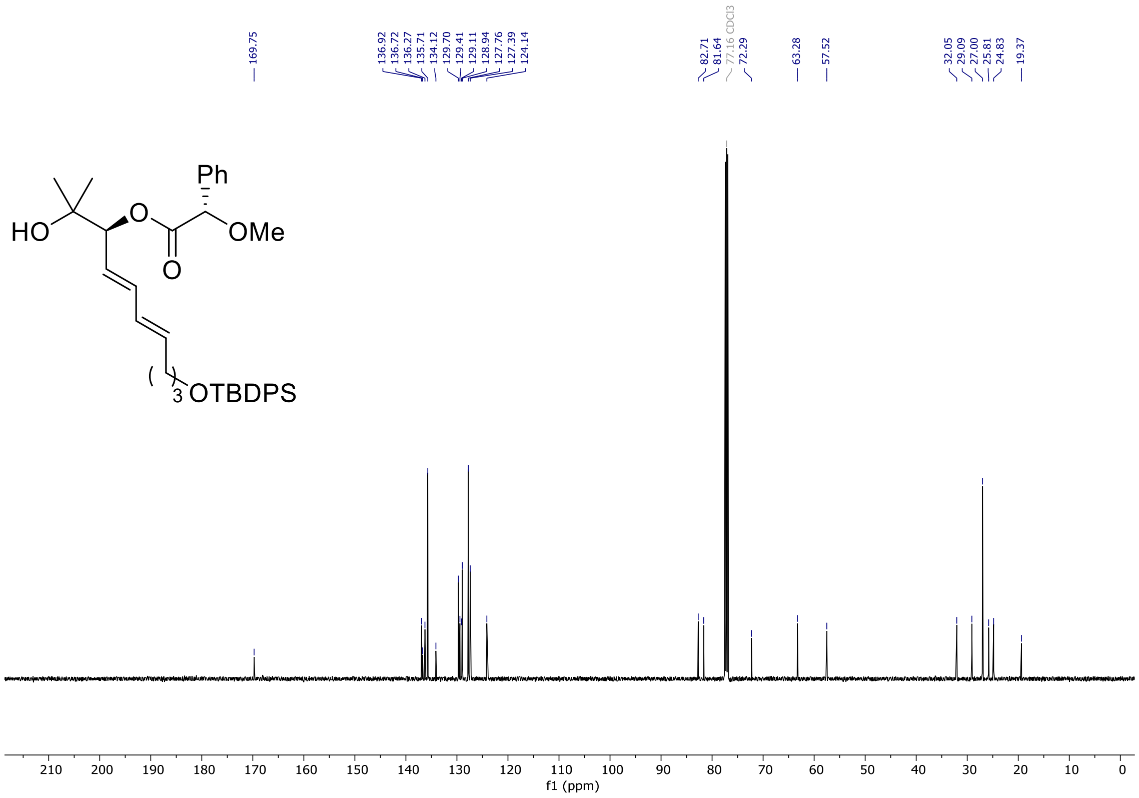

^{1}H NMR ($CDCl_3$, 300 MHz) **6e'**

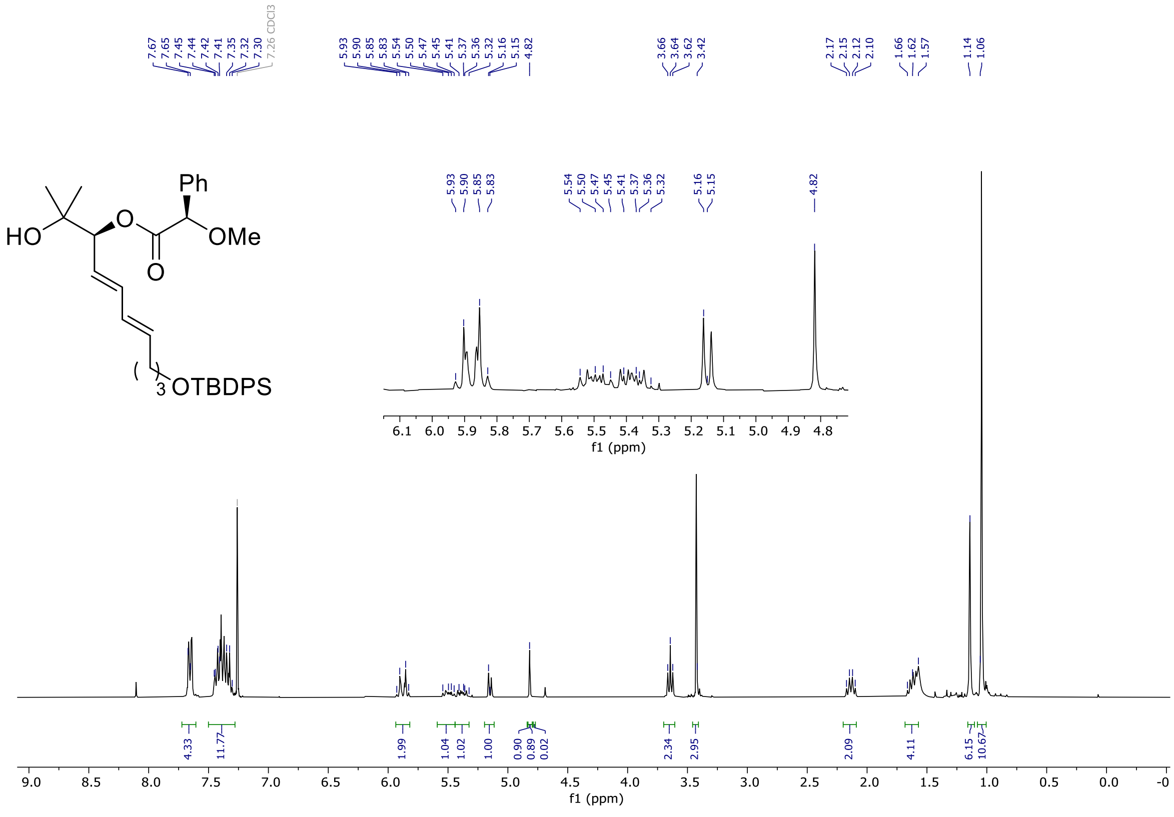

^{13}C NMR ($CDCl_3$, 125 MHz) **6e'**

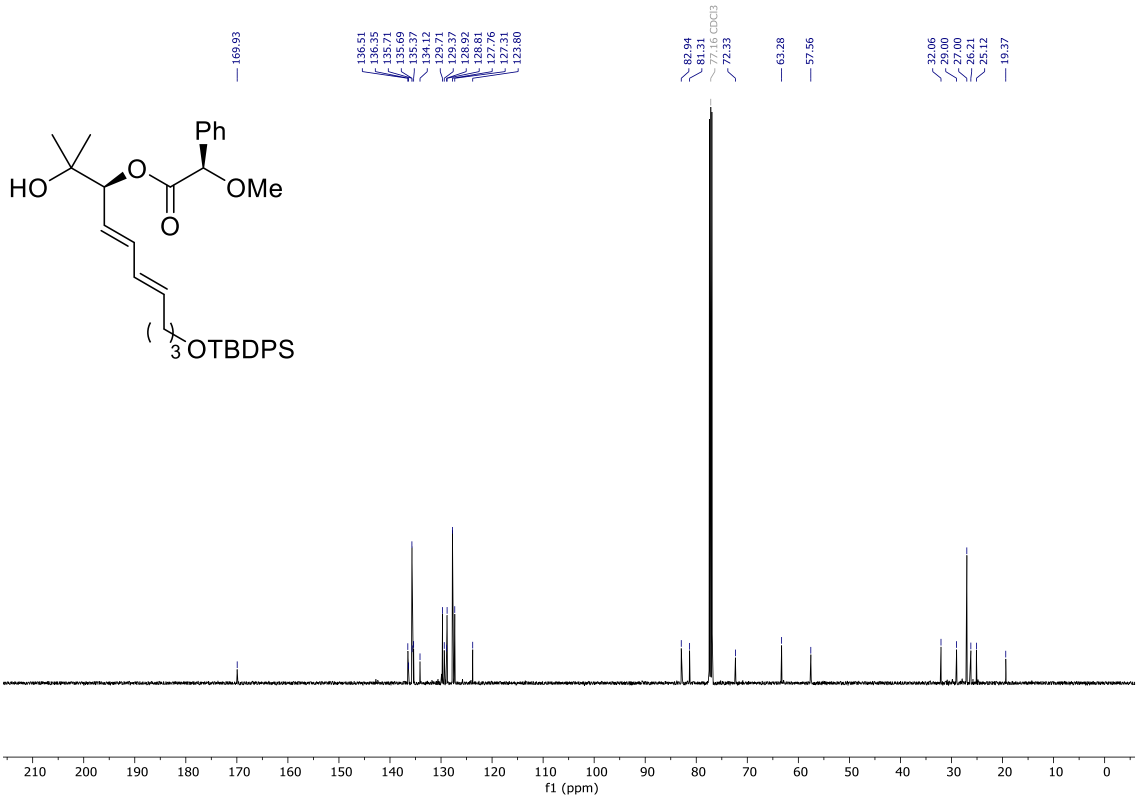

^{1}H NMR ($CDCl_3$, 400 MHz) **4f**

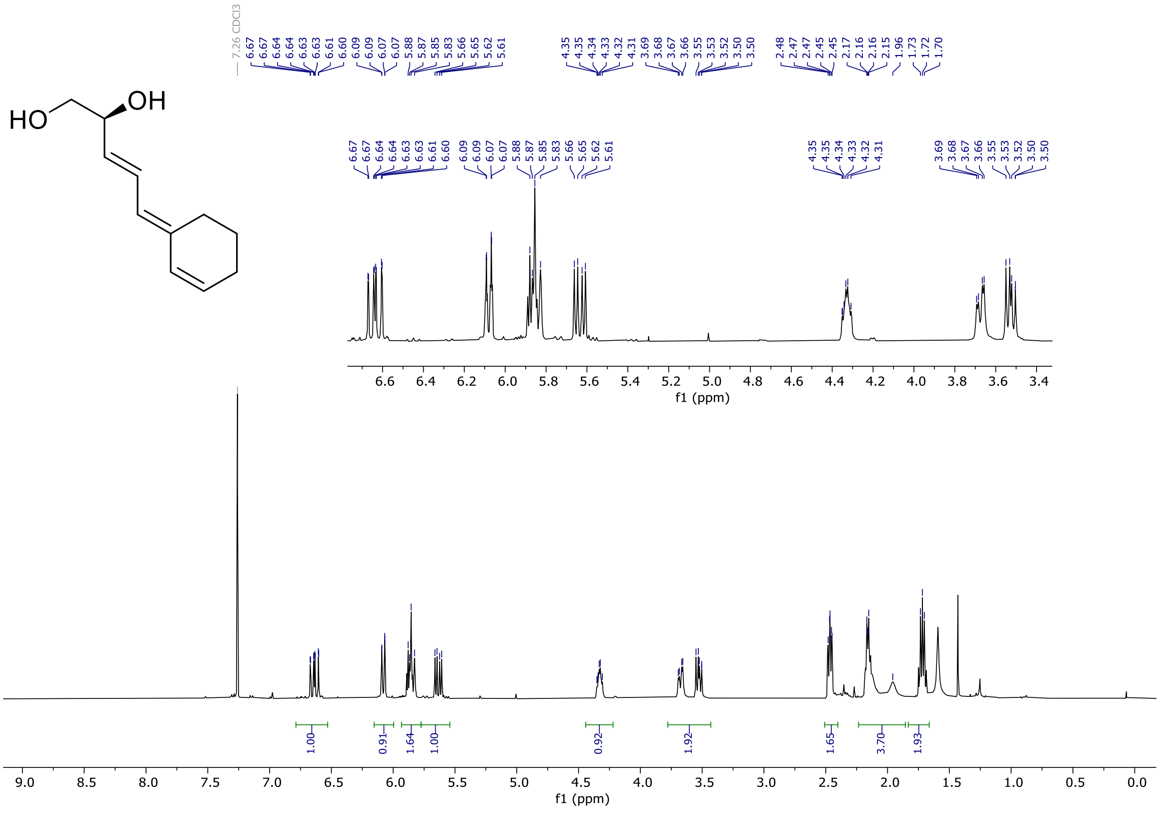

^{13}C NMR ($CDCl_3$, 100 MHz) **4f**

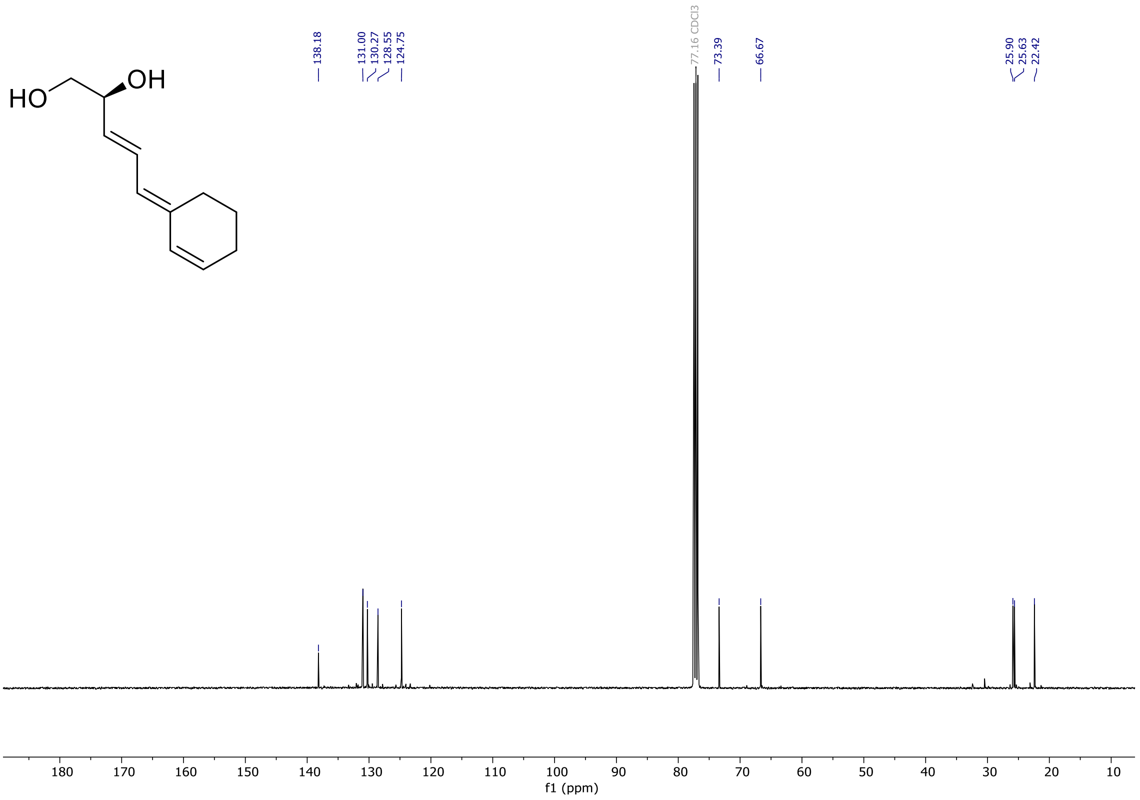

COSY (^1H, ^1H) **4f**

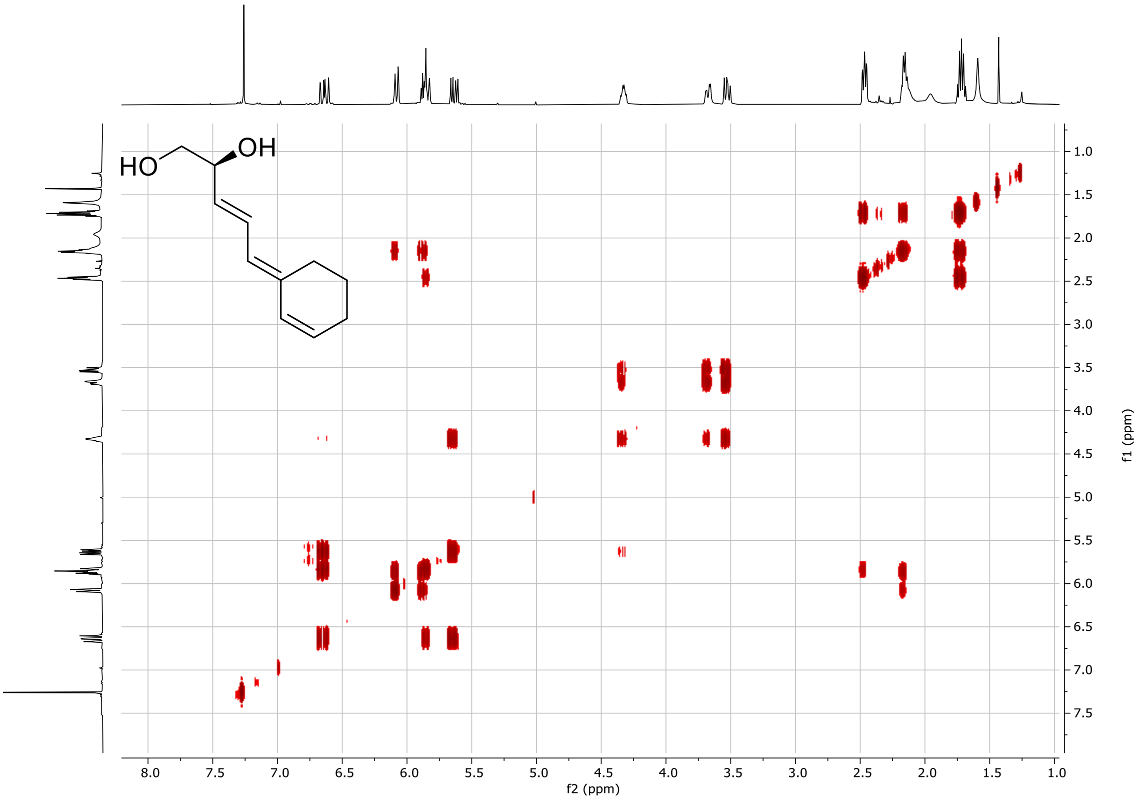

HSQC (^{1}H, ^{13}C) **4f**

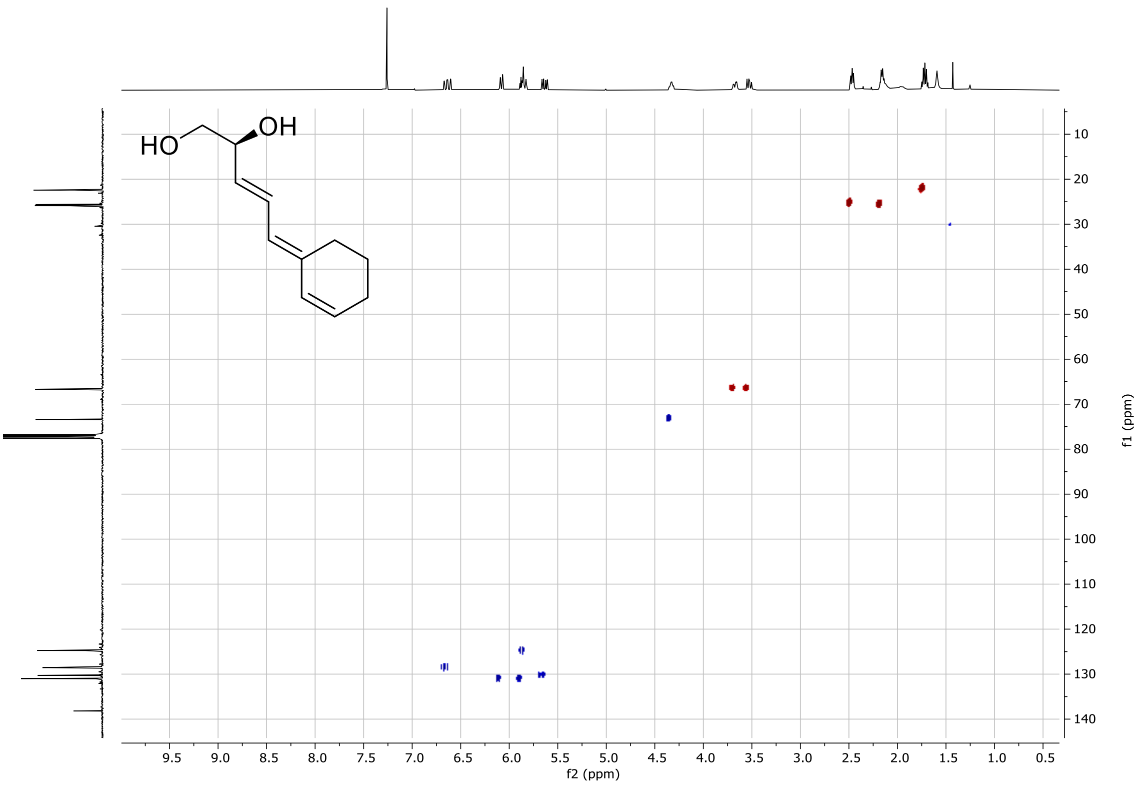

NOESY (^{1}H, ^{1}H) **4f**

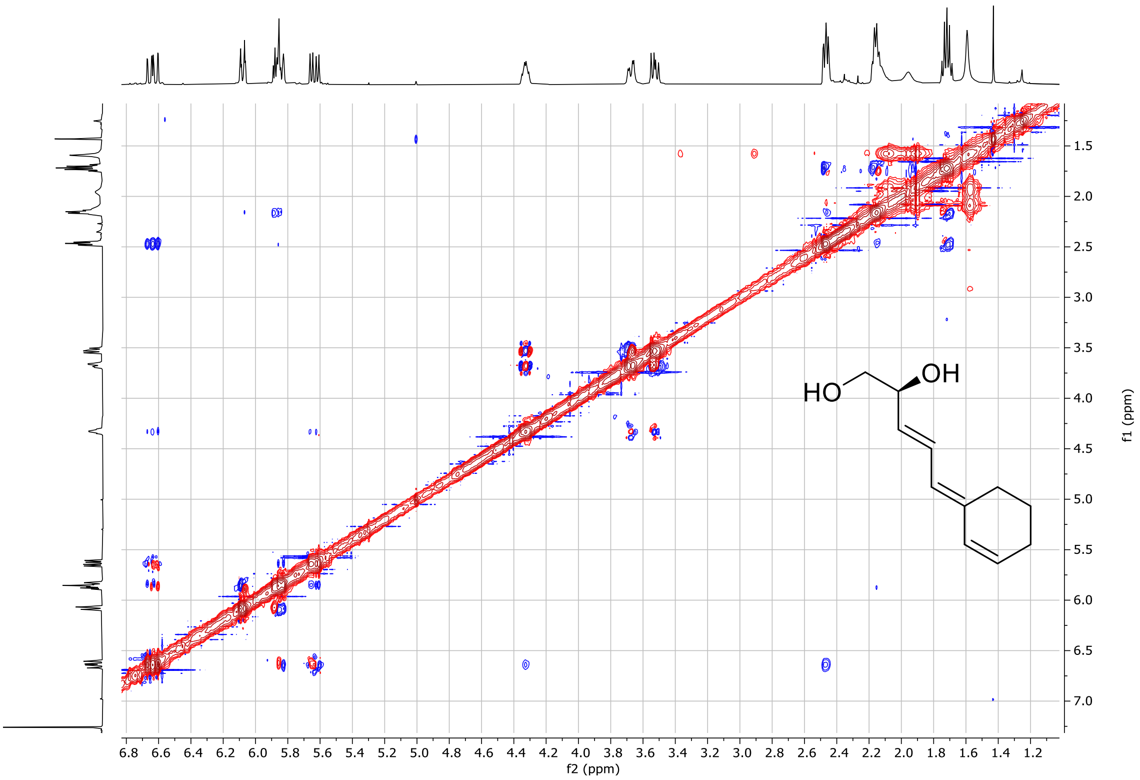

^{1}H NMR ($CDCl_3$, 400 MHz) **6f**

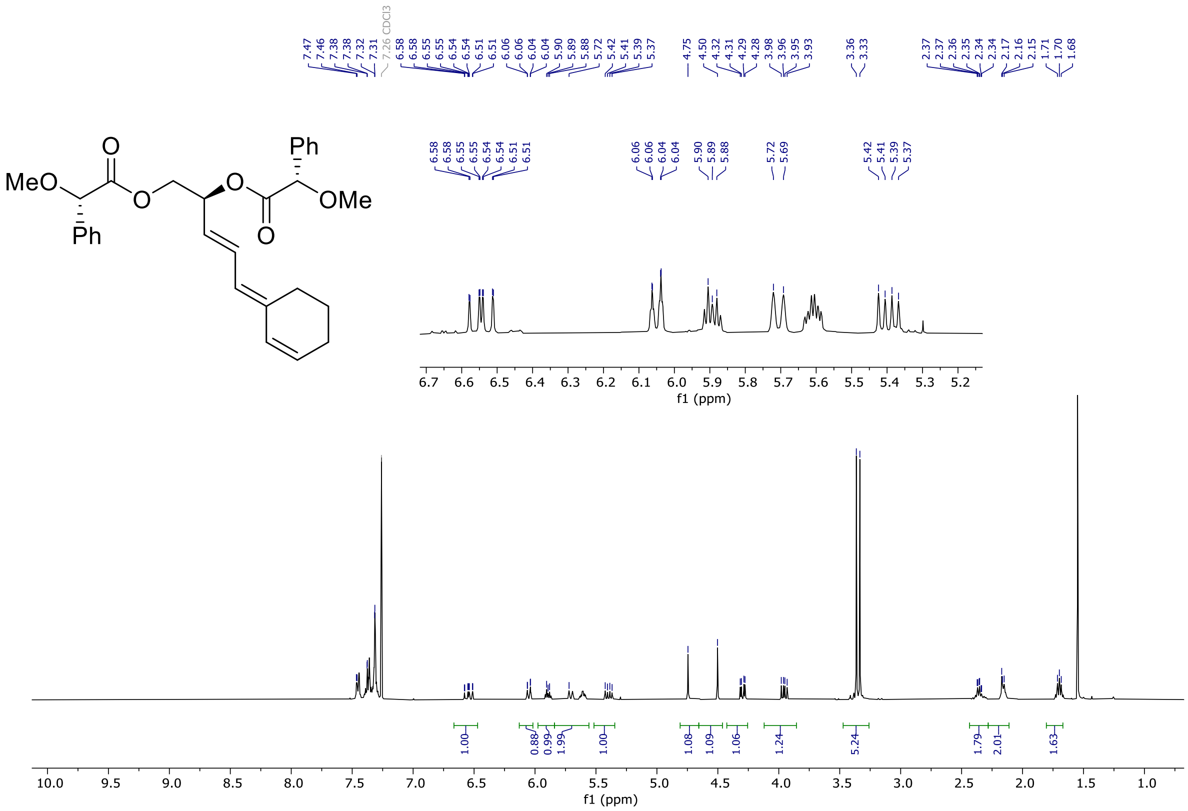

^{13}C NMR ($CDCl_3$, 100 MHz) **6f**

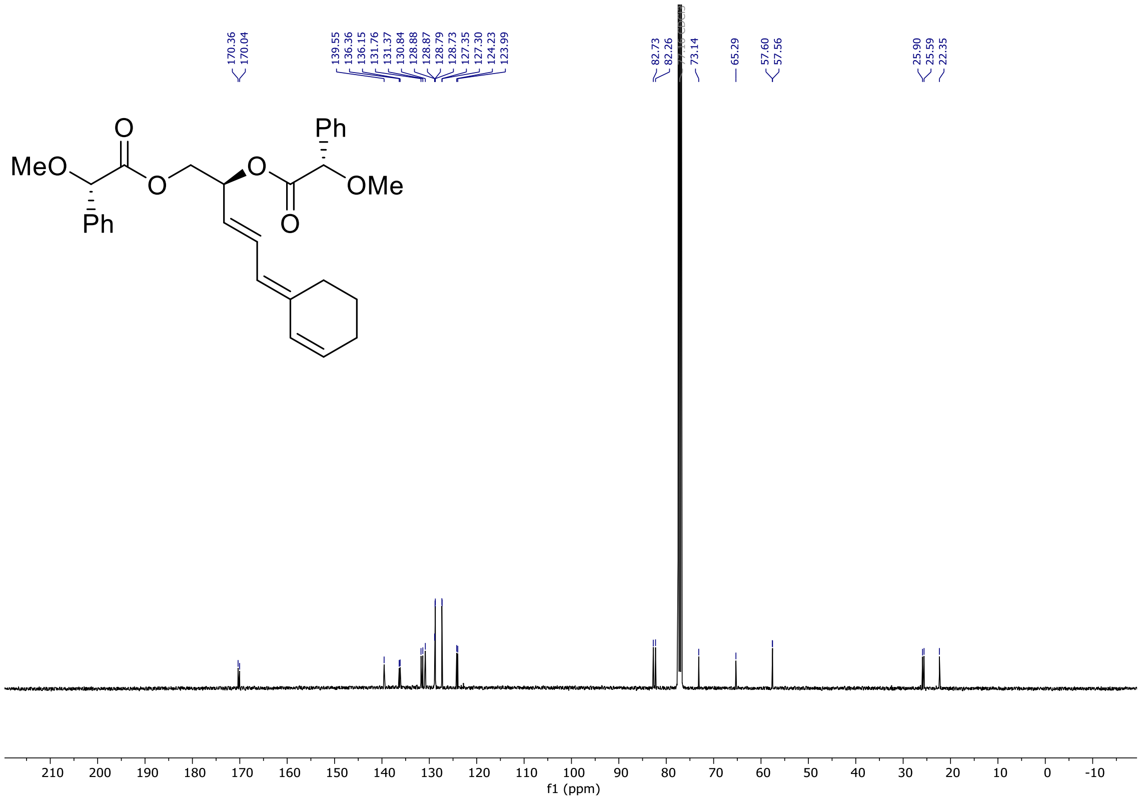

COSY (^{1}H, ^{1}H) **6f**

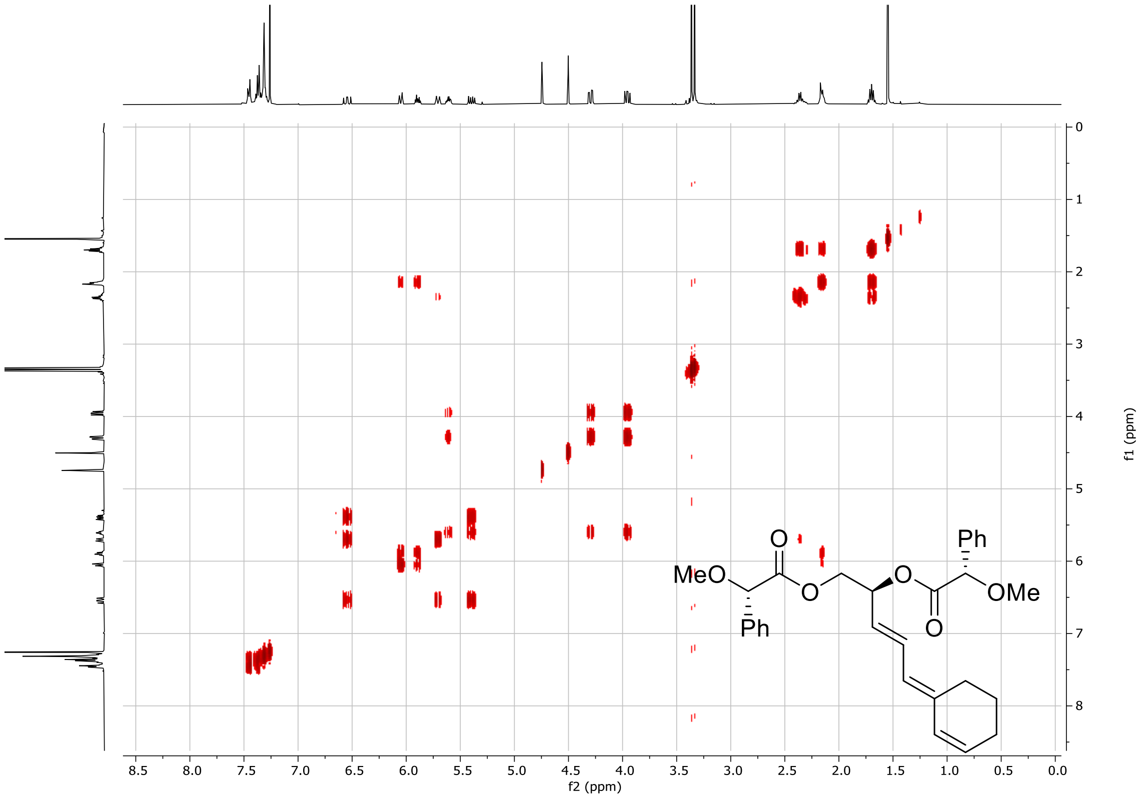

HSQC (^{1}H, ^{13}C) **6f**

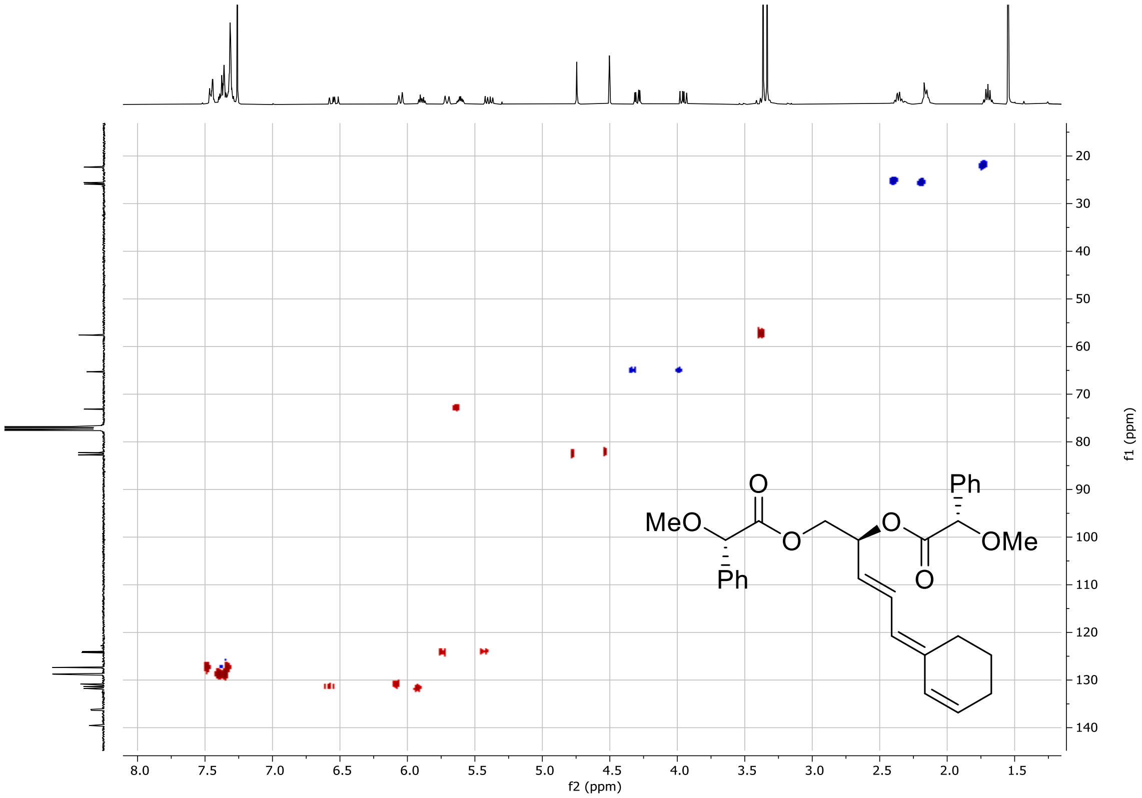

^{1}H NMR ($CDCl_3$, 400 MHz) **7f**

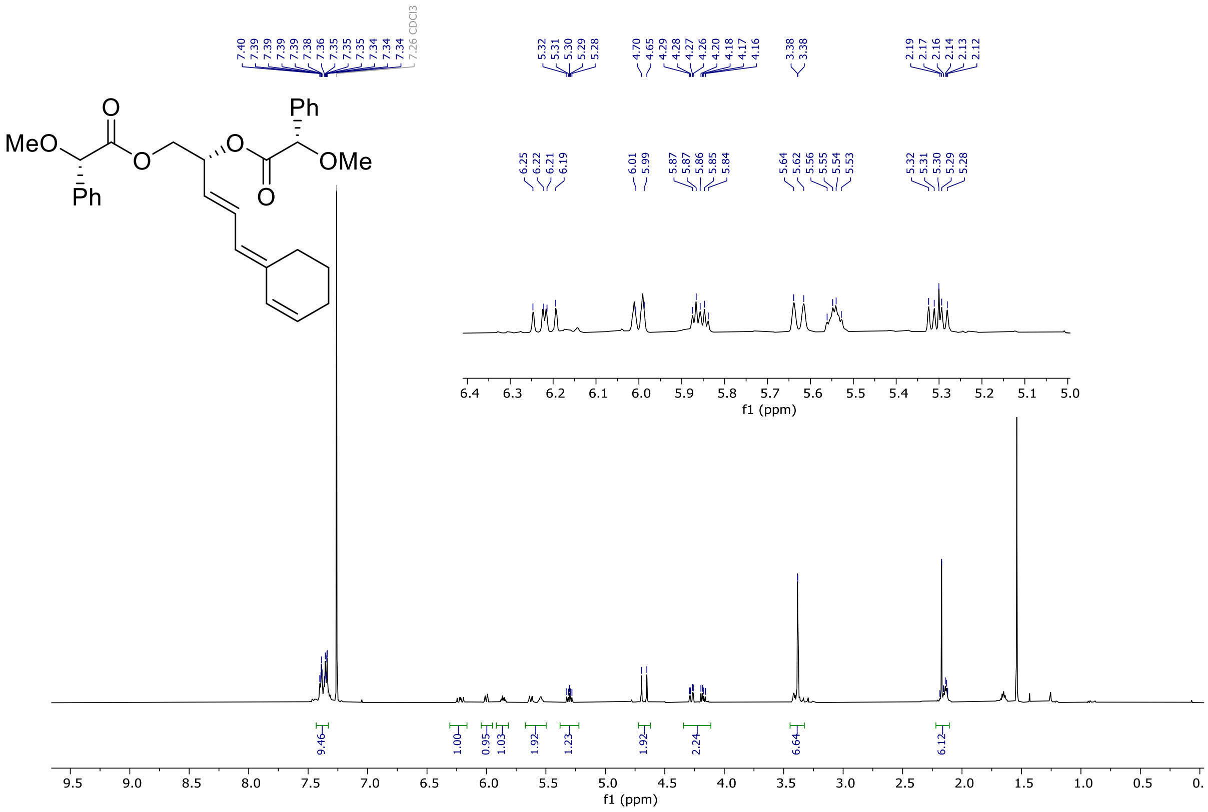

^{13}C NMR ($CDCl_3$, 100 MHz) **7f**

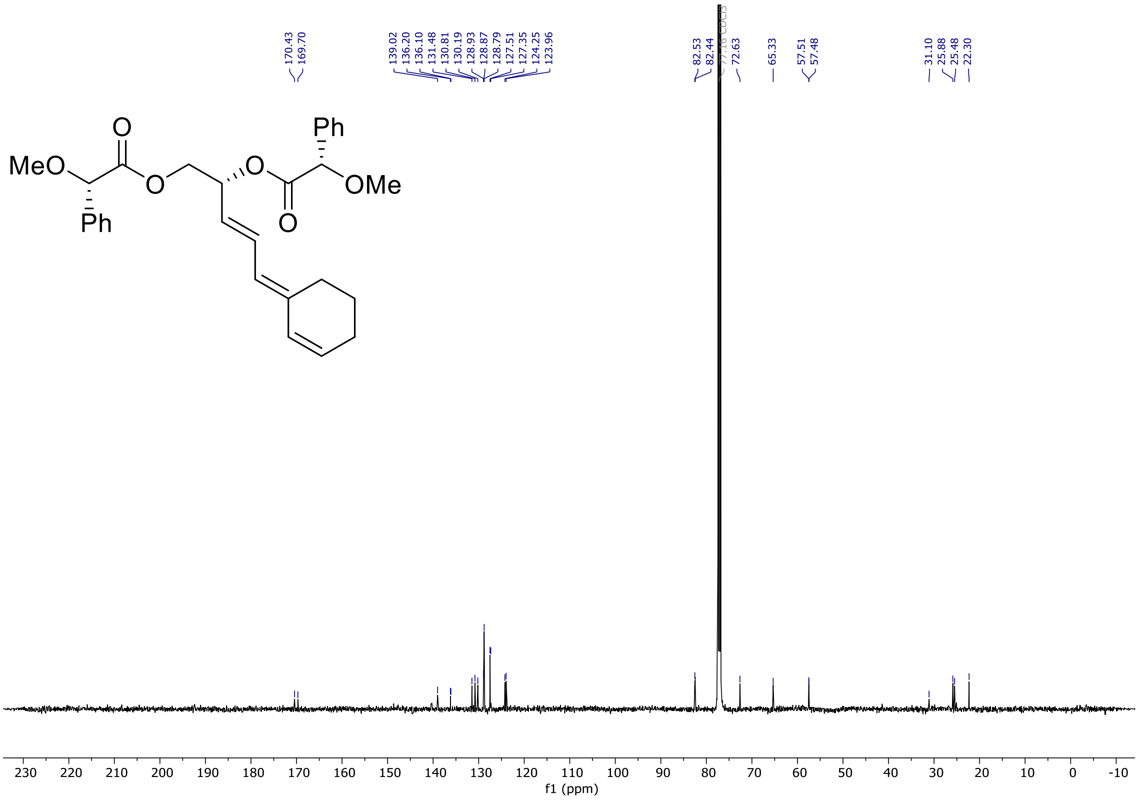

COSY (^{1}H, ^{1}H) **7f**

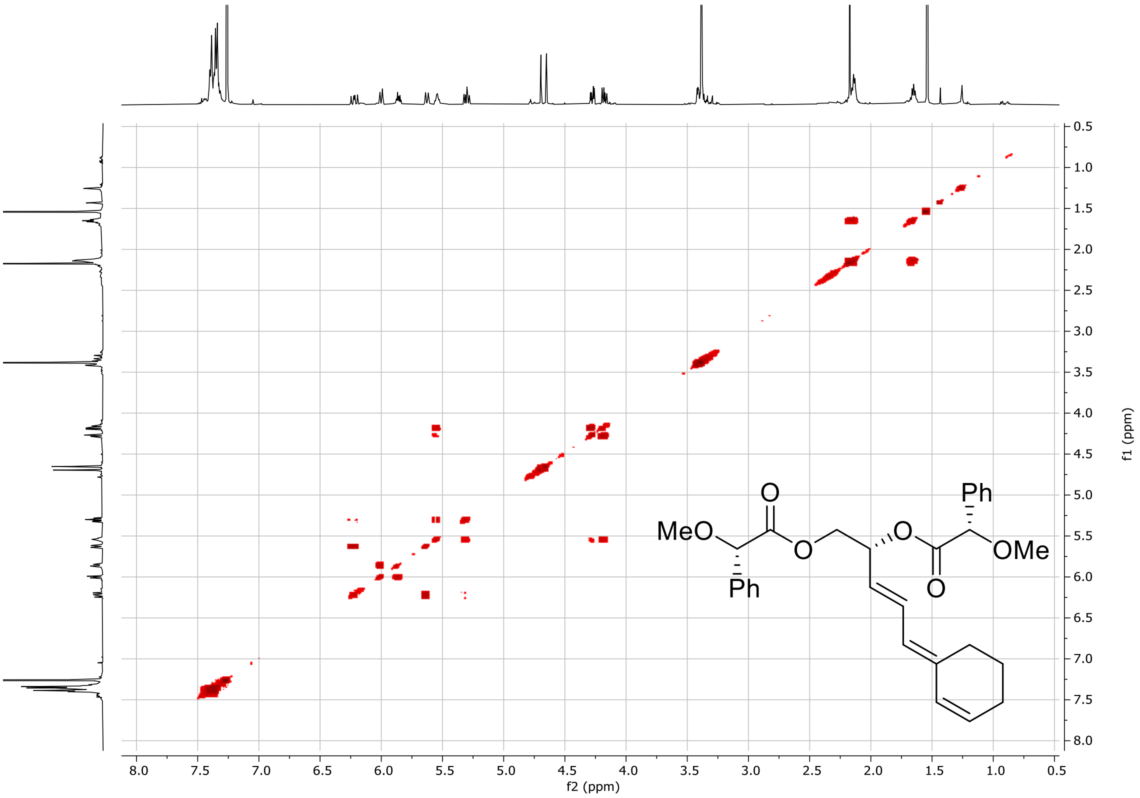

HSQC (^{1}H, ^{13}C) **7f**

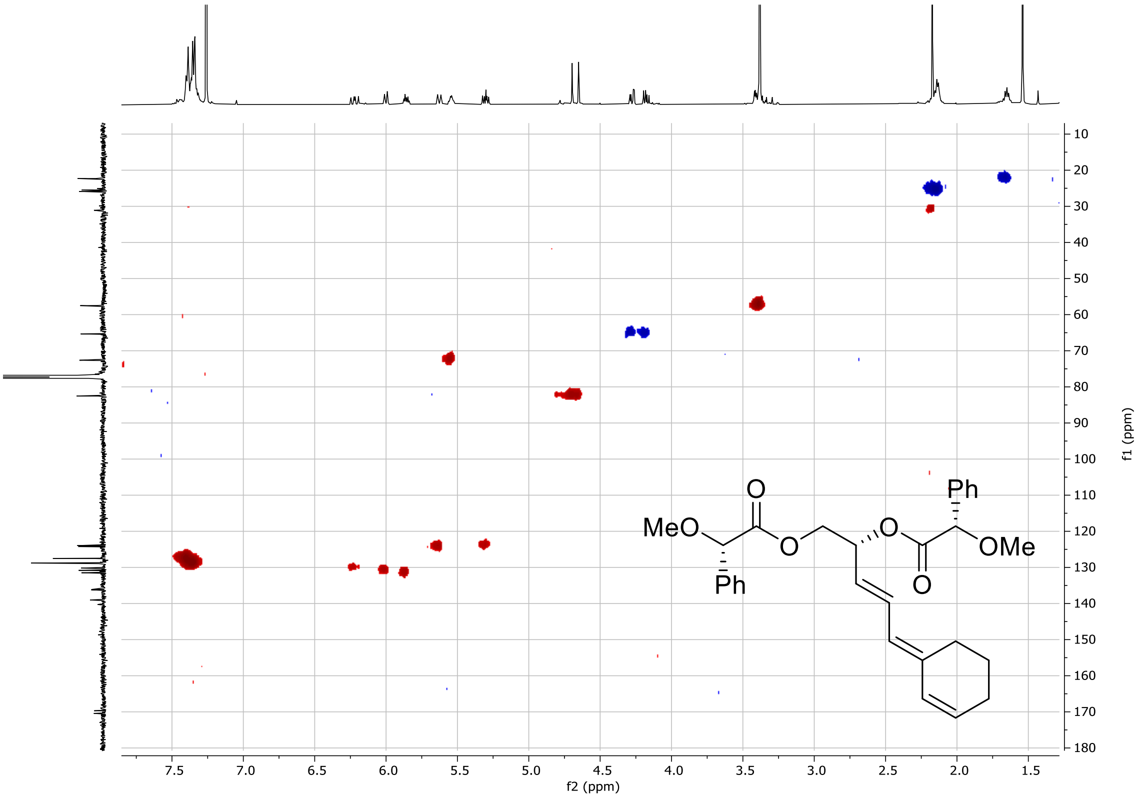

^{1}H NMR ($CDCl_3$, 400 MHz) **4g**

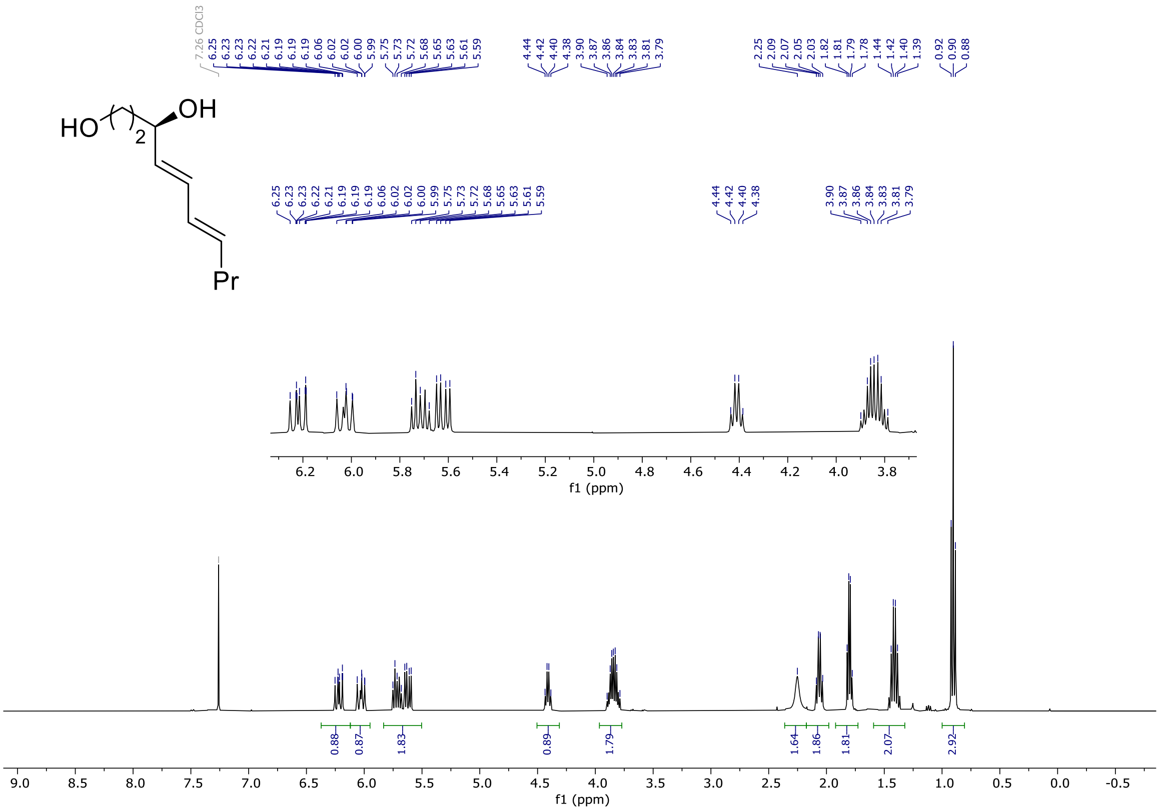

^{13}C NMR ($CDCl_3$, 100 MHz) **4g**

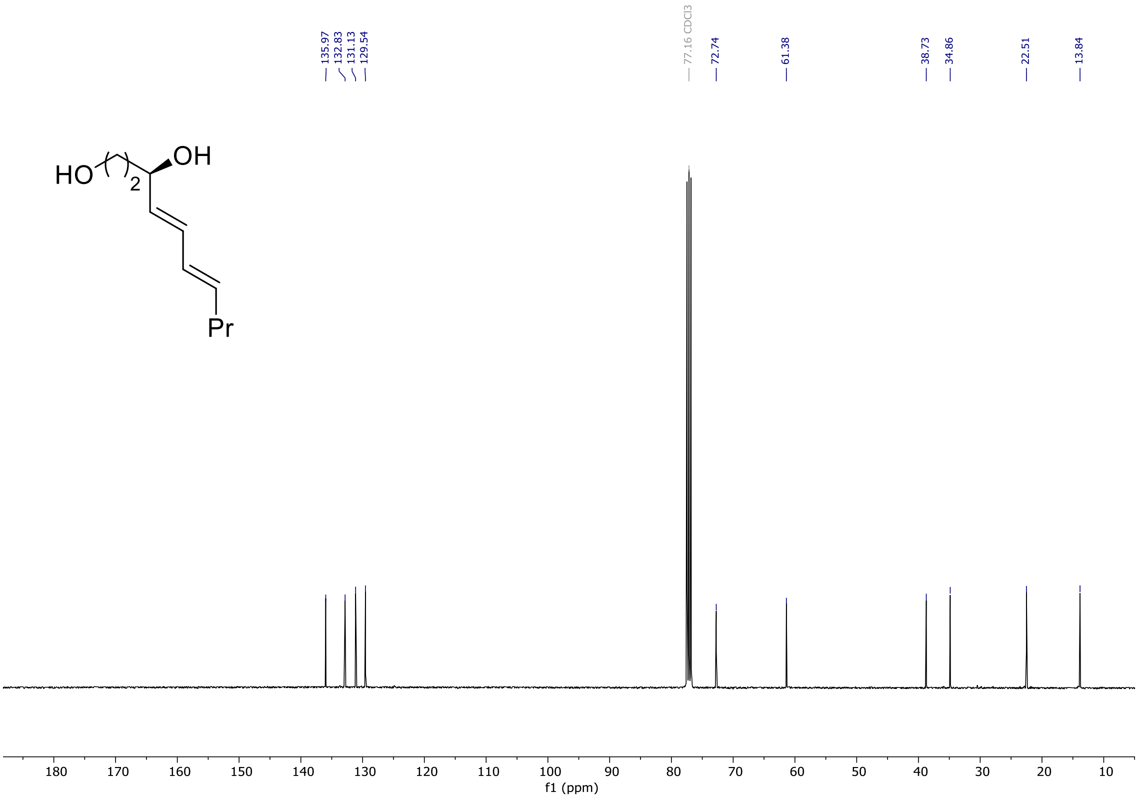

COSY (^{1}H, ^{1}H) **4g**

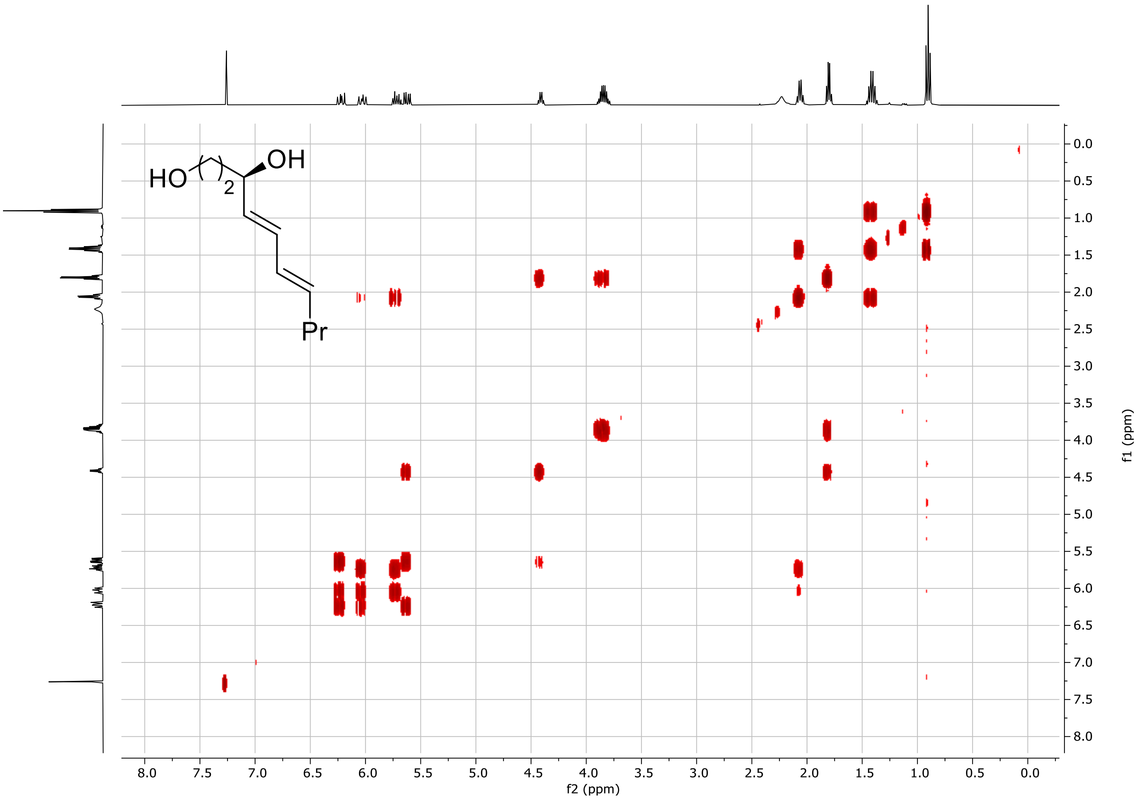

HSQC (^{1}H, ^{13}C) **4g**

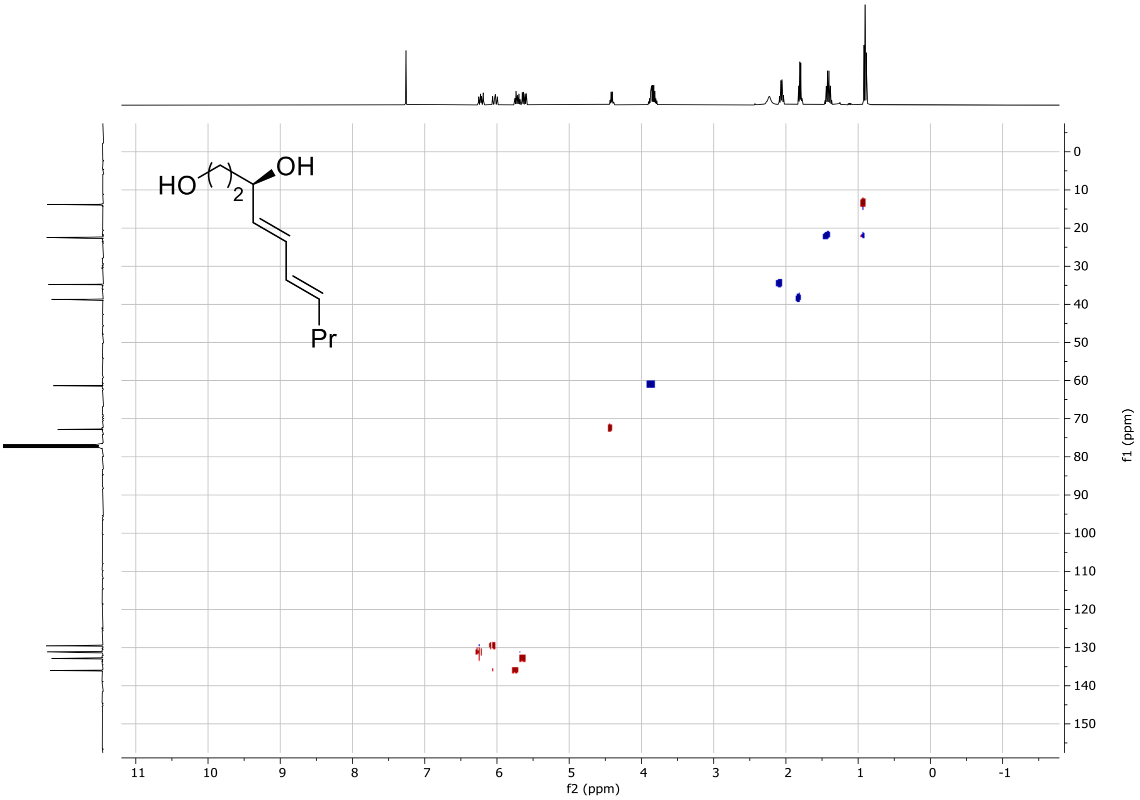

^{1}H NMR ($CDCl_3$, 400 MHz) **5g**

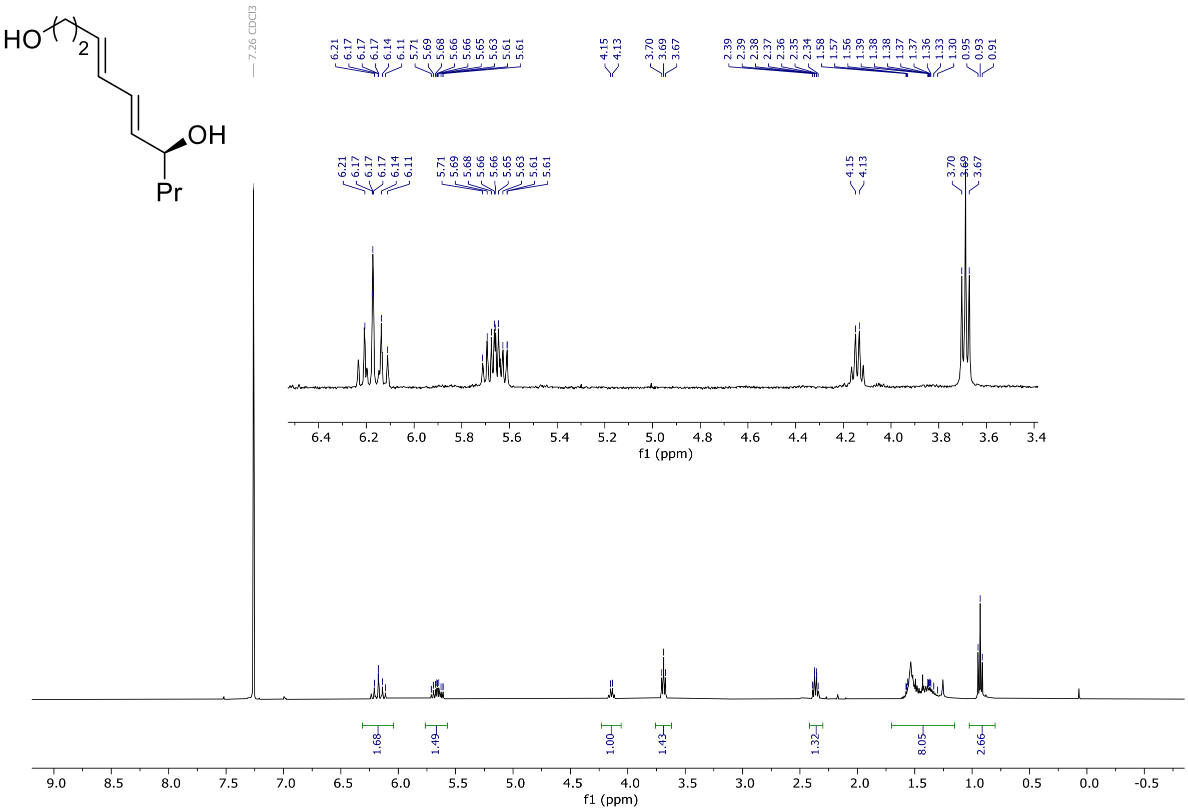

^{13}C NMR ($CDCl_3$, 125 MHz) **5g**

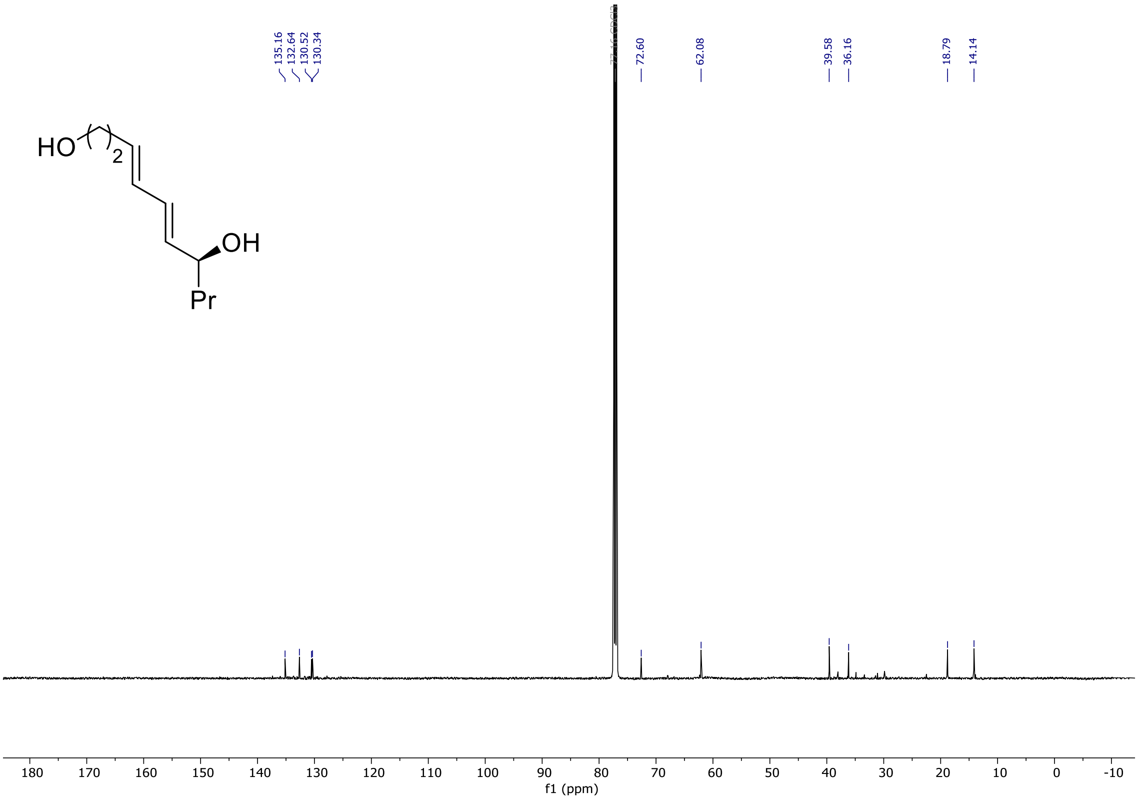

COSY (^1H, ^1H) **5g**

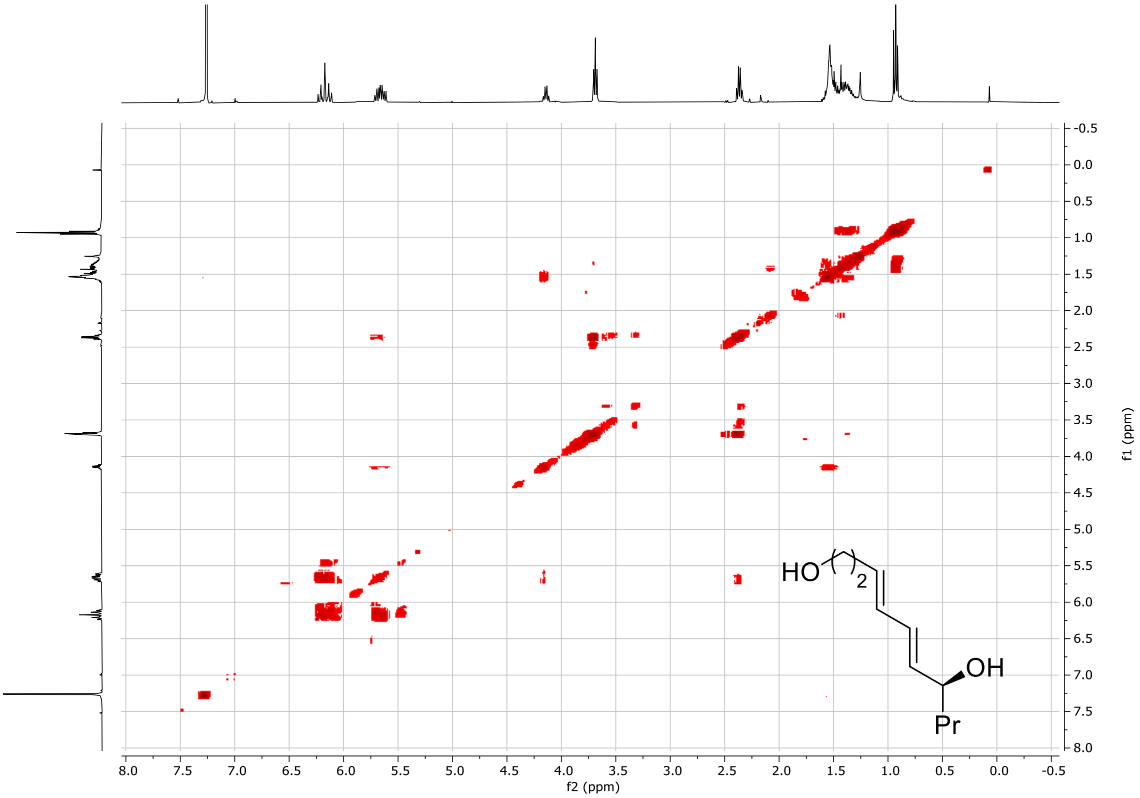

HSQC (^1H, ^{13}C) **5g**

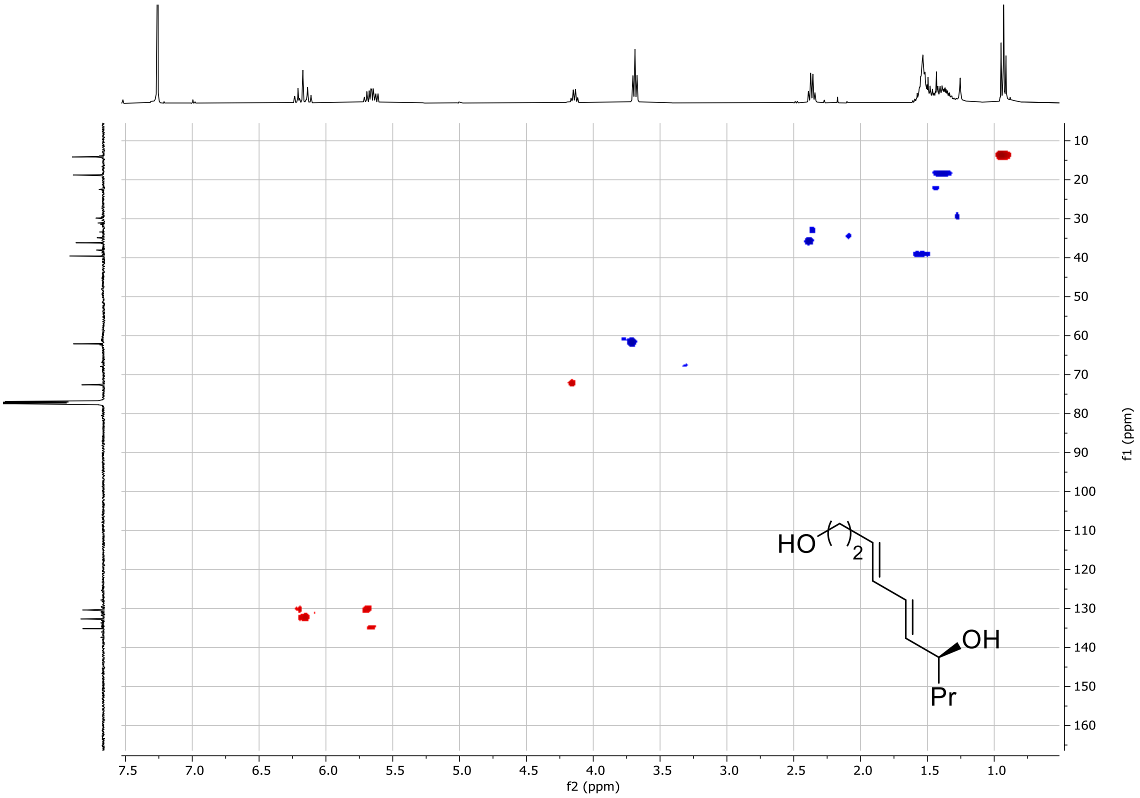

^{1}H NMR ($CDCl_3$, 400 MHz) **6g**

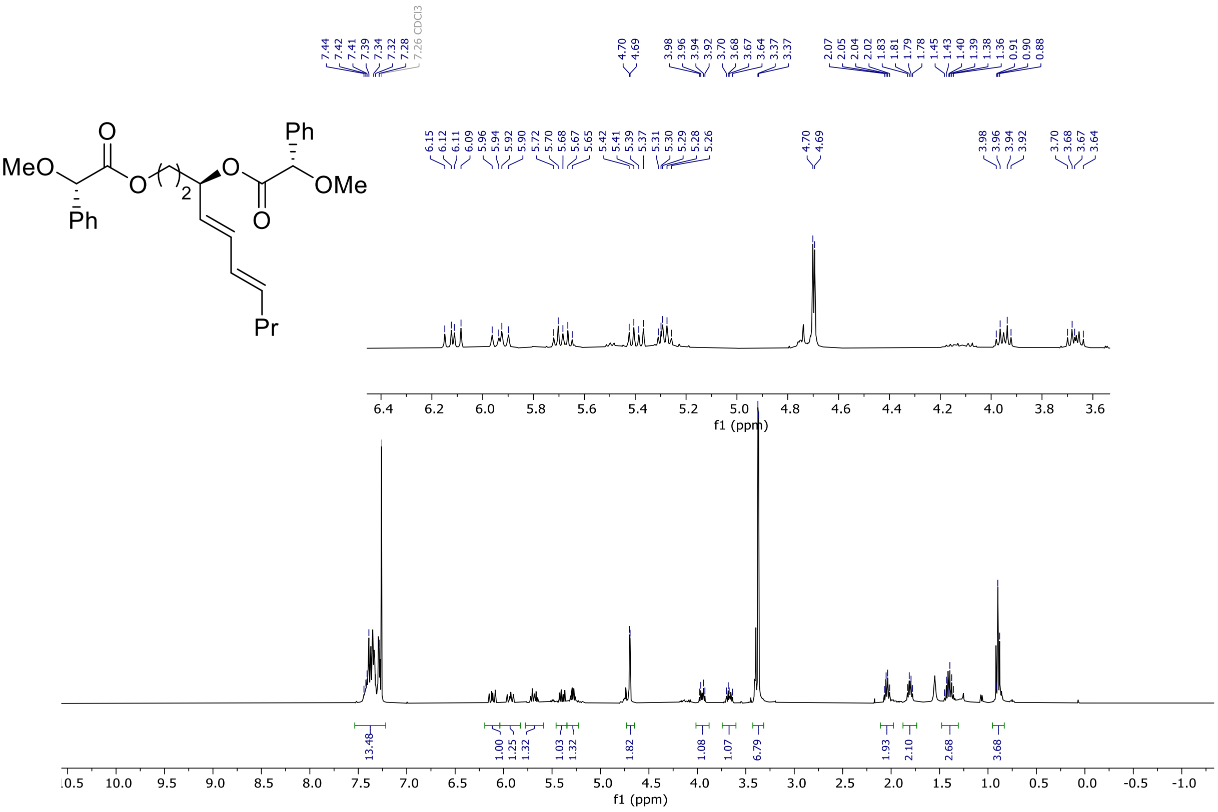

COSY (^{1}H, ^{1}H) **6g**

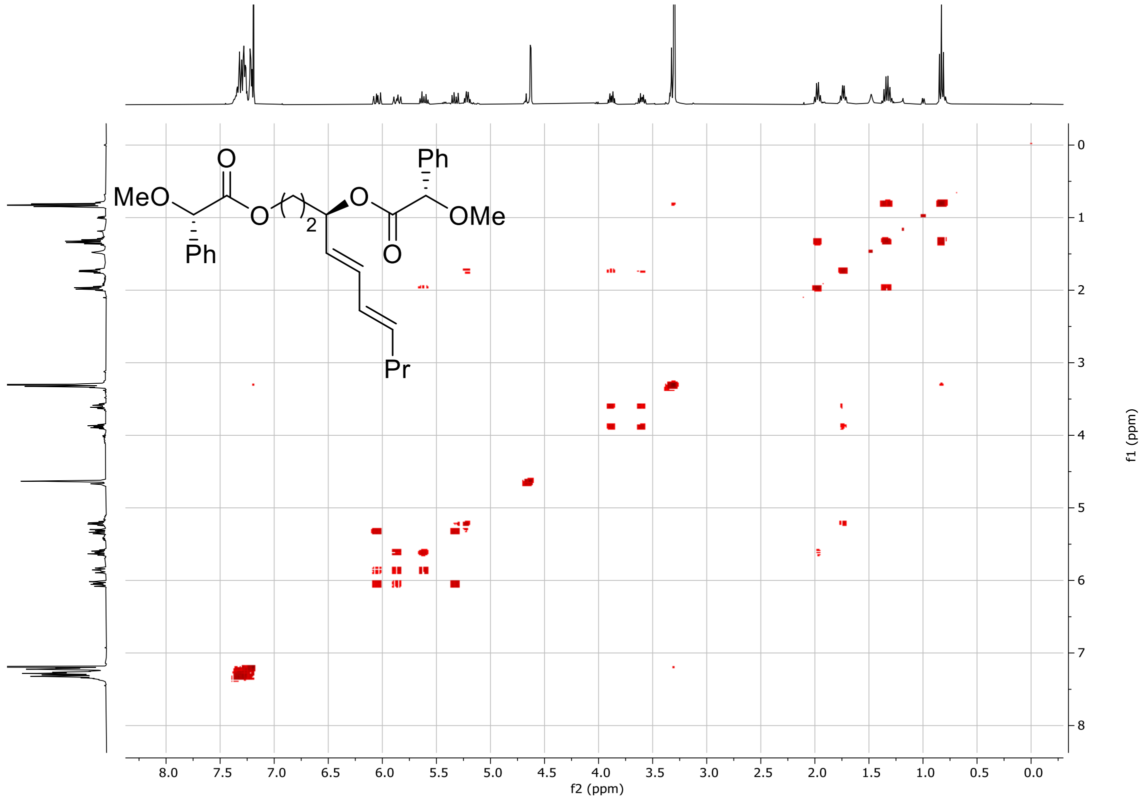

HSQC (^{1}H, ^{13}C) **6g**

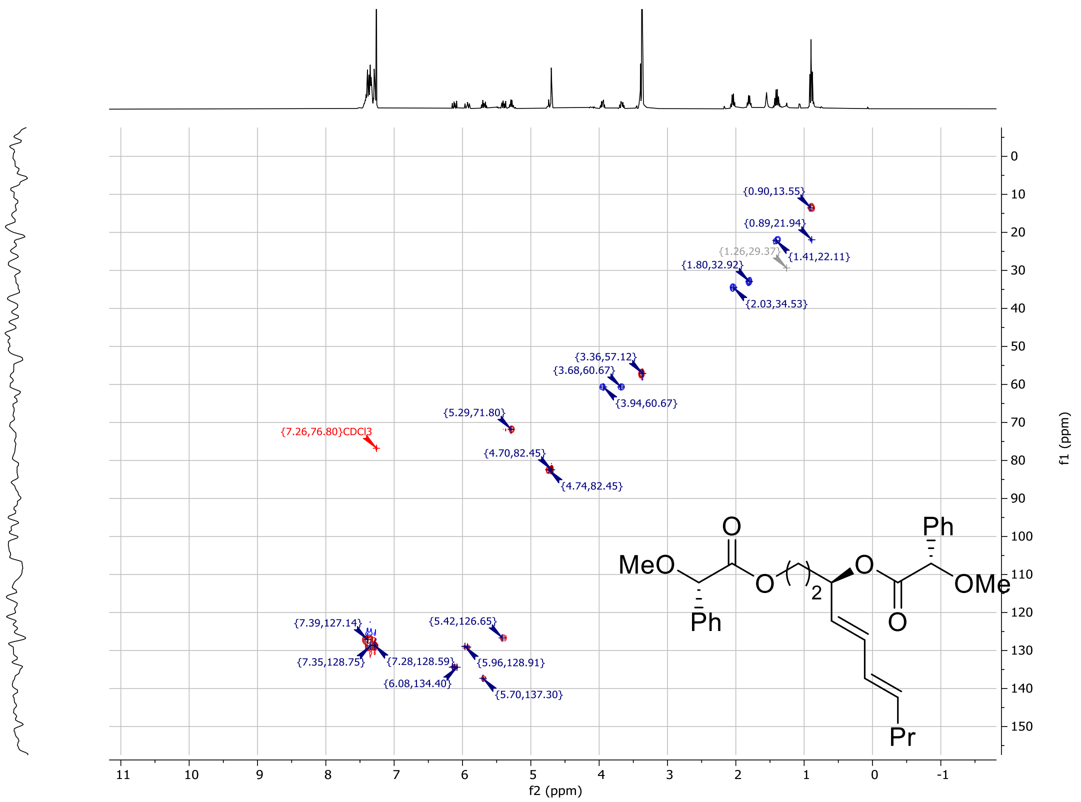

^{1}H NMR ($CDCl_3$, 400 MHz) **6g'**

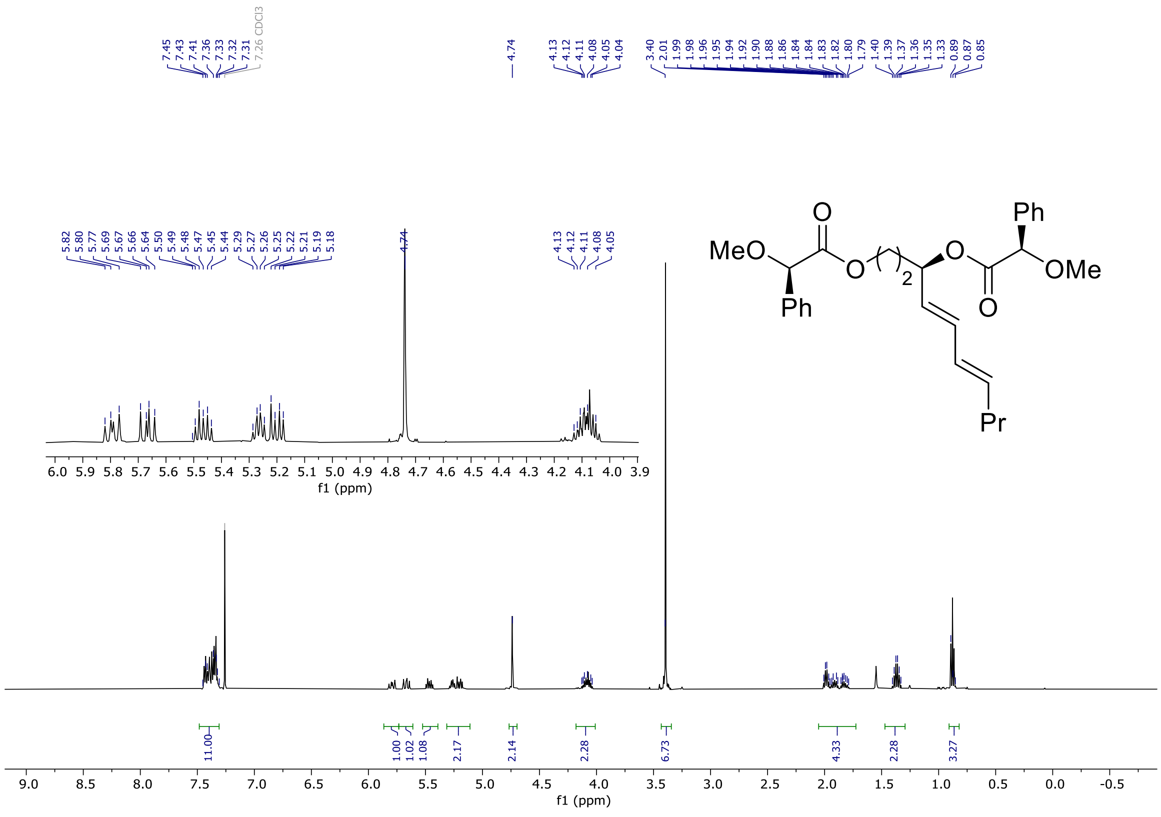

^{13}C NMR ($CDCl_3$, 100 MHz) **6g’**

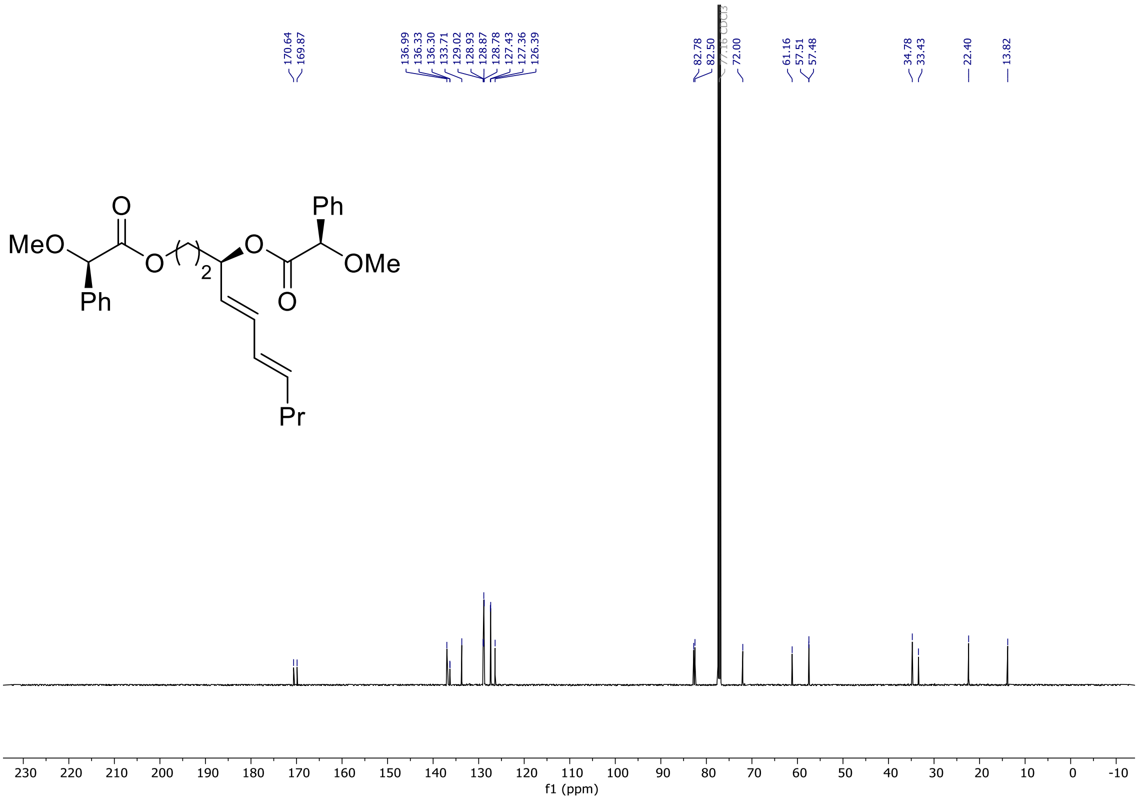

COSY (^1H, ^1H) **6g’**

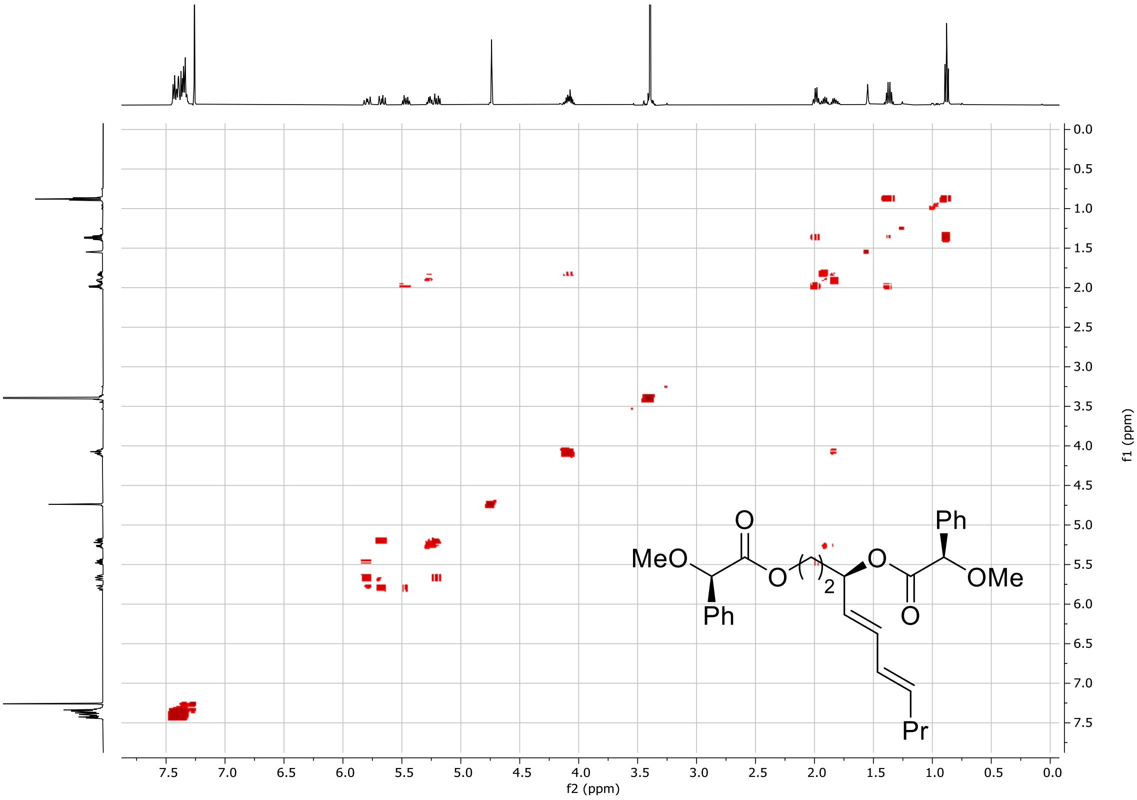

HSQC (^{1}H, ^{13}C) **6g’**

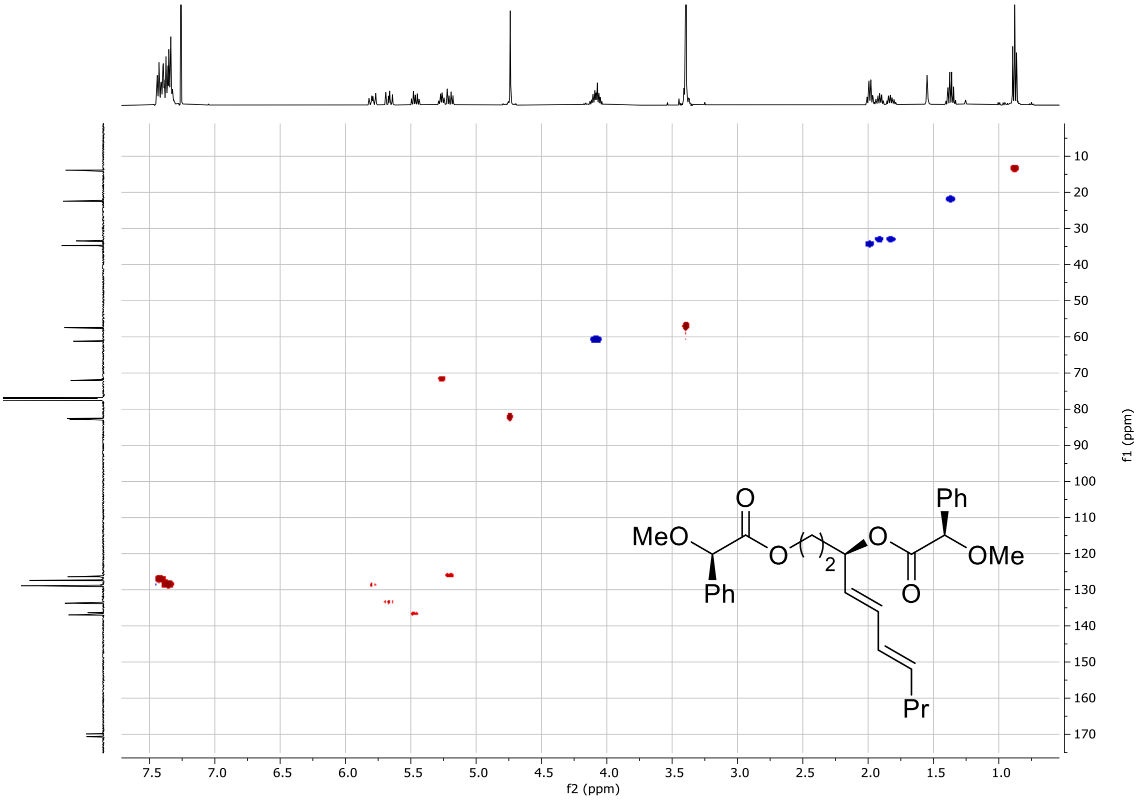

^{1}H NMR ($CDCl_3$, 300 MHz) **4h**

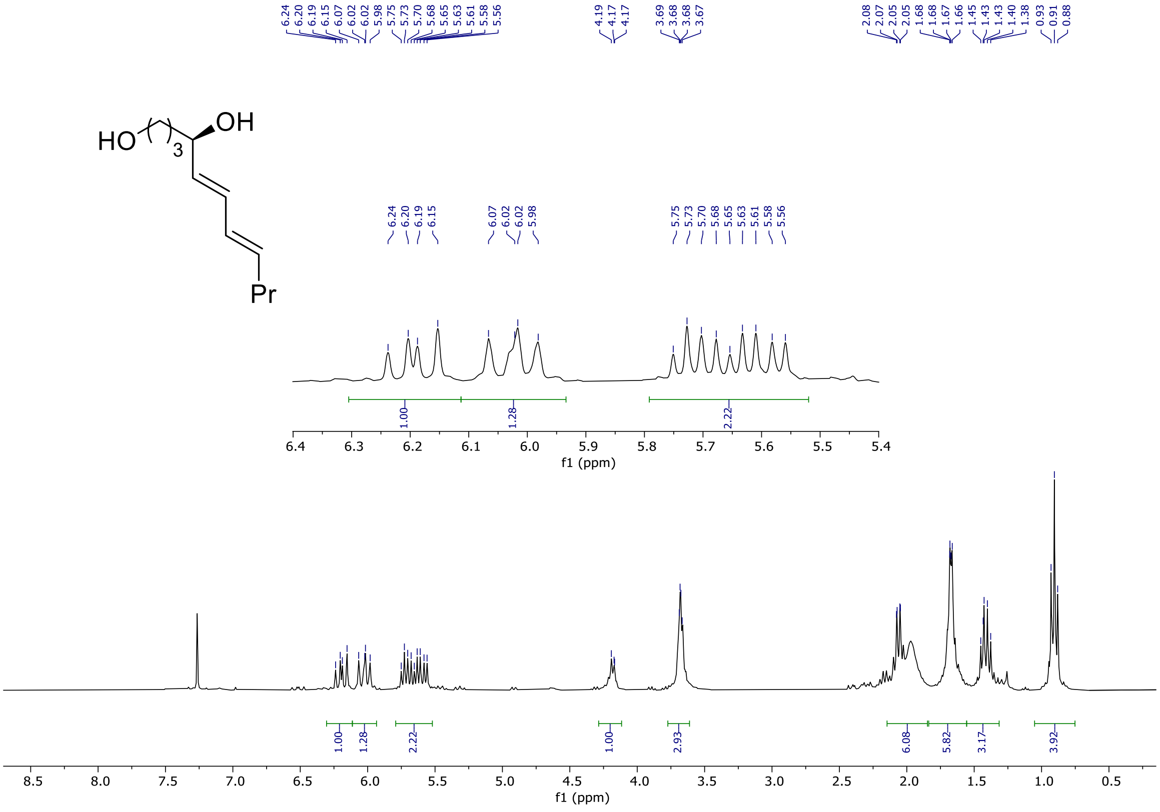

^{1}H NMR ($CDCl_3$, 400 MHz) **4i**

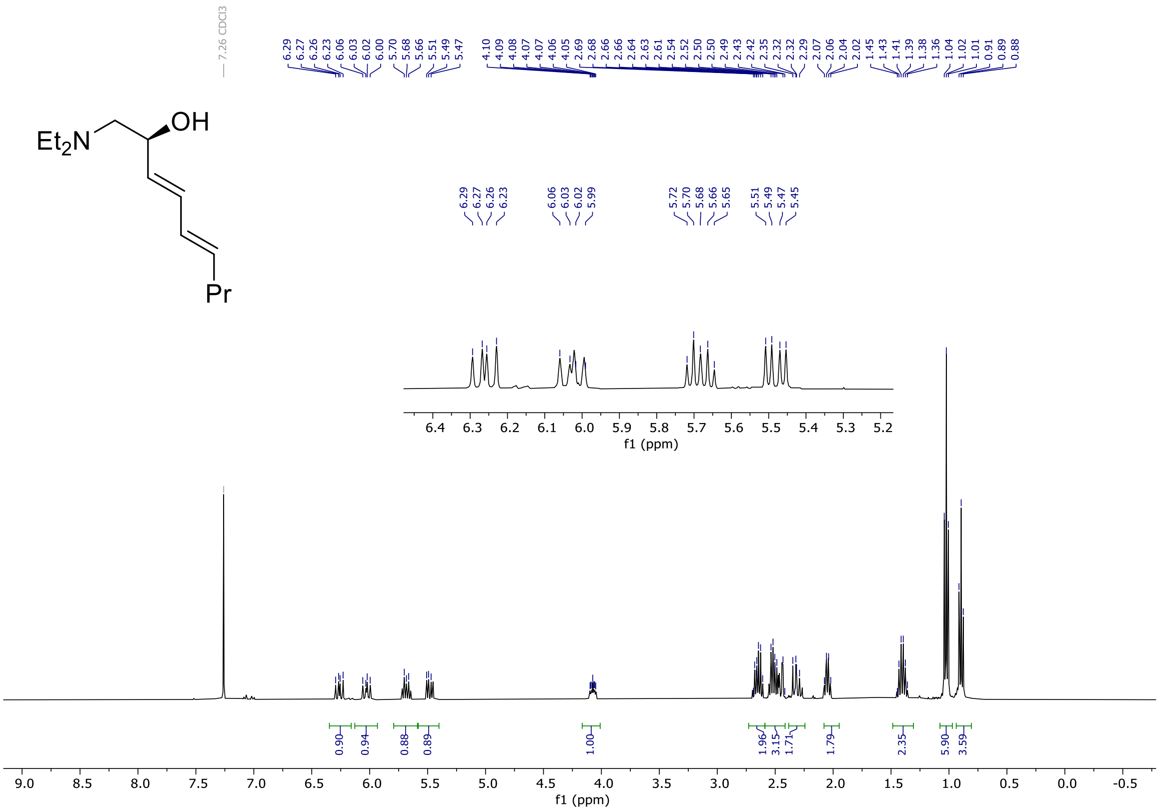

^{13}C NMR ($CDCl_3$, 100 MHz) **4i**

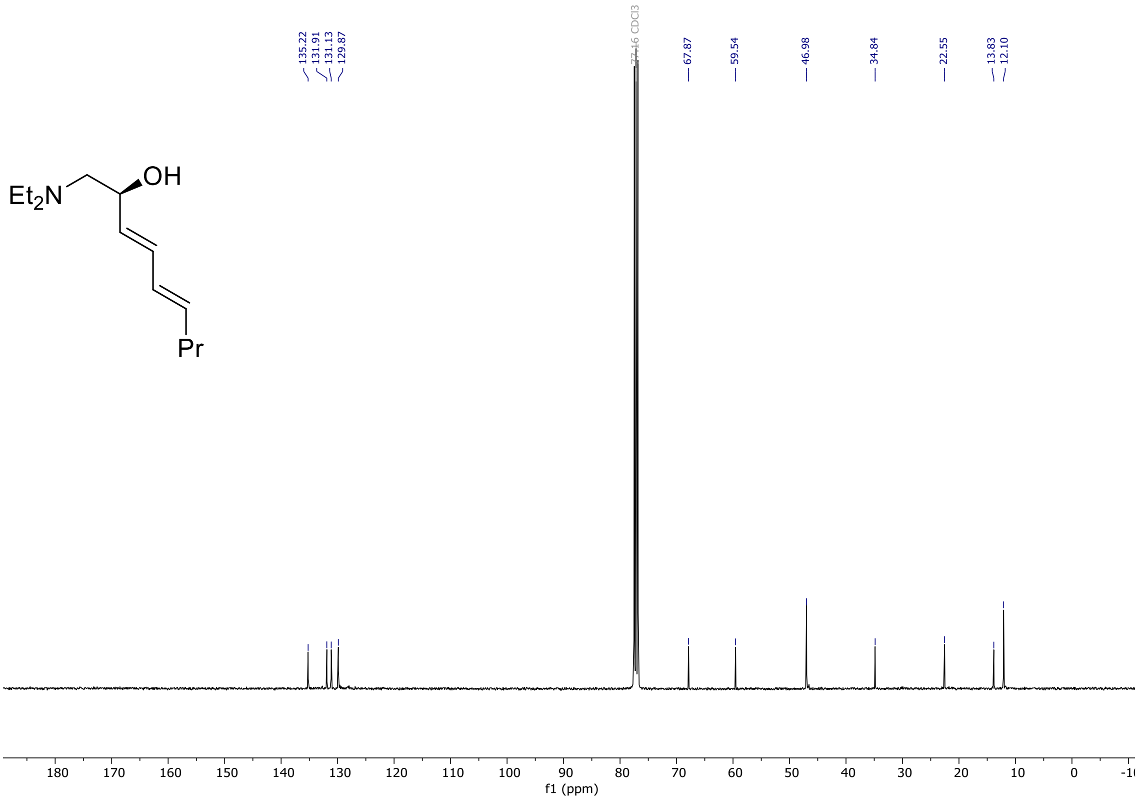

COSY (^{1}H, ^{1}H) **4i**

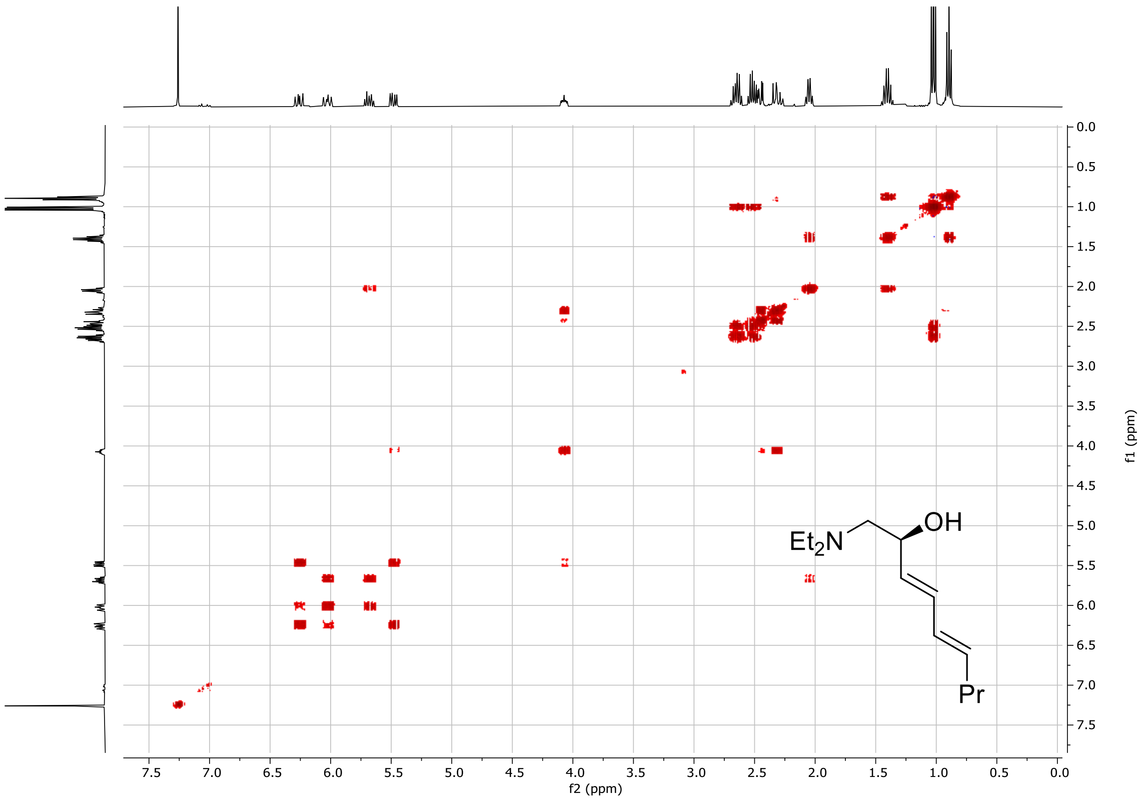

HSQC (^{1}H, ^{13}C) **4i**

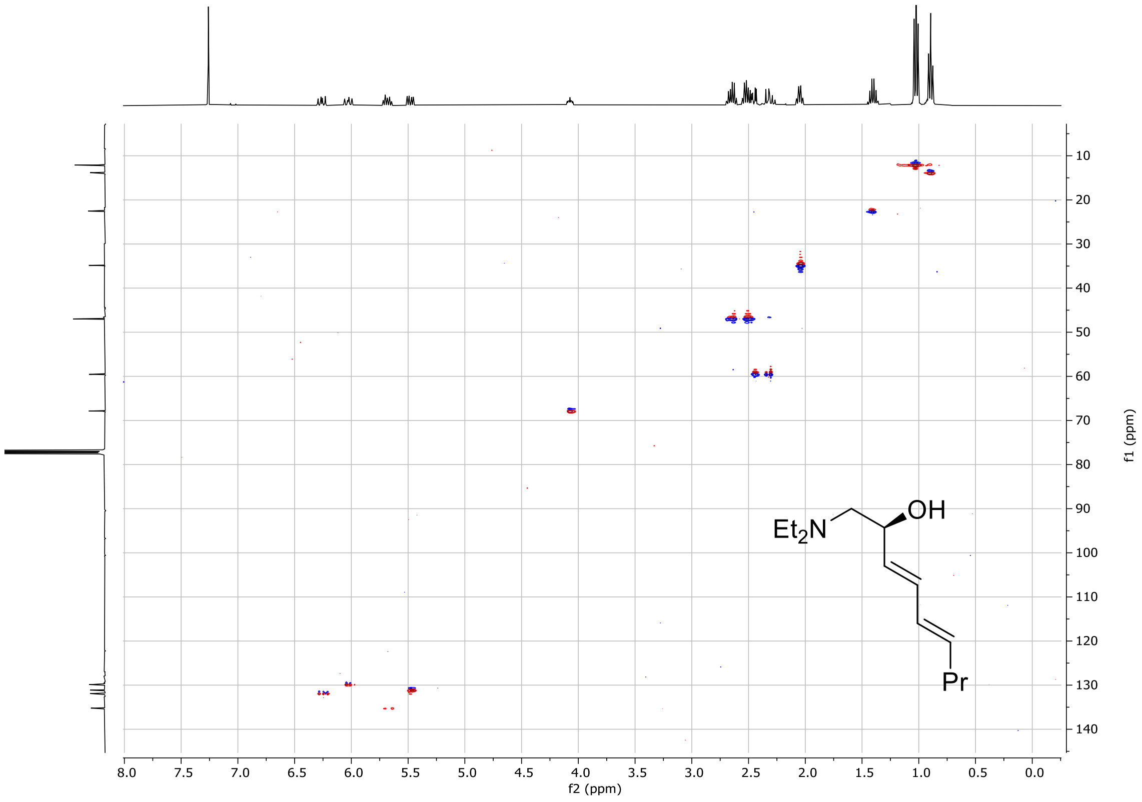

^{1}H NMR ($CDCl_3$, 400 MHz) **5i**

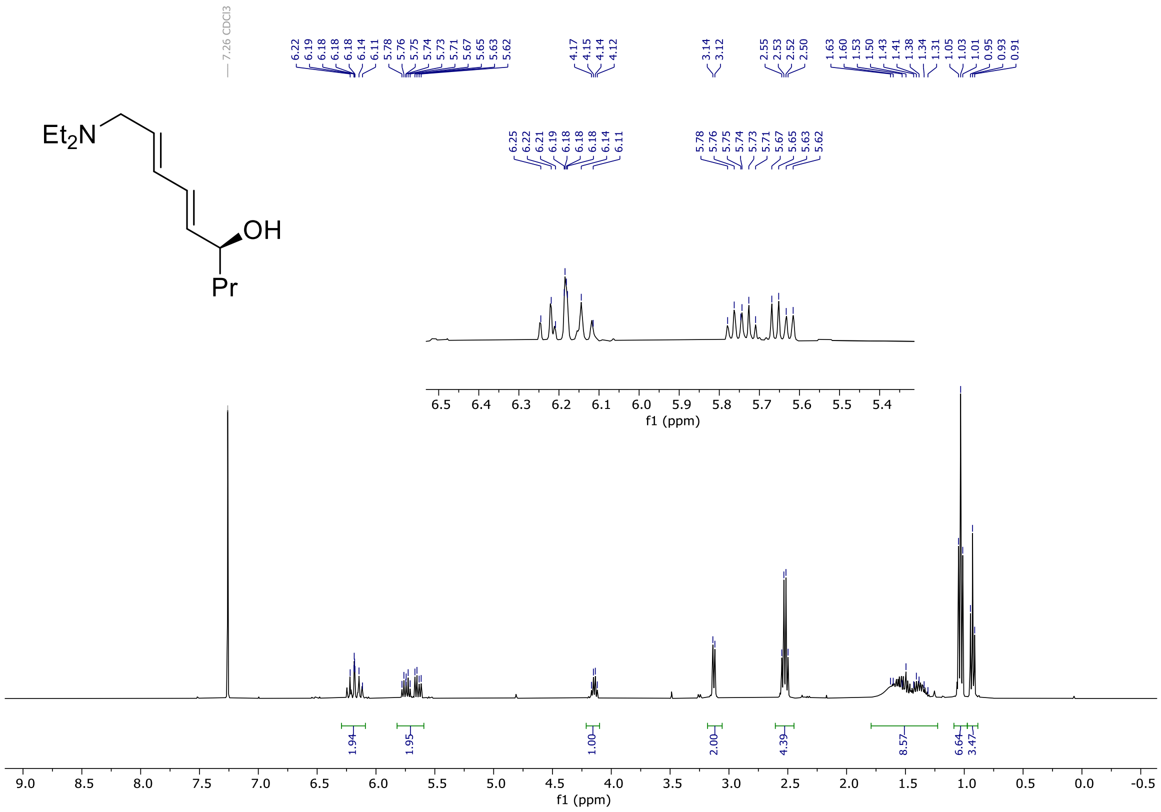

^{13}C NMR ($CDCl_3$, 100 MHz) **5i**

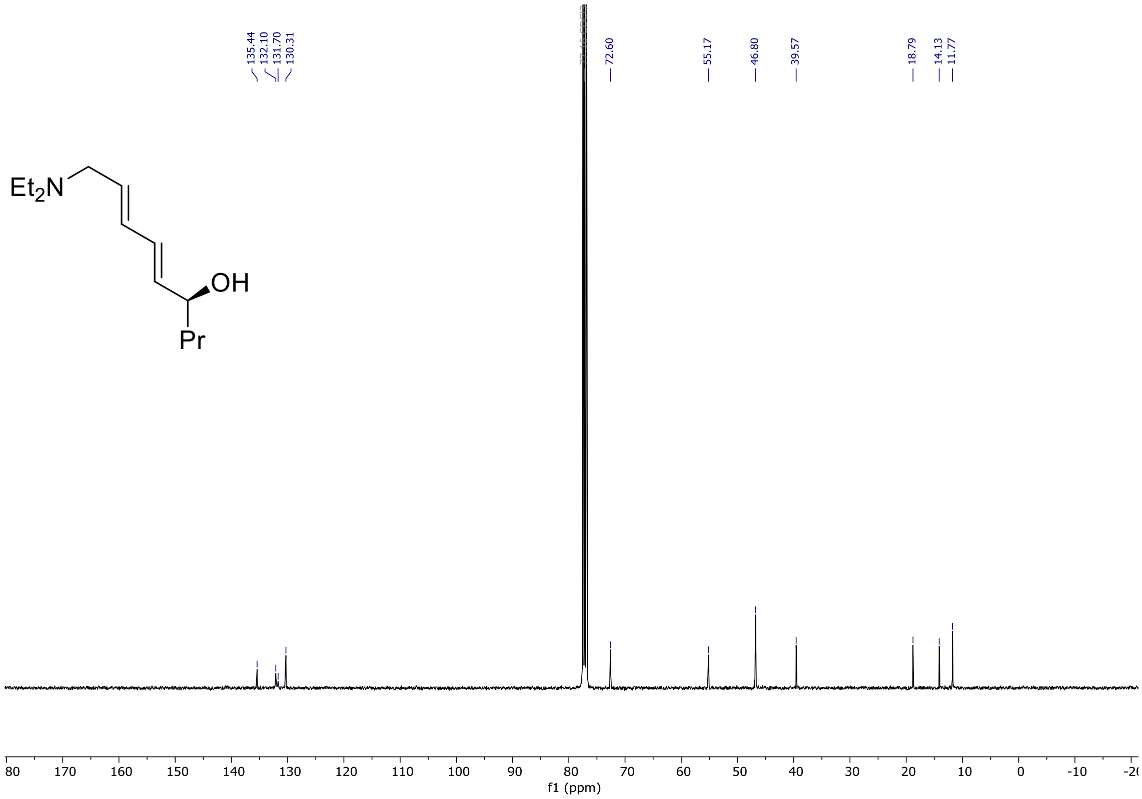

COSY (^{1}H, ^{1}H) **5i**

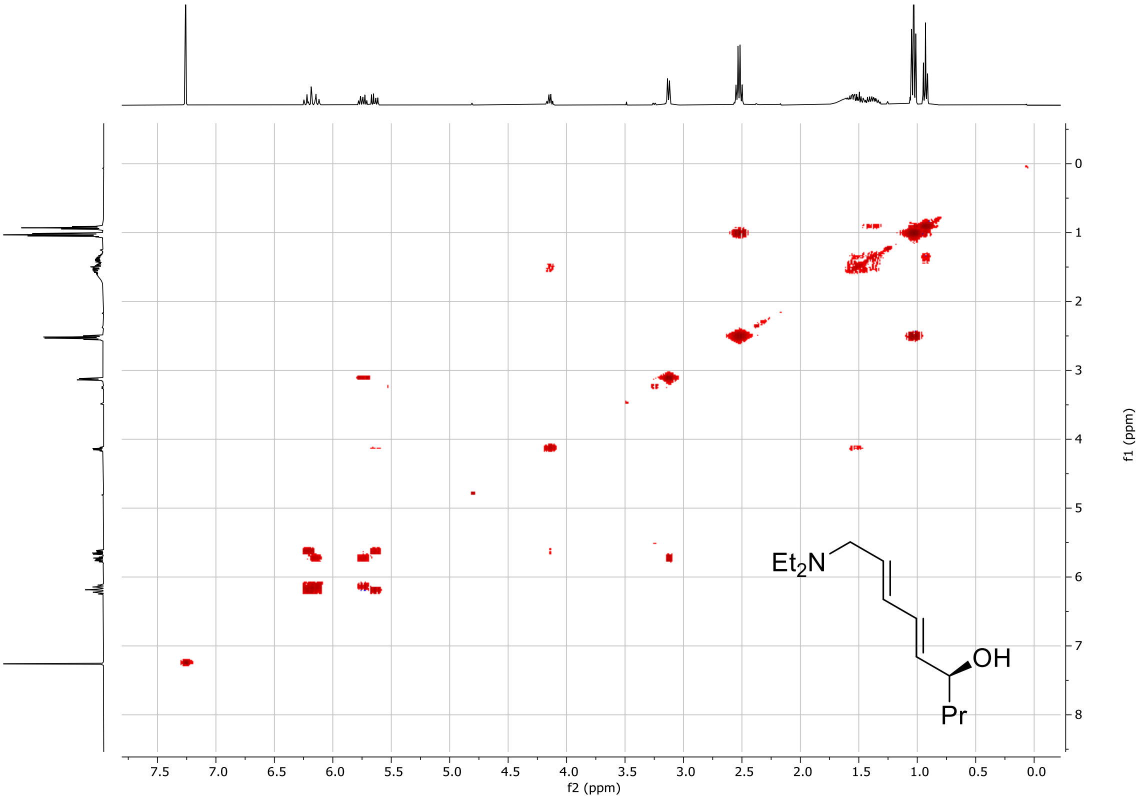

HSQC (^{1}H, ^{13}C) **5i**

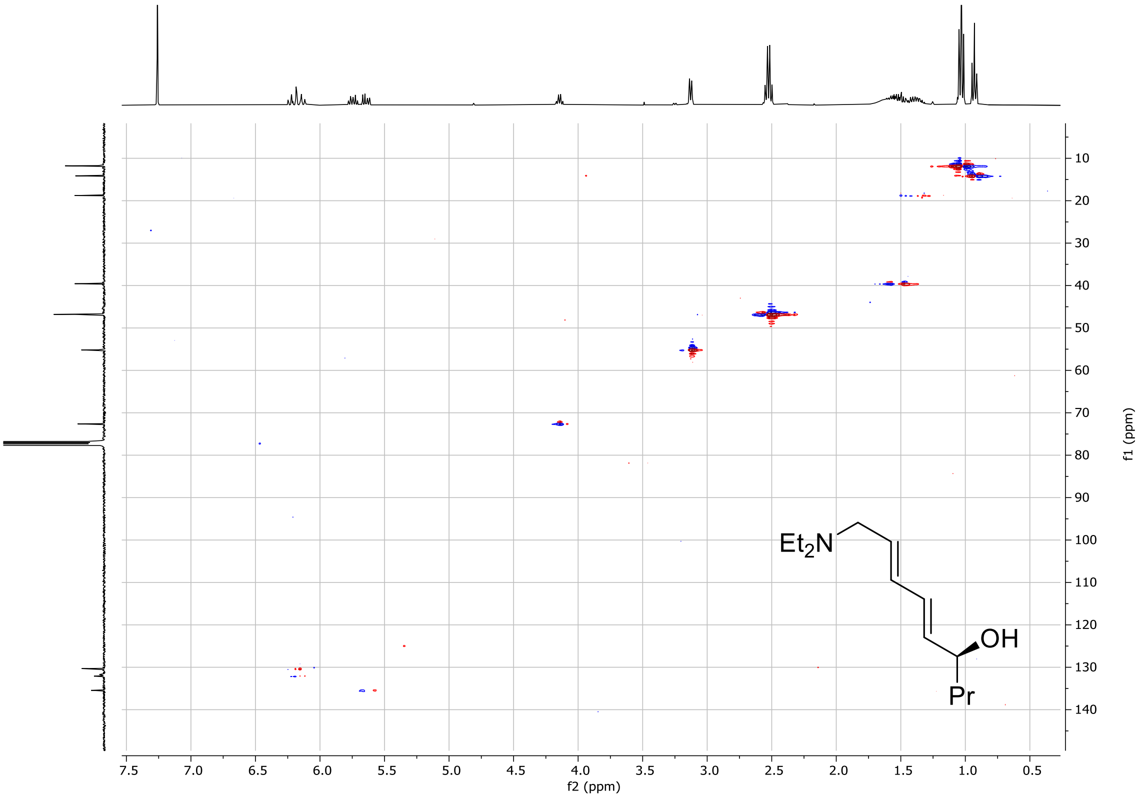

^1H NMR ($CDCl_3$, 500 MHz) **6i**

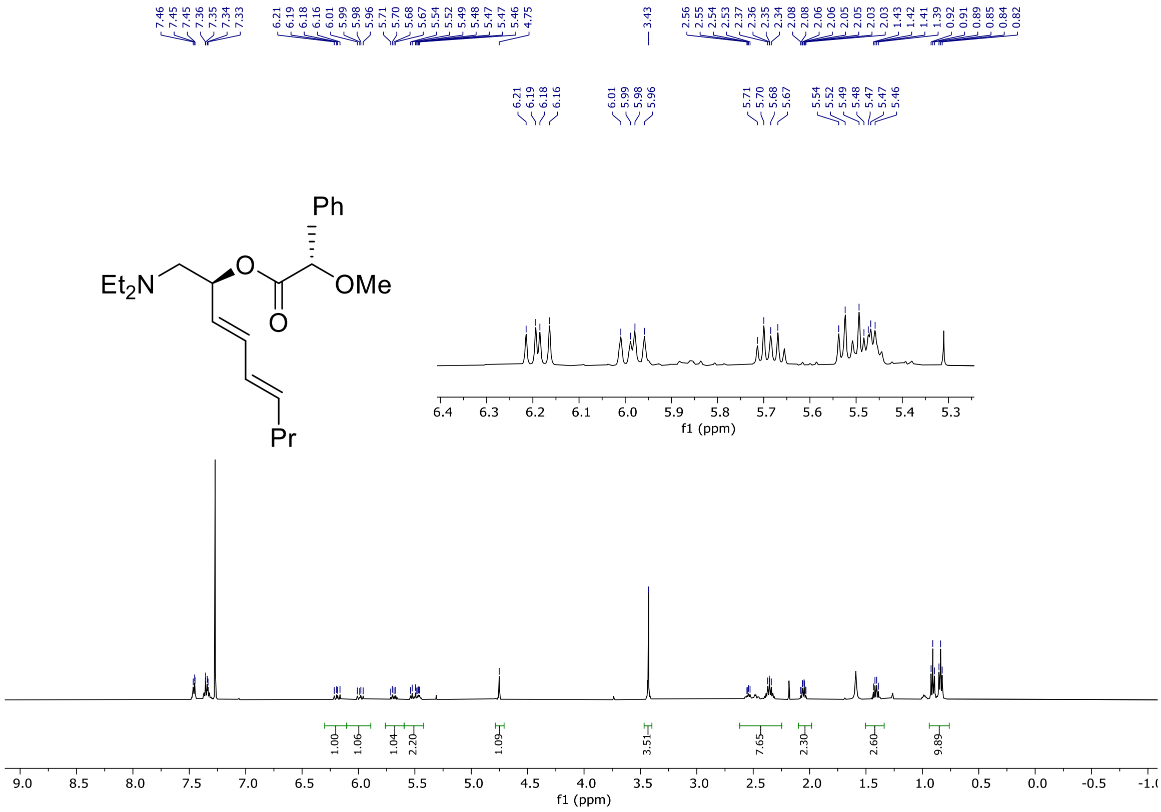

COSY (^1H, ^1H) **6i**

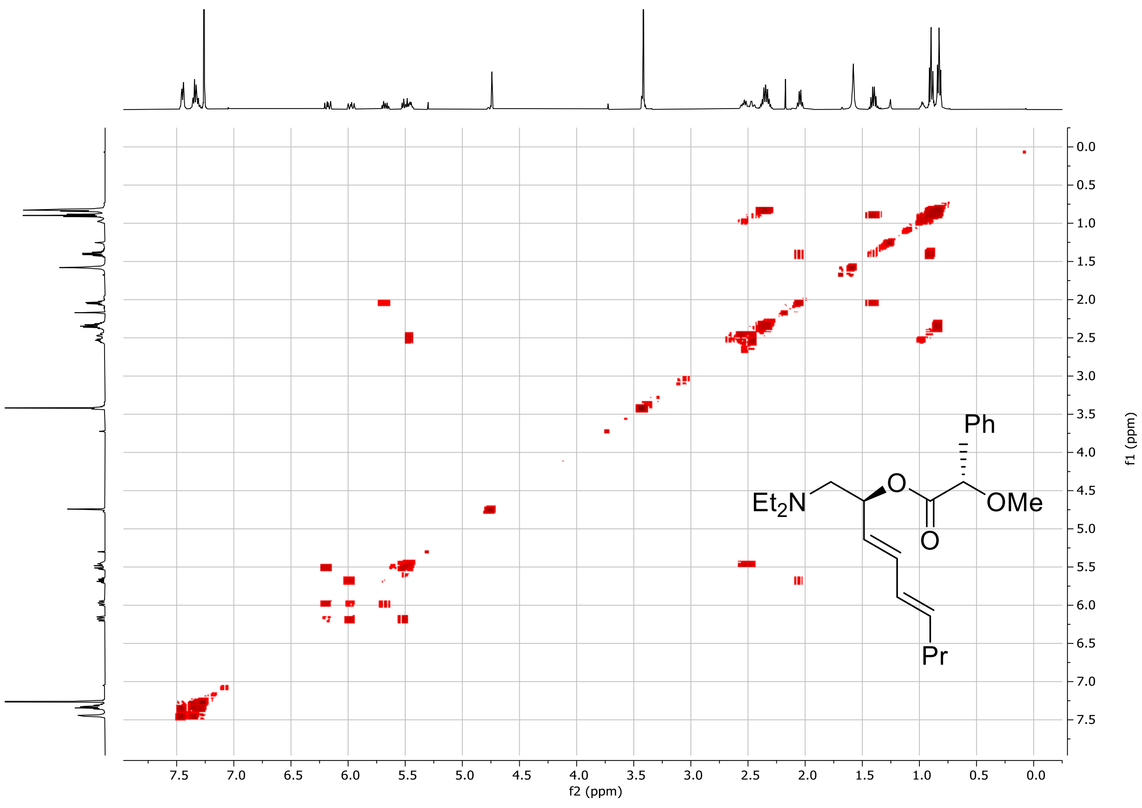

^{1}H NMR ($CDCl_3$, 500 MHz) **6i'**

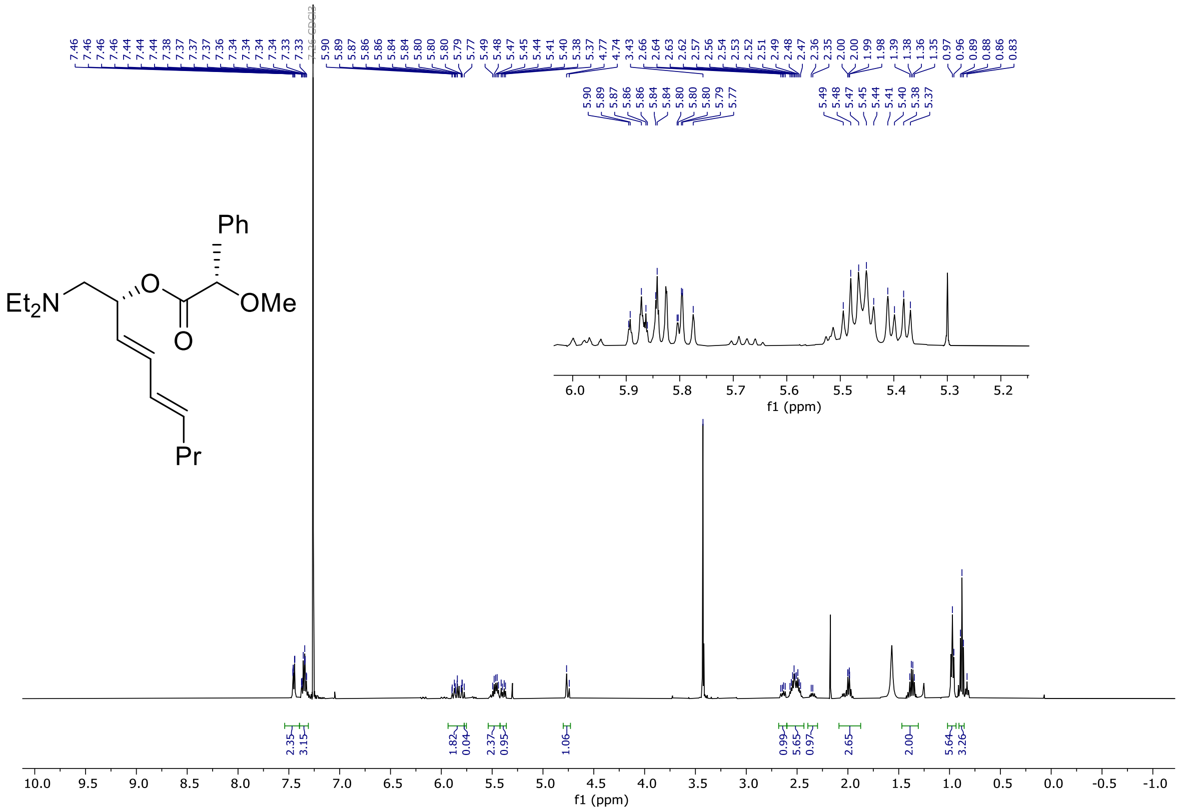

COSY (^{1}H, ^{1}H) **6i'**

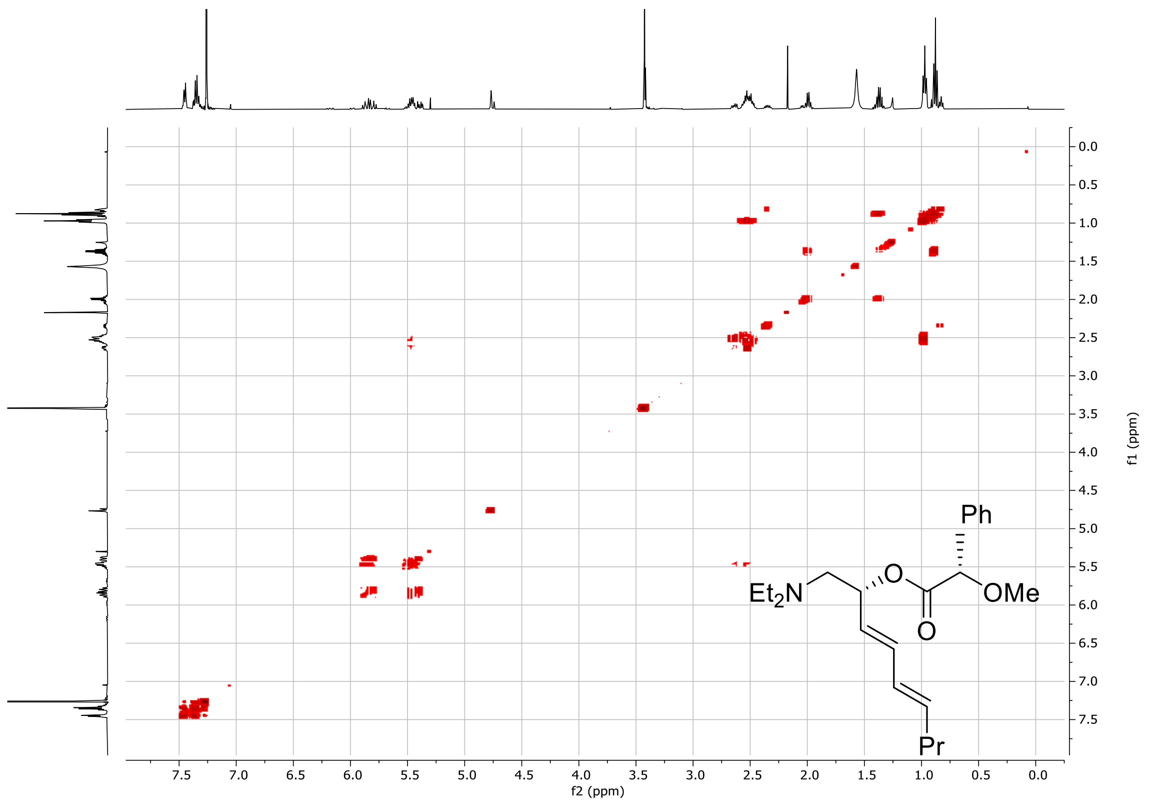

^{1}H NMR ($CDCl_3$, 500 MHz) **4j**

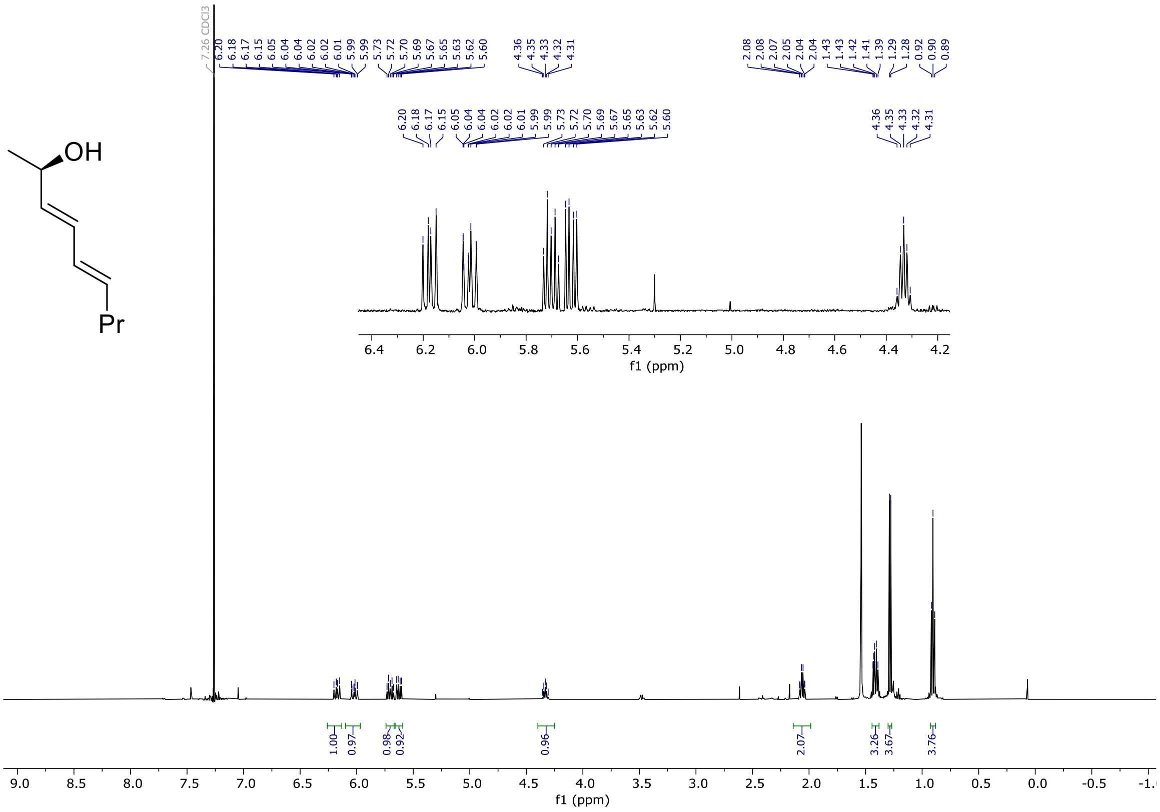

^{13}C NMR ($CDCl_3$, 125MHz) **4j**

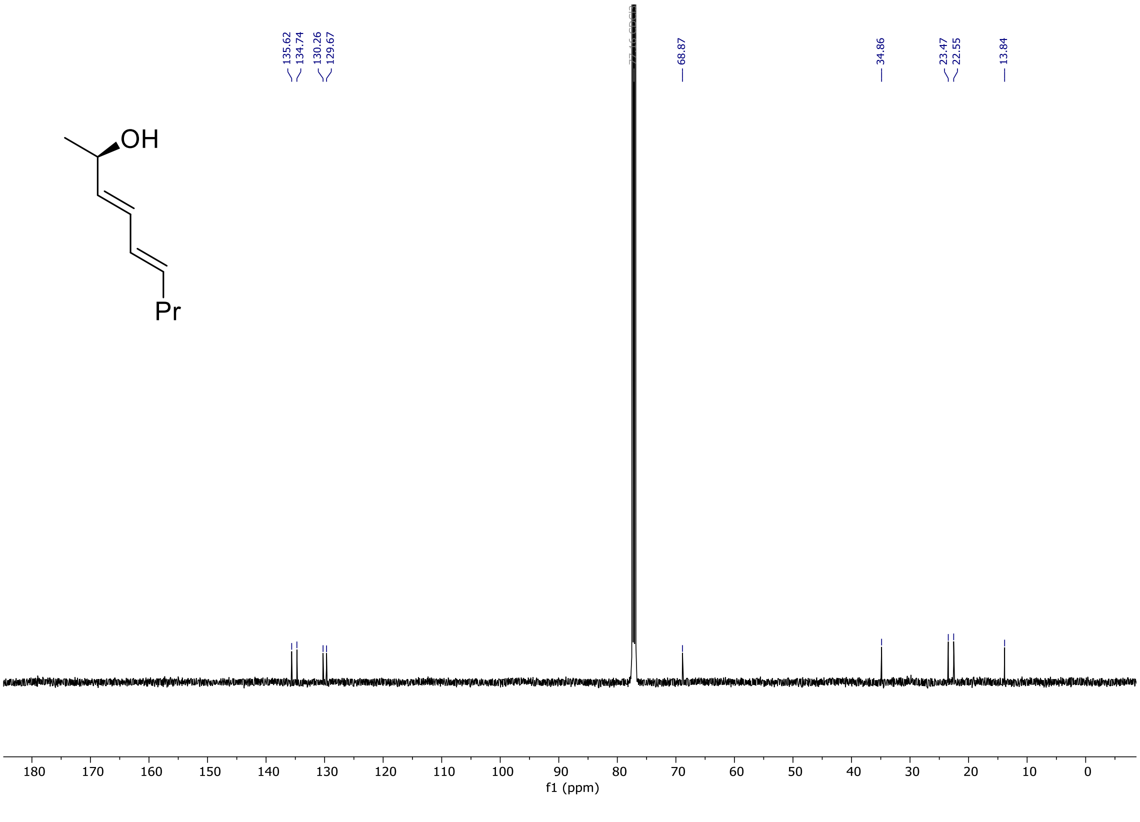

COSY (^{1}H, ^{1}H) **4j**

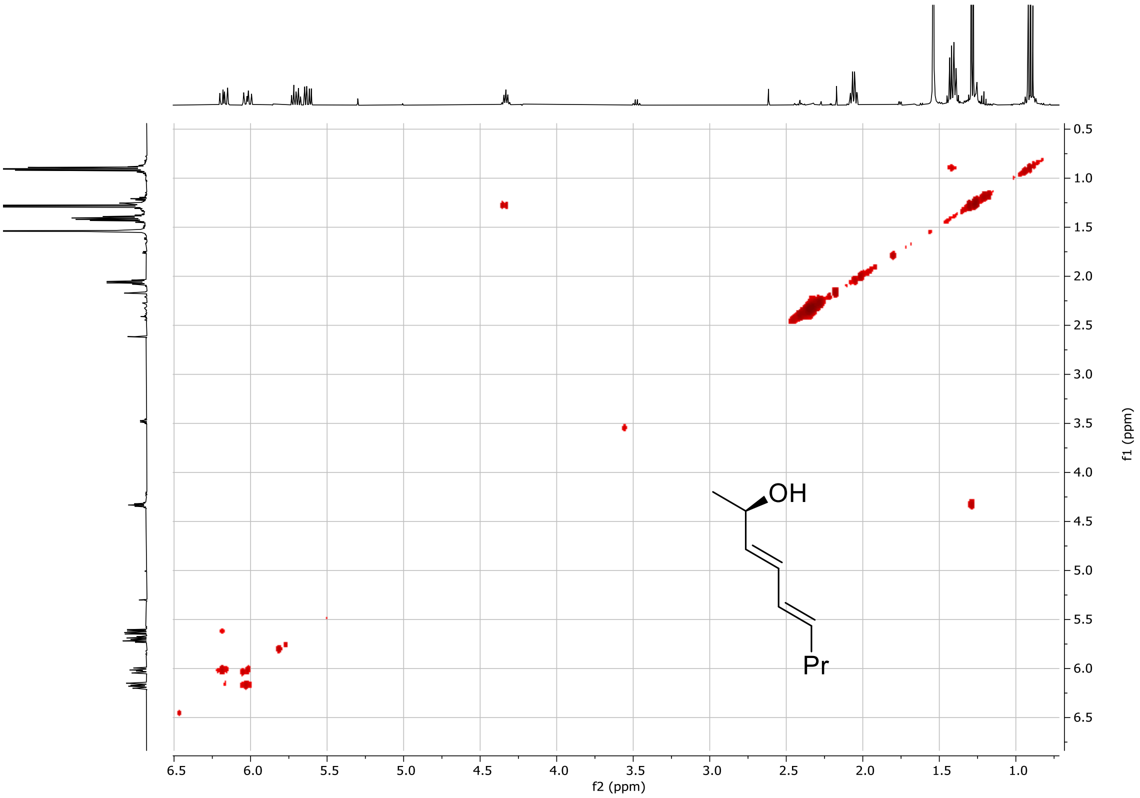

^{1}H NMR (CDCl$_3$, 500 MHz) **4k:5k**

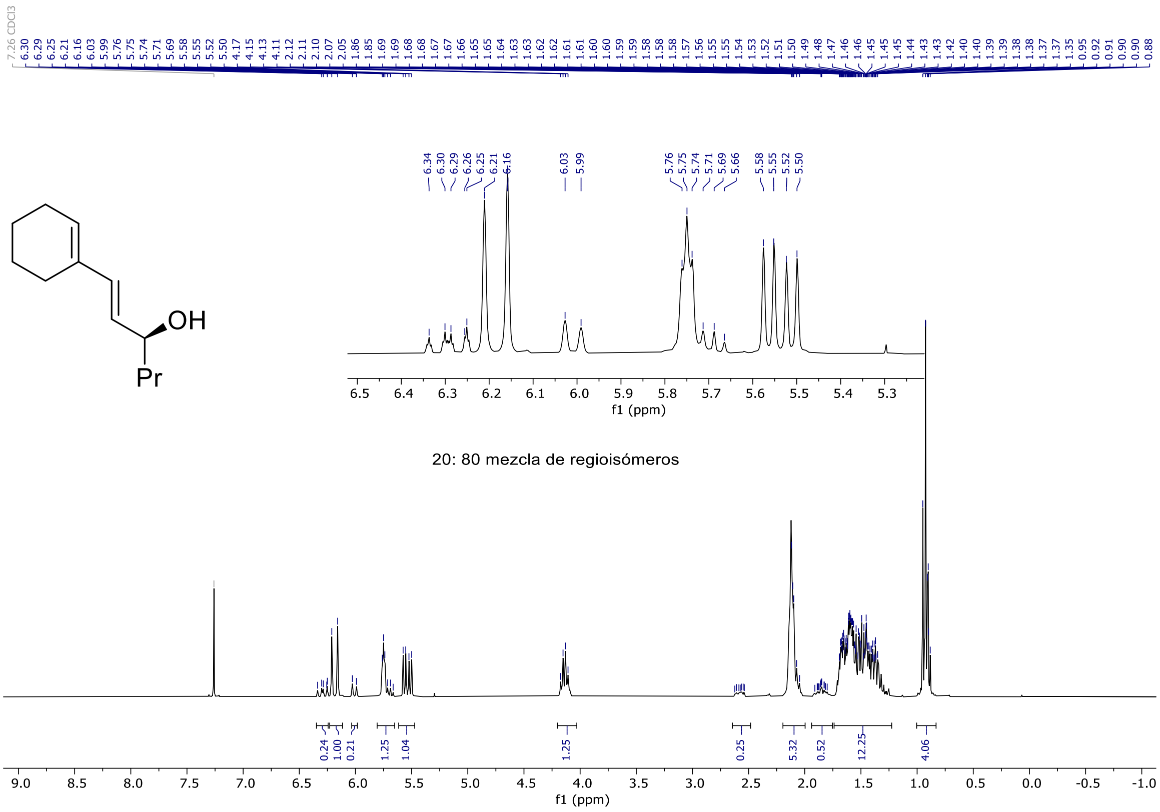

^{13}C NMR ($CDCl_3$, 125 MHz) **4k:5k**

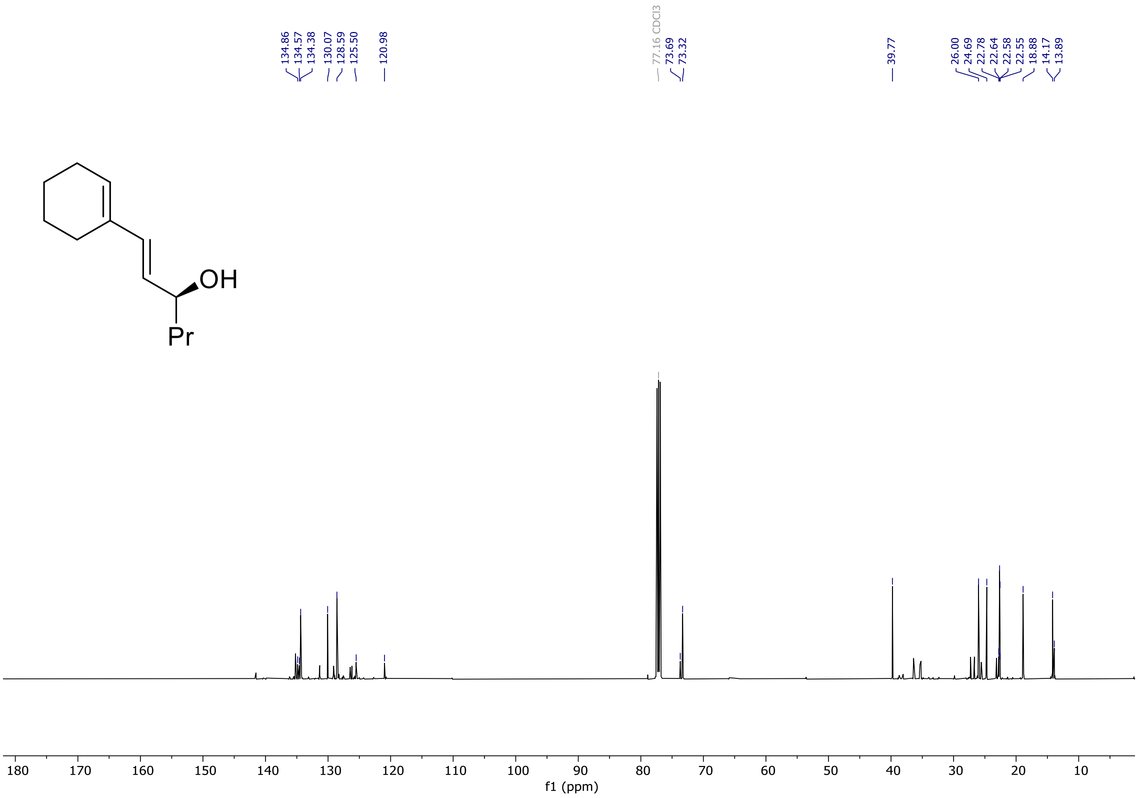

COSY (^{1}H, ^{1}H) **4k:5k**

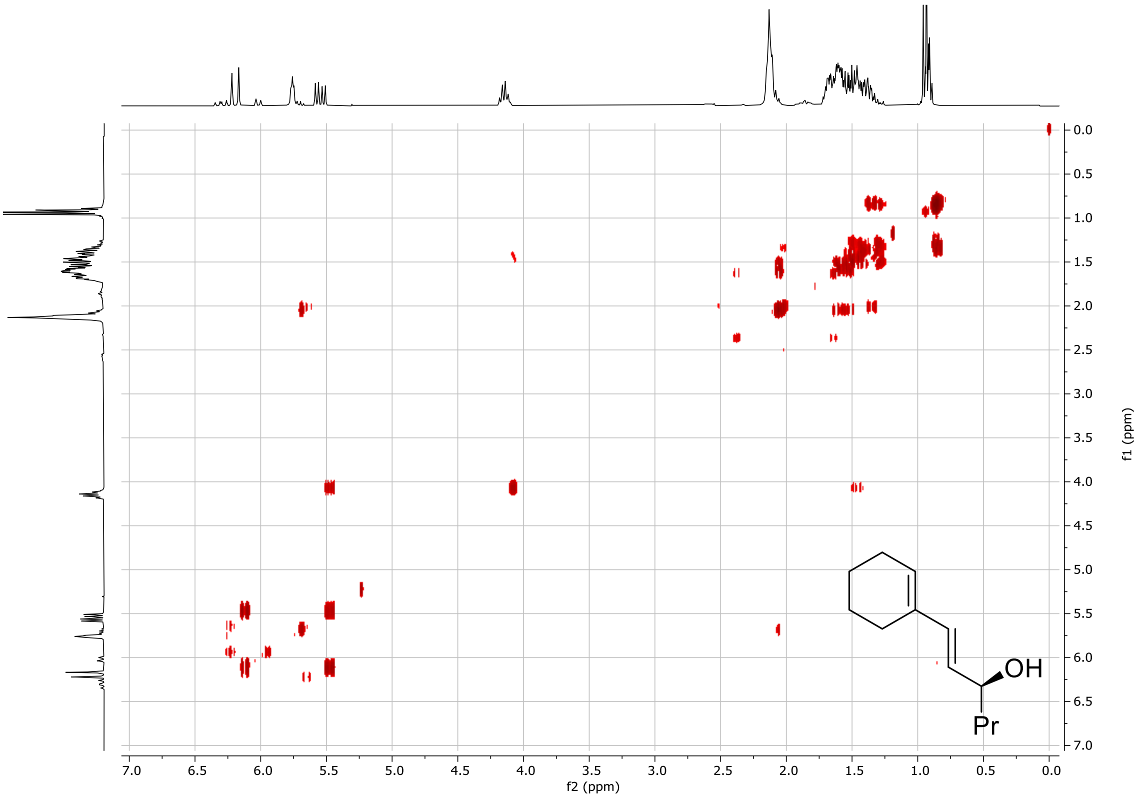

ROESY (^{1}H, ^{1}H) **4k:5k**

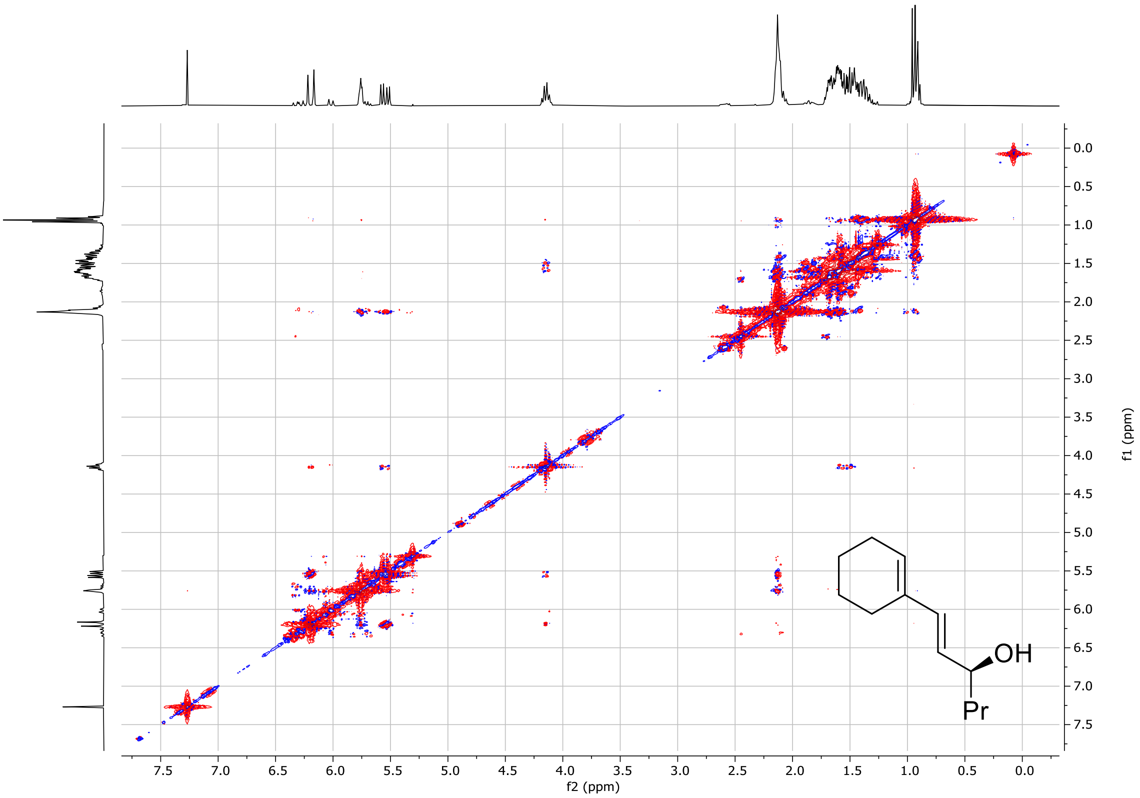

HSQC (^{1}H, ^{13}C) **4k:5k**

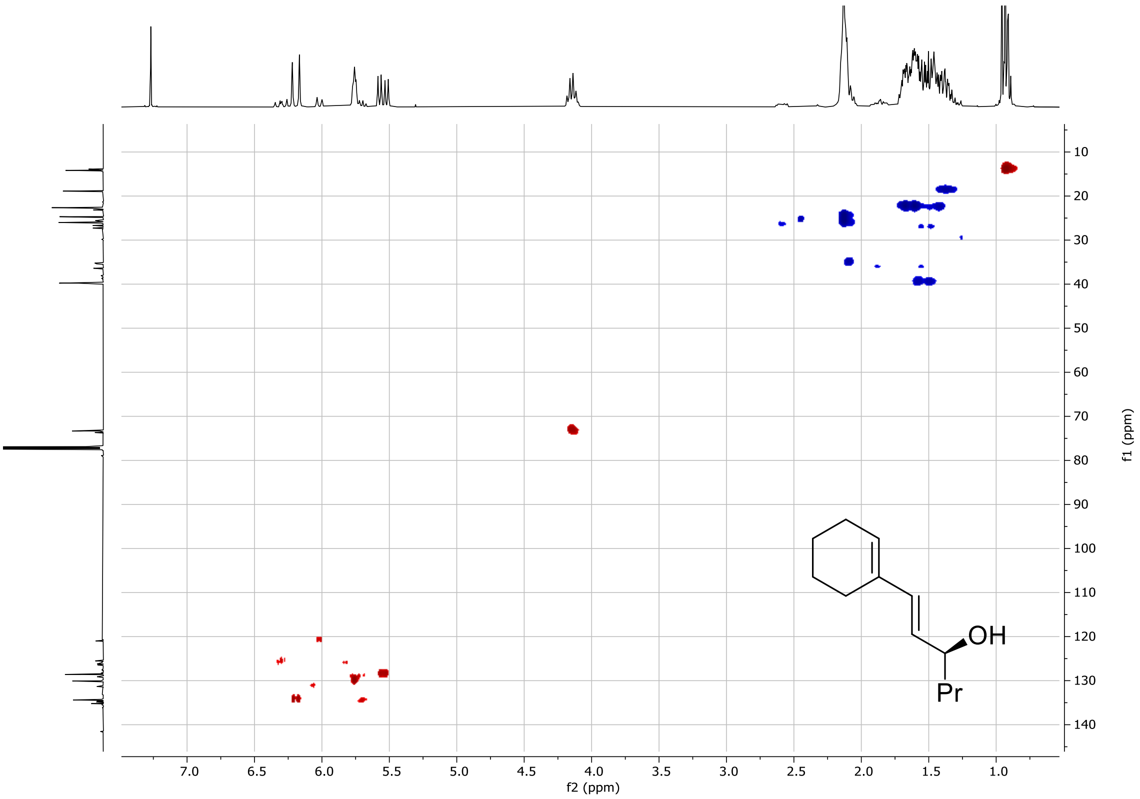

^{1}H NMR (CDCl$_3$, 400 MHz) **4l**

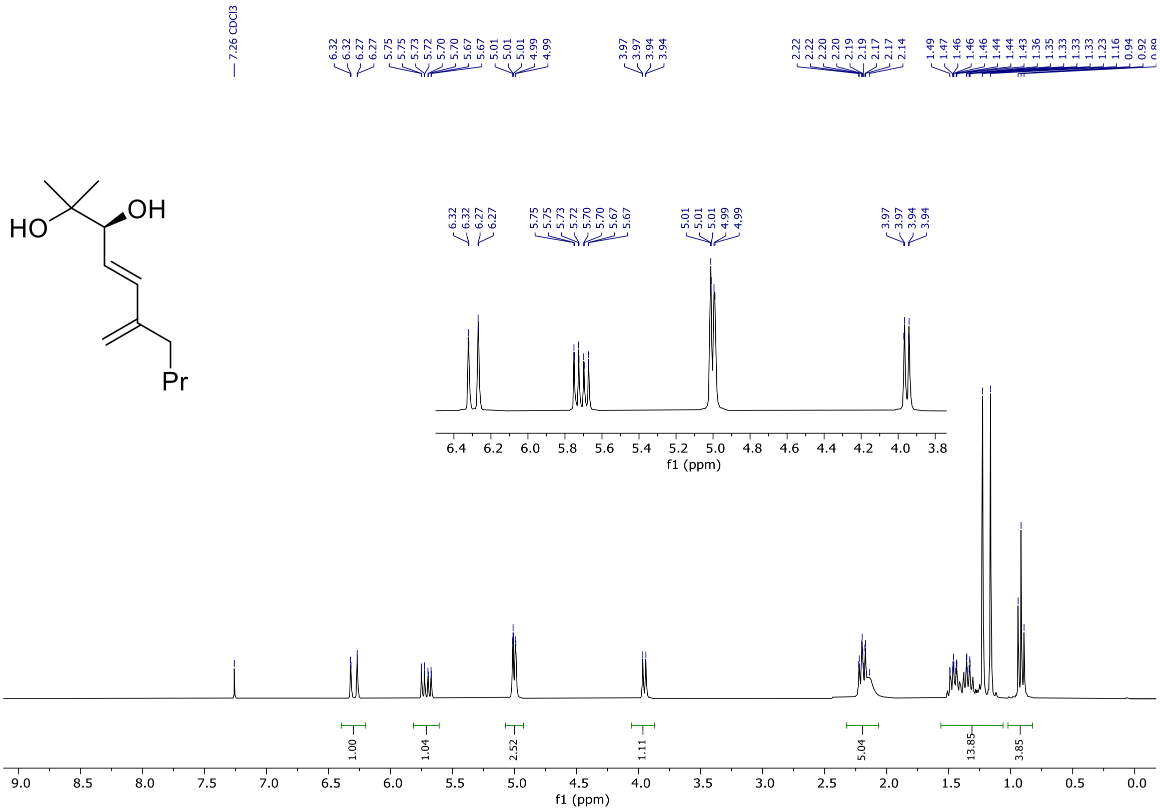

^{13}C NMR (CDCl$_3$, 100 MHz) **4l**

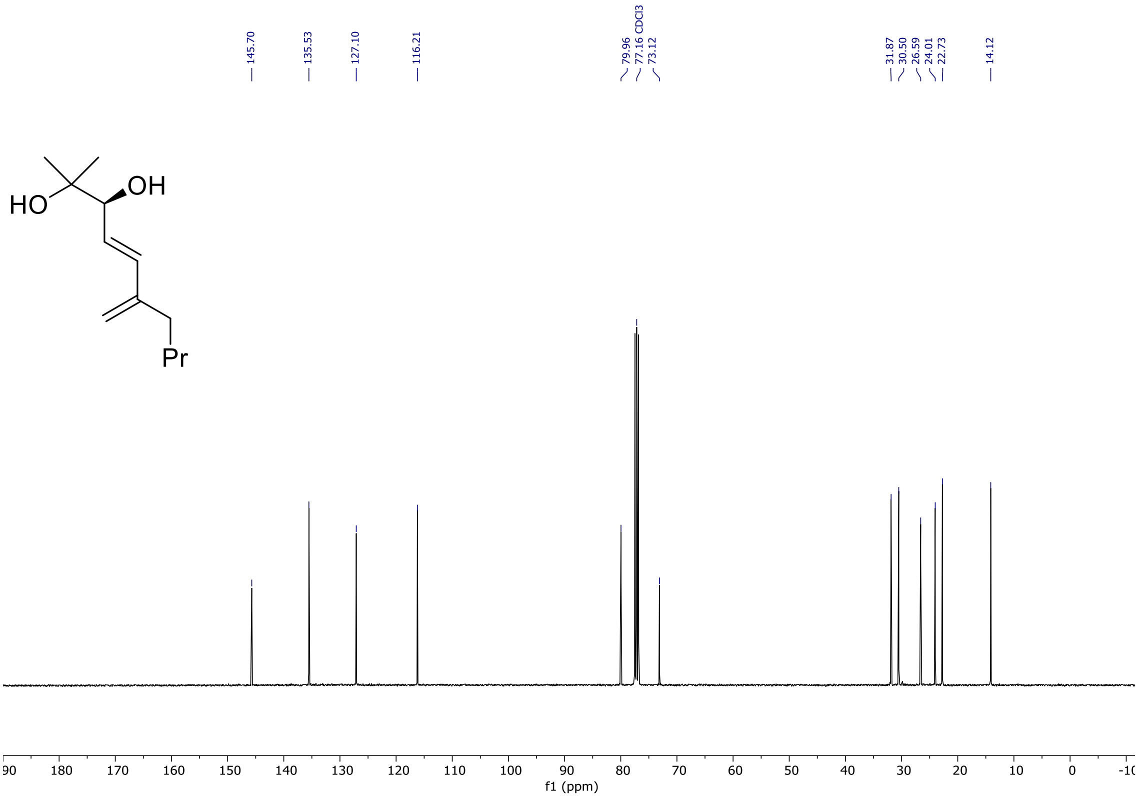

^1H NMR ($CDCl_3$, 400 MHz) **4la**

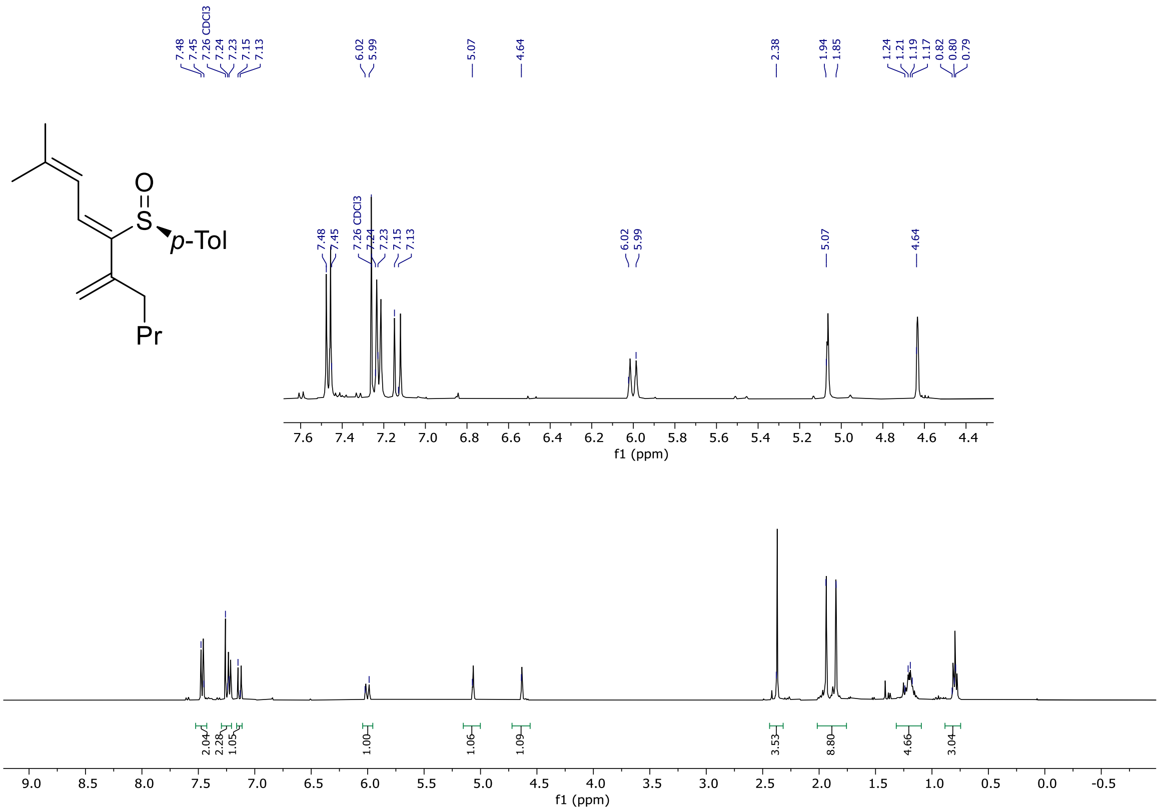

^{13}C NMR ($CDCl_3$, 100 MHz) **4la**

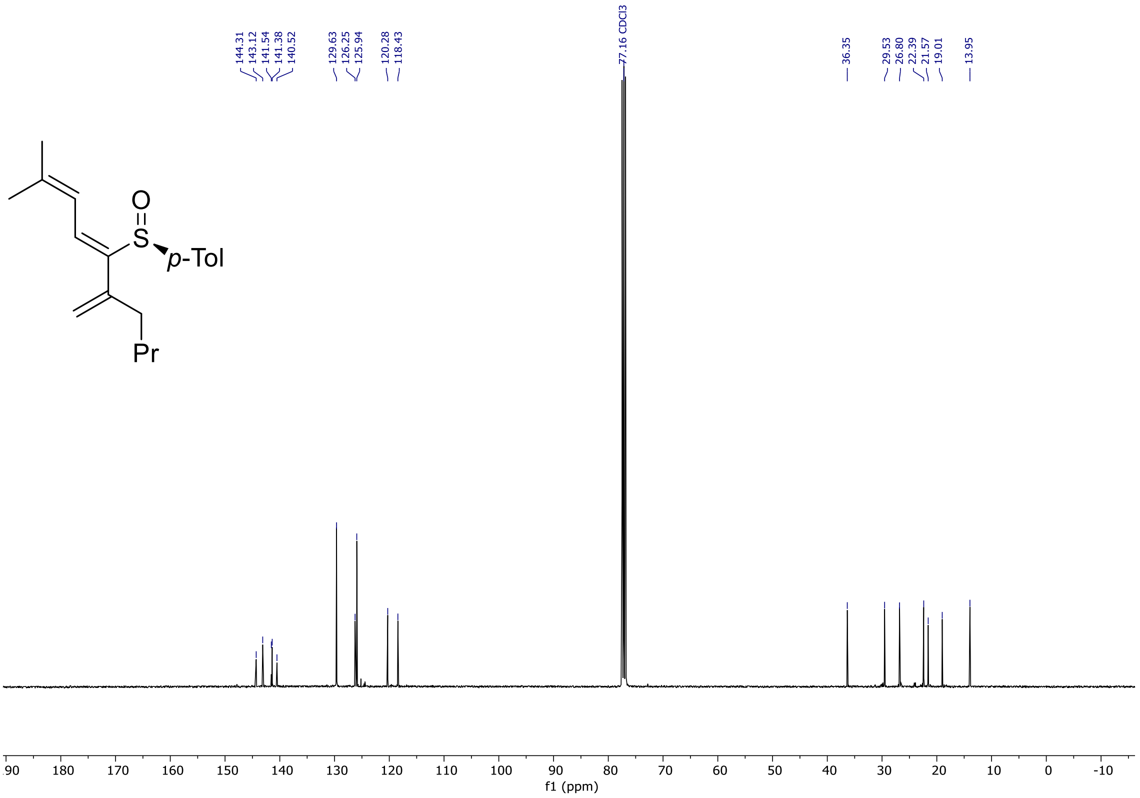

HSQC (^{1}H, ^{13}C) **4la**

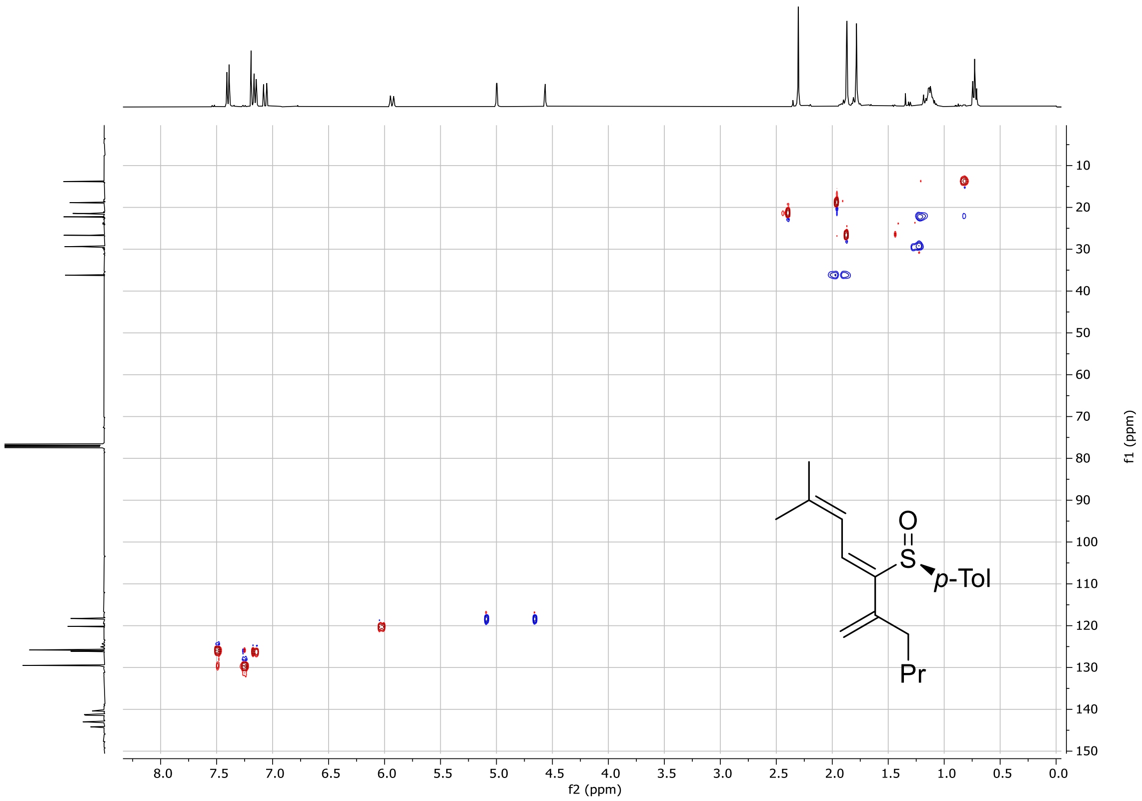

^{1}H NMR ($CDCl_3$, 500 MHz) **6l**

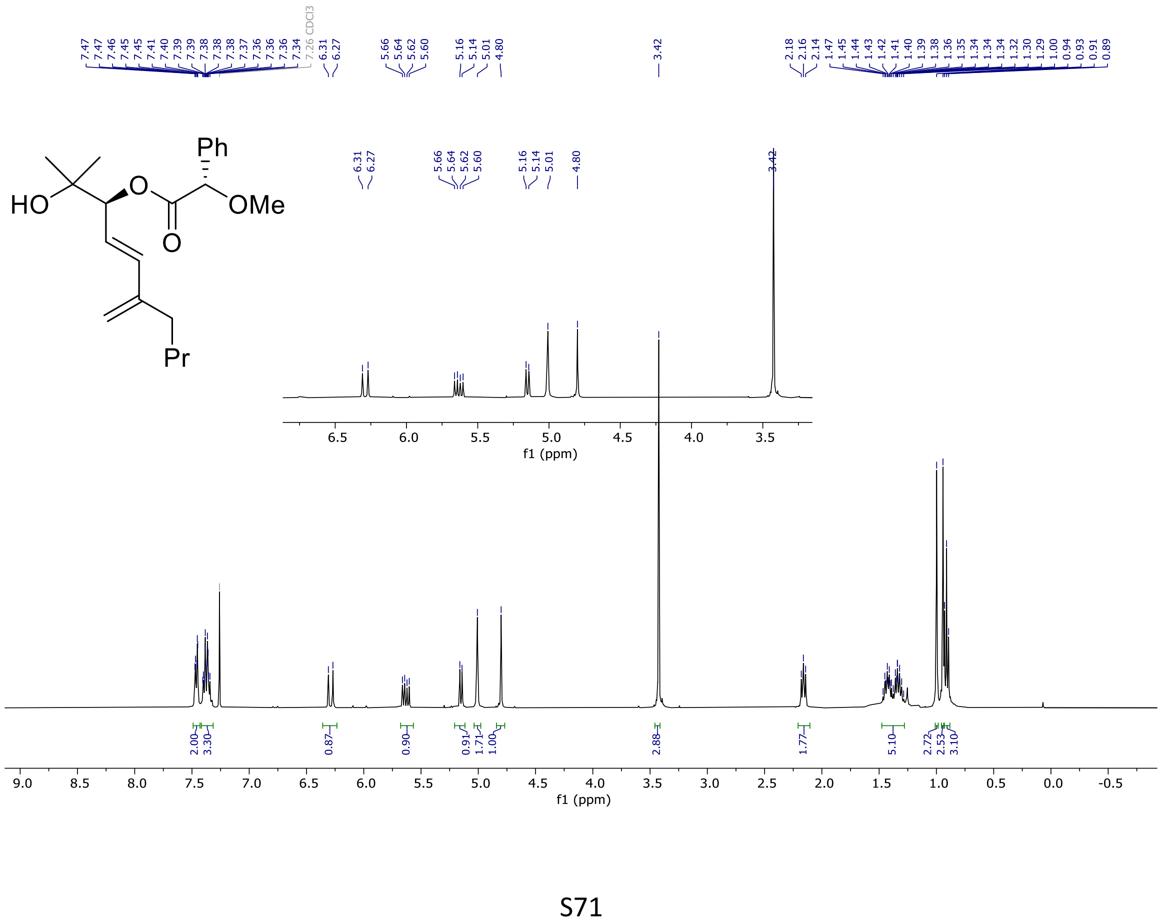

^{13}C NMR ($CDCl_3$, 125 MHz) **6l**

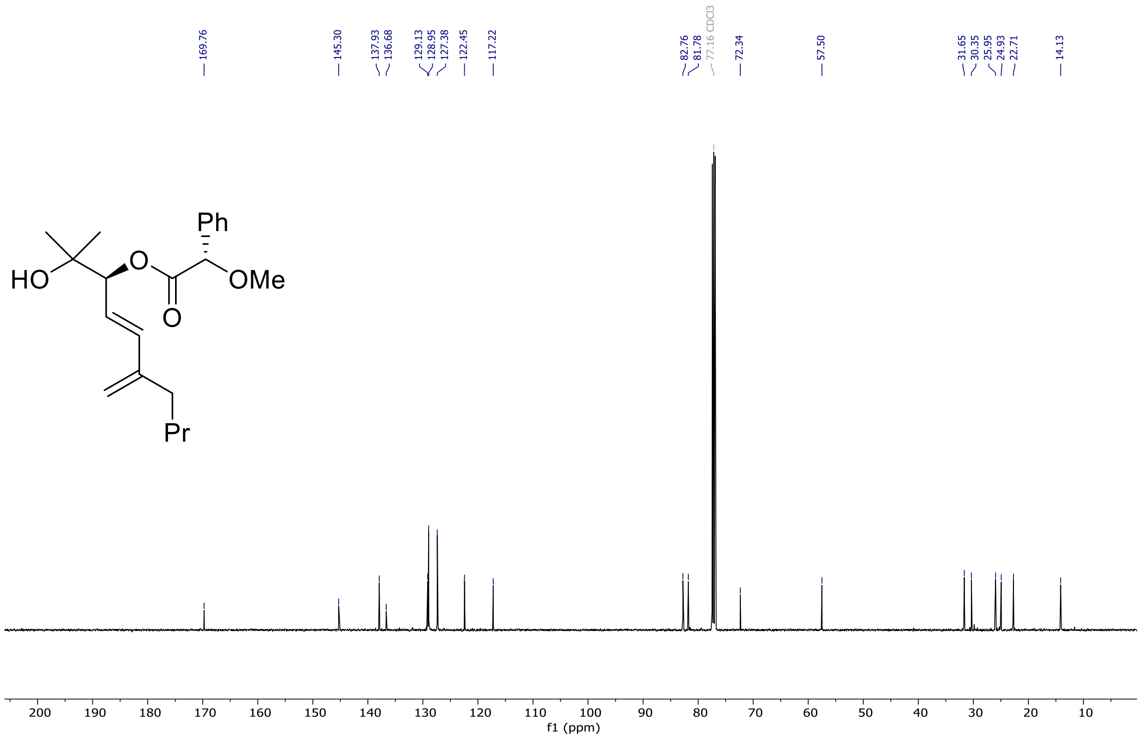

^{1}H NMR ($CDCl_3$, 300 MHz) **6l'**

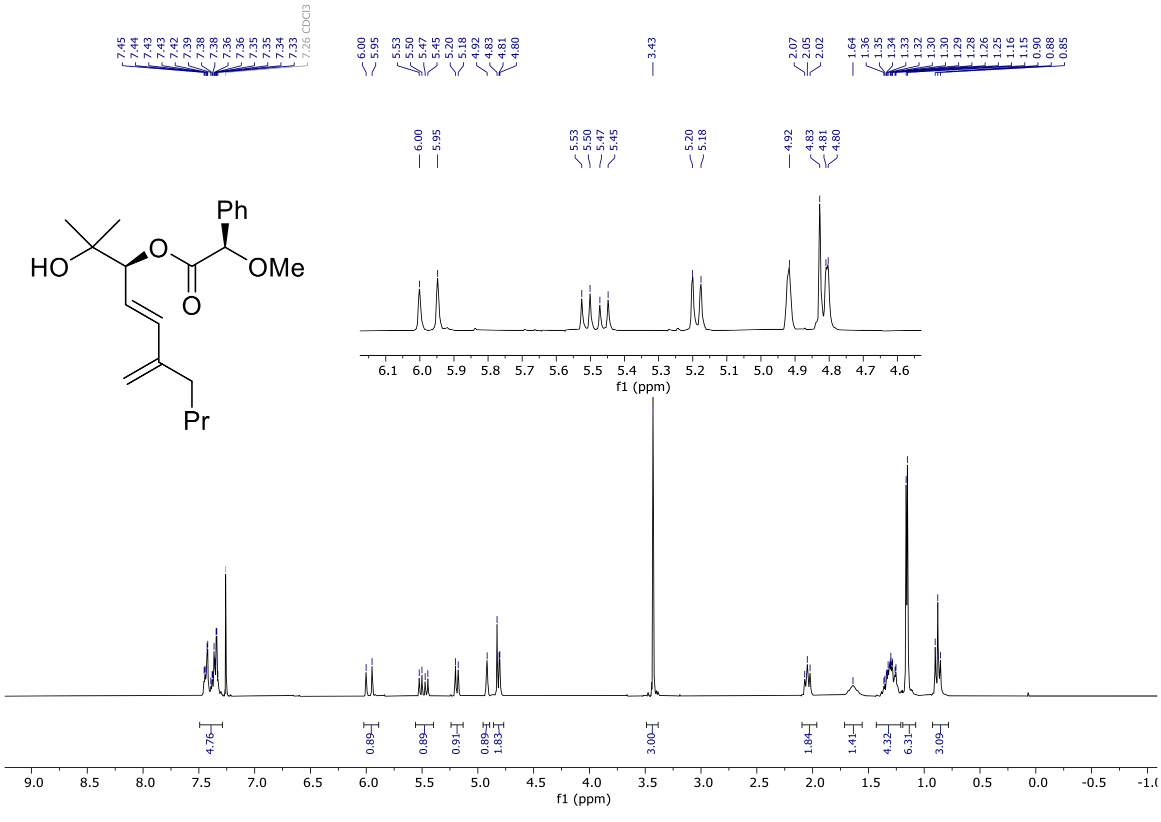

^{13}C NMR (CDCl$_3$, 125 MHz) **6l’**

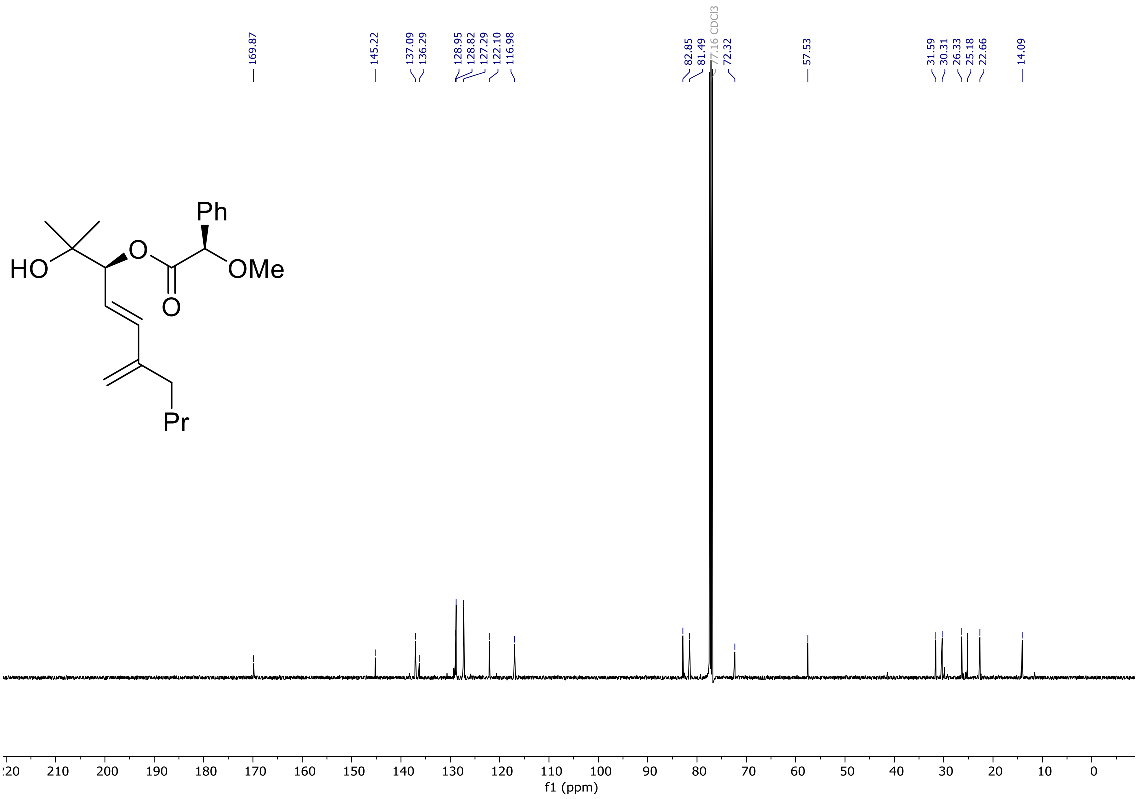

^{1}H NMR (CDCl$_3$, 500 MHz) **4m**

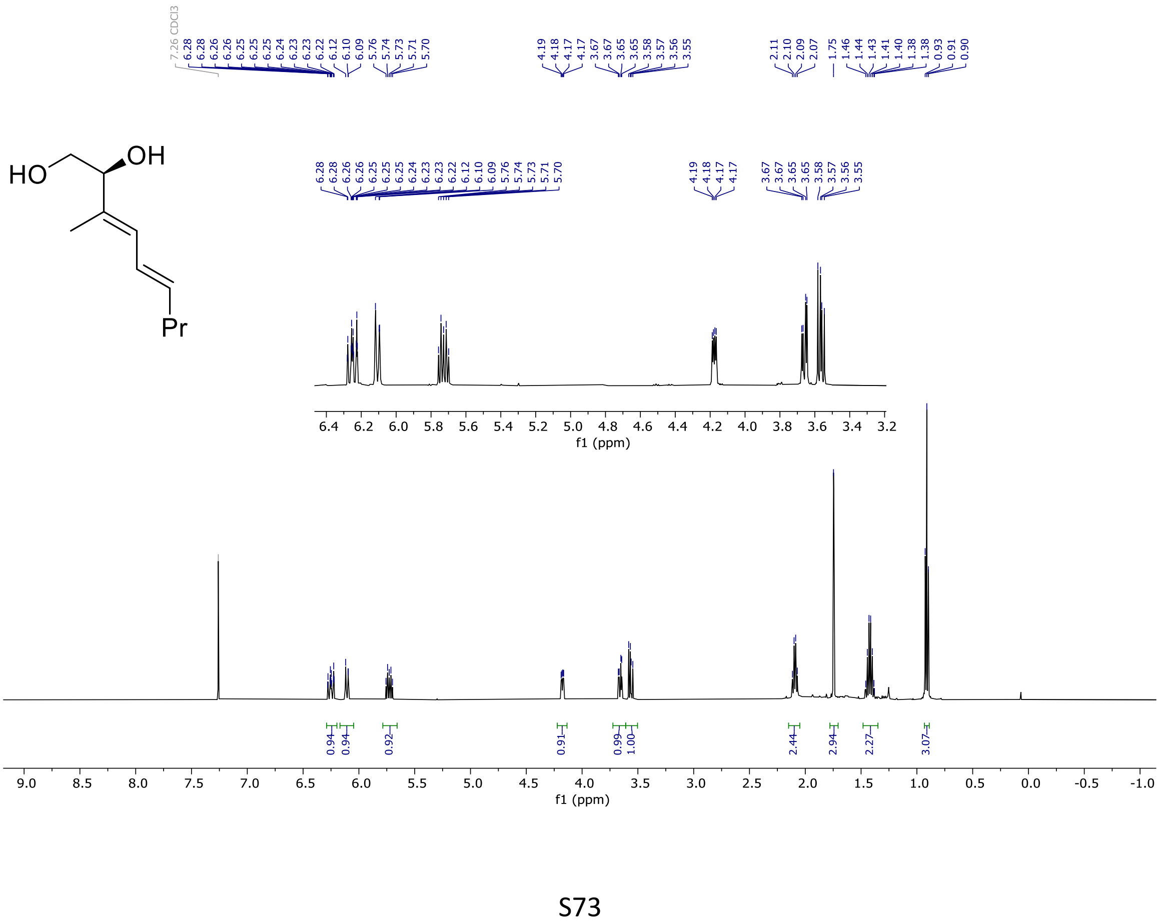

^{13}C NMR ($CDCl_3$, 125 MHz) **4m**

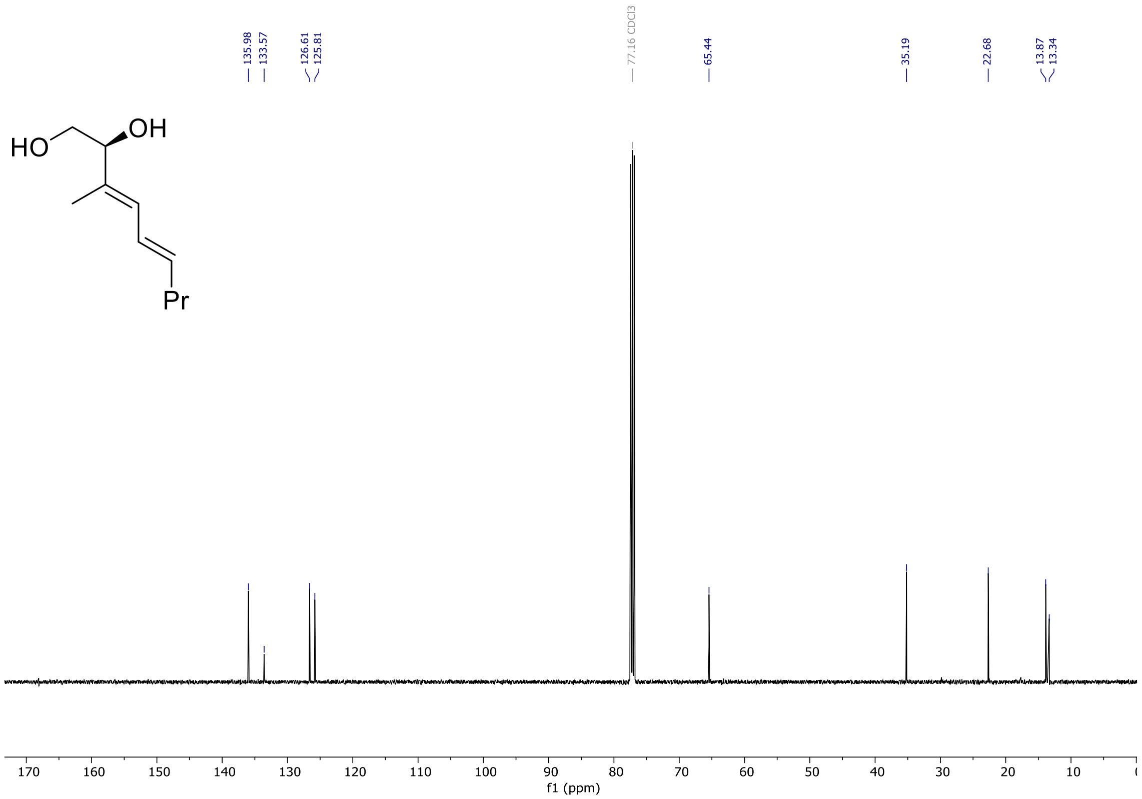

^{1}H NMR ($CDCl_3$, 300 MHz) **6m**

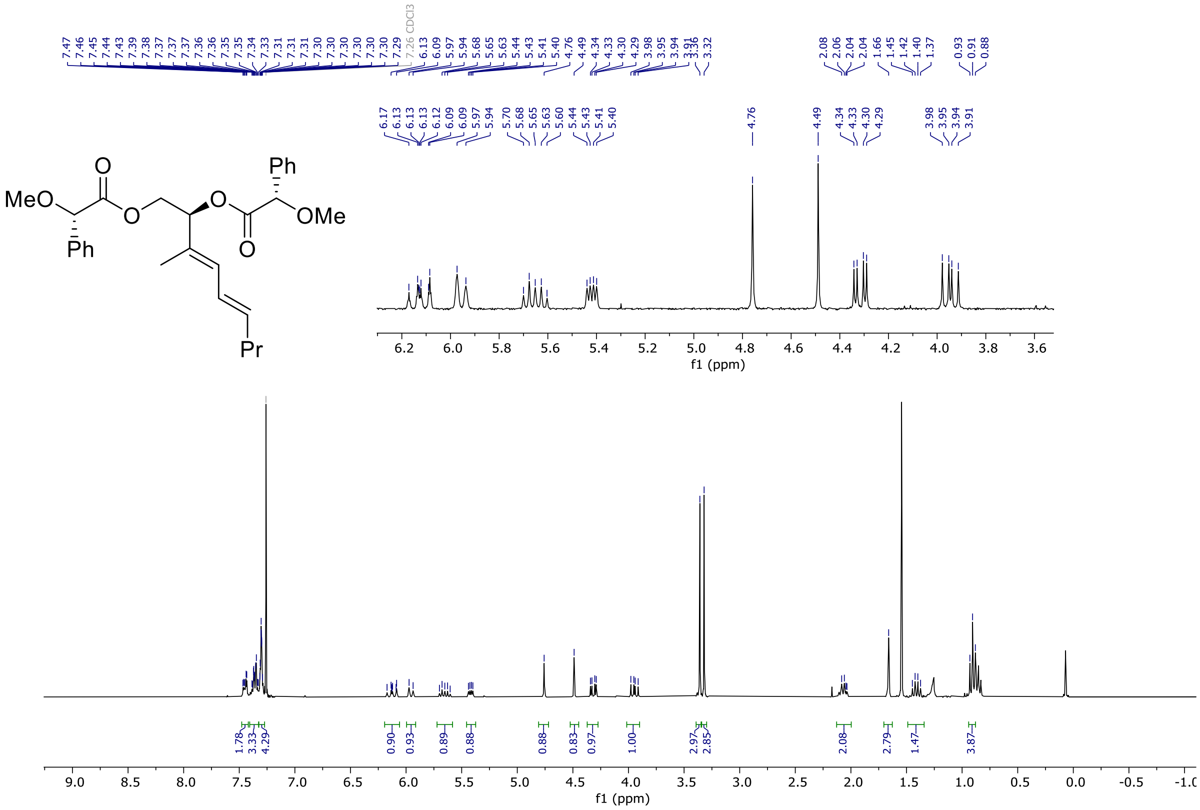

^{13}C NMR (CDCl$_3$, 125MHz) **6m**

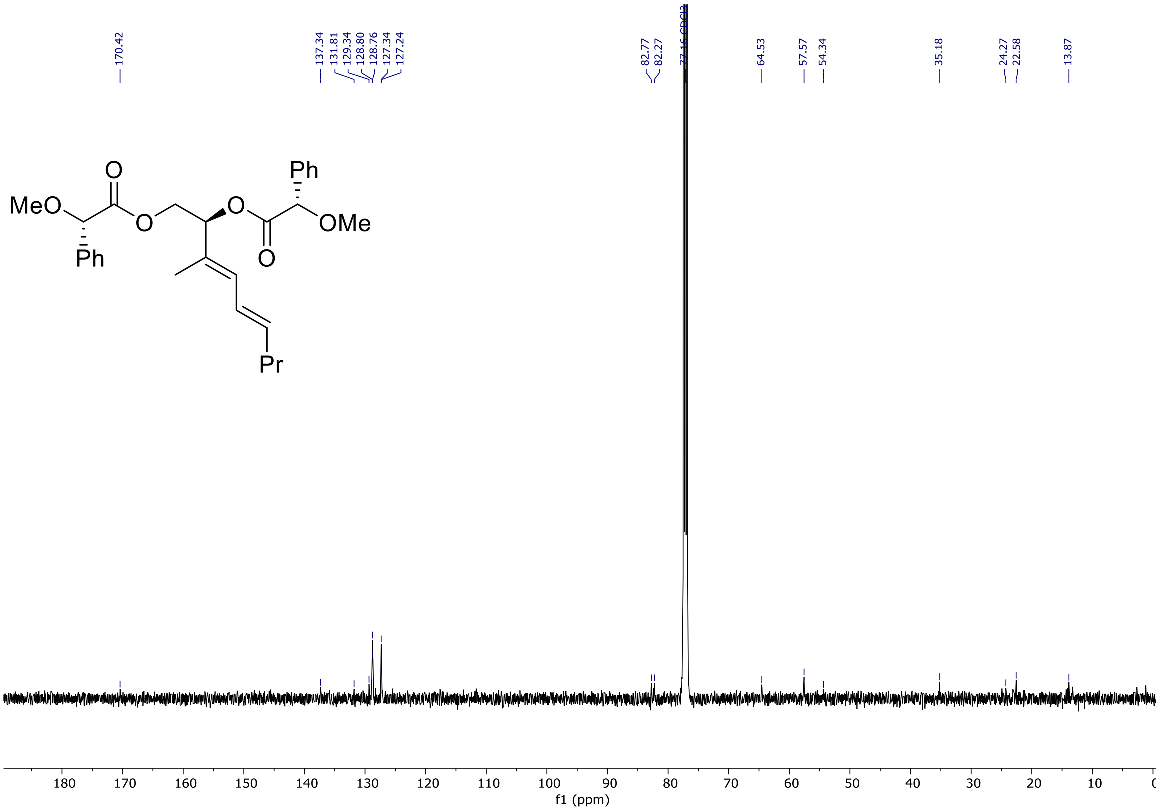

^{1}H NMR (CDCl$_3$, 500 MHz) **8**

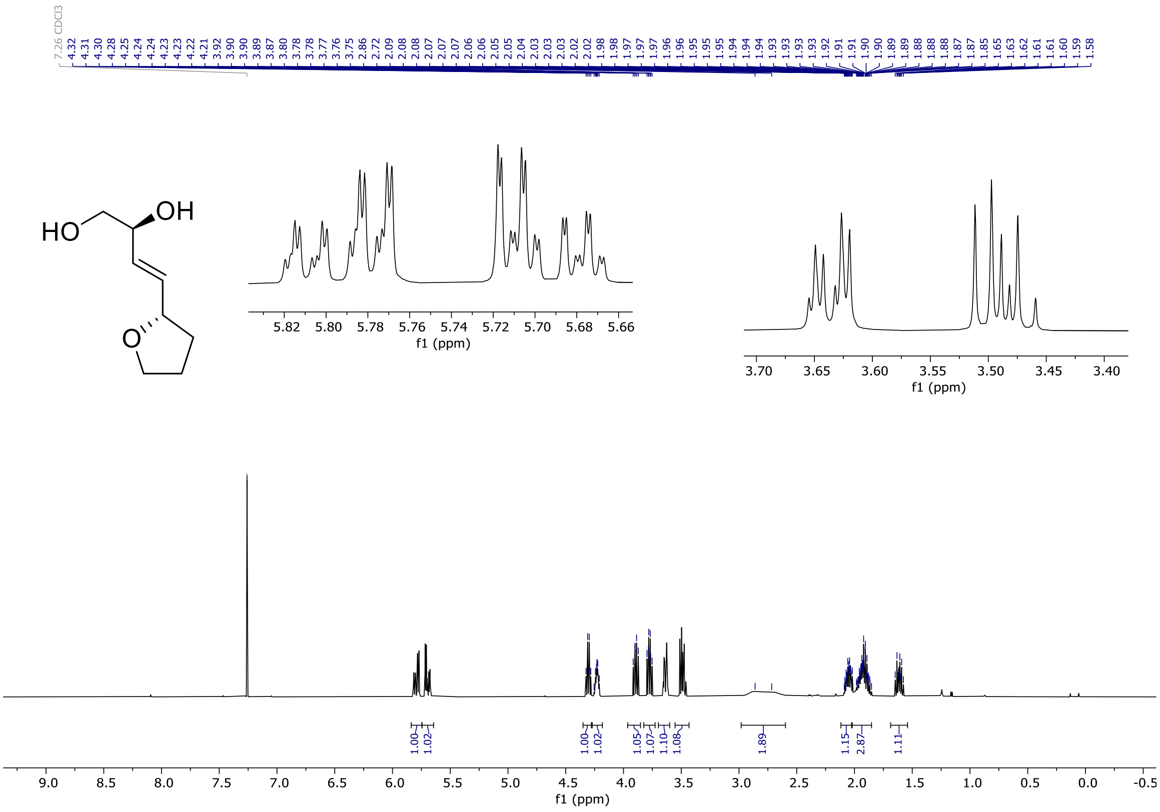

^{13}C NMR ($CDCl_3$, 125MHz) **8**

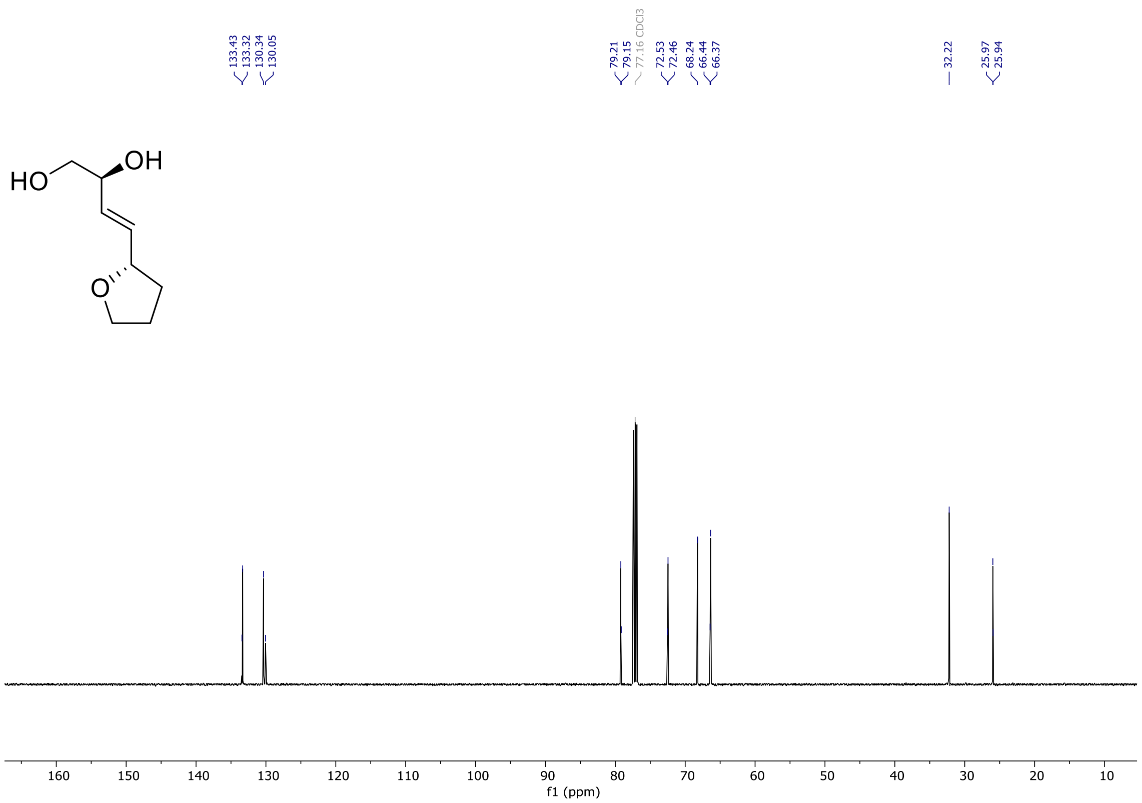

COSY (^{1}H, ^{1}H) **8**

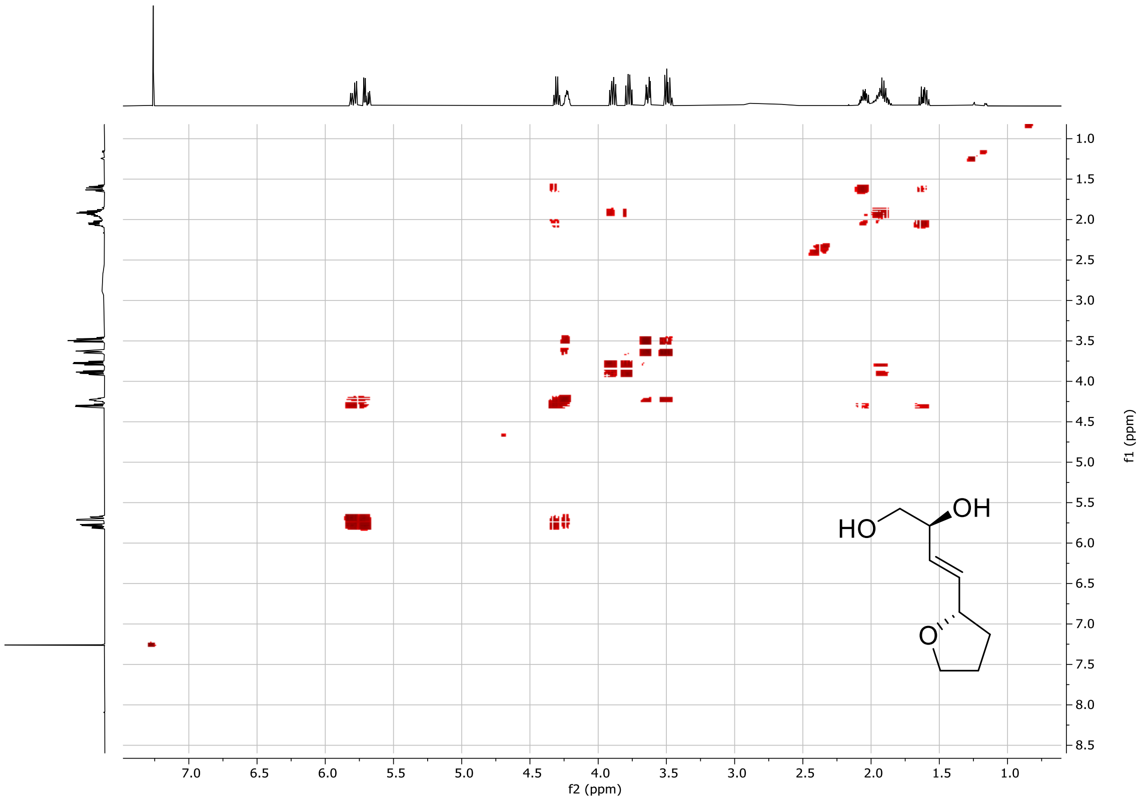

HSQC (^1H, ^{13}C) **8**

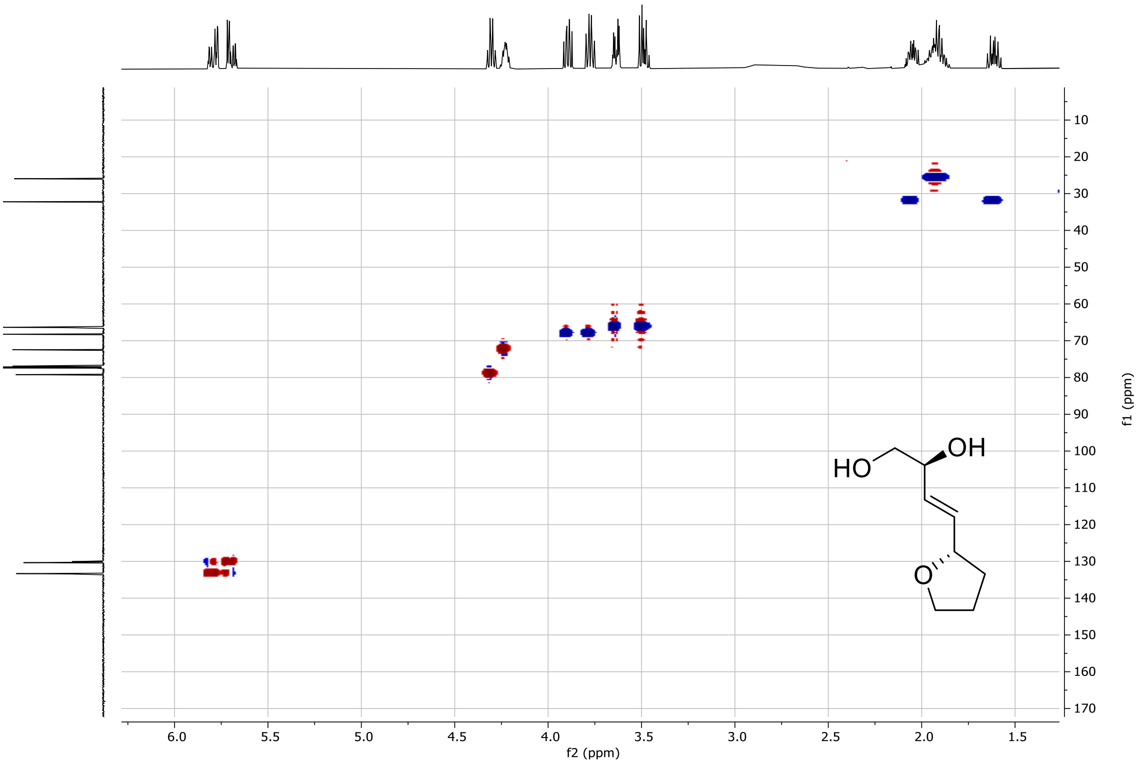

^1H NMR ($CDCl_3$, 500 MHz) **10a:10a'**

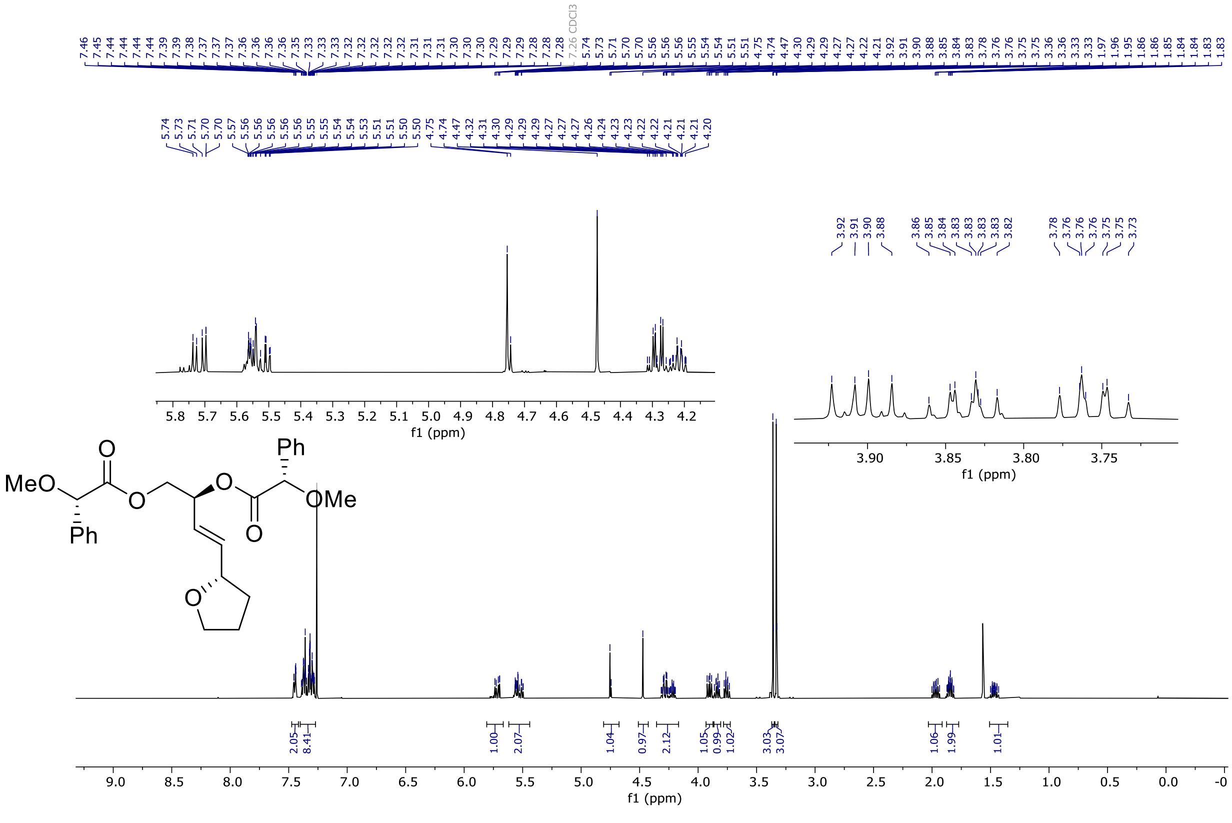

^{13}C NMR ($CDCl_3$, 125MHz) **10a:10a'**

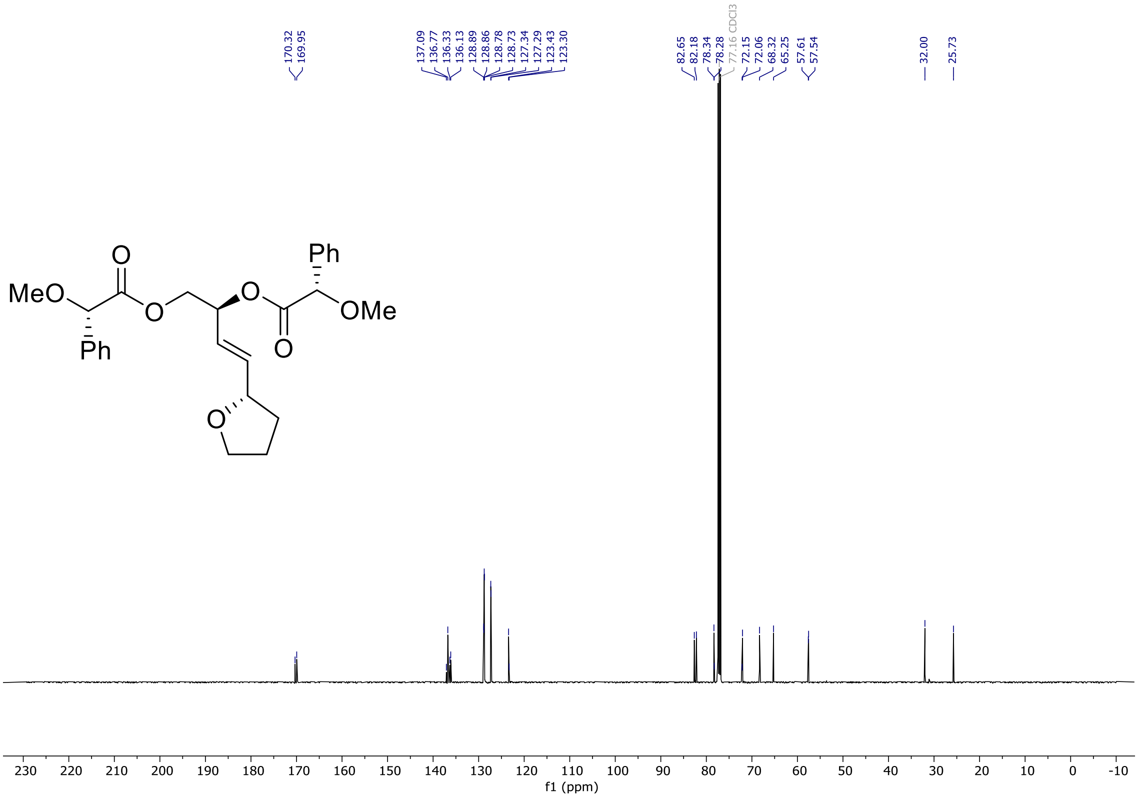

COSY (^{1}H, ^{1}H) **10a:10a'**

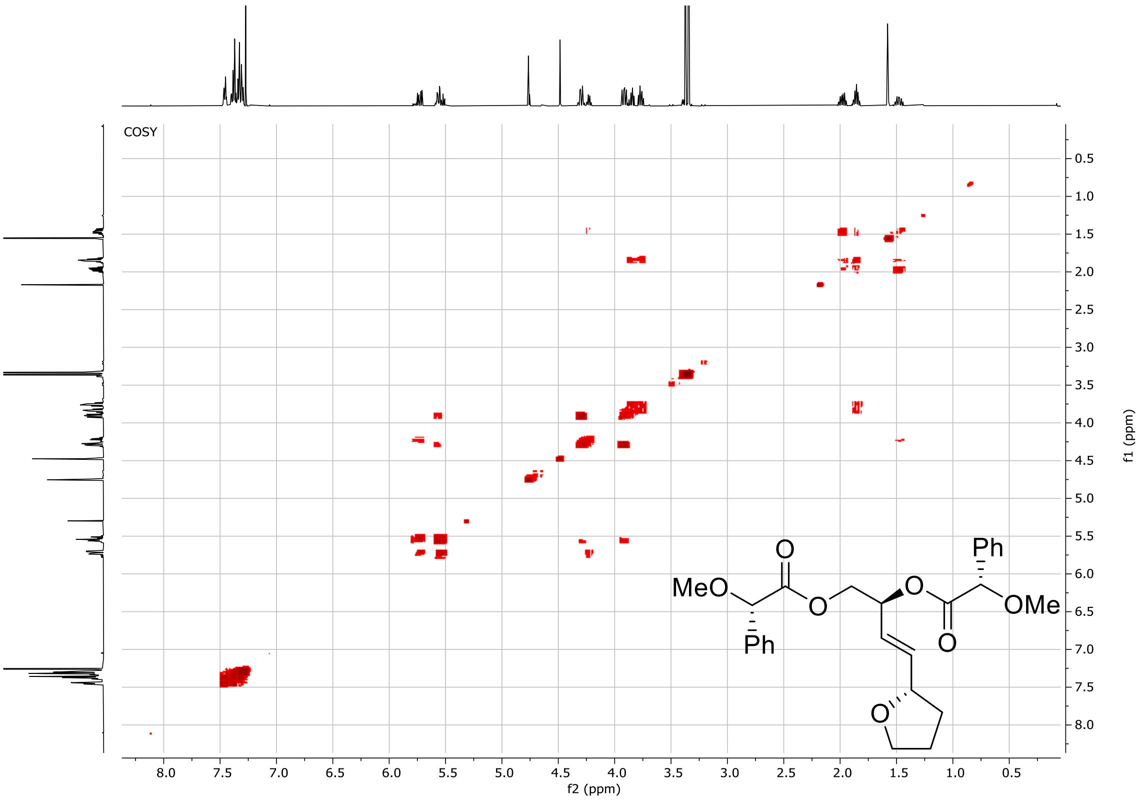

HSQC (^{1}H, ^{13}C) **10a:10a’**

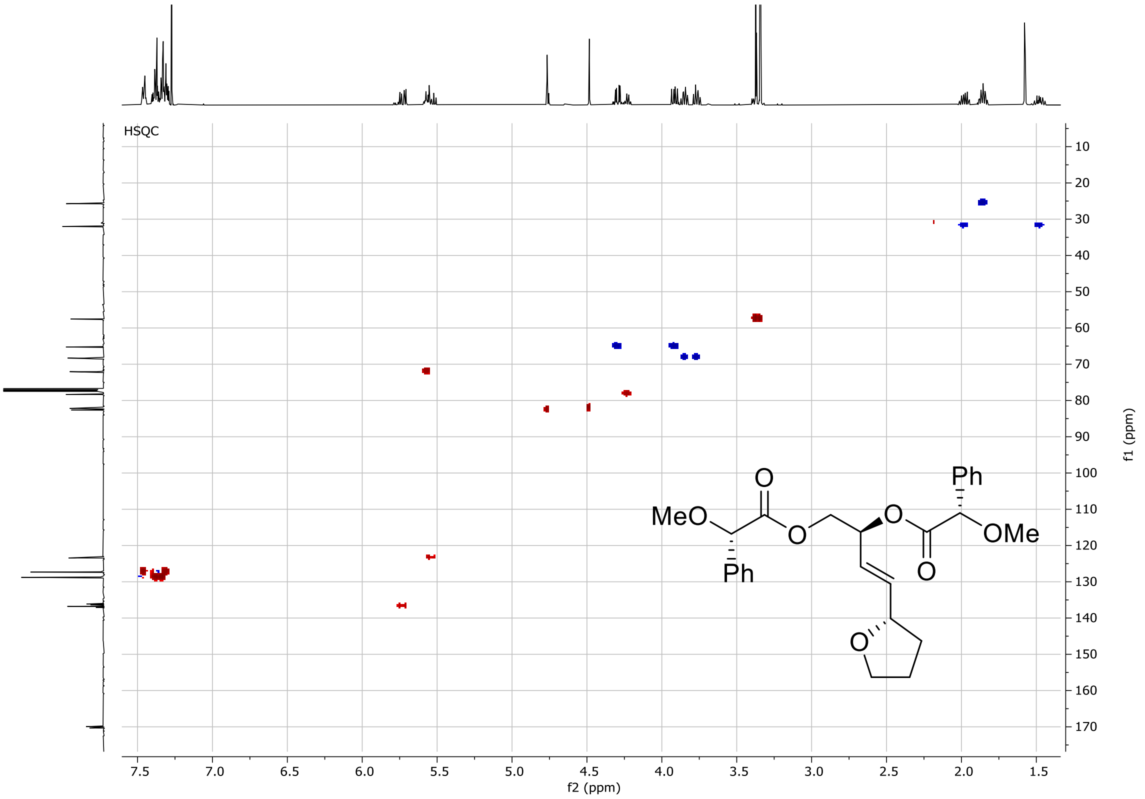

^{1}H NMR ($CDCl_3$, 500 MHz) **9**

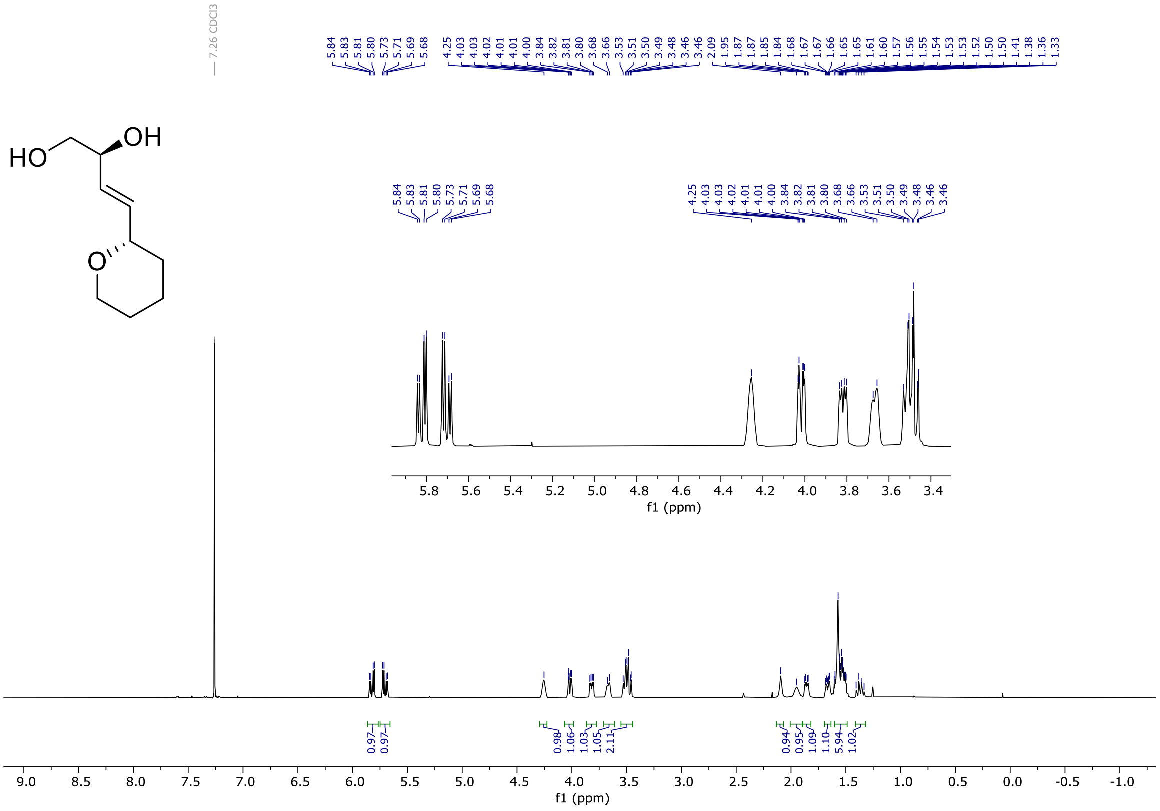

^{13}C NMR ($CDCl_3$, 125MHz) **9**

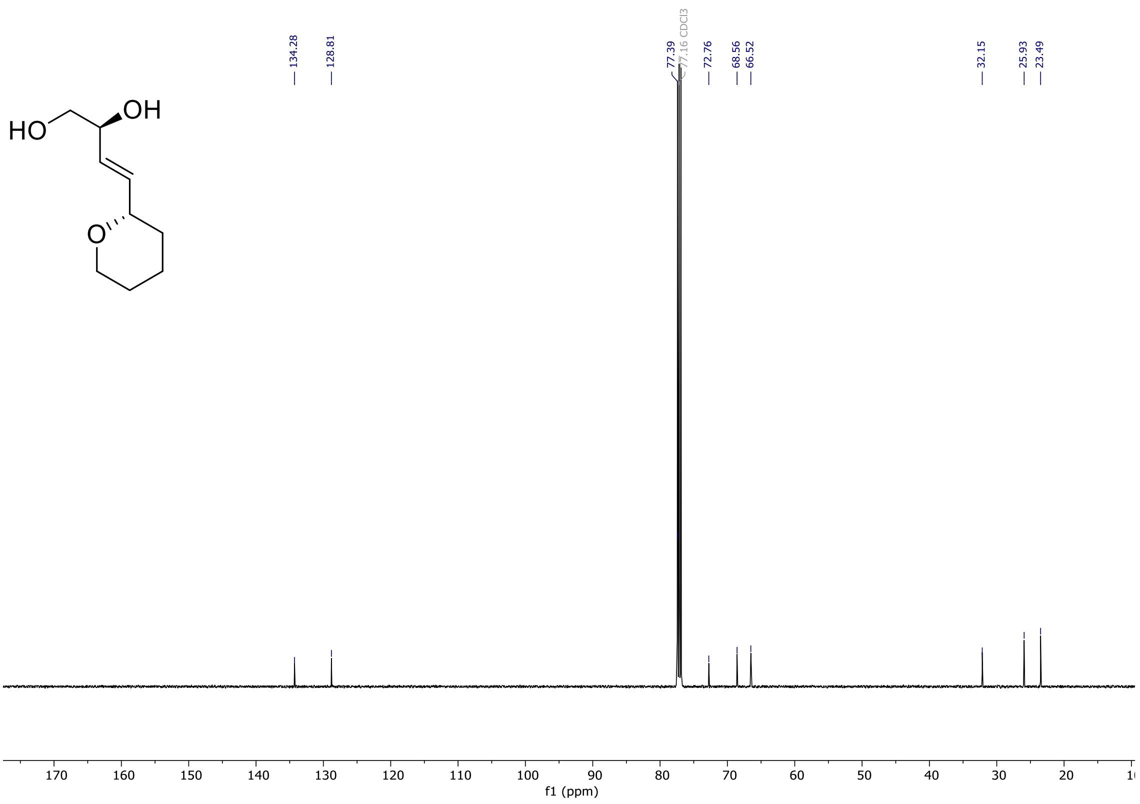

HSQC (^{1}H, ^{13}C) **9**

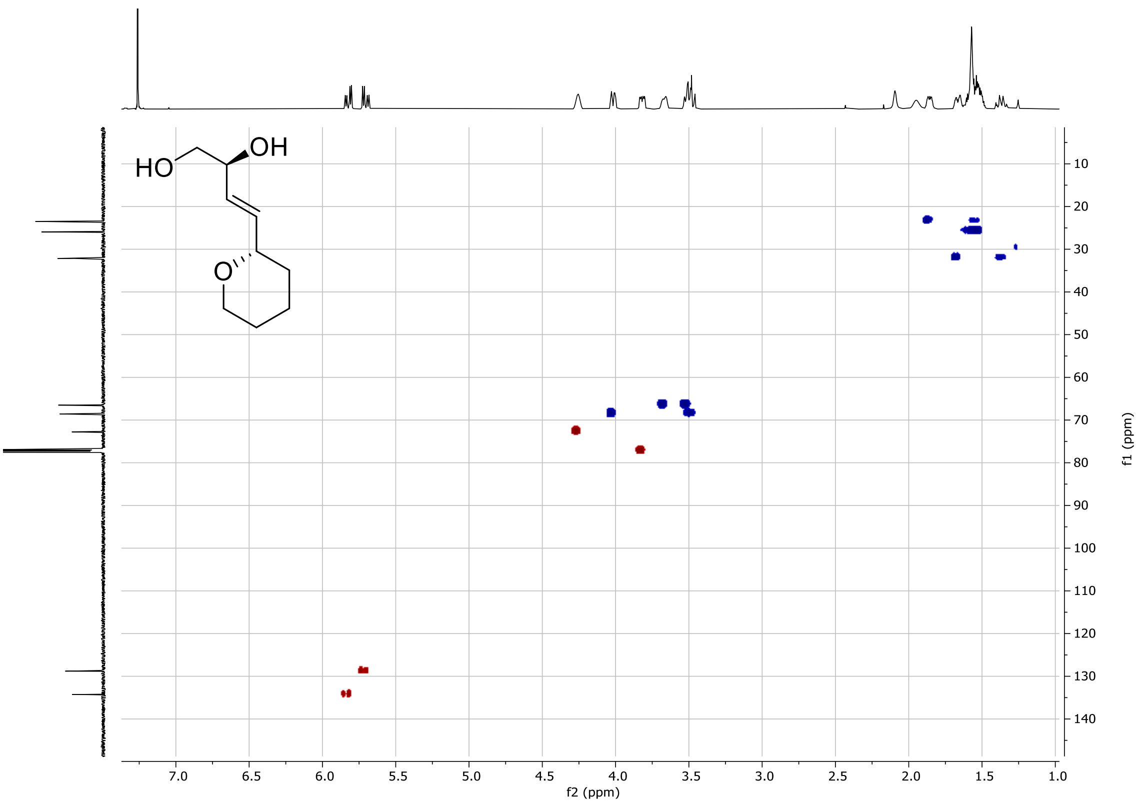

^{1}H NMR ($CDCl_3$, 500 MHz) **10b:10b’**

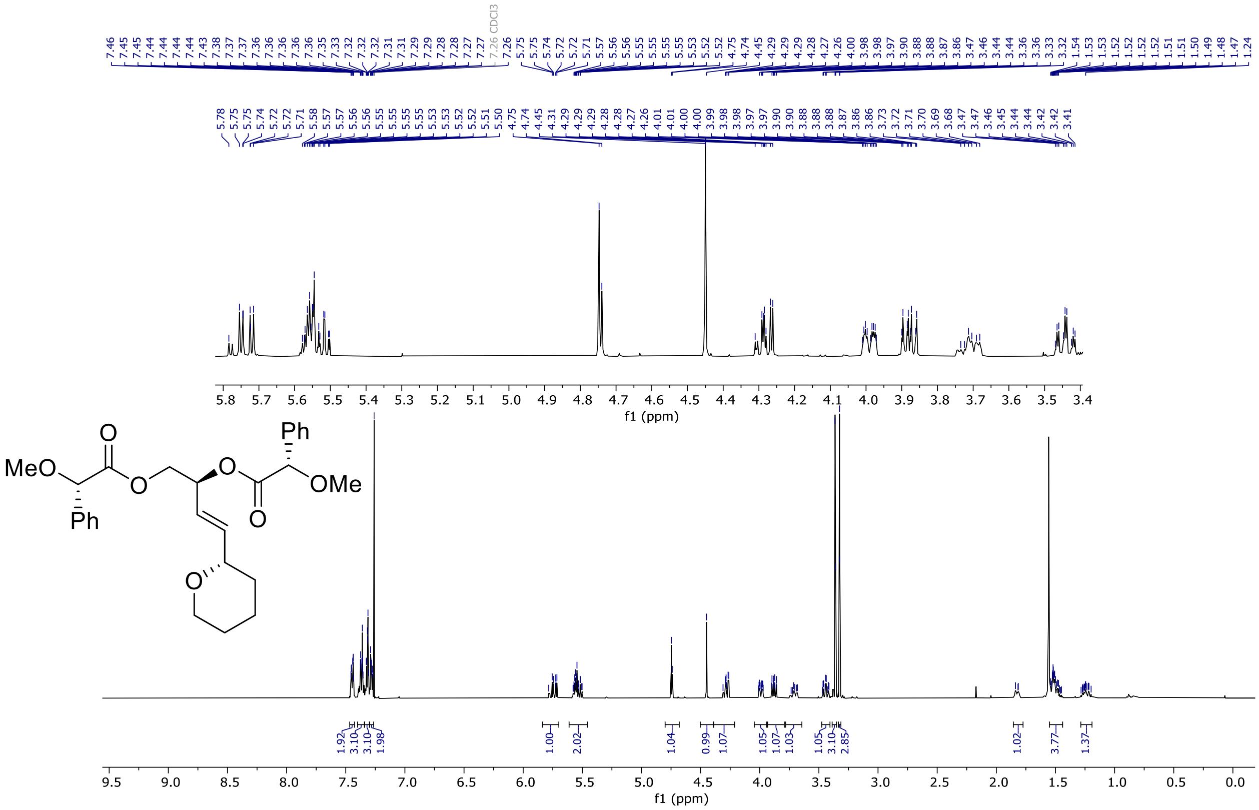

^{13}C NMR ($CDCl_3$, 125MHz) **10b:10b’**

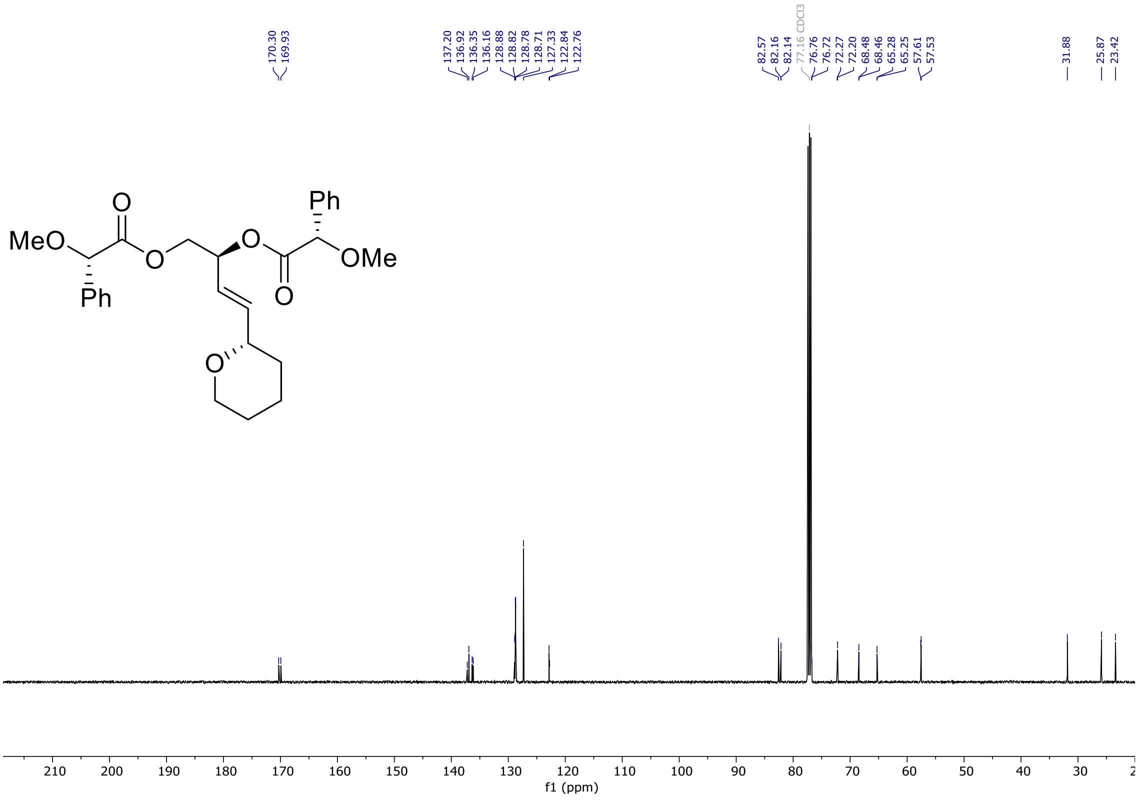

COSY (^{1}H, ^{1}H) **10b:10b'**

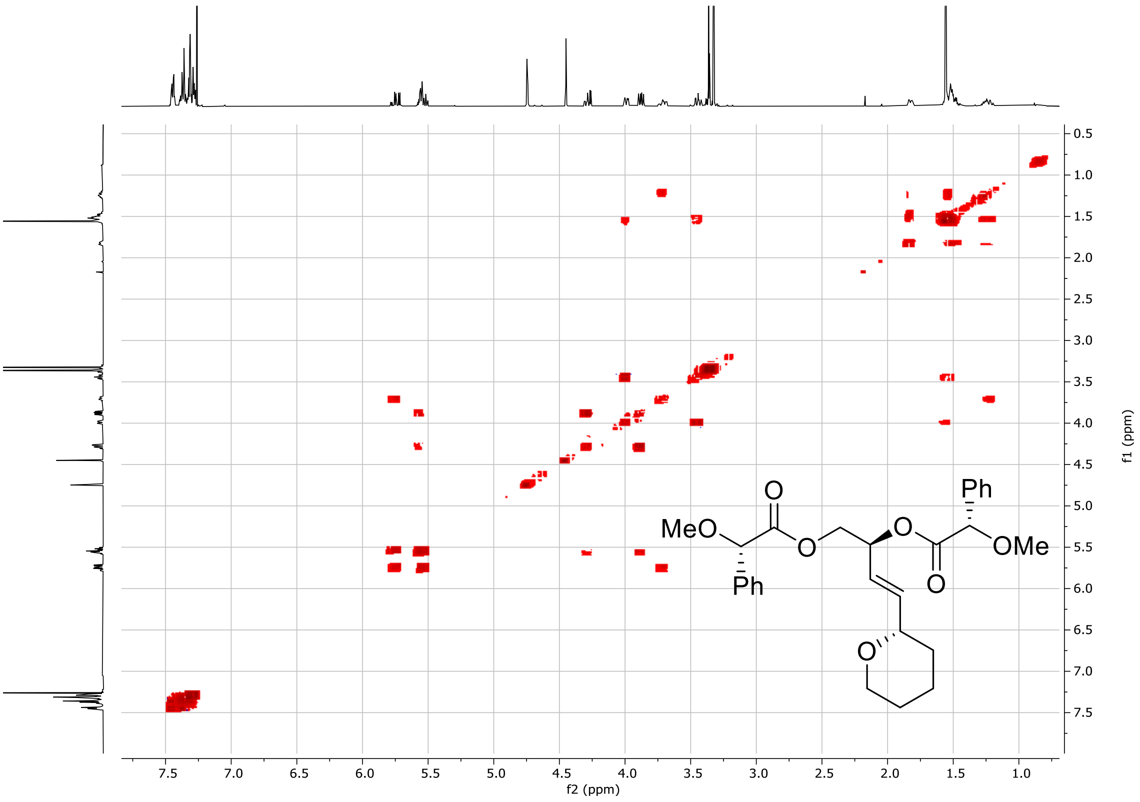

^{1}H NMR ($CDCl_3$, 400 MHz) **12**

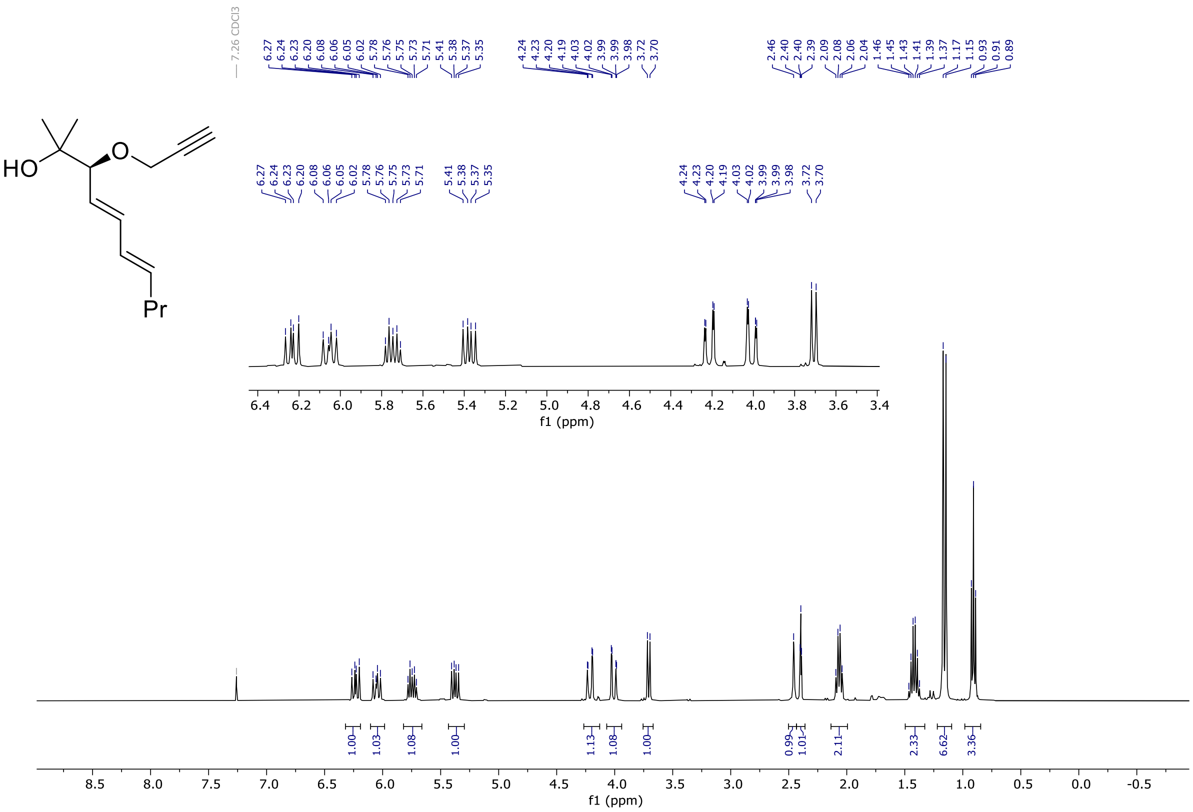

^{13}C NMR ($CDCl_3$, 100MHz) **12**

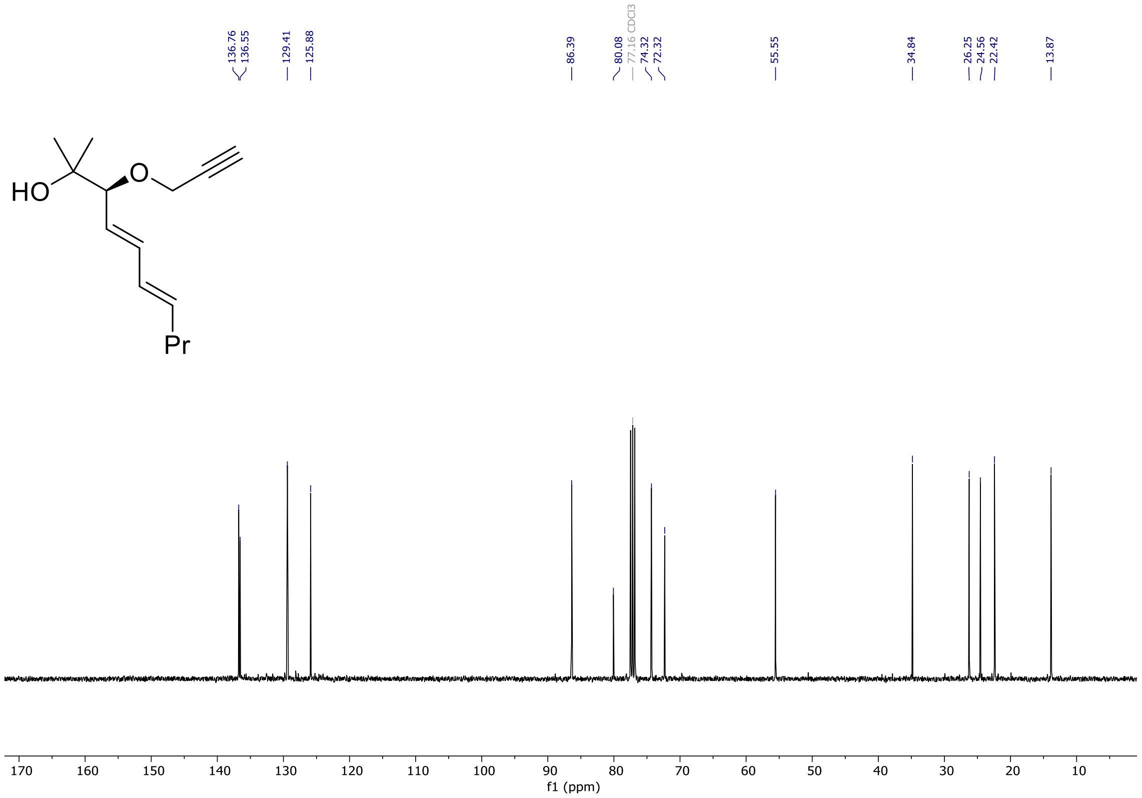

^{1}H NMR ($CDCl_3$, 400 MHz) **13**

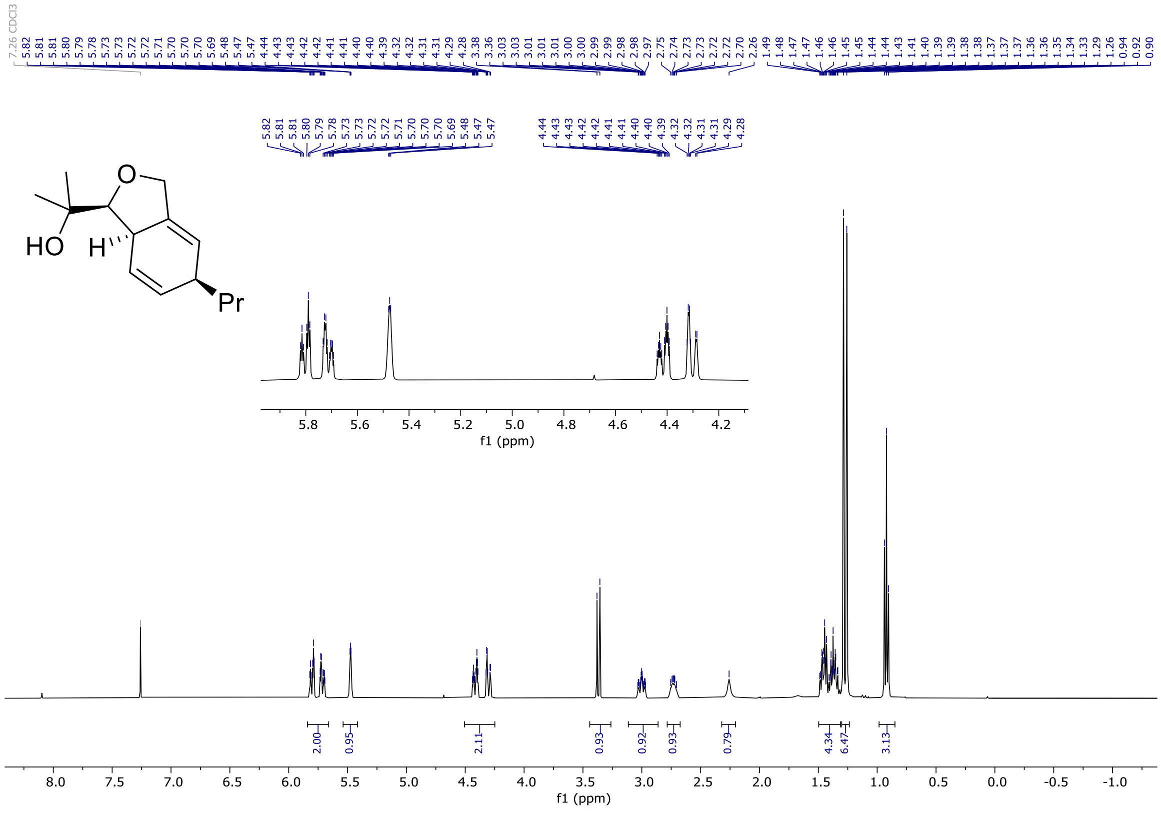

^{13}C NMR (CDCl$_3$, 100MHz) **13**

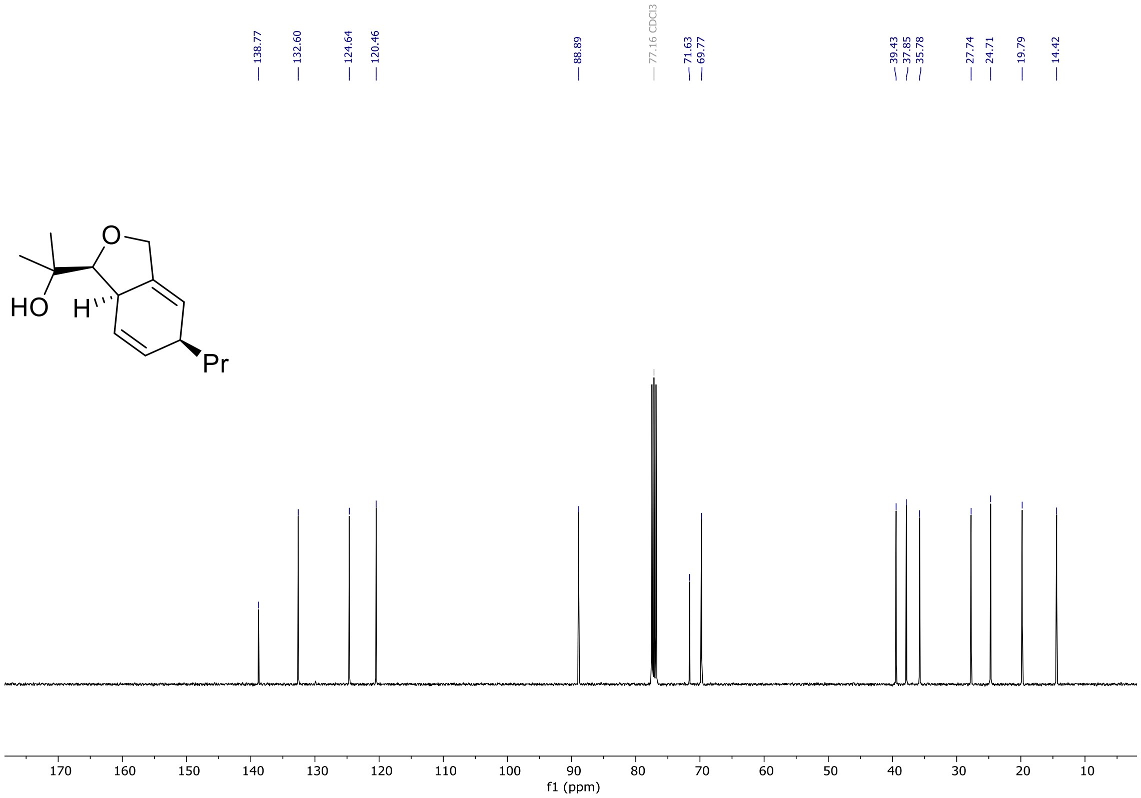

COSY (^{1}H, ^{1}H) **13**

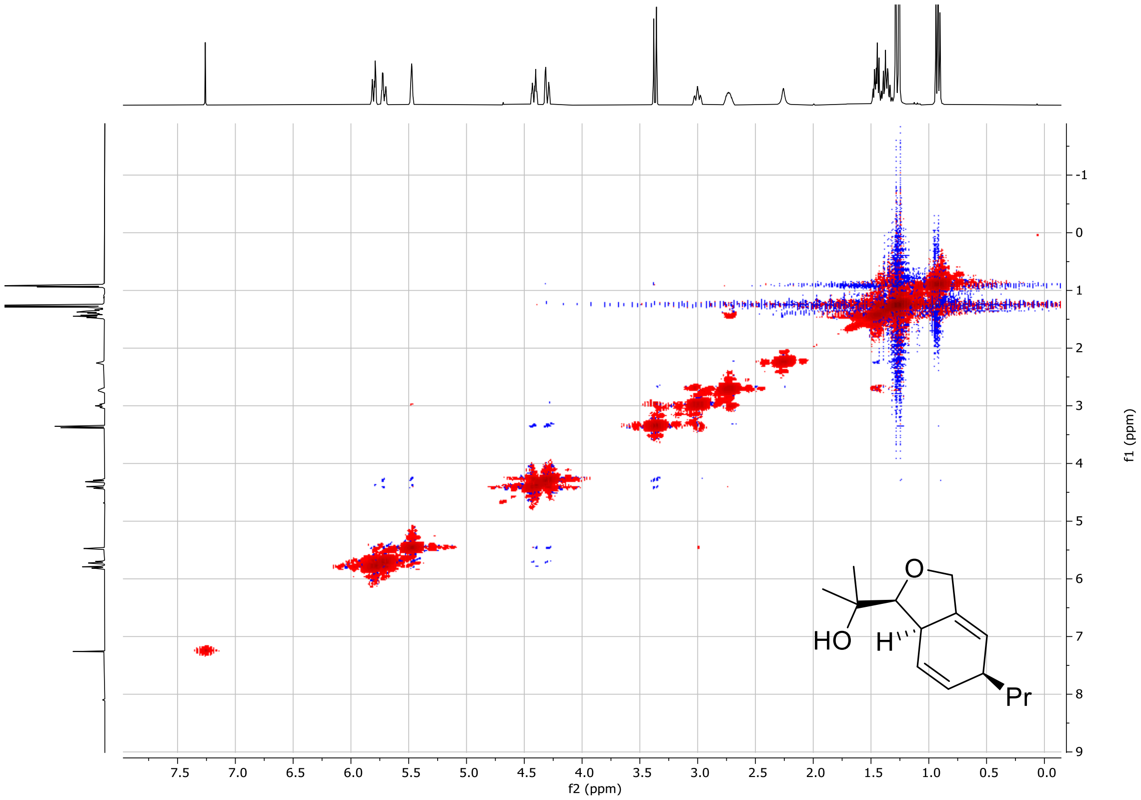

HSQC (^{1}H, ^{13}C) **13**

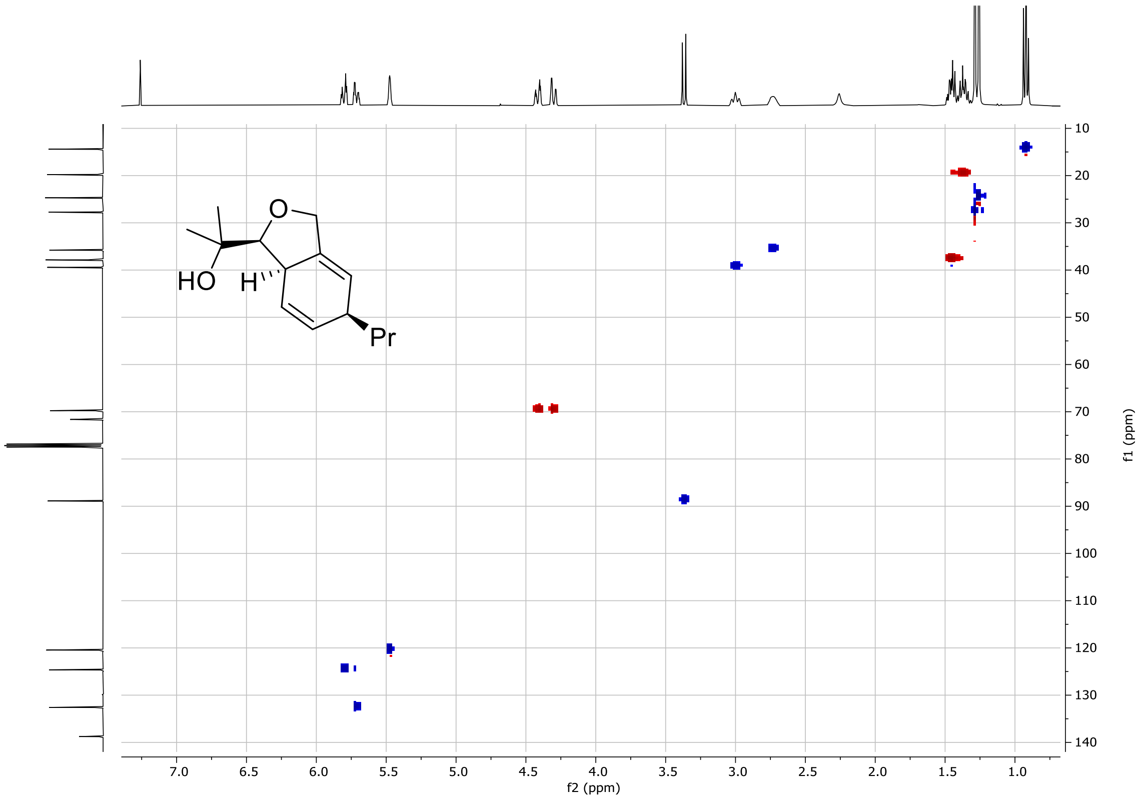

2. Theoretical calculations

2.1 Computational details

DFT calculations were performed with Gaussian 09.[1] All the energies reported were obtained with the M06-2X functional.[2] Solvation was introduced implicitly in all cases through the SMD model,[3] with toluene as the solvent (ε = 2.3741). All geometry optimizations were carried out in solution without symmetry restrictions using 6-31G(d) basis set for all atoms (BS1). Harmonic frequencies were calculated at the same level to characterize the stationary points and to determine the zero-point energies (ZPE). All potential energies were corrected by single-point calculations with the larger 6-311++G(d,p) basis set (BS2).

---

[1] *Gaussian 09, Revision E.01*, M. J. Frisch, G. W. Trucks, H. B. Schlegel, G. E. Scuseria, M. A. Robb, J. R. Cheeseman, G. Scalmani, V. Barone, B. Mennucci, G. A. Petersson, H. Nakatsuji, M. Caricato, X. Li, H. P. Hratchian, A. F. Izmaylov, J. Bloino, G. Zheng, J. L. Sonnenberg, M. Hada, M. Ehara, K. Toyota, R. Fukuda, J. Hasegawa, M. Ishida, T. Nakajima, Y. Honda, O. Kitao, H. Nakai, T. Vreven, J. A. Montgomery, Jr., J. E. Peralta, F. Ogliaro, M. Bearpark, J. J. Heyd, E. Brothers, K. N. Kudin, V. N. Staroverov, T. Keith, R. Kobayashi, J. Normand, K. Raghavachari, A. Rendell, J. C. Burant, S. S. Iyengar, J. Tomasi, M. Cossi, N. Rega, J. M. Millam, M. Klene, J. E. Knox, J. B. Cross, V. Bakken, C. Adamo, J. Jaramillo, R. Gomperts, R. E. Stratmann, O. Yazyev, A. J. Austin, R. Cammi, C. Pomelli, J. W. Ochterski, R. L. Martin, K. Morokuma, V. G. Zakrzewski, G. A. Voth, P. Salvador, J. J. Dannenberg, S. Dapprich, A. D. Daniels, O. Farkas, J. B. Foresman, J. V. Ortiz, J. Cioslowski, and D. J. Fox, Gaussian, Inc., Wallingford CT, 2013.

[2] a) Zhao, Y.; Truhlar, D. G. *Theor. Chem. Acc.*, **2008**, *120*, 215-241.

[3] S. A. V. Marenich, C. J. Cramer, D. G. Truhlar, *J. Phys. Chem. B*, **2009**, *113,* 6378-6396.

2.2 Cartesian coordinates (Å) and energies (hartrees) of all the optimized structures.

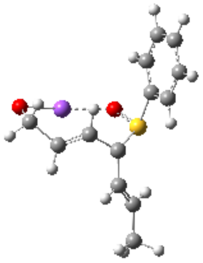

anion model

E(M062X/BS1)$_{toluene}$ = -1214.40956352
G(correction)= 0.191731
E(M062X /BS2)$_{toluene}$ = -1214.64376399

Imaginary frequencies: 0

6	0	-2.18056	1.34813	-0.58293
1	0	-2.72833	1.07399	-1.49151
6	0	-0.92535	0.63373	-0.46735
6	0	-2.75039	2.29697	0.18962
6	0	-0.79268	-0.74088	-0.95091
16	0	0.0572	0.98172	0.88851
1	0	-2.27517	2.59337	1.12519
6	0	-4.00647	3.02938	-0.18466
1	0	0.24065	-1.10389	-1.0521
6	0	-1.75746	-1.66028	-1.15335
8	0	-0.00617	-0.12308	1.99751
6	0	1.72245	0.71587	0.21955
1	0	-3.83782	4.10468	-0.34494
1	0	-4.78086	2.95172	0.59137
1	0	-4.4292	2.62679	-1.1125
1	0	-2.80079	-1.3853	-0.97066
6	0	-1.44354	-3.15336	-1.24227
6	0	2.09646	1.32762	-0.97575
6	0	2.63989	-0.03316	0.94915
1	0	-0.50268	-3.24778	-1.84302
1	0	-2.22602	-3.63348	-1.87855
8	0	-1.35393	-3.70734	0.00706
6	0	3.39701	1.17771	-1.44555
1	0	1.35468	1.88843	-1.53741
6	0	3.94126	-0.18368	0.46988
1	0	2.3136	-0.49435	1.87582
11	0	-0.8743	-2.06238	1.29331
6	0	4.32176	0.42169	-0.72425
1	0	3.68861	1.6442	-2.38245
1	0	4.65687	-0.77775	1.03169
1	0	5.33508	0.30289	-1.09711

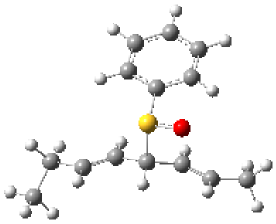

model **Ia**

E(M062X/BS1)$_{toluene}$ = -1017.35305999
G(correction)= 0.241422

E(M062X /BS2)$_{toluene}$ = -1017.55796461

Imaginary frequencies: 0

6	0	1.67947	0.28489	-0.31833
1	0	1.39409	-0.3072	-1.18854
6	0	0.63808	1.21335	0.21531
6	0	2.88444	0.11294	0.22877
6	0	-0.32565	1.73684	-0.78946
1	0	1.08632	2.0242	0.80073
16	0	-0.33011	0.31587	1.58864
1	0	3.16082	0.71088	1.10042
6	0	3.92847	-0.83578	-0.28215
1	0	-0.49633	1.12001	-1.67237
6	0	-1.0444	2.84646	-0.60345
8	0	-1.44712	1.24663	1.98355
6	0	-1.07793	-0.96421	0.5628
1	0	3.52977	-1.39285	-1.13768
6	0	5.21648	-0.10999	-0.67998
1	0	4.16036	-1.5719	0.49905
1	0	-0.87442	3.43517	0.29726
6	0	-2.09309	3.34294	-1.54887
6	0	-0.39604	-2.15412	0.31761
6	0	-2.3585	-0.74325	0.06751
1	0	5.97337	-0.81795	-1.03063
1	0	5.02443	0.61048	-1.48097
1	0	5.63636	0.43784	0.17036
1	0	-2.18629	2.68791	-2.42021
1	0	-1.86391	4.356	-1.89955
1	0	-3.06813	3.39323	-1.05007
6	0	-1.00371	-3.13038	-0.46756
1	0	0.59434	-2.315	0.73622
6	0	-2.95861	-1.72809	-0.71301
1	0	-2.86061	0.18878	0.3125
6	0	-2.28132	-2.91628	-0.98261
1	0	-0.48471	-4.06283	-0.66749
1	0	-3.95784	-1.57132	-1.10811
1	0	-2.75511	-3.68407	-1.5869

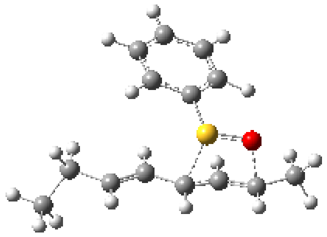

TSIa-*endo*

E(M062X/BS1)$_{toluene}$ = -1017.33526209
G(correction)= 0.241071
E(M062X /BS2)$_{toluene}$ = -1017.53948025

Imaginary frequencies: 1 (-283.9194 cm^{-1})

6	0	-1.38329	-0.97481	-0.42432
1	0	-1.31834	-0.24469	-1.23246
6	0	-0.14773	-1.67518	-0.08693
6	0	-2.54498	-1.13734	0.22194
6	0	0.96436	-1.66681	-0.94141

1	0	-0.23072	-2.47947	0.64369
1	0	-2.60035	-1.86428	1.03555
6	0	-3.80803	-0.39731	-0.09992
1	0	0.96504	-0.97324	-1.78175
6	0	2.165	-2.16082	-0.48105
1	0	-4.13664	0.15839	0.78901
1	0	-3.60773	0.34385	-0.88206
6	0	-4.93225	-1.34002	-0.53962
1	0	2.14368	-2.92357	0.2944
6	0	3.46643	-1.98169	-1.19108
8	0	2.37883	-0.77294	1.12486
1	0	-5.14922	-2.08007	0.23802
1	0	-5.85312	-0.78524	-0.74313
1	0	-4.65018	-1.8815	-1.44773
1	0	3.39628	-1.21218	-1.96537
1	0	3.7869	-2.91888	-1.66221
1	0	4.24277	-1.69118	-0.47646
16	0	0.97414	-0.30622	1.54439
6	0	0.67435	1.19199	0.63734
6	0	-0.50569	1.89823	0.8807
6	0	1.57895	1.61993	-0.33136
6	0	-0.78179	3.04076	0.13709
1	0	-1.20215	1.55505	1.64221
6	0	1.29233	2.76691	-1.06866
1	0	2.49328	1.05295	-0.48258
6	0	0.11438	3.47514	-0.83938
1	0	-1.69775	3.59415	0.3218
1	0	1.99323	3.11041	-1.82396
1	0	-0.10402	4.36865	-1.41613

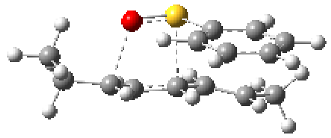

TSIa-*exo*

E(M062X/BS1)$_{toluene}$ = -1017.33221755
G(correction)= 0.240672
E(M062X /BS2)$_{toluene}$ = --1017.53586428

Imaginary frequencies: 1(-278.0624 cm^{-1})

6	0	-1.48589	1.72543	-0.53101
1	0	-1.88136	2.62568	-0.06154
6	0	-0.10198	1.50378	-0.51926
6	0	-2.31467	0.64477	-0.72572
6	0	0.88321	2.52252	-0.17138
1	0	0.27531	0.68109	-1.12864
1	0	-1.91302	-0.21168	-1.268
6	0	-3.80325	0.67763	-0.56288
8	0	-1.71106	-0.37604	1.08603
1	0	0.53145	3.37823	0.40614
6	0	2.17505	2.43883	-0.51769
1	0	-4.24152	1.02181	-1.51135
1	0	-4.06899	1.4238	0.19442
6	0	-4.39244	-0.68414	-0.20219
16	0	-0.25043	0.01479	1.37196
1	0	2.50135	1.5761	-1.10074

6	0	3.21826	3.45344	-0.17288
1	0	-5.48424	-0.63434	-0.1516
1	0	-4.1261	-1.43398	-0.95596
1	0	-4.01016	-1.0248	0.76213
6	0	0.74593	-1.21708	0.56022
1	0	2.79714	4.27628	0.4117
1	0	3.67368	3.87111	-1.07859
1	0	4.03056	2.99923	0.4073
6	0	0.1454	-2.26923	-0.12822
6	0	2.13578	-1.0829	0.59554
6	0	0.94832	-3.1966	-0.78682
1	0	-0.93809	-2.34819	-0.12685
6	0	2.9272	-2.01583	-0.06764
1	0	2.59295	-0.2588	1.13888
6	0	2.3363	-3.0722	-0.76009
1	0	0.48718	-4.02254	-1.32052
1	0	4.00852	-1.91859	-0.04217
1	0	2.95739	-3.7992	-1.27415

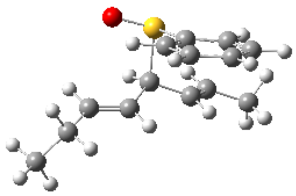

model **Ib**

E(M062X/BS1)$_{toluene}$ = -1017.35307862
G(correction)= 0.241422
E(M062X /BS2)$_{toluene}$ = -1017.5579646

Imaginary frequencies: 0

6	0	-1.38791	0.55076	-0.47954
1	0	-1.04177	0.1121	-1.41604
6	0	-0.39715	1.35658	0.28328
6	0	-2.61054	0.26857	-0.02319
6	0	0.69241	1.96898	-0.53408
1	0	-0.88967	2.10264	0.91729
16	0	0.38476	0.25577	1.62722
1	0	-2.9332	0.70146	0.92442
6	0	-3.60311	-0.60697	-0.72807
1	0	1.00026	1.41096	-1.41902
6	0	1.31282	3.10849	-0.2223
8	0	-0.76947	-0.41401	2.32862
6	0	1.10345	-0.98627	0.53664
1	0	-3.15828	-1.00202	-1.64879
1	0	-3.83331	-1.46966	-0.08876
6	0	-4.90253	0.13778	-1.04585
1	0	0.99449	3.65505	0.66699
6	0	2.42707	3.71527	-1.01687
6	0	0.37382	-2.14398	0.28936
6	0	2.37072	-0.79199	-0.009
1	0	-5.62854	-0.52405	-1.52787
1	0	-4.71181	0.98266	-1.71508
1	0	-5.36057	0.53089	-0.13193
1	0	2.68351	3.09679	-1.88178
1	0	2.1528	4.7137	-1.37674
1	0	3.32585	3.83705	-0.40106

6	0	0.9163	-3.1176	-0.54554
1	0	-0.59782	-2.26083	0.76142
6	0	2.90111	-1.76945	-0.84711
1	0	2.93481	0.10876	0.21996
6	0	2.17405	-2.92804	-1.11613
1	0	0.35887	-4.02672	-0.75015
1	0	3.88647	-1.63152	-1.2817
1	0	2.59448	-3.69022	-1.76529

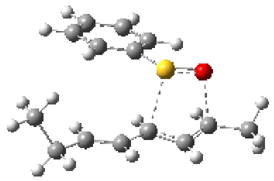

TSIb-*exo*

E(M062X/BS1)$_{toluene}$ = -1017.33261047
G(correction)= 0.241751
E(M062X /BS2)$_{toluene}$ = -1017.5366592

Imaginary frequencies: 1 (-277.6454 cm^{-1})

6	0	-0.87364	-2.10878	-0.34414
1	0	-0.80338	-3.05016	0.2021
6	0	0.37169	-1.40179	-0.62014
6	0	-2.07318	-1.6363	-0.71134
6	0	1.643	-1.98961	-0.58507
1	0	0.26601	-0.48865	-1.20792
16	0	0.82828	-0.0485	1.3313
1	0	-2.11555	-0.69067	-1.25661
6	0	-3.38869	-2.28502	-0.40712
1	0	1.76187	-2.97198	-0.12911
6	0	2.7387	-1.16661	-0.71199
8	0	2.34399	-0.02161	1.07582
6	0	0.16566	1.39805	0.53426
1	0	-3.22243	-3.25433	0.07585
1	0	-3.92433	-2.48007	-1.34536
6	0	-4.25986	-1.39557	0.48687
1	0	2.60989	-0.2293	-1.25005
6	0	4.1452	-1.59374	-0.45441
6	0	-1.21576	1.60333	0.56622
6	0	1.0053	2.28055	-0.14194
1	0	-3.78529	-1.24363	1.4616
1	0	-5.24256	-1.84716	0.6532
1	0	-4.41454	-0.41236	0.02869
1	0	4.18063	-2.51673	0.13059
1	0	4.68156	-1.75774	-1.39724
1	0	4.67663	-0.81172	0.09585
6	0	-1.75579	2.70496	-0.09093
1	0	-1.85997	0.90987	1.10275
6	0	0.45308	3.37999	-0.79342
1	0	2.0758	2.09637	-0.136
6	0	-0.92431	3.59335	-0.7717
1	0	-2.82879	2.87178	-0.06839
1	0	1.10159	4.07521	-1.3187
1	0	-1.34992	4.45331	-1.27953

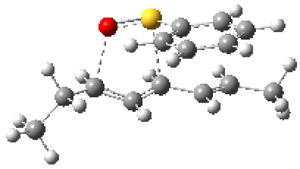

TSIb-*endo*

E(M062X/BS1)$_{toluene}$ = -1017.33522317
G(correction)= 0.241590
E(M062X /BS2)$_{toluene}$ = -1017.53921155

Imaginary frequencies: 1(-278.4322cm^{-1})

6	0	-1.36708	0.9029	-0.69634
1	0	-1.0671	0.29073	-1.54661
6	0	-0.38921	1.67511	-0.0535
6	0	-2.51563	0.57653	-0.00813
6	0	0.93308	1.89356	-0.63185
1	0	-0.71931	2.40462	0.68596
16	0	-0.10905	0.04604	1.68706
1	0	-2.86407	1.25801	0.76644
6	0	-3.51723	-0.42732	-0.48761
1	0	1.21374	1.24222	-1.46048
6	0	1.80842	2.80091	-0.18056
8	0	-1.55415	-0.47266	1.57847
6	0	0.8557	-1.04656	0.66865
1	0	-3.04591	-1.09878	-1.21466
1	0	-3.82716	-1.0392	0.36688
6	0	-4.74286	0.24706	-1.11271
1	0	1.51818	3.43812	0.65631
6	0	3.1802	3.0058	-0.74
6	0	0.21951	-2.04096	-0.07062
6	0	2.23873	-0.86704	0.58993
1	0	-5.478	-0.49848	-1.43088
1	0	-4.45874	0.84124	-1.98679
1	0	-5.23016	0.91599	-0.39558
1	0	3.38482	2.30576	-1.55516
1	0	3.30377	4.0264	-1.12124
1	0	3.94346	2.86482	0.03481
6	0	0.97776	-2.86205	-0.90208
1	0	-0.85582	-2.15938	0.02749
6	0	2.98496	-1.69167	-0.24608
1	0	2.72526	-0.0871	1.17108
6	0	2.35701	-2.68791	-0.99347
1	0	0.48906	-3.64261	-1.4779
1	0	4.06054	-1.55727	-0.31304
1	0	2.94535	-3.33064	-1.64116

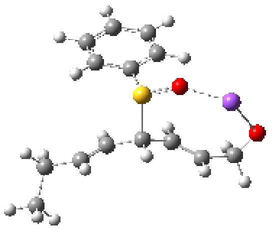

model **IIa**

E(M062X/BS1)$_{toluene}$ = -1254.24528195
G(correction)= 0.231698

E(M062X /BS2)$_{toluene}$ = -1254.48352479

Imaginary frequencies: 0

6	0	-1.67783	-0.95887	-0.21929
1	0	-1.76287	-0.3283	-1.10441
6	0	-0.294	-1.16901	0.30849
6	0	-2.77413	-1.47651	0.33824
6	0	0.78179	-1.21729	-0.71935
1	0	-0.25117	-2.0467	0.96383
1	0	-2.67094	-2.11189	1.22115
6	0	-4.16836	-1.28446	-0.18042
1	0	0.74838	-0.45111	-1.49704
6	0	1.77687	-2.11334	-0.71622
1	0	-4.77925	-0.80638	0.59677
1	0	-4.14987	-0.60364	-1.03888
6	0	-4.81562	-2.61478	-0.57523
1	0	1.80073	-2.87284	0.073
6	0	3.02069	-2.04329	-1.58765
1	0	-4.85002	-3.30191	0.2768
1	0	-5.84047	-2.46249	-0.92639
1	0	-4.24805	-3.09996	-1.37503
1	0	2.76552	-1.40761	-2.46802
1	0	3.19394	-3.05963	-2.00848
8	0	4.06491	-1.57774	-0.84051
11	0	3.36346	-0.61151	0.87312
16	0	0.0642	0.22196	1.55545
8	0	1.55802	0.15181	1.85773
6	0	-0.1579	1.64273	0.47643
6	0	-1.42957	2.20056	0.35043
6	0	0.93936	2.18237	-0.18576
6	0	-1.60403	3.30034	-0.48459
1	0	-2.27027	1.77841	0.89469
6	0	0.75458	3.28641	-1.01355
1	0	1.92384	1.74653	-0.04678
6	0	-0.51472	3.84046	-1.16592
1	0	-2.58968	3.74133	-0.59512
1	0	1.60329	3.71487	-1.53747
1	0	-0.65448	4.70205	-1.81155

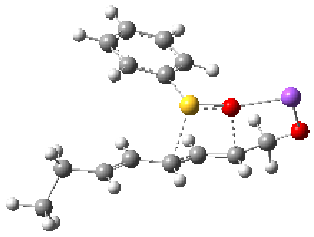

TSIIa-*endo*

E(M062X/BS1)$_{toluene}$ = -1254.22066442
G(correction)= 0.229115
E(M062X /BS2)$_{toluene}$ = -1254.46076862

Imaginary frequencies: 1 (-235.504 cm^{-1})

6	0	1.85281	-1.09614	0.43367
1	0	1.98311	-0.3448	1.21322
6	0	0.49766	-1.57541	0.20511
6	0	2.911	-1.50786	-0.27882

6	0	-0.55509	-1.31443	1.07631
1	0	0.37759	-2.37113	-0.52871
16	0	-0.44512	0.01212	-1.51656
1	0	2.75822	-2.25744	-1.05863
6	0	4.31812	-1.03888	-0.07417
1	0	-0.40131	-0.60146	1.88609
6	0	-1.84629	-1.65729	0.71515
8	0	-1.89962	-0.25623	-1.02296
6	0	0.07928	1.43069	-0.59215
1	0	4.68983	-0.59802	-1.00896
1	0	4.33562	-0.24761	0.68362
6	0	5.24674	-2.18654	0.3365
1	0	-1.98665	-2.43091	-0.04056
6	0	-3.09606	-1.28217	1.4789
11	0	-4.05114	-0.33884	-1.0993
6	0	1.33145	1.99274	-0.85795
6	0	-0.72206	1.92991	0.43346
1	0	5.24478	-2.98144	-0.41689
1	0	6.27595	-1.83412	0.45195
1	0	4.92434	-2.6235	1.28638
1	0	-2.8596	-0.32829	2.01496
1	0	-3.10978	-2.04295	2.30576
8	0	-4.23292	-1.26057	0.74881
6	0	1.77622	3.06215	-0.0874
1	0	1.95244	1.59615	-1.65802
6	0	-0.26563	3.00215	1.19639
1	0	-1.68649	1.46731	0.62318
6	0	0.9808	3.56877	0.94037
1	0	2.74797	3.50273	-0.29028
1	0	-0.88709	3.39266	1.99705
1	0	1.33198	4.40438	1.53788

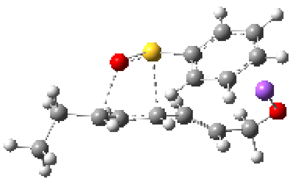

TSIIa-*exo*

E(M062X/BS1)$_{toluene}$ = -1254.20011545
G(correction)= 0.230344
E(M062X /BS2)$_{toluene}$ = -1254.43678461

Imaginary frequencies: 1(-260.8508 cm^{-1})

6	0	2.35815	1.22552	-0.12888
1	0	2.76628	1.86895	-0.90707
6	0	0.97688	1.19739	0.09795
6	0	3.09695	0.16587	0.34567
6	0	-0.01816	2.16931	-0.33643
1	0	0.66162	0.59501	0.95445
16	0	0.74553	-0.74414	-1.37341
1	0	2.71749	-0.38176	1.20666
6	0	4.5228	-0.09744	-0.01666
1	0	0.11811	2.65786	-1.30151
6	0	-1.07966	2.46731	0.43191
8	0	2.03114	-1.37061	-0.80951
6	0	-0.63321	-1.42162	-0.47052

1	0	4.78594	0.46858	-0.91671
1	0	4.61997	-1.16111	-0.26226
6	0	5.47449	0.25651	1.1313
1	0	-1.1468	1.98558	1.41707
6	0	-2.30583	3.27535	0.05974
6	0	-0.50567	-1.87119	0.8462
6	0	-1.86795	-1.50312	-1.12648
1	0	6.50982	0.02792	0.86151
1	0	5.41313	1.32103	1.37782
1	0	5.22864	-0.31267	2.034
1	0	-2.10891	3.73352	-0.93511
1	0	-2.37134	4.13527	0.76438
8	0	-3.40227	2.45983	0.09483
6	0	-1.6184	-2.37654	1.5145
1	0	0.47063	-1.84104	1.32328
6	0	-2.97408	-2.03586	-0.4591
1	0	-1.9576	-1.1776	-2.16043
11	0	-2.99428	0.45952	0.43183
6	0	-2.85504	-2.46026	0.86784
1	0	-1.51961	-2.7238	2.53859
1	0	-3.92425	-2.12589	-0.9791
1	0	-3.71316	-2.87939	1.38406

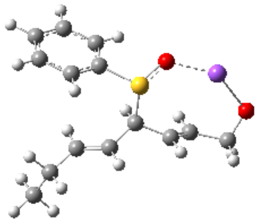

model **IIb**

E(M062X/BS1)$_{toluene}$ = -1254.24474296
G(correction)= 0.231850
E(M062X /BS2)$_{toluene}$ = -1254.48320074

Imaginary frequencies: 0

6	0	-0.54819	1.73288	-0.51287
1	0	-0.31189	2.28399	-1.42385
6	0	0.49343	0.77783	-0.02887
6	0	-1.72336	1.91604	0.09211
6	0	1.90349	1.24467	-0.18635
1	0	0.30049	0.44153	0.99655
1	0	-1.93944	1.3555	1.00489
6	0	-2.7941	2.85587	-0.37485
1	0	2.16505	1.69482	-1.14713
6	0	2.84233	1.10958	0.75818
1	0	-3.69785	2.27531	-0.60572
1	0	-2.47858	3.34183	-1.3048
6	0	-3.13367	3.9084	0.68347
1	0	2.56535	0.64979	1.71247
6	0	4.33303	1.32174	0.54819
1	0	-3.93301	4.56945	0.33583
1	0	-3.46798	3.43553	1.61294
1	0	-2.25792	4.52192	0.91545
1	0	4.71338	1.91697	1.40904
1	0	4.44741	1.99203	-0.33643
8	0	4.93594	0.10417	0.41698

11	0	3.54763	-1.35243	-0.16158
16	0	0.33669	-0.81931	-1.04603
8	0	1.42064	-1.749	-0.49589
6	0	-1.19893	-1.41172	-0.33751
6	0	-2.40164	-1.11849	-0.97473
6	0	-1.15756	-2.1446	0.84499
6	0	-3.59237	-1.55324	-0.39725
1	0	-2.41012	-0.55757	-1.90514
6	0	-2.35318	-2.57731	1.40977
1	0	-0.19698	-2.37625	1.29645
6	0	-3.56729	-2.27777	0.79244
1	0	-4.53813	-1.33209	-0.88207
1	0	-2.33805	-3.15197	2.33058
1	0	-4.49739	-2.61974	1.23592

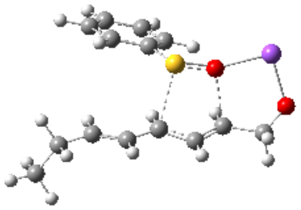

TSIIb-*exo*

E(M062X/BS1)$_{toluene}$ = -1254.21756157
G(correction)= 0.229743
E(M062X /BS2)$_{toluene}$ = -1254.45743924

Imaginary frequencies: 1 (-213.2856 cm^{-1})

6	0	-1.6288	-1.89051	0.19741
1	0	-1.57965	-2.77074	0.83853
6	0	-0.38318	-1.39044	-0.35624
6	0	-2.81101	-1.30684	-0.05451
6	0	0.81951	-2.08525	-0.38331
1	0	-0.47081	-0.51625	-1.00297
1	0	-2.8313	-0.43346	-0.7101
6	0	-4.13336	-1.78309	0.46168
1	0	0.91751	-3.00845	0.18765
6	0	1.95542	-1.44763	-0.84766
1	0	-4.61064	-0.97359	1.03
1	0	-3.98146	-2.61692	1.15587
6	0	-5.06647	-2.20759	-0.67732
1	0	1.83616	-0.56949	-1.48314
6	0	3.35755	-2.00484	-0.79093
1	0	-4.63338	-3.03883	-1.24184
1	0	-6.03795	-2.52626	-0.28807
1	0	-5.23654	-1.38069	-1.37478
1	0	3.37777	-2.69386	-1.67846
1	0	3.38053	-2.70739	0.07946
8	0	4.34344	-1.07865	-0.80529
11	0	4.07381	0.39427	0.63129
8	0	1.9567	0.08341	0.83065
16	0	0.5167	0.24529	1.40197
6	0	-0.2199	1.57736	0.49593
6	0	-1.52714	1.94753	0.82776
6	0	0.44866	2.20362	-0.55622
6	0	-2.15723	2.95708	0.10745
1	0	-2.04311	1.45204	1.6472

6	0	-0.19314	3.21332	-1.26888
1	0	1.45876	1.8908	-0.80566
6	0	-1.49312	3.59349	-0.94083
1	0	-3.17073	3.24849	0.36701
1	0	0.32534	3.70485	-2.087
1	0	-1.98747	4.38282	-1.49827

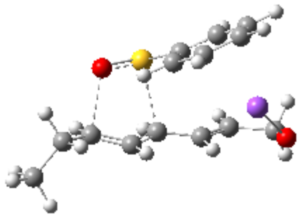

TSIIb-*endo*

E(M062X/BS1)$_{toluene}$ = -1254.21321991
G(correction)= 0.231439
E(M062X /BS2)$_{toluene}$ = -1254.44977101

Imaginary frequencies: 1(-233.1579 cm^{-1})

6	0	-1.8472	0.98639	-0.48248
1	0	-1.48875	0.6624	-1.4605
6	0	-0.91074	1.55051	0.40732
6	0	-3.04107	0.49924	-0.02154
6	0	0.42663	1.88942	-0.05099
1	0	-1.28175	2.05682	1.29732
16	0	-0.75986	-0.47697	1.72131
1	0	-3.42576	0.87734	0.92449
6	0	-4.00312	-0.29758	-0.84324
1	0	0.64914	1.64454	-1.09109
6	0	1.40373	2.45989	0.67094
8	0	-2.09954	-1.0577	1.24958
6	0	0.4963	-1.24715	0.72686
1	0	-4.30422	-1.17586	-0.26102
1	0	-3.50377	-0.66163	-1.74875
6	0	-5.24326	0.5211	-1.21815
1	0	1.20357	2.77556	1.69843
6	0	2.80463	2.6705	0.12391
6	0	1.83157	-1.16605	1.13932
6	0	0.15481	-1.93846	-0.43756
1	0	-5.94846	-0.08459	-1.79514
1	0	-5.76121	0.87883	-0.32216
1	0	-4.96946	1.39338	-1.81956
1	0	3.47962	2.36226	0.96482
1	0	2.93406	3.78166	0.10048
8	0	3.06824	2.06523	-1.0541
6	0	2.8224	-1.81704	0.40324
1	0	2.09331	-0.62017	2.04226
6	0	1.15374	-2.56607	-1.18301
1	0	-0.89266	-1.99994	-0.72028
11	0	2.40086	0.13251	-1.37208
6	0	2.48695	-2.5196	-0.76002
1	0	3.85528	-1.77429	0.73646
1	0	0.89015	-3.11617	-2.08168
1	0	3.25748	-3.0372	-1.32354

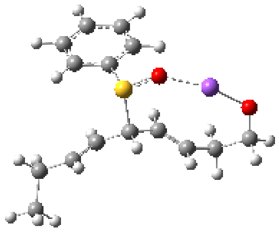

model **IIIa**

E(M062X/BS1)$_{toluene}$ = -1293.54532531
G(correction)= 0.258995
E(M062X /BS2)$_{toluene}$ = -1293.79311306

Imaginary frequencies: 0

6	0	-1.60622	-1.24464	-0.16333
1	0	-1.78666	-0.73008	-1.10696
6	0	-0.24986	-1.05211	0.44521
6	0	-2.57407	-1.9753	0.39358
6	0	0.85247	-0.89924	-0.55075
1	0	-0.0235	-1.85473	1.15746
16	0	-0.34951	0.44253	1.59275
1	0	-2.37447	-2.49007	1.33636
6	0	-3.93603	-2.18	-0.20059
1	0	0.71969	-0.12156	-1.30484
6	0	1.92243	-1.70314	-0.61234
8	0	1.08684	0.77235	1.98704
6	0	-0.83874	1.69995	0.40334
1	0	-4.69444	-1.80941	0.50151
1	0	-4.02832	-1.58595	-1.11676
6	0	-4.2104	-3.65657	-0.49897
1	0	2.02194	-2.49523	0.13738
6	0	3.02122	-1.61864	-1.622
6	0	-2.19674	1.90894	0.16669
6	0	0.13395	2.47223	-0.22304
1	0	-4.13771	-4.26084	0.4114
1	0	-5.2138	-3.79178	-0.9131
1	0	-3.48656	-4.04705	-1.22054
1	0	2.8531	-0.75595	-2.2794
1	0	3.01196	-2.52792	-2.24158
6	0	4.40989	-1.4713	-0.93328
6	0	-2.57962	2.88824	-0.74495
1	0	-2.94354	1.31522	0.68709
6	0	-0.26099	3.4525	-1.12956
1	0	1.1837	2.31294	0.005
8	0	4.50299	-0.38641	-0.13316
1	0	4.57942	-2.44356	-0.40172
1	0	5.15183	-1.48263	-1.76558
6	0	-1.61378	3.65622	-1.39304
1	0	-3.63361	3.05948	-0.93976
1	0	0.48976	4.05927	-1.62606
11	0	3.04198	0.3086	1.13095
1	0	-1.91816	4.42447	-2.09708

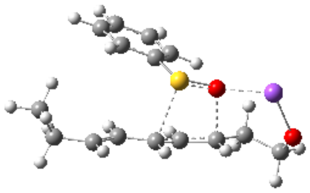

TSIIIa-*endo*

E(M062X/BS1)$_{toluene}$ = -1293.51606284
G(correction)= 0.256547
E(M062X /BS2)$_{toluene}$ = -1293.76597572

Imaginary frequencies: 1 (-244.1089 cm^{-1})

6	0	-1.65651	-1.51301	-0.84235
1	0	-1.94874	-0.76944	-1.58486
6	0	-0.24064	-1.59195	-0.51991
6	0	-2.59243	-2.28147	-0.26654
6	0	0.73411	-0.93629	-1.26055
1	0	0.06784	-2.37859	0.16714
1	0	-2.28282	-3.00992	0.48661
6	0	-4.05866	-2.21136	-0.56175
1	0	0.42322	-0.24504	-2.04316
6	0	2.03955	-0.89202	-0.79193
1	0	-4.24106	-1.46131	-1.33912
1	0	-4.39215	-3.1783	-0.96134
6	0	-4.8793	-1.88443	0.68994
1	0	2.36376	-1.66062	-0.09101
6	0	3.13939	-0.10058	-1.42419
1	0	-4.60934	-0.89987	1.08449
1	0	-5.94964	-1.87932	0.46401
1	0	-4.70506	-2.62361	1.47891
1	0	3.11412	0.92393	-1.01973
1	0	2.95976	-0.0185	-2.5061
6	0	4.52613	-0.70438	-1.08076
8	0	4.75566	-0.74786	0.25327
1	0	4.55232	-1.71056	-1.56709
1	0	5.27079	-0.10303	-1.65268
11	0	3.63518	0.13037	1.73174
8	0	1.58676	0.26763	0.98646
16	0	0.10159	0.04324	1.42366
6	0	-0.80471	1.30554	0.57705
6	0	-2.1758	1.41957	0.82356
6	0	-0.17906	2.12055	-0.36442
6	0	-2.92202	2.35501	0.11498
1	0	-2.65134	0.78256	1.56578
6	0	-0.93735	3.05637	-1.06464
1	0	0.88767	2.00984	-0.5354
6	0	-2.30559	3.17378	-0.83109
1	0	-3.98763	2.44643	0.30402
1	0	-0.45539	3.69529	-1.79894
1	0	-2.89065	3.90409	-1.38139

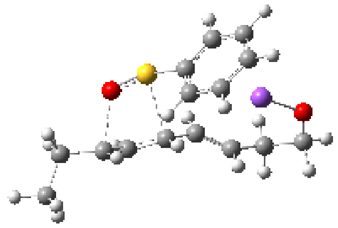

TSIIIa-*exo*

E(M062X/BS1)$_{toluene}$ = -1293.4982282
G(correction)= 0.257623
E(M062X /BS2)$_{toluene}$ = -1293.74469524

Imaginary frequencies: 1(-274.8039 cm^{-1})

6	0	2.33084	1.36188	-0.18316
1	0	2.6831	2.00463	-0.98888
6	0	0.95646	1.15282	-0.01598
6	0	3.18355	0.43898	0.37969
6	0	-0.12024	1.98267	-0.56357
1	0	0.68018	0.5871	0.87874
1	0	2.84582	-0.10402	1.26112
6	0	4.64704	0.35984	0.08529
8	0	2.40783	-1.25946	-0.74158
1	0	-0.03335	2.31433	-1.59849
6	0	-1.16282	2.38136	0.18341
1	0	4.85897	0.86449	-0.86348
1	0	4.91296	-0.6954	-0.04238
6	0	5.48635	0.97342	1.21112
16	0	1.05711	-0.85998	-1.35515
1	0	-1.17964	2.08018	1.2384
6	0	-2.31279	3.23192	-0.25917
1	0	6.55419	0.88066	0.9919
1	0	5.255	2.03551	1.33815
1	0	5.29384	0.47061	2.1647
6	0	-0.22204	-1.71438	-0.45377
1	0	-2.40248	3.18282	-1.3519
1	0	-2.12519	4.2818	0.0138
6	0	-3.62228	2.73396	0.40505
6	0	-1.3925	-2.05228	-1.14514
6	0	-0.08458	-2.03613	0.89949
8	0	-3.84044	1.409	0.21811
1	0	-3.53322	3.023	1.48408
1	0	-4.43733	3.38325	0.01331
6	0	-2.43253	-2.69893	-0.47232
1	0	-1.48492	-1.82402	-2.20448
6	0	-1.13154	-2.66996	1.567
1	0	0.84776	-1.80625	1.40891
11	0	-2.50883	-0.12838	0.36019
6	0	-2.30794	-2.99864	0.88667
1	0	-3.33643	-2.96693	-1.01112
1	0	-1.02778	-2.91635	2.61942
1	0	-3.1171	-3.49906	1.40862

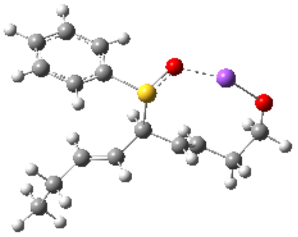

model **IIIb**

E(M062X/BS1)$_{toluene}$ = -1293.54477454
G(correction)= 0.258220
E(M062X /BS2)$_{toluene}$ = -1293.79317531

Imaginary frequencies: 0

6	0	-0.74081	1.66855	-0.6259
1	0	-0.54718	2.15777	-1.58116
6	0	0.26153	0.64845	-0.18601
6	0	-1.83027	1.98097	0.07783
6	0	1.68151	1.04059	-0.45776
1	0	0.12453	0.37223	0.86641
16	0	-0.07262	-0.95143	-1.12253
1	0	-2.00203	1.47867	1.03301
6	0	-2.8565	2.99553	-0.328
1	0	1.92777	1.29416	-1.49112
6	0	2.61457	1.16156	0.49445
8	0	0.96872	-1.93981	-0.59502
6	0	-1.60956	-1.39641	-0.31336
1	0	-3.82574	2.49137	-0.44321
1	0	-2.59449	3.4159	-1.30531
6	0	-2.99507	4.11324	0.70911
1	0	2.33627	0.92476	1.52638
6	0	4.03204	1.58481	0.28258
6	0	-2.817	-1.00824	-0.88735
6	0	-1.56134	-2.11738	0.87573
1	0	-2.05045	4.65287	0.8259
1	0	-3.76497	4.83047	0.41003
1	0	-3.27387	3.70756	1.68732
1	0	4.20207	2.54806	0.78668
1	0	4.22025	1.72297	-0.78984
6	0	5.02001	0.51878	0.84045
6	0	-4.00499	-1.33029	-0.23557
1	0	-2.83095	-0.45994	-1.82525
6	0	-2.75534	-2.43719	1.51483
1	0	-0.60001	-2.42986	1.27356
8	0	4.85617	-0.70424	0.29196
1	0	6.03624	0.95136	0.68602
1	0	4.87935	0.54057	1.95258
6	0	-3.97277	-2.03957	0.96328
1	0	-4.95461	-1.03208	-0.66886
1	0	-2.73623	-3.0012	2.4421
11	0	3.10199	-1.61801	-0.25564
1	0	-4.90114	-2.29267	1.46601

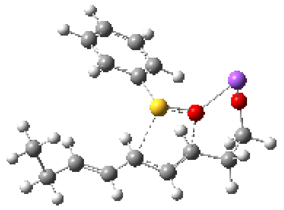

TSIIIb-*exo*

E(M062X/BS1)$_{toluene}$ = -1293.51386697
G(correction)= 0.257938
E(M062X /BS2)$_{toluene}$ = -1293.76204601

Imaginary frequencies: 1 (-213.2856 cm^{-1})

6	0	-2.10821	-1.99119	-0.37678
1	0	-2.20074	-2.95324	0.12759
6	0	-0.76557	-1.49115	-0.63108
6	0	-3.20681	-1.30212	-0.71715
6	0	0.39853	-2.25117	-0.52387
1	0	-0.7075	-0.56255	-1.2002
1	0	-3.08128	-0.33673	-1.21329
6	0	-4.61415	-1.72271	-0.4294
1	0	0.35423	-3.25198	-0.09603
6	0	1.60719	-1.59235	-0.64063
1	0	-4.61769	-2.72004	0.02418
1	0	-5.17511	-1.79286	-1.37042
6	0	-5.31462	-0.7204	0.49471
1	0	1.59559	-0.6151	-1.12112
6	0	2.97295	-2.1694	-0.45186
8	0	1.32552	-0.49956	1.26581
1	0	-5.32895	0.27955	0.04757
1	0	-6.34975	-1.01886	0.68541
1	0	-4.79481	-0.65185	1.45567
1	0	3.02162	-3.16514	-0.91766
1	0	3.15117	-2.31945	0.62439
6	0	4.04177	-1.19881	-1.0151
11	0	3.27386	0.46137	1.43544
16	0	-0.1718	-0.08832	1.37791
8	0	3.96807	0.0406	-0.46163
1	0	5.02704	-1.7016	-0.87818
1	0	3.88786	-1.1903	-2.12154
6	0	-0.29133	1.4303	0.46194
6	0	-1.50705	2.12043	0.49589
6	0	0.77157	1.89881	-0.31588
6	0	-1.6527	3.29223	-0.23888
1	0	-2.33042	1.74696	1.10147
6	0	0.61114	3.07872	-1.04101
1	0	1.70702	1.34221	-0.38411
6	0	-0.59323	3.7771	-1.00511
1	0	-2.59474	3.83164	-0.20852
1	0	1.43548	3.44463	-1.64625
1	0	-0.70845	4.6947	-1.57361

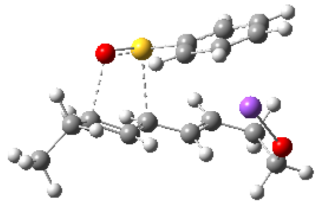

TSIIIb-*endo*

E(M062X/BS1)$_{toluene}$ = -1293.50862444
G(correction)= 0.259168
E(M062X /BS2)$_{toluene}$ = -1293.7549418

Imaginary frequencies: 1(-238.5004 cm^{-1})

6	0	1.73963	-1.1766	-0.39113
1	0	1.37191	-0.86743	-1.37143
6	0	0.79396	-1.54648	0.58449
6	0	3.01611	-0.83927	-0.02066
6	0	-0.59652	-1.80816	0.20913
1	0	1.16056	-2.0298	1.48924
1	0	3.41102	-1.24298	0.90957
6	0	4.01609	-0.20619	-0.93467
8	0	2.39551	0.83255	1.28755
1	0	-0.82048	-1.816	-0.8591
6	0	-1.59454	-2.04867	1.07506
1	0	3.50334	0.23083	-1.79966
1	0	4.50322	0.61415	-0.39549
6	0	5.07243	-1.21258	-1.40433
16	0	0.97413	0.52344	1.77261
1	0	-1.3561	-2.03296	2.14273
6	0	-3.01653	-2.3594	0.70802
1	0	5.81304	-0.72653	-2.04639
1	0	5.60133	-1.65181	-0.55219
1	0	4.61057	-2.02751	-1.97012
6	0	-0.09866	1.49401	0.73972
1	0	-3.66674	-1.59905	1.16948
1	0	-3.28703	-3.31436	1.18584
6	0	-3.31144	-2.40223	-0.80604
6	0	-1.43036	1.69246	1.12429
6	0	0.39848	2.07266	-0.43112
8	0	-3.12945	-1.21484	-1.43871
1	0	-2.67548	-3.22737	-1.21537
1	0	-4.35073	-2.79285	-0.89503
6	0	-2.26264	2.48984	0.33689
1	0	-1.80979	1.24239	2.03835
6	0	-0.44444	2.85811	-1.21959
1	0	1.44411	1.9213	-0.68482
11	0	-1.85892	0.3505	-1.25148
6	0	-1.77222	3.07318	-0.83617
1	0	-3.29389	2.65072	0.63572
1	0	-0.06149	3.31568	-2.127
1	0	-2.41986	3.69714	-1.4439

Representative examples of transition states structures showing geometric parameters related to the most important intramolecular interactions.

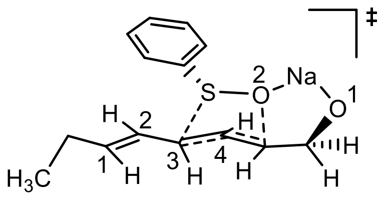

12.7, **TSIIa-*endo***

C^1-C^2-C^3-C^4: 169.3

Na-O^1: 2.07

Na-O^2: 2.15

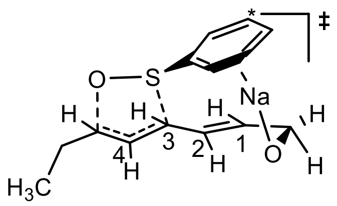

21.0, **TSIIb-*endo***

C^1-C^2-C^3-C^4: 178.2

Na-O: 2.07

Na-C*: 2.67

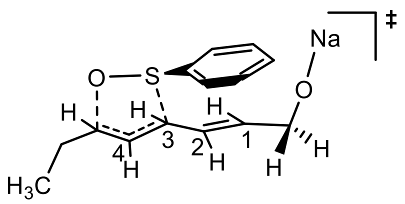

26.1, **TSIIb-*endo(2)***

C^1-C^2-C^3-C^4: -178.8

Na-O: 1.98

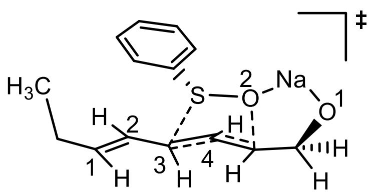

13.3, **TSIIa-*endo(1)***

C^1-C^2-C^3-C^4: 168.6

Na-O^1: 2.07

Na-O^2: 2.15

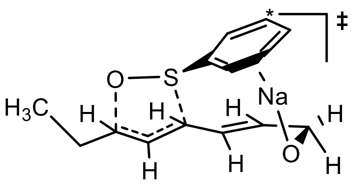

21.3, **TSIIb-*endo(1)***

C^1-C^2-C^3-C^4: 177.8

Na-O^1: 2.07

Na-C*: 2.66

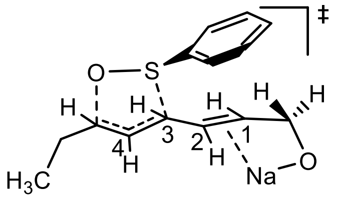

27.5, **TSIIb-*endo(3)***

C^1-C^2-C^3-C^4: 165.0

Na-O: 2.06

Na-C^1: 2.70

Figure S1. Spatial representation of different conformers of transition states **TSIIa** and **TSIIb** showing geometric parameters related with intramolecular interactions that determine their stability (M062XSMD(toluene)/6-311++G(d,p) //(M062XSMD(toluene)/6-31G (d). Relative G values at 298 K (kcal·mol^{-1}). Relevant distances (Å) and torsion angle (°) are indicated.
